# The terrestrial isopod symbiont ‘*Candidatus* Hepatincola porcellionum’ is a potential nutrient scavenger related to *Holosporales* symbionts of protists

**DOI:** 10.1038/s43705-023-00224-w

**Published:** 2023-03-08

**Authors:** Jessica Dittmer, Marius Bredon, Bouziane Moumen, Maryline Raimond, Pierre Grève, Didier Bouchon

**Affiliations:** 1 Dipartimento di Scienze Agrarie e Ambientali (DISAA), Università degli Studi di Milano, Via Celoria 2, 20133 Milano, Italy; 2 UMR 1345, Université d’Angers, Institut Agro, INRAE, IRHS, SFR Quasav, 42 Rue Georges Morel, 49070 Beaucouzé, France; 3 UMR CNRS 7267, Ecologie et Biologie des Interactions, Université de Poitiers, 3 Rue Jacques Fort, 86073 Poitiers, France; 4 Present Address: Université Paris-Sorbonne, Centre de Recherche Saint-Antoine, Equipe Microbiote, Intestin et Inflammation, 27 Rue Chaligny, 75012 Paris, France

**Keywords:** Microbial ecology, Phylogenomics, Comparative genomics

## Abstract

The order *Holosporales* (*Alphaproteobacteria*) encompasses obligate intracellular bacterial symbionts of diverse Eukaryotes. These bacteria have highly streamlined genomes and can have negative fitness effects on the host. Herein, we present a comparative analysis of the first genome sequences of ‘*Ca*. Hepatincola porcellionum’, a facultative symbiont occurring extracellularly in the midgut glands of terrestrial isopods. Using a combination of long-read and short-read sequencing, we obtained the complete circular genomes of two *Hepatincola* strains and an additional metagenome-assembled draft genome. Phylogenomic analysis validated its phylogenetic position as an early-branching family-level clade relative to all other established *Holosporales* families associated with protists. A 16S rRNA gene survey revealed that this new family encompasses diverse bacteria associated with both marine and terrestrial host species, which expands the host range of *Holosporales* bacteria from protists to several phyla of the Ecdysozoa (Arthropoda and Priapulida). *Hepatincola* has a highly streamlined genome with reduced metabolic and biosynthetic capacities as well as a large repertoire of transmembrane transporters. This suggests that this symbiont is rather a nutrient scavenger than a nutrient provider for the host, likely benefitting from a nutrient-rich environment to import all necessary metabolites and precursors. *Hepatincola* further possesses a different set of bacterial secretion systems compared to protist-associated *Holosporales*, suggesting different host-symbiont interactions depending on the host organism.

## Introduction

Numerous bacteria have evolved intricate and long-lasting associations with Eukaryotes, to the extent that some are no longer able to survive outside of their host organism. This strictly host-associated and often intracellular lifestyle has had profound impacts on the genomic evolution of these bacteria, resulting in some of the smallest bacterial genomes observed to date [1–3].

The orders *Rickettsiales* and *Holosporales* (*Alphaproteobacteria*) encompass obligate bacterial symbionts of diverse Eukaryotes. Historically, most research focussed on arthropod-vectored pathogens of vertebrates and humans (e.g., *Anaplasma* spp., *Ehrlichia* spp., *Orientia tsutsugamushi*, *Rickettsia* spp.) as well as *Wolbachia* spp., reproductive parasites of arthropods [4, 5]. However, this paradigm has shifted in recent years and it is now established that members of both orders are widespread symbionts of arthropods [6, 7], protists [8–16], corals [17], marine worms [18] and algae [19]. The appreciation of the ecological diversity of these bacteria has also led to the recent recognition of the *Holosporales* as a separate order, containing many taxa that had previously been considered basal or neglected *Rickettsiales* [20–22].

The majority of the known *Rickettsiales* and *Holosporales* are obligate intracellular bacteria and some even inhabit unusual intracellular niches: ‘*Ca*. Midichloria mitochondrii’ (*Rickettsiales*) occurs within the mitochondria of its tick hosts [23] and numerous members of the *Holosporales* infect the nuclei of amoebae (‘*Ca*. Nucleicultrix amoebiphila’) or ciliates (‘*Holospora* spp.’) [9, 24]. This obligate intracellular lifestyle has resulted in highly streamlined genomes with often reduced metabolic capacities. For instance, several pathways that are considered essential for most organisms across the tree of life (e.g., glycolysis, TCA cycle) are incomplete or missing in numerous members of the *Rickettsiales* and *Holosporales* [13, 14, 16, 17, 25, 26], highlighting their dependence on the host to scavenge nutrients and precursors. Therefore, these taxa represent ideal models to investigate evolutionary trajectories from free-living to strictly intracellular bacteria. Notably, the recent discovery of the first extracellular *Rickettsiales* (the ciliate episymbiont ‘*Ca*. Deianiraea vastatrix’) has motivated the new hypothesis that the *Rickettsiales* ancestor could have been an extracellular bacterium attached to the host surface via adhesion mechanisms [12]. Subsequently, intracellularity would have evolved multiple times independently from this ancestor, producing the different intracellular *Rickettsiales* families known today. The diversity of intracellular locations and cell entry mechanisms within the *Rickettsiales* lend further support to this hypothesis [4, 12].

A similar evolutionary trajectory may have been at work in the *Holosporales*. While most known members of this order are obligate intracellular or intranuclear symbionts of protists [9, 11, 14–16, 21, 24, 27–29], two extracellular taxa have been described to date: ‘*Ca*. Hepatincola porcellionum’, which occurs extracellularly in the lumen of the midgut caeca of terrestrial isopods [30, 31] and ‘*Ca*. Tenuibacter priapulorum’ from the gut of the marine worm *Priapulus caudatum* [18]. Both taxa are elongated rod-shaped bacteria associated with the microvilli on the surface of the midgut epithelium. Both were initially classified as *Rickettsiales*, but in recent 16S rRNA phylogenies, they form a family-level clade within the *Holosporales*, for which the name ‘*Ca*. Hepatincolaceae’ has been proposed [21]. To date, only 16S rRNA gene sequences are available for these bacteria, preventing deeper investigations of their evolutionary relationships, metabolic potential and host interactions.

‘*Ca*. H. porcellionum’ (hereafter *Hepatincola*) was first described in the common woodlouse *Porcellio scaber*, showing dense populations of bacteria with stalk-like appendages in the lumen of the midgut glands (hepatopancreas) [31]. These “stalks” were frequently inserted between the microvilli of the midgut epithelium although it remained unclear whether the bacteria were directly attached to the host cells. Since then, *Hepatincola* has been detected in several terrestrial isopod species, but infection rates in natural populations are highly variable [30, 32, 33], indicating that *Hepatincola* is a facultative symbiont for the isopod host, although the bacterium is likely not able to survive outside of a host organism. Its impact on host biology, be it beneficial or pathogenic, also remains to be elucidated. Based on the symbiont’s niche in the midgut glands of a host that feeds on recalcitrant food sources rich in lignocellulose, a nutritional role had initially been hypothesized ([30, 34, 35], reviewed in [36]). However, the only experiment investigating phenotypic effects observed an increased mortality in infected individuals of *P. scaber* [32].

Herein, we present the first complete genome sequences of *Hepatincola* symbionts from three terrestrial isopod host species to gain insights into their genetic diversity, metabolic potential and the interactions between the symbiont and its isopod host. Furthermore, we use available 16S rRNA sequences to investigate the diversity of the family ‘*Ca*. Hepatincolaceae’ across marine and terrestrial arthropod host species.

## Materials and methods

### Terrestrial isopod screening for *Hepatincola*

Twenty terrestrial isopod species were screened for the presence of *Hepatincola*. Most tested individuals came from population cages maintained by the UMR CNRS 7267 at the University of Poitiers (France), except for *Philoscia muscorum* and *Porcellionides pruinosus*, for which field-collected individuals from Ensoulesse (France) were also included (Supplementary Table S1). For four species, several populations could be tested, resulting in a total of 25 terrestrial isopod populations. One male and one female of each population were tested for the presence of *Hepatincola*. To this end, the midgut glands were dissected in Ringer solution under a stereomicroscope. As there are two pairs of midgut glands per individual, one pair was used for DNA extraction and diagnostic PCR, while the second pair was fixed for TEM. DNA was extracted using phenol-chloroform extraction and PCR was performed by amplification of the 16S rRNA gene using the *Hepatincola*-specific forward primer 137F (5’-ACACGTGGGAATTTGGCT-3’) in combination with the “universal” reverse primer 520R (5’- ATT-ACC-GCG-GCT-GCT-GG-3’) [37].

### Transmission electron microscopy (TEM)

Positive samples were further analyzed using TEM. The second pair of the midgut glands was fixed (3% glutaraldehyde in 0.1 M cacodylate buffer and 0.15 M NaCl (pH 7.4)) for 24 h at 4 °C. After washing with cacodylate buffer (pH 7.4) for 2 h at 4 °C, the tissues were post-fixed for 1 h in 1.3% OsO_4_ in 0.1 M cacodylate buffer and 0.3 M NaCl at room temperature. The samples were dehydrated in a graded acetone series and embedded in Epon® resin (Polysciences Inc, USA). Ultrathin sections (70 nm) were cut with an EMUC6 Leica ultramicrotome. The grids were contrasted with 2% uranyl acetate and lead citrate. Specimens were viewed using a Jeol JEM 1010 electron microscope with a Quemesa Olympus digital camera at the ImageUP facility at the University of Poitiers.

### Genome sequencing and assembly

#### Hepatincola symbiont of *Armadillidium vulgare*

The genome of the *Hepatincola* symbiont of *A. vulgare* was sequenced from a female isopod collected from a natural population in Availles-Thouarsais, France (46° 51’ 37” N, 0° 8’ 28” E) in 2014. *Hepatincola* was known to be present in this population from our previous metabarcoding study [33]. DNA was extracted from both pairs of midgut glands using phenol-chloroform extraction. In total, 4.5 µg of DNA were used for size selection using AMPure XP beads (Beckman Coulter, USA) at a bead:sample ratio of 0.7x to enrich in long fragments. In total, 3.5 µg of DNA were recovered and used for library preparation using the Oxford Nanopore Ligation Sequencing kit SQK-LSK 108 (Oxford Nanopore Technologies, UK). The library was sequenced on an R9.4 flowcell on the MinION sequencer for 58 h. The run was stopped and restarted several times to optimize pore use. Basecalling was done using Albacore v2.0.1 using a quality threshold of Q7. After discarding low quality (<Q7) and short (<500 bp) reads, we obtained 1,915,174 reads with an average quality of 13.2 and average length of 3.5 kb (range: 0.5–66 kb). Reads belonging to the isopod host were removed after mapping against the genome scaffold of *A. vulgare* (Accession: GCA_004104545.1) using Minimap2 v2.15 [38], resulting in 1,083,710 reads. The reads ≥1 kb were assembled using Canu v1.7 [39], producing a 1.37 Mbp circular contig containing two 16S rRNA genes 99% identical to the 16S rRNA gene sequence of *Hepatincola* from *P. scaber* (Accession: AY188585). This initial assembly was first polished with Nanopore reads using Nanopolish v0.11.1 [40] and subsequently with Illumina reads using Racon v1.4.3 [41]. Additional Illumina polishing with Polypolish v0.4.3 [42] did not detect additional errors. Illumina reads mapping onto the *Hepatincola* genome were extracted from *A. vulgare* shotgun metagenomic datasets from our previous study [43], in which DNA from different tissues (including the midgut glands) of the same *Hepatincola*-infected individual had been included.

#### Hepatincola symbiont of *Porcellio dilatatus petiti*

The genome of the *Hepatincola* symbiont of *P. dilatatus petiti* was assembled from Illumina shotgun metagenomic datasets from our previous study [44]. Specifically, we used two datasets corresponding to the metagenomes of pooled midgut glands from seven *P. dilatatus petiti* males and females, respectively. After removal of potential host reads by mapping against a custom database containing all isopod sequences available on NCBI and unpublished sequences produced by the laboratory UMR CNRS 7267, the remaining reads were assembled using Megahit v1.0.3 [45] with custom parameters (*--min-count 2 --k-min 21 --k-max 127 --k-steps 1*). Contigs greater than 1500 bp were grouped into bins using MetaBAT2 v2.12.1 [46]. Bin quality was checked with CheckM v1.0.13 [47] and only bins with a completeness >80% and a contamination rate <5% were retained. RNAmmer v1.2 [48] was used to predict ribosomal RNAs for each bin, which led to the detection of two partial 16S rRNA genes with >99% identity to the 16S rRNA gene sequence of *Hepatincola* from *P. scaber* (Accession: AY188585) in a bin from the midgut glands of female *P. dilatatus petiti*. The raw reads were mapped onto the contigs of the bin using BOWTIE2 v2.2.9 [49] with the --very-sensitive option. Mapped reads were *de novo* assembled using SPAdes v3.13.0 [50] with the --careful option. This produced 66 contigs with a combined length of 1.27 Mbp. The contigs were ordered using Mauve v2.4.0 [51] using the complete *Hepatincola* genome from *A. vulgare* as a reference. This resulted in a genome scaffold of 1,229,614 bp.

#### Hepatincola symbiont of *Porcellionides pruinosus*

The genome of the *Hepatincola* symbiont of *P. pruinosus* was sequenced from tissues of 45 females from a laboratory lineage established from a natural population sampled in Nevers, France (46° 59’ 27” N, 3° 9’ 46” E) in 1996. DNA was extracted from crushed tissues using the bacterial enrichment procedure from Badawi et al. [52]. Briefly, tissues were homogenized with a Dounce tissue grinder B in a PBS solution containing sucrose (0.25 M) and L-glutamine (5 mM) to crush cells while keeping the nuclei intact. The homogenate was centrifuged for 15 min at 200 × *g* and 4 °C to pellet cell nuclei. The supernatant was centrifuged for 15 min at 4100 × *g* and 4 °C. DNA was extracted from the resulting pellet using the DNeasy Blood and Tissue kit (QIAGEN, Hilden, Germany). RNA contaminants were removed with RNase A treatment (2.5 μg/μl in the final reaction at 20 °C for 5 min). In total, 1.2 µg of genomic DNA was used for library preparation using the Oxford Nanopore Ligation Sequencing kit SQK-LSK 109 (Oxford Nanopore Technologies, UK). The library was sequenced on an R9.4 flowcell on the MinION sequencer for 25 h. Basecalling was done using GUPPY v4.4.2 in high accuracy mode (Oxford Nanopore Technologies, UK) and trimming was performed using Porechop v0.2.4 [53] using the default parameters. In total, 2,151,363 reads were obtained with an average quality of 14 and average length of 2 kb (range: 0.5–141 kb). Reads were then *de novo* assembled using Flye v2.8 [54] in metagenome mode with an overlap of 1 kb, which produced a 1.29 Mbp circular contig containing two 16S rRNA genes with 97% identity to the 16S rRNA gene sequence of *Hepatincola* from *P. scaber* (Accession: AY188585). This initial assembly was first polished with Nanopore reads using Medaka v1.6.0 (https://github.com/nanoporetech/medaka) and subsequently with Illumina reads using Polypolish v0.4.3 [42]. Illumina reads were generated from the midgut glands of 30 individuals from the same laboratory lineage. Sequencing was performed on a HiSeq 2500 (Macrogen, Amsterdam, the Netherlands) resulting in 14.9 Gb of reads.

### Genome annotation and curation

All genomes were annotated using Prokka v1.14.6 [55]. The annotation of the *Hepatincola* genome from *A. vulgare* was also manually curated using Pfam [56], CD-Search [57] and BlastP [58]. This curated genome was then used to improve the annotations of the other two genomes (option --proteins for Prokka). Remaining frameshifts were corrected manually by inspecting the alignment of short reads against the genomes using Artemis [59]. BUSCO [60] was used to assess the completeness of the genomes based on the proteobacteria_odb10 dataset.

### Functional annotation

Synteny plots of conserved genes were created using Synima (Synteny Imager) (https://github.com/rhysf/Synima). A pangenome analysis of the three *Hepatincola* strains was performed using Anvi’o [61]. Clusters of Orthologous Genes (COG) categories were determined using eggNOG-mapper v2.1.7 [62] and KEGG pathway annotations were obtained using BlastKOALA v2.2 [63]. MacSyFinder 2.0 implemented in the tool TXSScan (galaxy.pasteur.fr, [64]) was used to identify bacterial secretion systems in the *Hepatincola* genomes as well as in all published *Holosporales* genomes. Signal peptides were identified using SignalP 6.0 [65] and transmembrane transporters were predicted using TransportDB 2.0 [66]. antiSMASH [67] was used to identify secondary metabolite synthesis gene clusters. Carbohydrate active enzymes (CAZymes) were identified using the CAZy database [68] and dbCAN2 was used to determine CAZy families [69]. Only CAZymes identified by at least two of the three tools integrated in dbCAN2 (i.e., Hotpep, Diamond, HMMER) were retained. Prophage regions were identified using Phaster [70] and the homology of prophage genes between the *Hepatincola* strains was verified using reciprocal BlastP searches. Circular genome plots were created using the CGView web server (https://proksee.ca/).

### Phylogenomics

Orthofinder v2.5.2 [71] was used to identify single-copy orthologous genes shared between *Hepatincola* and 44 representatives of all recognized families of the *Holosporales* and *Rickettsiales*. Only complete genomes were used, unless when no complete genome was available for a particular taxon. The quality of the selected genomes was checked using BUSCO [60] (proteobacteria_odb10 dataset) and draft genomes with high duplication rates were excluded from the analysis. The amino acid sequences of each conserved gene were aligned using Muscle v3.1.31 [72] and the alignments were concatenated into a partitioned supermatrix using the script geneStitcher.py (https://github.com/ballesterus/Utensils/blob/master/geneStitcher.py). IQ-TREE v1.6.1 [73] was used to predict the optimal amino acid substitution model for each gene partition [74, 75] and to produce a Maximum Likelihood phylogenetic tree with 1000 bootstrap iterations. The tree was visualized in FigTree v1.4.4 (https://github.com/rambaut/figtree).

### 16S rRNA gene database screening

To investigate the diversity, host range and environmental distribution of taxa belonging to the ‘*Ca*. Hepatincolaceae’ family, we downloaded all 47 16S rRNA gene sequences assigned to this family from the SILVA database version SSU r138.1 [76] (https://www.arb-silva.de/). The sequences were downloaded as ARB alignment and duplicates were removed. The 16S rRNA gene sequences of the three *Hepatincola* strains were manually added to the alignment, as were seven representatives of the *Caedimonadaceae* and *Paracaedibacteraceae* as outgroup. All-gap sites were removed manually. A Maximum Likelihood phylogenetic tree based on the GTR + F + R3 model was produced using IQ-TREE v1.6.1 [73] with 1000 bootstrap replicates for internal branch support. The tree was visualized in iTOL v6.5.8 [77].

## Results

### *Hepatincola* host range and ultrastructural characterization

Terrestrial isopods from 25 populations representing 20 species and 7 families were screened for the presence of *Hepatincola* (Supplementary Table S1). Despite a shallow screening of only two individuals per population, the symbiont was detected in eight species. In five species (*Armadillidium granulatum*, *A. versicolor*, *Porcellio dilatatus*, *Porcellionides pruinosus* and *Orthometopon planum*), the symbiont was detected for the first time, extending the known host range of *Hepatincola* to ten species from five terrestrial isopod families (Table 1).

**Table 1:** Terrestrial isopod species tested positive for *Hepatincola*.

Family	Genus	Species	Origin	Sampling site	Reference
Armadillidiidae	*Armadillidium*	*granulatum*	Lab	Kolymbari, Greece	This study
	*Armadillidium*	*versicolor*	Lab	Sankt Veit, Austria	This study
	*Armadillidium*	*vulgare*	Lab	Helsingør, Denmark	This study
	***Armadillidium***	***vulgare***	**Field**	**Availles-Thouarsais, France**	[33, 37]
	*Armadillidium*	*vulgare*	Field	Helgoland, Germany	[32]
Oniscidae	*Oniscus*	*asellus*	Lab	Borrisokane, Ireland	This study
	*Oniscus*	*asellus*	Field	Kiel, Germany	[30, 32]
	*Oniscus*	*asellus*	Field	Köln, Germany	[30]
Philosciidae	*Philoscia*	*muscorum*	Field	Ensoulesse, France	This study
	*Philoscia*	*muscorum*	Field	Kiel, Germany	[32]
Porcellionidae	***Porcellio***	***dilatatus***	**Lab**	**St-Honorat, France**	This study
	*Porcellio*	*scaber*	Field	Kiel, Germany	[31, 32]
	*Porcellio*	*scaber*	Field	Köln, Germany	[30]
	*Porcellio*	*scaber*	Field	Haines Island, Canada	[30]
	*Porcellio*	*scaber*	Field	Vancouver Island, Canada	[30]
	***Porcellionides***	***pruinosus***	**Field**	**Ensoulesse, France**	This study
Trachelipodidae	*Orthometopon*	*planum*	Lab	Sainte-Marguerite, France	This study
	*Trachelipus*	*rathkii*	Field	Kiel, Germany	[29]

The populations used for genome sequencing are indicated in bold.

**Fig. 1:**
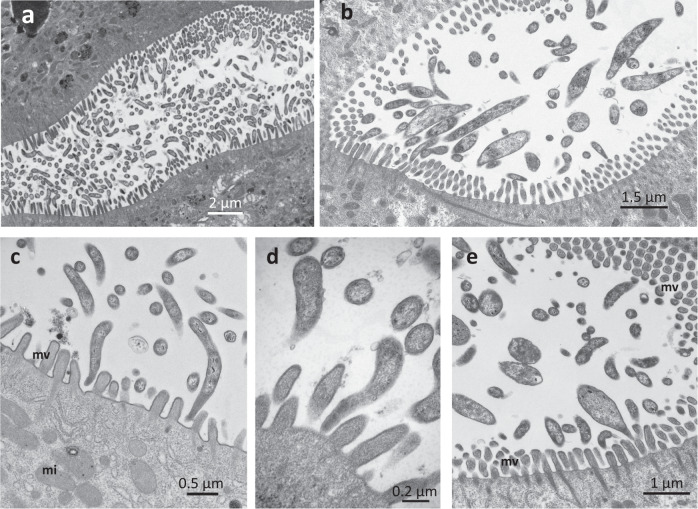
Extracellular bacteria in the midgut glands of terrestrial isopods positive for *Hepatincola*. TEM micrographs of extracellular bacteria in the midgut glands of *P. pruinosus* (**a**, **c**, **d**) and *O. planum* (**b**, **e**). **a**, **b** Large views showing dense bacterial populations in the lumen of the midgut glands of *P. pruinosus* (**a**) and *O. planum* (**b**), often in close vicinity to the midgut epithelium (**b**). The bacteria often form stalk-like protrusions at one cell pole, which is inserted between the microvilli of the epithelium (**c**–**e**). mi mitochondrion, mv microvilli. Scale bars: **a** = 2 µm, **b** = 1.5 µm, **c** = 500 nm, **d** = 200 nm, **e** = 1 µm.

TEM observations of the midgut glands of positive individuals revealed dense bacterial populations in the lumen of the organ (Fig. 1a, b). As previously described for the *Hepatincola* symbiont of *P. scaber* [31], the bacteria were rod-shaped, with one cell pole often elongated and inserted between the microvilli brush border of the midgut epithelium (Fig. 1c, d). The bacteria had a typical gram-negative cell wall (Fig. 1d) and electron-dense inclusions in the cytoplasm (Fig. 1b–d).

### *Hepatincola* genome features

The ONT long-read sequencing technology produced two complete circular chromosomes for the *Hepatincola* symbionts of *A. vulgare* (hereafter *HepAv*) and *P. pruinosus* (hereafter *HepPp*). An additional draft genome was obtained for *Hepatincola* of *P. dilatatus petiti* (hereafter *HepPdp*) (Table 2). *HepAv* had the largest genome with 1.34 Mbp, 1187 CDS and 29.53% GC content (Table 2). *HepPp* had a 1.28 Mbp genome with 1139 CDS and 31.62% GC. The draft genome of *HepPdp* was the smallest with 1.23 Mbp, 1104 CDS, and a GC content of 29.42% but this is due to some missing genome content. These genome sizes are quite typical for *Rickettsiales* but rather intermediate for *Holosporales*, whose genomes range from 0.59–2.85 Mbp (Supplementary Table S2). The two complete genomes further contained only one pseudogene, two ribosomal RNA operons, 32 tRNAs and 1–2 CRISPR arrays (Table 2). No plasmids were detected. Average nucleotide identity (ANI) was 95.70% between *HepAv* and *HepPdp*, 83.24% between *HepAv* and *HepPp* and 83.56% between *HepPdp* and *HepPp*. Considering the typical threshold of 95% ANI to define a bacterial species, *HepPp* might represent a different species from *HepAv* and *HepPdp*. On the other hand, the 16S rRNA genes of *HepAv* and *HepPp* were 97.42% identical, suggesting that both strains belong to the same species.

**Table 2:** Properties of the three *Hepatincola* genomes.

	*Hepatincola* Av	*Hepatincola* Pdp	*Hepatincola* Pp
Host species	*Armadillidium vulgare*	*Porcellio dilatatus petiti*	*Porcellionides pruinosus*
Sequencing method	Nanopore + Illumina	Illumina	Nanopore + Illumina
Assembly level	Complete	Scaffold	Complete
Length (bp)	1,340,442	1,229,614	1,284,103
%GC	29.53	29.42	31.62
Genes	1229	1139	1181
Protein-coding genes	1187	1104	1139
Pseudogenes	1	0	1
rRNA	6	2	6
tRNA	32	30	32
ncRNA	3	3	3
Crispr	2	0	1

**Fig. 2:**
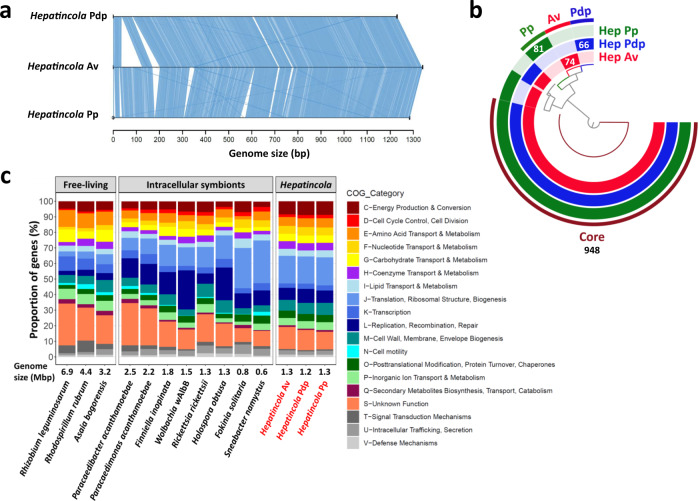
Comparison of the three *Hepatincola* genomes. **a** Synteny plot showing that the synteny is highly conserved between the three genomes. **b** Pangenome analysis showing that the three *Hepatincola* strains share a large core genome. Numbers correspond to the number of gene clusters making up the core genome and the strain-specific accessory genomes. **c** Comparison of COG categories between the three *Hepatincola* strains and other *Alphaproteobacteria*, either free-living or intracellular symbionts of arthropods or protists. Genome sizes for each bacterium are provided under the barplot. The three *Hepatincola* genomes are indicated in red.

Synteny was highly conserved between the three genomes (Fig. 2a), except for an 89 Kbp region that is present in both *HepAv* and *HepPp* (from locus tags HAV_00030/HPPR_00041 to HAV_00109/HPPR_00118) but absent from *HepPdp*. It is possible that this is due to a gap in the *HepPdp* assembly, as the missing region starts with a ribosomal RNA operon in *HepAv* and *HepPp* and the ribosomal RNA operons were largely missed in the *HepPdp* draft genome. A pangenome analysis based on orthologous gene clusters showed that the three strains share a large core genome, as 948 out of 1322 gene clusters (71.7%) were shared between all three genomes, 153 gene clusters were shared between any two strains and only 66–81 gene clusters were specific to a given strain (Fig. 2b). These corresponded mostly to hypothetical proteins. Moreover, the gene clusters specific for *HepAv* included numerous components of Type IV secretion systems, specifically *virB* family proteins (see detailed section on secretion systems below). The gene clusters specific for *HepPp* included genes coding for metallopeptidases and *recD*-like DNA helicases. Interestingly, the gene clusters specific for *HepPdp* contained two genes annotated as HigB-like toxins and two transcriptional regulators adjacent to the toxins, respectively. However, it is unlikely that these genes code for functional toxins as HigB toxins are part of HigB-HigA toxin-antitoxin systems. Not only do these generally occur on plasmids, but the HigA antitoxin is missing in *HepPdp*. Hence, a functional toxin without its antitoxin would kill the bacterium.

The genomes of the three *Hepatincola* strains were also similar in terms of broad functional categories, as classifying all protein-coding genes into clusters of orthologous genes (COGs) revealed that the three strains had highly similar proportions of each functional category (Fig. 2c). Although *Hepatincola* is not an intracellular symbiont, its functional profile was overall more similar to those of intracellular symbionts of arthropods and protists with reduced genomes than to those of free-living *Alphaproteobacteria* (Fig. 2c). Notably, the COG categories “Amino acid transport & metabolism” and “Transcription” tended to be less represented in *Hepatincola* as well as in intracellular symbionts compared to free-living taxa, whereas the categories “Translation, ribosomal structure, biogenesis” and “Replication, recombination, repair” were more represented in *Hepatincola* and intracellular symbionts.

### Prophage regions

**Fig. 3:**
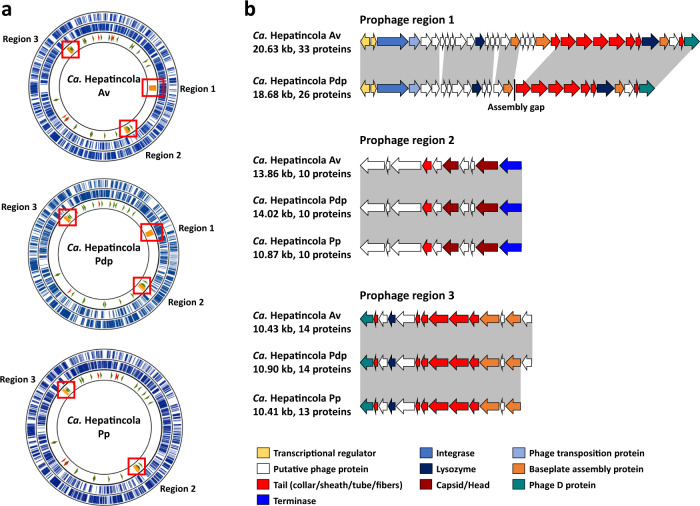
Prophage regions in *Hepatincola* genomes. **a** Circular genome plots showing the positions of the three prophage regions (orange). Outer circles represent protein-coding genes on both the forward and complement strands (blue). The inner circle indicates rRNA genes (red), tRNAs (green) and prophage regions (orange). **b** Schematic representation of the genes within the prophage regions. Shaded areas indicate homology between the different strains.

Three regions of prophage origin were identified based on the genome annotation and Phaster [70] (Fig. 3 and Supplementary Table S3). The first region (based on its position in the genome) was present only in *HepAv* and *HepPdp*. In *HepPp*, only three genes with homology to genes from this region were found scattered throughout the genome (Supplementary Table S3). This prophage region consisted in 33 proteins in *HepAv* and 26 proteins in *HepPdp*. The missing genes in *HepPdp* are due to an assembly gap within the prophage region (Fig. 3). This region contained transcriptional regulators, an integrase and phage transposition protein, two genes coding for lysozymes, several genes for phage baseplate and tail assembly as well as a phage late control D protein (Fig. 3 and Supplementary Table 3). Phaster also identified phage attachment sites flanking this region in both *Hepatincola* strains.

The other two prophage regions comprised 10 and 13–14 genes, respectively, and were present in all three *Hepatincola* strains (Fig. 3 and Supplementary Table 3). Prophage region 2 contained a phage tail tubular protein A, a putative major capsid protein and a head-tail connector protein. Prophage region 3 contained several genes for baseplate and tail assembly, a lysozyme and a phage late control D protein. No phage attachment sites were identified for regions 2 and 3.

Prophage gene taxonomic identification based on the Phaster database yielded highly variable best blast hits, preventing us from identifying the potential sources of the prophage regions. Nonetheless, the fact that region 1 was not present in all strains, contained mobile elements (integrase and transposase) and presented phage attachment sites suggests that this region might be of more recent origin than the other two.

### ‘*Ca*. Hepatincolaceae’ form a diverse and early-branching family within the Holosporales

**Fig. 4:**
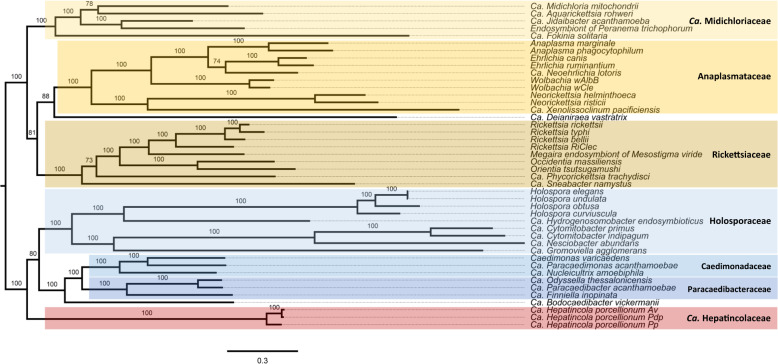
Phylogenetic placement of *Hepatincola* within the *Holosporales*. Maximum likelihood tree based on the concatenated amino acid sequence alignment of 62 single-copy orthologous genes from 44 representatives of the orders *Holosporales* (blue) and *Rickettsiales* (yellow). The three *Hepatincola* strains are highlighted in red. Branch support is based on 1000 bootstrap iterations.

A maximum likelihood phylogenomic analysis based on 62 single-copy genes present in 44 genomes (3 *Hepatincola* strains, 16 *Holosporales* and 25 *Rickettsiales* (Supplementary Table 2)) recovered all recognized families within the *Holosporales* and *Rickettsiales* (Fig. 4). Within the *Holosporales*, the *Holosporaceae* formed a sister group to the families *Caedimonadaceae* and ‘*Ca*. Paracaedibacteraceae’. *Hepatincola* was placed in an early-branching position relative to all three families, with full bootstrap support (BS: 100) and in agreement with recent 16S rRNA gene-based phylogenies [16, 78]. Hence, our genome-scale analysis validates the proposal of a new family ‘*Ca*. Hepatincolaceae’ within the order *Holosporales* [21]. The phylogenetic tree further supported the close relatedness between *HepAv* and *HepPdp*, whereas *HepPp* was more divergent (Fig. 4), suggesting that the genus *Hepatincola* might contain several bacterial species associated with terrestrial isopods.

**Fig. 5:**
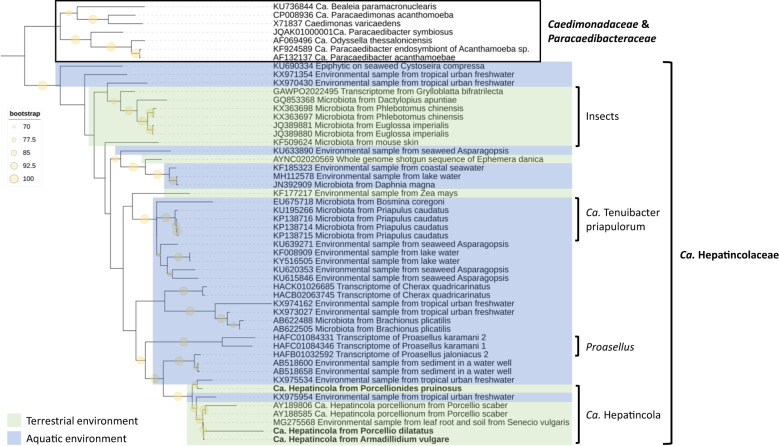
Diversity and environmental distribution of taxa within the family ‘*Ca*. Hepatincolaceae’. Maximum likelihood tree based on the 16S rRNA gene sequences of 45 taxa assigned to the family ‘*Ca*. Hepatincolaceae’. Seven strains from the *Caedimonadaceae* and *Paracaedibacteraceae* were used as outgroup. Branch support is based on 1000 bootstrap iterations, only bootstraps ≥70 are shown. Sequences of aquatic origin are highlighted in blue, those of terrestrial origin in green. The 16S rRNA gene sequences from the three *Hepatincola* genomes are indicated in bold.

To investigate the diversity, host range and environmental distribution of taxa belonging to the ‘*Ca*. Hepatincolaceae’ family, we performed another phylogenetic analysis based on the 16S rRNA gene sequences from the three *Hepatincola* genomes and 42 closely-related 16S rRNA gene sequences retrieved from the SILVA database (Fig. 5). These sequences came from various host organisms of the Ecdysozoa (i.e., the terrestrial isopod *P. scaber*, freshwater isopods, decapods, marine invertebrates and insects), but also from environmental sources (i.e., freshwater, seawater, seaweed, soil, mouse skin) (Supplementary Table S4). Of course, this does not mean that the bacteria were necessarily free-living in these environments, they might have been host-associated but the precise host organism is unknown. The three *Hepatincola* strains from this study formed a genus-level clade (>95% sequence similarity) with the *Hepatincola* strain from *P. scaber*, but also with three sequences from environmental samples (soil and urban freshwater) (Fig. 5). The next most closely-related bacteria (86–93% sequence similarity with the *Hepatincola* strains) came from freshwater isopods of the genus *Proasellus* (Fig. 5). Overall, the majority of the sequences (30/45) were of aquatic origin, either from aquatic host organisms or environmental sources (Fig. 5). Most of these taxa have not been previously described, except for ‘*Ca*. Tenuibacter priapulorum’, the extracellular gut symbiont of the marine worm *Priapulus caudatus* [18]. Apart from *Hepatincola*, only seven sequences came from terrestrial arthropods and these represented diverse insects (orchid bees, sand flies, scale insects, rock crawlers and mayflies). All but one of the insect-derived sequences formed a well-supported clade (Fig. 5), indicating potential insect-associated genera within the family ‘*Ca*. Hepatincolaceae’.

### *Hepatincola* has limited metabolic capacities but many transmembrane transporters

**Fig. 6:**
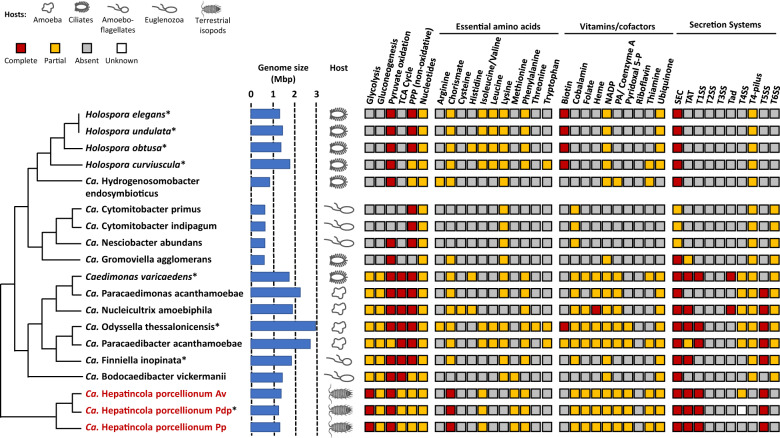
Metabolic capacity of *Hepatincola* compared to other *Holosporales*. KEGG pathway comparison shows that *Hepatincola* has limited metabolic capacities different from other *Holosporales* families, notably regarding glycolysis, biosynthesis of essential amino acids, vitamins and cofactors as well as secretion systems. Genome sizes and host organisms are indicated. Complete, partial, absent or unknown (due to missing genomic region in the *HepPdp* draft genome) KEGG pathways are indicated in red, orange, gray and white respectively. Asterisks indicate draft genomes. Phylogenetic relationships are represented based on Fig. 4 and not to scale. PPP pentose phosphate pathway, PA pantothenic acid, Pyridoxal 5-P pyridoxal 5-phosphate, TxSS type x secretion system.

Assigning all protein-coding genes to KEGG pathways (Supplementary Table S5) revealed that *Hepatincola* has a reduced metabolism reminiscent of other *Holosporales* bacteria [14, 16, 26] (Fig. 6). All three strains have a complete glycolysis pathway, a unique feature among the sequenced *Holosporales* bacteria (Fig. 6), which have either reduced glycolysis pathways (*Caedimonadaceae, Paracaedimonadaceae*) or completely lost this pathway (*Holosporaceae*) [14, 26]. One reaction is missing for gluconeogenesis (namely the Fructose-1,6-bisphosphatase fbp), therefore it is uncertain whether this pathway is functional. *Hepatincola* retains the pyruvate dehydrogenase complex and is therefore able to produce acetyl-CoA from pyruvate. The TCA cycle is truncated: whereas the first carbon oxidation from oxaloacetate to 2-oxoglutarate and the reactions from succinate to oxaloacetate are present, the steps in between (2-oxoglutarate dehydrogenase and succinyl-CoA synthetase) are missing. Like most *Holosporales*, *Hepatincola* retains only the non-oxidative phase of the pentose phosphate pathway, except for the transaldolase tal, a gene that is also missing from ‘*Ca*. Bodocaedibacter vickermanii’. *Hepatincola* is able to convert ribose-5-phosphate to phosphoribosyl pyrophosphate and possesses complete gene sets for oxidative phosphorylation complexes I, II and IV as well as for F-type ATPase and key cell membrane components such as fatty acids, lipopolysaccharides and peptidoglycan. Apart from these, *Hepatincola* has very limited biosynthetic capacities (Fig. 6). For instance, it is likely not able to synthesize nucleotides as many genes involved in nucleotide metabolism and biosynthesis are missing, including the nucleoside-diphosphate kinase ndk that is required to convert nucleoside diphosphates to nucleoside triphosphates. Similarly, most amino acid, vitamin and cofactor biosynthesis pathways are missing, except for a complete Shikimate pathway for the production of chorismate (Fig. 6). Furthermore, antiSMASH [67] did not identify any secondary metabolite synthesis gene clusters. Proteins containing ankyrin repeat domains that might be involved in protein-protein interactions with the host are also scarce (*N* = 2 in each *Hepatincola* strain). In contrast, several CAZymes (7–10 per genome) were identified (Supplementary Table S6), including 5 glycosyltransferases (GT), 2 carbohydrate esterases (CE) and 2 glycoside hydrolases (GH). Among them, only the GH3 family could potentially contribute to the degradation of lignocellulose, the main nutrient source of terrestrial isopods. However, only a single gene belonging to the GH3 family was present in each *Hepatincola* strain and its relatively low similarity to known proteins (57% amino acid sequence identity) precluded a more precise functional prediction.

To compensate for its limited metabolic capacities, *Hepatincola* possesses a large number of transmembrane transporters, specifically 110, 115 and 117 in *HepPdp, HepPp* and *HepAv*, respectively (Supplementary Table S7). Some of these are associated with the transport of amino acids, metabolites, sugars and nucleosides, e.g., ATP-binding cassettes (ABC, *N* = 30–37 depending on the *Hepatincola* strain), the major facilitator superfamily (MFS, *N* = 9–11), sodium ion:proton antiporters (*N* = 7–8), sugar specific PTS (*N* = 4–5), drug/metabolite transporters (DMT, *N* = 4–5), multidrug/oligosaccharidyl-lipid/polysaccharide (MOP) flippase (*N* = 3), proton/sodium ion:glutamate/aspartate symporters (*N* = 2) and a sodium ion:nucleoside symporter of the Concentrative Nucleoside Transporter (CNT) family (*N* = 1). In contrast, we did not identify any tlc ADP/ATP translocases, which are thought to enable the import of ATP from the host cytosol in several *Rickettsiales* and *Holosporales* bacteria [12, 14–17]. Each strain also has 3–4 porins and four beta barrel-containing outer membrane proteins, which allow the diffusion of larger molecules in and out of the cell.

*Hepatincola* also possesses several protein export and secretion systems (Fig. 6 and Supplementary Table S8). Both the Tat and Sec translocons are present in all three strains. However, no Tat signal peptides were detected by SignalP 6.0 [65] (Supplementary Table 9), suggesting that this translocon may no longer be functional. The Sec translocon likely functions primarily for co-translational translocation, as the Signal Recognition Protein and its receptor are present but not the preprotein translocase secB, which is necessary for post-translational translocation. All three strains further possess a complete Type I secretion system and an additional tolC outer membrane protein, which could function as a two-step secretion system in combination with the Sec translocon, as in *Rickettsia* [79]. All components for a *Rickettsia*-type Type V secretion system [79] are also present, i.e., the Sec translocon, the chaperone surA, the outer membrane protein assembly proteins bamA/B/D/E, the outer membrane protein skp/ompH and an autotransporter beta-domain protein (Supplementary Table S8). In addition, *HepAv* possesses a reduced Type IV secretion system without a pilus (Supplementary Table S8). This secretion system is absent from the complete genome of *HepPp* but could be present in the closely-related *HepPdp*. However, the corresponding genome region is missing from the *HepPdp* draft assembly. This set of protein secretion systems is different from other *Holosporales*, most of which possess a Sec translocon, incomplete Type IV pili and reduced but potentially functional Type VI secretion systems (Fig. 6). In addition, Type I, Type IV and/or Type V secretion systems as well as tad (tight adherence) pili are present in some *Caedimonadaceae* and *Paracaedimonadaceae* (Fig. 6).

## Discussion

Here we present a comparative analysis of the first genome sequences of ‘*Ca*. Hepatincola porcellionum’, a bacterium occurring in the lumen of the midgut glands of terrestrial isopods [30, 31]. A screening of different host species from diverse origins confirmed its facultative character for the host and extends the known host range of this symbiont to five terrestrial isopod families. Our phylogenomic analysis supported its phylogenetic position as an early-branching family-level clade relative to all other established *Holosporales* families associated with protists. Hence, this work provides the first genomic insights into a member of the recently proposed family ‘*Ca*. Hepatincolaceae’ [21]. However, considering that the *Holosporales* present high and heterogeneous evolutionary rates that complicate phylogenetic analyses [22], this phylogenetic placement may be revised when additional genomes of other ‘*Ca*. Hepatincolaceae’ genera become available. Furthermore, our 16S rRNA gene survey revealed that this new *Holosporales* family encompasses diverse bacteria associated with both marine and terrestrial host species, i.e., marine worms, aquatic and terrestrial arthropods. This greatly extends the host range of *Holosporales* bacteria from protists (mainly amoebae and ciliates) to several phyla of the Ecdysozoa (Arthropoda and Priapulida). Unfortunately, the majority of these symbiotic relationships are as yet uncharacterized and broader genomic and phenotypic research is needed to better understand the ecological roles of these symbionts.

*Hepatincola* and ‘*Ca*. Tenuibacter priapulorum’ [18] are the only extracellular *Holosporales* symbionts known to date, and both bacteria belong to the ‘*Ca*. Hepatincolaceae’. From an evolutionary perspective, this sheds light on the emergence of intracellularity within the *Holosporales*, notably in comparison with the more deeply investigated *Rickettsiales*. For the latter, a recently proposed model, motivated by the discovery of the first extracellular *Rickettsiales* bacterium, suggests that intracellularity could have evolved multiple times from an extracellular ancestor [12]. Considering the early-branching position of *Hepatincola* in the phylogeny of the *Holosporales*, the intracellular lifestyle of the protist-associated *Holosporales* families could have evolved from an extracellular bacterium that had previously established a niche in animal digestive systems. Alternatively, the same free-living ancestor (likely in a marine environment) could have given rise to both intracellular symbionts of unicellular host organisms and extracellular symbionts of metazoan hosts. An intriguing aspect in this regard is the repertoire of bacterial secretion systems, which may be involved in the interaction with the host organism. All three *Hepatincola* strains possess a complete Type I secretion system, an additional tolC outer membrane protein and all necessary components for a Type V secretion system as in *Rickettsia*. In addition, *HepAv* has a reduced Type IV secretion system without a pilus. This is unlike any other previously sequenced *Holosporales* symbionts from protists, many of which possess Type IV or tad pili and Type VI secretion systems [14–16]. Hence, the secretion systems of *Hepatincola* are not typical for *Holosporales* but instead similar to the secretome of *Rickettsia* [79], which infect arthropods. Elucidating the role of different secretion systems for the interaction with different host organisms therefore represents an interesting perspective for future research on *Holosporales* symbionts.

The bacteria most closely-related to *Hepatincola* were found in freshwater isopods of the genus *Proasellus*, which belong to a different isopod suborder than terrestrial isopods. This suggests that *Hepatincola* may have evolved from a bacterium associated with the marine ancestor of extent isopod suborders. If proven true, this would contradict an earlier hypothesis proposing that bacterial symbionts present in the midgut glands of terrestrial isopods would have been a prerequisite for the colonization of land, providing the necessary enzymes to digest terrestrial plant food sources rich in lignocellulose [34, 35]. While the terrestrial isopod microbiome definitely contributes tremendously to the degradation of recalcitrant plant material [43, 44], *Hepatincola* possesses only a single gene that might contribute to this process. Similar to other *Holosporales*, *Hepatincola* has a highly streamlined genome with reduced metabolic and biosynthetic capacities. Notably, it cannot produce most amino acids, vitamins and co-factors and has to import essential metabolites and nucleotides from the host. This suggests that *Hepatincola* is rather a nutrient scavenger than a nutrient provider for the host. On the other hand, no virulence factors were observed, apart from two potential relics of a toxin-antitoxin system in *HepPdp*, therefore the symbiont is unlikely to be a true pathogen. The most probable scenario based on the functional repertoire of the sequenced genomes is that *Hepatincola* benefits from a nutrient-rich environment to import all necessary metabolites and precursors, including sugars for glycolysis. However, considering the high abundance that the symbiont can reach in the midgut glands (up to 6.7 × 10^7^ cells/individual in *P. scaber* [31]), its presence could have negative fitness effects on the host, especially during periods of nutrient scarcity. This would be in line with the increased mortality of infected individuals observed in *P. scaber* [32], suggesting a parasitic interaction.

The inclusion of several *Hepatincola* strains from three different host species in this study also provided insights into the genetic diversity of this taxon. The average nucleotide identity (ANI) of only 83% between *HepPp* and the two other strains suggests that *HepPp* might represent a different species from *HepAv* and *HepPdp* and that more divergent strains could be uncovered by obtaining additional genomes from other terrestrial isopod families. The observed relationships further suggest that there is no strict co-divergence between *Hepatincola* and its hosts, since *HepAv* and *HepPdp* are closely-related strains despite being associated with terrestrial isopod species from two distant families. In turn, *HepPp* and *HepPdp* are more distantly related despite being associated with host species from the same host family. However, an absence of host-symbiont co-divergence is not necessarily surprising for a facultative symbiont with often low prevalence in natural populations. Other differences between *HepPp* and *HepAv/HepPdp* consist in the prophage regions, with the largest prophage region being present only in *HepAv* and *HepPdp*. Hence, this region has either been lost from *HepPp* or it was instead acquired more recently by the lineage including *HepAv* and *HepPdp*. The latter seems to be the more realistic scenario based on the available data, as this prophage region is the only one with identifiable phage attachment sites as well as other mobile elements, namely an integrase and phage transposition protein. However, it is unlikely that any of the prophage regions could still produce an active phage, since none contains all components to form an intact phage.

Taken together, this work presents the first genomic insights into an extracellular *Holosporales* symbiont of arthropods. Similar to protist-associated *Holosporales* and *Rickettsiales*, it is likely a nutrient parasite, siphoning nutrients from the gut lumen to fuel its reduced metabolism. Additional genomes from other members of this early-branching *Holosporales* family will be instrumental to shed light on the evolutionary trajectories leading to different symbiotic lifestyles within the *Holosporales*.

## Supplementary information

Supplementary material list

Table S1

Table S2

Table S3

Table S4

Table S5

Table S6

Table S7

Table S8

Table S9

## Data Availability

The three *Hepatincola* genomes produced for this work are accessible in the NCBI database under BioProject accessions PRJNA690767 (*Hepatincola* from *A. vulgare*), PRJNA690772 (*Hepatincola* from *P. dilatatus petiti*) and PRJNA853587 (*Hepatincola* from *P. pruinosus*).
