# Supplementary material for: The terrestrial isopod symbiont ‘Candidatus Hepatincola porcellionum’ is a potential nutrient scavenger related to Holosporales symbionts of protists
Source: ISME Commun. 2023 Mar 8;3:18. doi: 10.1038/s43705-023-00224-w (PMC9992710; doi:10.1038/s43705-023-00224-w)
Supplement: Supplementary file 1 — Supplementary material list [file 43705_2023_224_MOESM1_ESM.docx]

**Supplementary material for:**

**The terrestrial isopod symbiont ‘*Candidatus* Hepatincola porcellionum’ is a potential nutrient scavenger related to Holosporales symbionts of protists**

Jessica Dittmer, Marius Bredon, Bouziane Moumen, Maryline Raimond, Pierre Grève, Didier Bouchon

**Table of contents:**

**Suppl. Table S1. Terrestrial isopod populations tested for *Hepatincola* in this study.**

Two individuals (one female/one male) were tested per population. Positive individuals (F=female, M=male, F/M=both) are provided in the last column.

**Suppl. Table S2. Genomes included in the phylogenomics analysis.** Taxonomy, host organism, intracellular location, accession number, genome size and assembly level are provided.

**Suppl. Table S3. Annotation of the prophage regions in *Hepatincola* genomes.** Contains locus tags and gene annotation for the three prophage regions.

**Suppl. Table S4. 16S rRNA gene sequences included in the phylogenetic analysis.** Accession numbers and sample metadata regarding host species or environmental origin are provided.

**Suppl. Table S5. KEGG pathway annotations for the three genomes.**

**Suppl. Table S6. Carbohydrate active enzymes (CAZymes) identified in the *Hepatincola* genomes.**

**Suppl. Table S7. Predicted transmembrane transporters in the *Hepatincola* genomes.**

**Suppl. Table S8. Annotation of bacterial secretion systems in the *Hepatincola* genomes.**

**Suppl. Table S9. Proteins with predicted signal peptides.**
