# Supplementary material for: The terrestrial isopod symbiont ‘Candidatus Hepatincola porcellionum’ is a potential nutrient scavenger related to Holosporales symbionts of protists
Source: ISME Commun. 2023 Mar 8;3:18. doi: 10.1038/s43705-023-00224-w (PMC9992710; doi:10.1038/s43705-023-00224-w)
Supplement: Supplementary file 2 — Table S1 [file 43705_2023_224_MOESM2_ESM.pdf]

Suppl. Table S1. Terrestrial isopod populations tested for *Hepatincola* in this study.
Two individuals (one female/one male) were tested per population. Positive individuals (F=female, M=male, F/M=both) are provided in the last column.

Family	Genus	Species	Origin	Sampling Site	*Hepatincola* positive (F/M)
**Armadillidae**	*Armadillo*	*officinalis*	Lab	Koymbari, Greece	
**Armadillidiidae**	*Armadillidium*	*assimile*	Lab	Saint Maixent, France	
	Armadillidium	*depressum*	Lab	Saintt-Guilhem-le-Désert, France	
	Armadillidium	*granulatum*	Lab	Kolymbari, Greece	F
	Armadillidium	*maculatum*	Lab	Sainte-Marguerite, France	
	Armadillidium	*nasatum*	Lab	Chizé, France	
	Armadillidium	*siculorum*	Lab	Agrigento, Italy	
	Armadillidium	*tunisiense*	Lab	Khniss, Tunisia	
	Armadillidium	*versicolor*	Lab	Sankt Veit, Austria	M
	Armadillidium	*vulgare*	Lab	Helsingør A, Denmark	
	Armadillidium	*vulgare*	Lab	Helsingør B, Denmark	F
	Eluma	*purpurescens*	Lab	Chizé, France	
**Oniscidae**	*Oniscus*	*asellus*	Lab	Borrisokane, Ireland	M
**Philosciidae**	*Philoscia*	*muscorum*	Field	Ensoulesse, France	M
**Porcellionidae**	*Porcellio*	*dispar*	Lab	Santa Maria del Sol, Spain	
	Porcellio	*dilatatus*	Lab	Rom, France	
	Porcellio	*dilatatus*	Lab	Saint-Honorat, France	F
	Porcellio	*dilatatus*	Lab	Sainte-Marguerite, France	
	Porcellio	*laevis*	Lab	Ichkeul, Tunisia	
	Porcellio	*scaber*	Lab	Vancouver, Canada	
	Porcellio	*scaber*	Lab	Dunedin, New Zealand	
	Porcellionides	*pruinosus*	Field	Ensoulesse, France	M
	Porcellionides	*pruinosus*	Lab	Guadalquivir, Spain	
**Trachelipodidae**	*Orthometopon*	*planum*	Lab	Sainte-Marguerite, France	F/M
**Tylidae**	*Helleria*	*brevicornis*	Lab	Sainte-Marguerite, France	
