# Supplementary material for: The terrestrial isopod symbiont ‘Candidatus Hepatincola porcellionum’ is a potential nutrient scavenger related to Holosporales symbionts of protists
Source: ISME Commun. 2023 Mar 8;3:18. doi: 10.1038/s43705-023-00224-w (PMC9992710; doi:10.1038/s43705-023-00224-w)
Supplement: Supplementary file 3 — Table S2 [file 43705_2023_224_MOESM3_ESM.pdf]

Order	Family	Genus	Strain	Symbiotic association	Location	Accession	Length (plasmid)	Contigs
Rickettsiales	**Anaplasmataceae**	*Anaplasma*	*Anaplasma marginale* Florida	Tick-borne bovine pathogen	Intracellular	GCF_000020305.1	1,202,435	1
			Anaplasma phagocytophilum HZ	Tick-borne human pathogen	Intracellular	GCF_000013125.1	1,471,282	1
		Ehrlichia	*Ehrlichia canis* Jake	Tick-borne pathogen of dogs	Intracellular	GCF_000012565.1	1,315,030	1
			Ehrlichia ruminantium Welgevonden	Tick-borne pathogen of ruminants	Intracellular	GCF_000026005.1	1,516,355	1
		Ca. Neoehrlichia	*Ca.* Neoehrlichia lotoris RAC413	Tick-borne human pathogen	Intracellular	GCF_000964795.1	1,268,660	1
		Neorickettsia	*Neorickettsia helminthoeca* Oregon	Trematode-borne pathogen of dogs	Intracellular	GCF_000632985.1	884,232	1
			Neorickettsia risticii Illinois	Trematode-borne pathogen of horses	Intracellular	GCF_000022525.1	879,977	1
		Wolbachia	*Wolbachia w* AlbB (Supergroup B)	Symbiont of mosquitoes	Intracellular	GCF_004171285.1	1,484,007	1
			Wolbachia w Cle (Supergroup F)	Symbiont of bed bugs	Intracellular	GCF_000829315.1	1,250,060	1
		Ca. Xenolissoclinum	*Ca.* Xenolissoclinum pacificiensis	Symbiont of tunicates (marine invertebrates)	Intracellular	GCA_000512675.1	1,039,565	13
	***Ca.* Deianiraeaceae**	*Ca.* Deianiraea	*Ca.* Deianiraea vastratrix CyL4-1	Pathogen of *Paramecium*	Extracellular	GCF_007993655.1	1,205,153	3
	***Ca.* Midichloriaceae**	*Ca.* Aquarickettsia	*Ca.* Aquarickettsia rohweri	Energy parasite of corals	Unclear, maybe intracellular	GCF_003953955.1	1,284,848	155
		Ca. Fokinia	*Ca.* Fokinia solitaria	Endosymbiont of *Paramecium*	Intracellular	GCF_003072485.1	837,348	1
		Ca. Jidaibacter	*Ca.* Jidaibacter acanthamoeba UWC8	Amoebae symbiont	Intracelluar	GCF_000730245.1	1,615,277	1
		Ca. Midichloria	*Ca.* Midichloria mitochondrii IricVA	Tick symbiont	Intracellular/Intramitochondrial	GCF_000219355.1	1,183,732	1
		Unclassified	Unclassified Rickettsiales	Endosymbiont of protist *Peranema trichophorum*	Intracelluar	GCF_004210275.1	1,375,759	125
	**Rickettsiaceae**	*Ca.* Megaira	*Ca.* Megaira	Endosymbiont of *Mesostigma viride* algae	Intracellular	GCA_020410825.1	1,448,425 (83,984)	1
		Occidentia	*Occidentia massiliensis* Os18	Tick symbiont	Intracellular	GCF_000309075.1	1,469,252	301
		Orientia	*Orientia tsutsugamushi* Boryong	Mite-vectored human pathogen	Intracellular	GCF_000063545.1	2,127,051	1
		Ca. Phycorickettsia	*Ca.* Phycorickettsia trachydisci	Symbiont of green algae	Intracellular	GCF_003015145.1	1,472,411	1
		Rickettsia	*Rickettsia bellii* RML369-C	Nonpathogenic tick symbiont	Intracellular	GCF_000012385.1	1,522,076	1
			Rickettsia RiClec	Endosymbiont of bed bugs	Intracellular	GCA_020410805.1	1,611,726	1
			Rickettsia rickettsii Iowa	Tick-borne human pathogen	Intracellular	GCF_001951015.1	1,268,242	1
			Rickettsia typhi Wilmington	Flea-borne human pathogen	Intracellular	GCF_000008045.1	1,111,496	1
		Ca. Sneabacter	*Ca.* Sneabacter namystus 1621	Endosymbiont of protists	Intracellular	GCF_008189685.1	605,411 (27,732)	1
Holosporales	***Ca.* Bodocaedibacteraceae**	*Ca.* Bodocaedibacter	*Ca.* Bodocaedibacter vickermanii	Symbiont of protists	Intracellular	GCA_014896945.1	1,391,311	1
	**Caedimonadaceae**	*Caedimonas*	*Caedimonas varicaedens*	Symbiont of ciliates	Intracellular	GCF_001192655.1	1,686,852	142
		Ca. Nucleicultrix	*Ca.* Nucleicultrix amoebiphila FS5	Symbiont of amoebae	Intranuclear	GCF_002117145.1	1,838,212	1
		Ca. Paracaedimonas	*Ca.* Paracaedimonas acanthamoebae	Symbiont of amoebae	Intracellular	GCA_000743035.1	1,722,347 (453,426)	1
	**Holosporaceae**	*Ca.* Cytomitobacter	*Ca.* Cytomitobacter indipagum 1605	Symbiont of protists	Intracellular	GCF_008189285.1	628,024	1
			Ca. Cytomitobacter primus 1604LC	Symbiont of protists	Intracellular	GCF_008189405.1	622,357	1
		Ca. Glomoviella	*Ca.* Gromoviella agglomerans Sp8-1	Pathogen of *Paramecium*	Intracellular	GCF_021065005.1	589,967	1
		Holospora	*Holospora curviuscula* NRB217	Symbiont of ciliates	Intranuclear	GCF_002930195.1	1,715,500	210
			Holospora elegans E1	Symbiont of ciliates	Intranuclear	GCF_000648275.1	1,268,333	152
			Holospora obtusa F1	Symbiont of ciliates	Intranuclear	GCF_000469665.2	1,334,837	91
			Holospora undulata HU1	Symbiont of ciliates	Intranuclear	GCF_000388175.3	1,402,636	208
		Ca. Hydrogenosomobacter	*Ca.* Hydrogenosomobacter endosymbioticus 200920	Symbiont of ciliates	Intracellular	GCF_021654655.1	826,669	1
		Ca. Nesciobacter	*Ca.* Nesciobacter abundans 1604HC	Symbiont of protists	Intracellular	GCF_008189525.1	616,088	1
	***Ca.* Paracaedibacteraceae**	*Ca* . Finniella	*Ca.* Finniella inopinata HOLO01	Symbiont of protists	Intracellular	GCF_004210305.1	1,792,168	28
		Ca. Odyssella	*Ca.* Odyssella thessalonicensis L13	Symbiont of amoebae	Intracellular	GCF_000190415.1	2,847,648	20
		Ca. Paracaedibacter	*Ca.* Paracaedibacter acanthamoebae PRA3	Symbiont of amoebae	Intracellular	GCF_000742835.1	2,470,036 (15,074)	1
