# Supplementary material for: The terrestrial isopod symbiont ‘Candidatus Hepatincola porcellionum’ is a potential nutrient scavenger related to Holosporales symbionts of protists
Source: ISME Commun. 2023 Mar 8;3:18. doi: 10.1038/s43705-023-00224-w (PMC9992710; doi:10.1038/s43705-023-00224-w)
Supplement: Supplementary file 4 — Table S3 [file 43705_2023_224_MOESM4_ESM.pdf]

Phage Region 1 - HepAv, HepPdp

CDS HepAv	CDS HepPdp	CDS HepPP	Annotation
HAV_00296	HPDP_00194		Helix-turn-helix transcriptional regulator
HAV_00297	HPDP_00195		Transcriptional regulator
HAV_00298	HPDP_00196		Integrase/transposase/recombinase
HAV_00299	HPDP_00197		Phage transposition protein
HAV_00300	HPDP_00198		Putative phage protein
HAV_00301	HPDP_00199		Putative phage protein
HAV_00302			Putative phage protein
HAV_00303	HPDP_00200		Putative phage protein
HAV_00304	HPDP_00201		Putative phage protein
HAV_00305	HPDP_00202		Putative phage protein
HAV_00306	HPDP_00203		Helix-turn-helix domain phage protein
HAV_00307	HPDP_00204		Phage lysozyme protein
HAV_00308	HPDP_00205		Putative phage protein
HAV_00309	HPDP_00206		Putative phage protein
HAV_00310			Phage virion morphogenesis protein
HAV_00311	HPDP_00207		Putative phage protein
HAV_00312	HPDP_00208		Phage baseplate assembly protein V
HAV_00313		HPPR_00282	Putative phage protein
HAV_00314			Putative phage protein
HAV_00315			Putative phage protein
HAV_00316			Baseplate J family protein
HAV_00317			Phage tail protein I
HAV_00318	HPDP_00209	HPPR_00523, HPPR_00524	Phage tail-collar fiber protein 2
HAV_00319	HPDP_00210		Phage tail fiber protein
HAV_00320	HPDP_00211		Phage tail fiber protein
HAV_00321	HPDP_00212		Putative prophage major tail sheath protein
HAV_00322	HPDP_00213		Phage major tail tube protein
HAV_00323	HPDP_00214		Phage tail assembly protein
HAV_00324	HPDP_00215		Phage lysozyme
HAV_00325	HPDP_00216		Phage baseplate assembly protein
HAV_00326	HPDP_00217		Putative phage protein
HAV_00327	HPDP_00218		Phage tail protein X
HAV_00328	HPDP_00219		Phage late control D protein

Phage Region 2 - HepAv, HepPdp, HepPp

CDS HepAv	CDS HepPdp	CDS HepPp	Annotation
HAV_00520	HPDP_00415	HPPR_00458	Putative phage protein
HAV_00521	HPDP_00416	HPPR_00459	Putative phage protein
HAV_00522	HPDP_00417	HPPR_00460	Putative phage protein
HAV_00523	HPDP_00418	HPPR_00461	Phage tail tubular protein A
HAV_00524	HPDP_00419	HPPR_00462	Putative phage protein
HAV_00525	HPDP_00420	HPPR_00463	Putative major capsid protein
HAV_00526	HPDP_00421	HPPR_00464	Putative phage protein
HAV_00527	HPDP_00422	HPPR_00465	Putative phage protein
HAV_00528	HPDP_00423	HPPR_00466	Head-tail connector phage protein
HAV_00529	HPDP_00424	HPPR_00467	Phage large terminase protein

Phage Region 3 - HepAv, HepPdp, HepPp

CDS HepAv	CDS HepPdp	CDS HepPp	Annotation
HAV_01073	HPDP_00981	HPPR_01019	Phage late control D protein
HAV_01074	HPDP_00982	HPPR_01020	Phage tail protein X
HAV_01075	HPDP_00983	HPPR_01021	Putative phage protein
HAV_01076	HPDP_00984	HPPR_01022	Phage lysozyme protein
HAV_01077	HPDP_00985	HPPR_01023	Putative phage protein
HAV_01078	HPDP_00986	HPPR_01024	Phage tail assembly protein
HAV_01079	HPDP_00987	HPPR_01025	Phage major tail tube protein
HAV_01080	HPDP_00988	HPPR_01026	Putative prophage major tail sheath protein
HAV_01081	HPDP_00989	HPPR_01027	Phage tail-collar fiber protein
HAV_01082	HPDP_00990	HPPR_01028	Phage tail protein I
HAV_01083	HPDP_00991	HPPR_01029	Baseplate assembly protein
HAV_01084	HPDP_00992	HPPR_01030	Putative phage protein
HAV_01085	HPDP_00993	HPPR_01031	Phage baseplate assembly protein V
HAV_01086	HPDP_00994		Putative phage protein
