# Supplementary material for: The terrestrial isopod symbiont ‘Candidatus Hepatincola porcellionum’ is a potential nutrient scavenger related to Holosporales symbionts of protists
Source: ISME Commun. 2023 Mar 8;3:18. doi: 10.1038/s43705-023-00224-w (PMC9992710; doi:10.1038/s43705-023-00224-w)
Supplement: Supplementary file 5 — Table S4 [file 43705_2023_224_MOESM5_ESM.pdf]

Accession Number	Sample type	Host species	Common name	Environmental source	Origin
This study	Host-associated	*Armadillidium vulgare*	Terrestrial isopod		France
This study	Host-associated	*Porcellio dilatatus petiti*	Terrestrial isopod		France
This study	Host-associated	*Porcellionides pruinosus*	Terrestrial isopod		France
AY188585	Host-associated	*Porcellio scaber*	Terrestrial isopod		Germany
AY189806	Host-associated	*Porcellio scaber*	Terrestrial isopod		Germany
MG275568	Environmental sample			Leaf, root, soil from *Senecio vulgaris*	China
KX975954	Environmental sample			Tropical urban freshwater	Singapore
KX975534	Environmental sample			Tropical urban freshwater	Singapore
HAFB01032592	Host-associated	*Proasellus jaloniacus*	Freshwater isopod		Unknown
AB518658	Environmental sample			Sediment in a water well	Japan
AB518600	Environmental sample			Sediment in a water well	Japan
HAFC01084331	Host-associated	*Proasellus karamani*	Freshwater isopod		Unknown
HAFC01084346	Host-associated	*Proasellus karamani*	Freshwater isopod		Unknown
HACK01026685	Host-associated	*Cherax quadricarinatus*	Decapod		Australia
HACB02063745	Host-associated	*Cherax quadricarinatus*	Decapod		Australia
AB622505	Host-associated	*Brachionus plicatilis*	Rotifer		Japan
AB622488	Host-associated	*Brachionus plicatilis*	Rotifer		Japan
KX974162	Environmental sample			Tropical urban freshwater	Singapore
KX973027	Environmental sample			Tropical urban freshwater	Singapore
KP138714	Host-associated	*Priapulus caudatus*	Marine worm		Sweden
KP138715	Host-associated	*Priapulus caudatus*	Marine worm		Sweden
KP138716	Host-associated	*Priapulus caudatus*	Marine worm		Sweden
KU195266	Host-associated	*Priapulus caudatus*	Marine worm		Sweden
EU675718	Host-associated	*Bosmina coregoni*	Branchiopod		Germany
KF008909	Environmental sample			Poyang Lake water	China
KY516505	Environmental sample			Lake water	Canada
KU639271	Environmental sample			Seaweed (*Asparagopsis*)	Portugal
KU620353	Environmental sample			Seaweed (*Asparagopsis*)	Portugal
KU615846	Environmental sample			Seaweed (*Asparagopsis*)	Portugal
KF177217	Environmental sample			*Zea mays*	China
AYNC02020569	Host-associated	*Ephemera danica*	Insect, mayfly		Germany
KF185323	Environmental sample			Coastal seawater	Italy
MH112578	Environmental sample			Lake water	Switzerland
JN392909	Host-associated	*Daphnia magma*	Branchiopod		China
KF509624	Environmental sample			Mouse skin	USA
KX971354	Environmental sample			Tropical urban freshwater	Singapore
GAWP02042495	Host-associated	*Grylloblatta bifratrilecta*	Insect, rock crawler		USA
GQ853368	Host-associated	*Dactylopius opuntiae*	Insect, scale insect		Mexico
KX363697	Host-associated	*Phlebotomus chinensis*	Insect, sand fly		China
KX363698	Host-associated	*Phlebotomus chinensis*	Insect, sand fly		China
JQ389880	Host-associated	*Euglossa imperialis*	Insect, orchid bee		Panama
JQ389881	Host-associated	*Euglossa imperialis*	Insect, orchid bee		Panama
KX970430	Environmental sample			Tropical urban freshwater	Singapore
KU633890	Environmental sample			Seaweed (*Asparagopsis*)	Portugal
KU690334	Environmental sample			Epiphytic on seaweed *Cystoseira compressa*	Italy
