# Supplementary material for: The terrestrial isopod symbiont ‘Candidatus Hepatincola porcellionum’ is a potential nutrient scavenger related to Holosporales symbionts of protists
Source: ISME Commun. 2023 Mar 8;3:18. doi: 10.1038/s43705-023-00224-w (PMC9992710; doi:10.1038/s43705-023-00224-w)
Supplement: Supplementary file 6 — Table S5 [file 43705_2023_224_MOESM6_ESM.pdf]

Query	KO	Definition	Score	Second best	Score
HAV_00001					
HAV_00002					
HAV_00003					
HAV_00004			2	K23743	1
HAV_00005			3	K23743	1
HAV_00006			251		
HAV_00007	K01652	E2.2.1.6L, ilvB, ilvG, ilvI; acetolactate synthase I/II/III large subunit [EC:2.2.1.6]	317		
HAV_00008	K01647	CS, gltA; citrate synthase [EC:2.3.3.1]	217		
HAV_00009	K00031	IDH1, IDH2, icd; isocitrate dehydrogenase [EC:1.1.1.42]	248		
HAV_00010	K01681	ACO, acnA; aconitate hydratase [EC:4.2.1.3]	757		
HAV_00011	K00024	mdh; malate dehydrogenase [EC:1.1.1.37]	196		
HAV_00012	K13821	putA; RHH-type transcriptional regulator, proline utilization regulon repressor / pro	392		
HAV_00013	K11928	putP; sodium/proline symporter	167		
HAV_00014	K03325	ACR3, arsB; arsenite transporter	190		
HAV_00015	K14155	patB, malY; cysteine-S-conjugate beta-lyase [EC:4.4.1.13]	139		
HAV_00016	K01489	cdd, CDA; cytidine deaminase [EC:3.5.4.5]	79		
HAV_00017	K00758	deoA, TYMP; thymidine phosphorylase [EC:2.4.2.4]	214		
HAV_00018	K01619	deoC, DERA; deoxyribose-phosphate aldolase [EC:4.1.2.4]	98		
HAV_00019	K03317	TC.CNT; concentrative nucleoside transporter, CNT family	213		
HAV_00022					
HAV_00023			2	K06968	1
HAV_00024			1	K06936	1
HAV_00025					
HAV_00026	K06207	typA, bipA; GTP-binding protein	373		
HAV_00027			33	K15270	3
HAV_00028			34	K15270	5
HAV_00029			40	K15270	2
HAV_00032			36	K19427	2
HAV_00033	K00831	serC, PSAT1; phosphoserine aminotransferase [EC:2.6.1.52]	151		
HAV_00035			35	K09809	1
HAV_00036	K03070	secA; preprotein translocase subunit SecA [EC:7.4.2.8]	444		
HAV_00037	K03558	cvpA; membrane protein required for colicin V production	15		
HAV_00038			18		
HAV_00039	K17713	bamB; outer membrane protein assembly factor BamB	33		
HAV_00040	K03977	engA, der; GTPase	168		
HAV_00041	K04485	radA, sms; DNA repair protein RadA/Sms	226		
HAV_00042					
HAV_00043	K17103	CHO1, pssA; CDP-diacylglycerol---serine O-phosphatidyltransferase [EC:2.7.8.8]	61		
HAV_00044	K01613	psd, PISD; phosphatidylserine decarboxylase [EC:4.1.1.65]	150		
HAV_00045	K01878	glyQ; glycyl-tRNA synthetase alpha chain [EC:6.1.1.14]	183		
HAV_00046	K01879	glyS; glycyl-tRNA synthetase beta chain [EC:6.1.1.14]	164		
HAV_00047			1	K07787	1
HAV_00048	K02779	ptsG; glucose PTS system EIICB or EIICBA component [EC:2.7.1.199]	198		
HAV_00050			2	K01881	1
HAV_00051			1	K03215	1
HAV_00052	K06980	ygfZ; tRNA-modifying protein YgfZ	55	K22073	10
HAV_00053	K01919	gshA; glutamate--cysteine ligase [EC:6.3.2.2]	159		
HAV_00054	K09761	rsmE; 16S rRNA (uracil1498-N3)-methyltransferase [EC:2.1.1.193]	55		
HAV_00055	K03179	ubiA; 4-hydroxybenzoate polyprenyltransferase [EC:2.5.1.39]	105		
HAV_00056	K03182	ubiD; 4-hydroxy-3-polyprenylbenzoate decarboxylase [EC:4.1.1.98]	296		
HAV_00057	K04566	lysK; lysyl-tRNA synthetase, class I [EC:6.1.1.6]	285		
HAV_00058	K13583	gcrA; GcrA cell cycle regulator	19		
HAV_00059			63		
HAV_00060	K02004	ABC.CD.P; putative ABC transport system permease protein	20		
HAV_00061			16	K09808	15
HAV_00062	K02003	ABC.CD.A; putative ABC transport system ATP-binding protein	102		
HAV_00063			18	K01711	1
HAV_00064			12	K01183	2
HAV_00065			3	K00425	1
HAV_00066	K09952	csn1, cas9; CRISPR-associated endonuclease Csn1 [EC:3.1.-.-]	19		
HAV_00067	K15342	cas1; CRISP-associated protein Cas1	66		
HAV_00068	K09951	cas2; CRISPR-associated protein Cas2	52		
HAV_00069			26	K10986	1
HAV_00070	K02986	RP-S4, rpsD; small subunit ribosomal protein S4	146		
HAV_00071	K21469	pbp4b; serine-type D-Ala-D-Ala carboxypeptidase [EC:3.4.16.4]	135		
HAV_00072					
HAV_00073			1	K01576	1

HAV_00074			2	K12340	1
HAV_00075			3	K01838	1
HAV_00076					
HAV_00077	K06999	K06999; phospholipase/carboxylesterase	46		
HAV_00078			2	K02557	1
HAV_00079	K07390	grxD, GLRX5; monothiol glutaredoxin	19		
HAV_00080	K03630	radC; DNA repair protein RadC	93		
HAV_00081	K01265	map; methionyl aminopeptidase [EC:3.4.11.18]	170		
HAV_00082	K06206	sfsA; sugar fermentation stimulation protein A	91		
HAV_00083			36	K18162	13
HAV_00084			61		
HAV_00085	K03676	grxC, GLRX, GLRX2; glutaredoxin 3	42	K00384	1
HAV_00086	K00568	ubiG; 2-polyprenyl-6-hydroxyphenyl methylase / 3-demethylubiquinone-9 3-methy	84		
HAV_00087	K02371	fabK; enoyl-[acyl-carrier protein] reductase II [EC:1.3.1.9]	192		
HAV_00088					
HAV_00089	K03526	gcpE, ispG; (E)-4-hydroxy-3-methylbut-2-enyl-diphosphate synthase [EC:1.17.7.1 1.	220		
HAV_00090	K01892	HARS, hisS; histidyl-tRNA synthetase [EC:6.1.1.21]	172		
HAV_00091	K02835	prfA, MTRF1, MRF1; peptide chain release factor 1	214		
HAV_00092	K02493	hemK, prmC, HEMK; release factor glutamine methyltransferase [EC:2.1.1.297]	80		
HAV_00093	K02871	RP-L13, MRPL13, rplM; large subunit ribosomal protein L13	130		
HAV_00094	K02996	RP-S9, MRPS9, rpsI; small subunit ribosomal protein S9	129		
HAV_00095			2	K00342	1
HAV_00096	K09816	znuB; zinc transport system permease protein	73		
HAV_00097	K09817	znuC; zinc transport system ATP-binding protein [EC:7.2.2.20]	74		
HAV_00098	K01999	livK; branched-chain amino acid transport system substrate-binding protein	39		
HAV_00099			64		
HAV_00100	K00566	mnmA, trmU; tRNA-uridine 2-sulfurtransferase [EC:2.8.1.13]	125		
HAV_00101			77		
HAV_00102	K01875	SARS, serS; seryl-tRNA synthetase [EC:6.1.1.11]	193		
HAV_00103			30	K06194	1
HAV_00104	K03210	yajC; preprotein translocase subunit YajC	33		
HAV_00105	K03072	secD; preprotein translocase subunit SecD	96	K12257	47
HAV_00106	K03074	secF; preprotein translocase subunit SecF	78	K12257	7
HAV_00107			12	K09008	1
HAV_00108	K08305	mltB; membrane-bound lytic murein transglycosylase B [EC:4.2.2.-]	25		
HAV_00109	K03427	hsdM; type I restriction enzyme M protein [EC:2.1.1.72]	54		
HAV_00110	K06867	K06867; uncharacterized protein	10		
HAV_00111					
HAV_00112	K09815	znuA; zinc transport system substrate-binding protein	53		
HAV_00113	K03089	rpoH; RNA polymerase sigma-32 factor	114		
HAV_00116			7	K01271	1
HAV_00117	K03923	mdaB; NADPH dehydrogenase (quinone) [EC:1.6.5.10]	159		
HAV_00118					
HAV_00119			2	K01686	1
HAV_00120			119		
HAV_00121	K17686	copA, ctpA, ATP7; P-type Cu+ transporter [EC:7.2.2.8]	298		
HAV_00123	K00022	HADH; 3-hydroxyacyl-CoA dehydrogenase [EC:1.1.1.35]	98		
HAV_00124	K02440	GLPF; glycerol uptake facilitator protein	132		
HAV_00125	K00864	glpK, GK; glycerol kinase [EC:2.7.1.30]	320		
HAV_00126	K00111	glpA, glpD; glycerol-3-phosphate dehydrogenase [EC:1.1.5.3]	217		
HAV_00127	K03564	BCP, PRXQ, DOT5; thioredoxin-dependent peroxiredoxin [EC:1.11.1.24]	65		
HAV_00128	K03564	BCP, PRXQ, DOT5; thioredoxin-dependent peroxiredoxin [EC:1.11.1.24]	54		
HAV_00129					
HAV_00130			1	K07776	1
HAV_00131	K06895	lysE, argO; L-lysine exporter family protein LysE/ArgO	145		
HAV_00132	K07266	kpsC, lipA; capsular polysaccharide export protein	11		
HAV_00133	K07266	kpsC, lipA; capsular polysaccharide export protein	30		
HAV_00134	K08344	scsB; suppressor for copper-sensitivity B	124		
HAV_00135			100		
HAV_00136	K22044	ybiO; moderate conductance mechanosensitive channel	41		
HAV_00137	K00759	APRT, apt; adenine phosphoribosyltransferase [EC:2.4.2.7]	104		
HAV_00138			1		
HAV_00139	K02523	ispB; octaprenyl-diphosphate synthase [EC:2.5.1.90]	175		
HAV_00140			58	K15460	7
HAV_00141			110		
HAV_00142	K02902	RP-L28, MRPL28, rpmB; large subunit ribosomal protein L28	66		
HAV_00143					
HAV_00144	K03699	tlyC; magnesium and cobalt exporter, CNNM family	137		

HAV_00145	K01735	aroB; 3-dehydroquinate synthase [EC:4.2.3.4]	92		
HAV_00146	K00891	aroK, aroL; shikimate kinase [EC:2.7.1.71]	48		
HAV_00147					
HAV_00148	K04763	xerD; integrase/recombinase XerD	116		
HAV_00149	K01962	accA; acetyl-CoA carboxylase carboxyl transferase subunit alpha [EC:6.4.1.2 2.1.3.1	173		
HAV_00151			2	K12069	1
HAV_00152			2		
HAV_00153			1	K16562	1
HAV_00154	K03199	virB4, lvhB4; type IV secretion system protein VirB4 [EC:7.4.2.8]	34	K12053	8
HAV_00155			6	K06079	1
HAV_00156			5	K18434	1
HAV_00157					
HAV_00158					
HAV_00159			13	K09799	
HAV_00160			1	K04565	1
HAV_00161			2	K20266	1
HAV_00162	K03201	virB6, lvhB6; type IV secretion system protein VirB6	11		
HAV_00163	K03203	virB8, lvhB8; type IV secretion system protein VirB8	10	K12050	1
HAV_00164					
HAV_00165	K03195	virB10, lvhB10; type IV secretion system protein VirB10	11	K20533	3
HAV_00166			3	K03816	1
HAV_00167	K03205	virD4, lvhD4; type IV secretion system protein VirD4 [EC:7.4.2.8]	56		
HAV_00168	K14623	dinD; DNA-damage-inducible protein D	129		
HAV_00169			8	K01153	1
HAV_00170			2	K02171	1
HAV_00171	K03169	topB; DNA topoisomerase III [EC:5.6.2.1]	229		
HAV_00172			2	K06174	1
HAV_00173			4	K14652	1
HAV_00174			7	K23779	1
HAV_00175			5	K07733	1
HAV_00176			38		
HAV_00177			2	K09900	1
HAV_00178			2	K00406	1
HAV_00179					
HAV_00180	K02257	COX10, ctaB, cyoE; heme o synthase [EC:2.5.1.141]	101		
HAV_00181	K02297	cyoA; cytochrome o ubiquinol oxidase subunit II [EC:7.1.1.3]	122		
HAV_00182	K02298	cyoB; cytochrome o ubiquinol oxidase subunit I [EC:7.1.1.3]	485		
HAV_00183	K02299	cyoC; cytochrome o ubiquinol oxidase subunit III	119		
HAV_00184	K02300	cyoD; cytochrome o ubiquinol oxidase subunit IV	35		
HAV_00185	K03305	TC.POT; proton-dependent oligopeptide transporter, POT family	160		
HAV_00186					
HAV_00187	K19303	mepH; murein DD-endopeptidase [EC:3.4.-.-]	20		
HAV_00188			35	K21948	1
HAV_00189	K00571	E2.1.1.72; site-specific DNA-methyltransferase (adenine-specific) [EC:2.1.1.72]	47	K13581	30
HAV_00190			58		
HAV_00191			7		
HAV_00192			1	K08363	1
HAV_00193	K03545	tig; trigger factor	125		
HAV_00194	K01358	clpP, CLPP; ATP-dependent Clp protease, protease subunit [EC:3.4.21.92]	181		
HAV_00195	K03544	clpX, CLPX; ATP-dependent Clp protease ATP-binding subunit ClpX	201		
HAV_00196	K01338	lon; ATP-dependent Lon protease [EC:3.4.21.53]	362		
HAV_00197	K03530	hupB; DNA-binding protein HU-beta	41		
HAV_00201	K01752	E4.3.1.17, sdaA, sdaB, tdcG; L-serine dehydratase [EC:4.3.1.17]	206		
HAV_00202	K07240	chrA; chromate transporter	38		
HAV_00203	K07240	chrA; chromate transporter	30		
HAV_00204	K01207	nagZ; beta-N-acetylhexosaminidase [EC:3.2.1.52]	126		
HAV_00205			70		
HAV_00206	K05304	NANS, SAS; sialic acid synthase [EC:2.5.1.56 2.5.1.57 2.5.1.132]	166	K01654	32
HAV_00207			9	K02030	1
HAV_00208			1	K00906	1
HAV_00209	K03778	ldhA; D-lactate dehydrogenase [EC:1.1.1.28]	170		
HAV_00210	K01915	glnA, GLUL; glutamine synthetase [EC:6.3.1.2]	17		
HAV_00211	K01961	accC; acetyl-CoA carboxylase, biotin carboxylase subunit [EC:6.4.1.2 6.3.4.14]	280		
HAV_00212	K02160	accB, bccP; acetyl-CoA carboxylase biotin carboxyl carrier protein	41		
HAV_00213	K03786	aroQ, qutE; 3-dehydroquinate dehydratase II [EC:4.2.1.10]	65		
HAV_00214	K01581	E4.1.1.17, ODC1, speC, speF; ornithine decarboxylase [EC:4.1.1.17]	190		
HAV_00215			37		
HAV_00216	K03308	TC.NSS; neurotransmitter:Na+ symporter, NSS family	181		

HAV_00217	K03308	TC.NSS; neurotransmitter:Na+ symporter, NSS family	134		
HAV_00218			58		
HAV_00219			5	K03286	3
HAV_00220	K07462	recJ; single-stranded-DNA-specific exonuclease [EC:3.1.-.-]	171		
HAV_00222					
HAV_00223					
HAV_00224			5	K07646	1
HAV_00225	K00820	glmS, GFPT; glutamine---fructose-6-phosphate transaminase (isomerizing) [EC:2.6.1	294		
HAV_00226			21	K11746	20
HAV_00227			3	K12509	1
HAV_00228	K06199	crcB, FEX; fluoride exporter	35		
HAV_00229			104		
HAV_00230					
HAV_00231	K03595	era, ERAL1; GTPase	108		
HAV_00232	K03584	recO; DNA repair protein RecO (recombination protein O)	70		
HAV_00233	K00573	E2.1.1.77, pcm; protein-L-isoaspartate(D-aspartate) O-methyltransferase [EC:2.1.1.	37		
HAV_00234			3	K09991	1
HAV_00235	K01873	VARS, valS; valyl-tRNA synthetase [EC:6.1.1.9]	412		
HAV_00236	K00330	nuoA; NADH-quinone oxidoreductase subunit A [EC:7.1.1.2]	70		
HAV_00237	K00331	nuoB; NADH-quinone oxidoreductase subunit B [EC:7.1.1.2]	163		
HAV_00238	K00332	nuoC; NADH-quinone oxidoreductase subunit C [EC:7.1.1.2]	86		
HAV_00239	K00333	nuoD; NADH-quinone oxidoreductase subunit D [EC:7.1.1.2]	308		
HAV_00240	K00334	nuoE; NADH-quinone oxidoreductase subunit E [EC:7.1.1.2]	45	K03943	8
HAV_00241	K00335	nuoF; NADH-quinone oxidoreductase subunit F [EC:7.1.1.2]	243		
HAV_00242	K00336	nuoG; NADH-quinone oxidoreductase subunit G [EC:7.1.1.2]	298		
HAV_00243	K00337	nuoH; NADH-quinone oxidoreductase subunit H [EC:7.1.1.2]	217		
HAV_00244	K00338	nuoI; NADH-quinone oxidoreductase subunit I [EC:7.1.1.2]	98		
HAV_00245	K00339	nuoJ; NADH-quinone oxidoreductase subunit J [EC:7.1.1.2]	76		
HAV_00246	K00340	nuoK; NADH-quinone oxidoreductase subunit K [EC:7.1.1.2]	52		
HAV_00247	K00341	nuoL; NADH-quinone oxidoreductase subunit L [EC:7.1.1.2]	226		
HAV_00248	K00342	nuoM; NADH-quinone oxidoreductase subunit M [EC:7.1.1.2]	164		
HAV_00249	K00343	nuoN; NADH-quinone oxidoreductase subunit N [EC:7.1.1.2]	141		
HAV_00250	K03524	birA; BirA family transcriptional regulator, biotin operon repressor / biotin---[acety	45		
HAV_00251	K03525	coaX; type III pantothenate kinase [EC:2.7.1.33]	33		
HAV_00252	K12574	rnj; ribonuclease J [EC:3.1.-.-]	156		
HAV_00253			23	K03279	1
HAV_00254			23	K03276	1
HAV_00255	K25227	sdsA1; linear primary-alkylsulfatase [EC:3.1.6.21]	309		
HAV_00256	K06901	pbuG, azgA, ghxP, ghxQ, adeQ; adenine/guanine/hypoxanthine permease	158		
HAV_00257	K00459	ncd2, npd; nitronate monooxygenase [EC:1.13.12.16]	88		
HAV_00258	K01881	PARS, proS; prolyl-tRNA synthetase [EC:6.1.1.15]	247		
HAV_00259	K09808	lolC_E; lipoprotein-releasing system permease protein	169		
HAV_00260	K09810	lolD; lipoprotein-releasing system ATP-binding protein [EC:7.6.2.-]	98		
HAV_00261	K02337	dnaE; DNA polymerase III subunit alpha [EC:2.7.7.7]	387		
HAV_00262			1	K22081	1
HAV_00263	K06949	rsgA, engC; ribosome biogenesis GTPase / thiamine phosphate phosphatase [EC:3.(	103		
HAV_00264					
HAV_00265			18	K07006	1
HAV_00266					
HAV_00267	K04564	SOD2; superoxide dismutase, Fe-Mn family [EC:1.15.1.1]	113		
HAV_00268			2	K16092	1
HAV_00269			3		
HAV_00270					
HAV_00271	K03642	rlpA; rare lipoprotein A	76		
HAV_00272	K07258	dacC, dacA, dacD; serine-type D-Ala-D-Ala carboxypeptidase (penicillin-binding pro	104		
HAV_00273	K00943	tmk, DTYMK; dTMP kinase [EC:2.7.4.9]	32		
HAV_00274	K02341	holB; DNA polymerase III subunit delta' [EC:2.7.7.7]	43		
HAV_00275			11	K14654	4
HAV_00276	K00374	narI, narV; nitrate reductase gamma subunit [EC:1.7.5.1 1.7.99.-]	14	K01011	1
HAV_00277					
HAV_00278	K01286	E3.4.16.4; D-alanyl-D-alanine carboxypeptidase [EC:3.4.16.4]	69	K07258	20
HAV_00279	K00525	E1.17.4.1A, nrdA, nrdE; ribonucleoside-diphosphate reductase alpha chain [EC:1.17	652		
HAV_00280			35		
HAV_00281	K02067	mlaD, linM; phospholipid/cholesterol/gamma-HCH transport system substrate-bin	15		
HAV_00282	K02067	mlaD, linM; phospholipid/cholesterol/gamma-HCH transport system substrate-bin	51		
HAV_00283			18		
HAV_00284			91		
HAV_00285	K03701	uvrA; excinuclease ABC subunit A	590		

HAV_00286			21	K04763	1
HAV_00287	K03111	ssb; single-strand DNA-binding protein	83		
HAV_00288	K03270	kdsC; 3-deoxy-D-manno-octulosonate 8-phosphate phosphatase (KDO 8-P phospha	54		
HAV_00289	K03811	pnuC; nicotinamide mononucleotide transporter	25		
HAV_00290	K03811	pnuC; nicotinamide mononucleotide transporter	28		
HAV_00291	K02967	RP-S2, MRPS2, rpsB; small subunit ribosomal protein S2	131		
HAV_00292	K02357	tsf, TSFM; elongation factor Ts	147		
HAV_00293	K09903	pyrH; uridylate kinase [EC:2.7.4.22]	127		
HAV_00294	K02838	frr, MRRF, RRF; ribosome recycling factor	86		
HAV_00295	K00806	uppS; undecaprenyl diphosphate synthase [EC:2.5.1.31]	93		
HAV_00296			7	K01356	1
HAV_00297	K07724	ner, nlp, sfsB; Ner family transcriptional regulator	11		
HAV_00298	K07497	K07497; putative transposase	83		
HAV_00299			81		
HAV_00300			3	K10254	1
HAV_00301					
HAV_00302			1	K07156	1
HAV_00303					
HAV_00304			1	K07497	1
HAV_00305					
HAV_00306			7	K03790	1
HAV_00307			52		
HAV_00308			4	K04100	1
HAV_00309			3	K13525	1
HAV_00310			7	K01739	1
HAV_00311			7	K24117	1
HAV_00312			10	K08151	1
HAV_00313			2		
HAV_00314			3	K02656	1
HAV_00315					
HAV_00316			122		
HAV_00317			36		
HAV_00318			10	K12472	1
HAV_00319			10	K01816	1
HAV_00320			8	K06920	1
HAV_00321	K06907	K06907; uncharacterized protein	138		
HAV_00322	K06908	K06908; uncharacterized protein	58		
HAV_00323			12	K04568	1
HAV_00324			2	K04517	1
HAV_00325	K06903	K06903; uncharacterized protein	15		
HAV_00326			2	K01803	1
HAV_00327			5	K20074	1
HAV_00328	K06905	K06905; uncharacterized protein	20		
HAV_00329					
HAV_00330			8	K22847	1
HAV_00331			3	K15372	1
HAV_00332	K03664	smpB; SsrA-binding protein	58		
HAV_00333	K00981	E2.7.7.41, CDS1, CDS2, cdsA; phosphatidate cytidylyltransferase [EC:2.7.7.41]	30		
HAV_00334	K17837	bla2, blm, ccrA, blaB; metallo-beta-lactamase class B [EC:3.5.2.6]	55	K18782	11
HAV_00335			220		
HAV_00336	K03303	lctP; lactate permease	197		
HAV_00337	K01095	pgpA; phosphatidylglycerophosphatase A [EC:3.1.3.27]	15		
HAV_00338			2	K19075	1
HAV_00339	K03060	rpoZ; DNA-directed RNA polymerase subunit omega [EC:2.7.7.6]	21		
HAV_00340	K01139	spoT; GTP diphosphokinase / guanosine-3',5'-bis(diphosphate) 3'-diphosphatase [E	205		
HAV_00341	K03474	pdxJ; pyridoxine 5-phosphate synthase [EC:2.6.99.2]	116		
HAV_00342	K00997	acpS; holo-[acyl-carrier protein] synthase [EC:2.7.8.7]	39	K17758	1
HAV_00343	K03100	lepB; signal peptidase I [EC:3.4.21.89]	85		
HAV_00344	K03685	rnc, DROSHA, RNT1; ribonuclease III [EC:3.1.26.3]	61	K03595	2
HAV_00345			19	K21687	1
HAV_00346	K01624	FBA, fbaA; fructose-bisphosphate aldolase, class II [EC:4.1.2.13]	237		
HAV_00347			28	K02199	3
HAV_00348	K01478	arcA; arginine deiminase [EC:3.5.3.6]	165		
HAV_00349	K00611	OTC, argF, argI; ornithine carbamoyltransferase [EC:2.1.3.3]	277		
HAV_00350			221		
HAV_00351	K00926	arcC; carbamate kinase [EC:2.7.2.2]	232		
HAV_00352					
HAV_00353			1	K03321	1

HAV_00354			2	K17723	1
HAV_00355			10	K19334	7
HAV_00356	K03655	recG; ATP-dependent DNA helicase RecG [EC:5.6.2.4]	232		
HAV_00357	K03723	mfd; transcription-repair coupling factor (superfamily II helicase) [EC:3.6.4.-]	377		
HAV_00358			95	K00721	6
HAV_00359	K01476	E3.5.3.1, rocF, arg; arginase [EC:3.5.3.1]	101		
HAV_00360					
HAV_00361	K16092	btuB; vitamin B12 transporter	69		
HAV_00363			29	K03327	3
HAV_00364			1	K18981	1
HAV_00365	K02073	metQ; D-methionine transport system substrate-binding protein	128		
HAV_00366	K02073	metQ; D-methionine transport system substrate-binding protein	115		
HAV_00367	K02072	metI; D-methionine transport system permease protein	114		
HAV_00368	K02071	metN; D-methionine transport system ATP-binding protein	147		
HAV_00369	K00615	E2.2.1.1, tktA, tktB; transketolase [EC:2.2.1.1]	269		
HAV_00370	K01783	rpe, RPE; ribulose-phosphate 3-epimerase [EC:5.1.3.1]	113		
HAV_00371			72		
HAV_00372			2	K03286	1
HAV_00373					
HAV_00374	K19286	nfrA2; FMN reductase [NAD(P)H] [EC:1.5.1.39]	77		
HAV_00375	K02804	nagE; N-acetylglucosamine PTS system EIICBA or EIICB component [EC:2.7.1.193]	198		
HAV_00376			2		
HAV_00377	K03652	MPG; DNA-3-methyladenine glycosylase [EC:3.2.2.21]	22		
HAV_00378			26		
HAV_00379	K03308	TC.NSS; neurotransmitter:Na+ symporter, NSS family	311		
HAV_00380	K00259	ald; alanine dehydrogenase [EC:1.4.1.1]	197		
HAV_00381					
HAV_00382	K03770	ppiD; peptidyl-prolyl cis-trans isomerase D [EC:5.2.1.8]	47		
HAV_00383	K01803	TPI, tpiA; triosephosphate isomerase (TIM) [EC:5.3.1.1]	122		
HAV_00384	K03075	secG; preprotein translocase subunit SecG	13		
HAV_00385	K01937	pyrG, CTPS; CTP synthase [EC:6.3.4.2]	316		
HAV_00386	K01627	kdsA; 2-dehydro-3-deoxyphosphooctonate aldolase (KDO 8-P synthase) [EC:2.5.1.5	187		
HAV_00387	K01689	ENO, eno; enolase [EC:4.2.1.11]	293		
HAV_00388			3	K00575	1
HAV_00389			1	K00865	1
HAV_00390			3	K23258	1
HAV_00391					
HAV_00392					
HAV_00393	K02445	glpT; MFS transporter, OPA family, glycerol-3-phosphate transporter	234		
HAV_00394			6	K03586	1
HAV_00395	K00161	PDHA, pdhA; pyruvate dehydrogenase E1 component alpha subunit [EC:1.2.4.1]	158		
HAV_00396	K00162	PDHB, pdhB; pyruvate dehydrogenase E1 component beta subunit [EC:1.2.4.1]	262		
HAV_00397	K00627	DLAT, aceF, pdhC; pyruvate dehydrogenase E2 component (dihydrolipoamide acet	158		
HAV_00398	K00382	DLD, lpd, pdhD; dihydrolipoamide dehydrogenase [EC:1.8.1.4]	297		
HAV_00399			2	K01814	1
HAV_00400			4	K06919	1
HAV_00401			2	K06951	1
HAV_00402			3		
HAV_00403	K01750	E4.3.1.12, ocd; ornithine cyclodeaminase [EC:4.3.1.12]	161		
HAV_00404			21	K15268	1
HAV_00405			3	K00341	1
HAV_00406			8	K03796	1
HAV_00407			12	K00799	4
HAV_00408	K01679	E4.2.1.2B, fumC, FH; fumarate hydratase, class II [EC:4.2.1.2]	261		
HAV_00409	K14445	SLC13A2_3_5; solute carrier family 13 (sodium-dependent dicarboxylate transport	159		
HAV_00410	K03644	lipA, LIAS, LIP1, LIP5; lipoyl synthase [EC:2.8.1.8]	177		
HAV_00411	K18588	COQ10; coenzyme Q-binding protein COQ10	49		
HAV_00412			29	K07266	1
HAV_00413	K01874	MARS, metG; methionyl-tRNA synthetase [EC:6.1.1.10]	276		
HAV_00414			1	K01854	1
HAV_00415			2	K03406	1
HAV_00416			1	K07025	1
HAV_00417			4	K11206	1
HAV_00418	K03424	tatD; TatD DNase family protein [EC:3.1.21.-]	143		
HAV_00419	K06167	phnP; phosphoribosyl 1,2-cyclic phosphate phosphodiesterase [EC:3.1.4.55]	88		
HAV_00420			2	K03284	1
HAV_00421			1	K23743	1
HAV_00422			12	K16381	1

HAV_00423	K03665	hflX; GTPase	90		
HAV_00424	K03666	hfq; host factor-I protein	37		
HAV_00425	K03499	trkA, ktrA, ktrC; trk/ktr system potassium uptake protein	76		
HAV_00426	K09159	cptB; antitoxin CptB	22		
HAV_00427					
HAV_00428			1	K02338	1
HAV_00429	K00240	sdhB, frdB; succinate dehydrogenase / fumarate reductase, iron-sulfur subunit [EC	181		
HAV_00430	K00239	sdhA, frdA; succinate dehydrogenase / fumarate reductase, flavoprotein subunit [E	409		
HAV_00431	K00242	sdhD, frdD; succinate dehydrogenase / fumarate reductase, membrane anchor sub	26		
HAV_00432	K00241	sdhC, frdC; succinate dehydrogenase / fumarate reductase, cytochrome b subunit	37		
HAV_00433	K07791	dcuA; anaerobic C4-dicarboxylate transporter DcuA	230		
HAV_00434	K07791	dcuA; anaerobic C4-dicarboxylate transporter DcuA	185		
HAV_00435	K01744	aspA; aspartate ammonia-lyase [EC:4.3.1.1]	227		
HAV_00436	K05540	dusB; tRNA-dihydrouridine synthase B [EC:1.-.-.-]	161		
HAV_00437	K12506	ispDF; 2-C-methyl-D-erythritol 4-phosphate cytidylyltransferase / 2-C-methyl-D-ery	139		
HAV_00438	K00748	lpxB; lipid-A-disaccharide synthase [EC:2.4.1.182]	159		
HAV_00439	K01759	GLO1, gloA; lactoylglutathione lyase [EC:4.4.1.5]	77		
HAV_00440	K06867	K06867; uncharacterized protein	11		
HAV_00441			79		
HAV_00442	K01607	pcaC; 4-carboxymuconolactone decarboxylase [EC:4.1.1.44]	71		
HAV_00443			21	K01928	1
HAV_00444	K00963	UGP2, galU, galF; UTP--glucose-1-phosphate uridylyltransferase [EC:2.7.7.9]	139		
HAV_00445	K01256	pepN; aminopeptidase N [EC:3.4.11.2]	319		
HAV_00446			48		
HAV_00447			2	K02020	1
HAV_00448	K00088	IMPDH, guaB; IMP dehydrogenase [EC:1.1.1.205]	107		
HAV_00449			2	K14059	1
HAV_00450	K01756	purB, ADSL; adenylosuccinate lyase [EC:4.3.2.2]	240		
HAV_00451	K01939	purA, ADSS; adenylosuccinate synthase [EC:6.3.4.4]	269		
HAV_00452	K03186	ubiX, bsdB, PAD1; flavin prenyltransferase [EC:2.5.1.129]	94		
HAV_00453					
HAV_00454					
HAV_00455	K04094	trmFO, gid; methylenetetrahydrofolate--tRNA-(uracil-5-)-methyltransferase [EC:2.1	214		
HAV_00456	K00648	fabH; 3-oxoacyl-[acyl-carrier-protein] synthase III [EC:2.3.1.180]	183		
HAV_00457	K03621	plsX; phosphate acyltransferase [EC:2.3.1.274]	187		
HAV_00458	K02911	RP-L32, MRPL32, rpmF; large subunit ribosomal protein L32	47		
HAV_00459			13	K00428	1
HAV_00460			231		
HAV_00461			224		
HAV_00462			5	K13652	1
HAV_00463			67	K01607	2
HAV_00464			10	K07251	1
HAV_00465			11	K06186	5
HAV_00466	K00946	thiL; thiamine-monophosphate kinase [EC:2.7.4.16]	125		
HAV_00467	K00600	glyA, SHMT; glycine hydroxymethyltransferase [EC:2.1.2.1]	243		
HAV_00468	K01808	rpiB; ribose 5-phosphate isomerase B [EC:5.3.1.6]	63		
HAV_00469	K00761	upp, UPRT; uracil phosphoribosyltransferase [EC:2.4.2.9]	83		
HAV_00470	K02824	uraA, pyrP; uracil permease	211		
HAV_00471			315		
HAV_00472			29	K03769	6
HAV_00473			72		
HAV_00474			37		
HAV_00475			2		
HAV_00476	K09949	lpxI; UDP-2,3-diacylglucosamine hydrolase [EC:3.6.1.54]	103		
HAV_00477	K00677	lpxA; UDP-N-acetylglucosamine acyltransferase [EC:2.3.1.129]	127		
HAV_00478	K02372	fabZ; 3-hydroxyacyl-[acyl-carrier-protein] dehydratase [EC:4.2.1.59]	57	K16363	5
HAV_00479	K02536	lpxD; UDP-3-O-[3-hydroxymyristoyl] glucosamine N-acyltransferase [EC:2.3.1.191]	117		
HAV_00480	K06142	hlpA, ompH; outer membrane protein	14		
HAV_00481	K01809	manA, MPI; mannose-6-phosphate isomerase [EC:5.3.1.8]	59		
HAV_00482			2	K07126	1
HAV_00483			2	K00928	1
HAV_00484	K07277	SAM50, TOB55, bamA; outer membrane protein insertion porin family	212		
HAV_00485	K11749	rseP; regulator of sigma E protease [EC:3.4.24.-]	111		
HAV_00486	K00099	dxr; 1-deoxy-D-xylulose-5-phosphate reductoisomerase [EC:1.1.1.267]	133		
HAV_00487			10	K07251	5
HAV_00488	K03811	pnuC; nicotinamide mononucleotide transporter	43		
HAV_00489	K01885	EARS, gltX; glutamyl-tRNA synthetase [EC:6.1.1.17]	231		
HAV_00490			54		

HAV_00491			4	K07448	1
HAV_00492	K03118	tatC; sec-independent protein translocase protein TatC	85		
HAV_00493			3	K07126	1
HAV_00494	K01887	RARS, argS; arginyl-tRNA synthetase [EC:6.1.1.19]	253		
HAV_00495	K15724	erpA; iron-sulfur cluster insertion protein	23		
HAV_00496	K01142	E3.1.11.2, xthA; exodeoxyribonuclease III [EC:3.1.11.2]	113		
HAV_00497			2	K08738	1
HAV_00498			20		
HAV_00499	K06926	K06926; uncharacterized protein	38		
HAV_00501			2	K07403	1
HAV_00502			1		
HAV_00503	K01662	dxs; 1-deoxy-D-xylulose-5-phosphate synthase [EC:2.2.1.7]	237		
HAV_00504	K13789	GGPS; geranylgeranyl diphosphate synthase, type II [EC:2.5.1.1 2.5.1.10 2.5.1.29]	36	K00795	22
HAV_00505	K03602	xseB; exodeoxyribonuclease VII small subunit [EC:3.1.11.6]	20		
HAV_00506	K01876	DARS2, aspS; aspartyl-tRNA synthetase [EC:6.1.1.12]	382		
HAV_00507			15		
HAV_00508			6	K02020	1
HAV_00509			2	K07320	1
HAV_00510			28		
HAV_00512			19		
HAV_00513			8	K01552	1
HAV_00514			8	K02670	1
HAV_00516			16	K03972	3
HAV_00517			29	K03671	1
HAV_00518					
HAV_00519			209		
HAV_00520			2	K00525	1
HAV_00521			2	K23257	1
HAV_00522			14	K09809	1
HAV_00523			14	K00703	1
HAV_00524			3	K01270	1
HAV_00525			20	K06857	1
HAV_00526			2	K03424	1
HAV_00527			2	K07459	1
HAV_00528			103		
HAV_00529			28	K06909	3
HAV_00530			3		
HAV_00531			3	K03629	1
HAV_00532			22	K03657	1
HAV_00533	K05569	mnhE, mrpE; multicomponent Na+:H+ antiporter subunit E	32		
HAV_00534	K05570	mnhF, mrpF; multicomponent Na+:H+ antiporter subunit F	12		
HAV_00535	K05571	mnhG, mrpG; multicomponent Na+:H+ antiporter subunit G	12	K05564	1
HAV_00536	K05566	mnhB, mrpB; multicomponent Na+:H+ antiporter subunit B	71		
HAV_00537	K05567	mnhC, mrpC; multicomponent Na+:H+ antiporter subunit C	32		
HAV_00538	K05568	mnhD, mrpD; multicomponent Na+:H+ antiporter subunit D	108		
HAV_00539	K05568	mnhD, mrpD; multicomponent Na+:H+ antiporter subunit D	209		
HAV_00540	K05568	mnhD, mrpD; multicomponent Na+:H+ antiporter subunit D	62		
HAV_00541			36		
HAV_00542					
HAV_00543			3	K09769	2
HAV_00544			4	K07058	1
HAV_00545	K01610	E4.1.1.49, pckA; phosphoenolpyruvate carboxykinase (ATP) [EC:4.1.1.49]	272		
HAV_00546			1	K15532	1
HAV_00547			12	K03406	1
HAV_00548	K04047	dps; starvation-inducible DNA-binding protein	61		
HAV_00549	K03760	eptA, pmrC; lipid A ethanolaminephosphotransferase [EC:2.7.8.43]	166		
HAV_00550					
HAV_00551			1	K01928	1
HAV_00552	K00009	mtlD; mannitol-1-phosphate 5-dehydrogenase [EC:1.1.1.17]	159		
HAV_00553					
HAV_00554	K02800	mtlA, cmtA; mannitol PTS system EIICBA or EIICB component [EC:2.7.1.197]	356		
HAV_00555					
HAV_00556			1	K03679	1
HAV_00557					
HAV_00558			1	K01953	1
HAV_00559			3	K07025	1
HAV_00560	K04095	fic; cell filamentation protein	60		
HAV_00561			6	K05606	4

HAV_00562	K03837	sdaC; serine transporter	191		
HAV_00563					
HAV_00564	K07552	bcr, tcaB; MFS transporter, DHA1 family, multidrug resistance protein	107		
HAV_00565			14		
HAV_00566	K03788	aphA; acid phosphatase (class B) [EC:3.1.3.2]	54		
HAV_00567			3		
HAV_00569	K00954	E2.7.7.3A, coaD, kdtB; pantetheine-phosphate adenylyltransferase [EC:2.7.7.3]	71		
HAV_00570	K02469	gyrA; DNA gyrase subunit A [EC:5.6.2.2]	464		
HAV_00571	K07276	K07276; uncharacterized protein	53		
HAV_00572	K03527	ispH, lytB; 4-hydroxy-3-methylbut-2-en-1-yl diphosphate reductase [EC:1.17.7.4]	174		
HAV_00573	K07323	mlaC; phospholipid transport system substrate-binding protein	11		
HAV_00574	K00970	pcnB; poly(A) polymerase [EC:2.7.7.19]	94		
HAV_00575	K07082	K07082; UPF0755 protein	89		
HAV_00576	K09458	fabF, OXSM, CEM1; 3-oxoacyl-[acyl-carrier-protein] synthase II [EC:2.3.1.179]	233		
HAV_00577	K02078	acpP; acyl carrier protein	46		
HAV_00578	K00059	fabG, OAR1; 3-oxoacyl-[acyl-carrier protein] reductase [EC:1.1.1.100]	138		
HAV_00579	K00645	fabD, MCAT, MCT1; [acyl-carrier-protein] S-malonyltransferase [EC:2.3.1.39]	132		
HAV_00580	K02990	RP-S6, MRPS6, rpsF; small subunit ribosomal protein S6	37		
HAV_00581	K02963	RP-S18, MRPS18, rpsR; small subunit ribosomal protein S18	21		
HAV_00582			1	K23509	1
HAV_00583	K02939	RP-L9, MRPL9, rplI; large subunit ribosomal protein L9	72		
HAV_00584	K02314	dnaB; replicative DNA helicase [EC:5.6.2.3]	183		
HAV_00585	K01775	alr; alanine racemase [EC:5.1.1.1]	127		
HAV_00586	K02427	rlmE, rrmJ, ftsJ; 23S rRNA (uridine2552-2'-O)-methyltransferase [EC:2.1.1.166]	94		
HAV_00587			3		
HAV_00588	K03722	dinG; ATP-dependent DNA helicase DinG [EC:5.6.2.3]	179		
HAV_00589	K02065	mlaF, linL, mkl; phospholipid/cholesterol/gamma-HCH transport system ATP-bindir	109		
HAV_00590	K02066	mlaE, linK; phospholipid/cholesterol/gamma-HCH transport system permease prot	193		
HAV_00591			28		
HAV_00592	K04755	fdx; ferredoxin, 2Fe-2S	33	K22071	11
HAV_00593	K04044	hscA; molecular chaperone HscA	252		
HAV_00594	K04082	hscB, HSCB, HSC20; molecular chaperone HscB	20		
HAV_00595	K13628	iscA; iron-sulfur cluster assembly protein	64	K22063	3
HAV_00596	K04488	iscU, nifU; nitrogen fixation protein NifU and related proteins	95		
HAV_00597	K04487	iscS, NFS1; cysteine desulfurase [EC:2.8.1.7]	253		
HAV_00598	K13643	iscR; Rrf2 family transcriptional regulator, iron-sulfur cluster assembly transcriptior	59		
HAV_00599	K07018	K07018; uncharacterized protein	87		
HAV_00600	K01866	YARS, tyrS; tyrosyl-tRNA synthetase [EC:6.1.1.1]	209		
HAV_00601			3	K13714	1
HAV_00602	K02836	prfB; peptide chain release factor 2	145		
HAV_00603	K05366	mrcA; penicillin-binding protein 1A [EC:2.4.1.129 3.4.16.4]	228		
HAV_00604	K01448	amiABC; N-acetylmuramoyl-L-alanine amidase [EC:3.5.1.28]	63		
HAV_00605	K08300	rne; ribonuclease E [EC:3.1.26.12]	191		
HAV_00606			82		
HAV_00607			4	K03286	1
HAV_00609	K02434	gatB, PET112; aspartyl-tRNA(Asn)/glutamyl-tRNA(Gln) amidotransferase subunit B	273		
HAV_00610	K02433	gatA, QRSL1; aspartyl-tRNA(Asn)/glutamyl-tRNA(Gln) amidotransferase subunit A [	231		
HAV_00611	K02435	gatC, GATC; aspartyl-tRNA(Asn)/glutamyl-tRNA(Gln) amidotransferase subunit C [E	28		
HAV_00612	K07447	ruvX; putative pre-16S rRNA nuclease [EC:3.1.-.-]	57		
HAV_00613	K08591	plsY; acyl phosphate:glycerol-3-phosphate acyltransferase [EC:2.3.1.275]	104		
HAV_00614	K04096	smf; DNA processing protein	135		
HAV_00615	K03168	topA; DNA topoisomerase I [EC:5.6.2.1]	334		
HAV_00616	K12573	rnr, vacB; ribonuclease R [EC:3.1.13.1]	121		
HAV_00617	K02913	RP-L33, MRPL33, rpmG; large subunit ribosomal protein L33	52		
HAV_00618					
HAV_00619	K06180	rluD; 23S rRNA pseudouridine1911/1915/1917 synthase [EC:5.4.99.23]	102		
HAV_00620			1	K21555	1
HAV_00621			20		
HAV_00622	K08303	prtC, trhP; U32 family peptidase [EC:3.4.-.-]	355		
HAV_00623	K01493	comEB; dCMP deaminase [EC:3.5.4.12]	73		
HAV_00624					
HAV_00625	K03469	rnhA, RNASEH1; ribonuclease HI [EC:3.1.26.4]	98		
HAV_00626	K07735	algH; putative transcriptional regulator	49		
HAV_00627			3	K10201	1
HAV_00628			30		
HAV_00629	K03215	rumA; 23S rRNA (uracil1939-C5)-methyltransferase [EC:2.1.1.190]	79		
HAV_00630			134		
HAV_00631	K01972	E6.5.1.2, ligA, ligB; DNA ligase (NAD+) [EC:6.5.1.2]	190		

HAV_00632	K03631	recN; DNA repair protein RecN (Recombination protein N)	178		
HAV_00633	K05807	bamD; outer membrane protein assembly factor BamD	59		
HAV_00634	K02535	lpxC; UDP-3-O-[3-hydroxymyristoyl] N-acetylglucosamine deacetylase [EC:3.5.1.10	163		
HAV_00635			61		
HAV_00636	K03531	ftsZ; cell division protein FtsZ	91		
HAV_00637	K03590	ftsA; cell division protein FtsA	11		
HAV_00638	K03589	ftsQ; cell division protein FtsQ	20		
HAV_00639	K01921	ddl; D-alanine-D-alanine ligase [EC:6.3.2.4]	112		
HAV_00640	K00075	murB; UDP-N-acetylmuramate dehydrogenase [EC:1.3.1.98]	121		
HAV_00641	K01924	murC; UDP-N-acetylmuramate--alanine ligase [EC:6.3.2.8]	194		
HAV_00642	K02563	murG; UDP-N-acetylglucosamine--N-acetylmuramyl-(pentapeptide) pyrophosphory	76		
HAV_00643	K03588	ftsW, spoVE; cell division protein FtsW	166		
HAV_00644	K01925	murD; UDP-N-acetylmuramoylalanine--D-glutamate ligase [EC:6.3.2.9]	120		
HAV_00645	K01000	mraY; phospho-N-acetylmuramoyl-pentapeptide-transferase [EC:2.7.8.13]	135		
HAV_00646	K01929	murF; UDP-N-acetylmuramoyl-tripeptide--D-alanyl-D-alanine ligase [EC:6.3.2.10]	106		
HAV_00647	K01928	murE; UDP-N-acetylmuramoyl-L-alanyl-D-glutamate--2,6-diaminopimelate ligase [E	133	K15792	8
HAV_00648	K03587	ftsI; cell division protein FtsI (penicillin-binding protein 3) [EC:3.4.16.4]	152		
HAV_00649			7	K03587	1
HAV_00650	K03438	mraW, rsmH; 16S rRNA (cytosine1402-N4)-methyltransferase [EC:2.1.1.199]	109		
HAV_00651					
HAV_00654			6	K00036	
HAV_00656	K08996	yagU; putative membrane protein	105		
HAV_00657	K08996	yagU; putative membrane protein	103		
HAV_00658	K09862	K09862; uncharacterized protein	10		
HAV_00659	K08301	rng, cafA; ribonuclease G [EC:3.1.26.-]	36		
HAV_00660	K06287	yhdE; nucleoside triphosphate pyrophosphatase [EC:3.6.1.-]	92		
HAV_00661	K02518	infA; translation initiation factor IF-1	22		
HAV_00662	K00790	murA; UDP-N-acetylglucosamine 1-carboxyvinyltransferase [EC:2.5.1.7]	277		
HAV_00663			4	K01991	
HAV_00665	K03216	trmL, cspR; tRNA (cytidine/uridine-2'-O-)-methyltransferase [EC:2.1.1.207]	102		
HAV_00666	K00791	miaA, TRIT1; tRNA dimethylallyltransferase [EC:2.5.1.75]	77		
HAV_00667	K03569	mreB; rod shape-determining protein MreB and related proteins	198		
HAV_00668	K03570	mreC; rod shape-determining protein MreC	26		
HAV_00669					
HAV_00670	K05515	mrdA; penicillin-binding protein 2 [EC:3.4.16.4]	183		
HAV_00671	K05837	rodA, mrdB; rod shape determining protein RodA	189		
HAV_00672	K07263	pqqL; zinc protease [EC:3.4.24.-]	45		
HAV_00673	K07263	pqqL; zinc protease [EC:3.4.24.-]	94		
HAV_00674			6	K10441	1
HAV_00675	K03101	lspA; signal peptidase II [EC:3.4.23.36]	40		
HAV_00676	K01870	IARS, ileS; isoleucyl-tRNA synthetase [EC:6.1.1.5]	428		
HAV_00677	K11753	ribF; riboflavin kinase / FMN adenylyltransferase [EC:2.7.1.26 2.7.7.2]	93		
HAV_00678					
HAV_00679	K06020	ettA; energy-dependent translational throttle protein EttA	318		
HAV_00680					
HAV_00681			70		
HAV_00682					
HAV_00683			15	K17278	1
HAV_00684			3	K02348	1
HAV_00685	K06956	K06956; uncharacterized protein	234		
HAV_00686			137	K18367	35
HAV_00687			61		
HAV_00688	K00384	trxB, TRR; thioredoxin reductase (NADPH) [EC:1.8.1.9]	162		
HAV_00689	K02114	ATPF1E, atpC; F-type H+-transporting ATPase subunit epsilon	12		
HAV_00690	K02112	ATPF1B, atpD; F-type H+/Na+-transporting ATPase subunit beta [EC:7.1.2.2 7.2.2.1	325		
HAV_00691	K02115	ATPF1G, atpG; F-type H+-transporting ATPase subunit gamma	56		
HAV_00692	K02111	ATPF1A, atpA; F-type H+/Na+-transporting ATPase subunit alpha [EC:7.1.2.2 7.2.2.1	313		
HAV_00693	K02113	ATPF1D, atpH; F-type H+-transporting ATPase subunit delta	39		
HAV_00694	K04066	priA; primosomal protein N' (replication factor Y) (superfamily II helicase) [EC:3.6.4	284		
HAV_00695	K03733	xerC; integrase/recombinase XerC	109		
HAV_00696			11	K17837	3
HAV_00697					
HAV_00698	K00426	cydB; cytochrome bd ubiquinol oxidase subunit II [EC:7.1.1.7]	181		
HAV_00699	K00425	cydA; cytochrome bd ubiquinol oxidase subunit I [EC:7.1.1.7]	316		
HAV_00700	K00133	asd; aspartate-semialdehyde dehydrogenase [EC:1.2.1.11]	147		
HAV_00701	K02884	RP-L19, MRPL19, rplS; large subunit ribosomal protein L19	82		
HAV_00702	K00554	trmD; tRNA (guanine37-N1)-methyltransferase [EC:2.1.1.228]	105		
HAV_00703	K02860	rimM; 16S rRNA processing protein RimM	48		

HAV_00704	K02959	RP-S16, MRPS16, rpsP; small subunit ribosomal protein S16	35		
HAV_00705	K03106	SRP54, ffh; signal recognition particle subunit SRP54 [EC:3.6.5.4]	171		
HAV_00706	K01778	dapF; diaminopimelate epimerase [EC:5.1.1.7]	48		
HAV_00707	K18707	mtaB; threonylcarbamoyladenosine tRNA methylthiotransferase MtaB [EC:2.8.4.5]	166		
HAV_00708			6	K05606	4
HAV_00709	K09125	yhhQ; queuosine precursor transporter	11		
HAV_00710			1	K07052	1
HAV_00711			5	K07729	1
HAV_00712			11	K11434	3
HAV_00713	K25564	sznF, stzF; nitrosourea synthase [EC:1.14.13.250]	199		
HAV_00714					
HAV_00715			77		
HAV_00716			128		
HAV_00717	K00567	ogt, MGMT; methylated-DNA-[protein]-cysteine S-methyltransferase [EC:2.1.1.63]	29		
HAV_00718			57	K08217	3
HAV_00719	K13529	ada-alkA; AraC family transcriptional regulator, regulatory protein of adaptative re	12	K01247	5
HAV_00720	K01854	glf; UDP-galactopyranose mutase [EC:5.4.99.9]	161		
HAV_00721	K03110	ftsY; fused signal recognition particle receptor	89		
HAV_00722	K03559	exbD; biopolymer transport protein ExbD	18	K03560	3
HAV_00723	K03561	exbB; biopolymer transport protein ExbB	20		
HAV_00724	K03832	tonB; periplasmic protein TonB	10		
HAV_00725	K16087	TC.FEV.OM3, tbpA, hemR, lbpA, hpuB, bhuR, hugA, hmbR; hemoglobin/transferrin/	108		
HAV_00726					
HAV_00727					
HAV_00728	K01591	pyrF; orotidine-5'-phosphate decarboxylase [EC:4.1.1.23]	60		
HAV_00729	K17828	pyrDI; dihydroorotate dehydrogenase (NAD+) catalytic subunit [EC:1.3.1.14]	21	K00254	5
HAV_00730			102		
HAV_00731			3	K07728	1
HAV_00732			16	K11530	3
HAV_00734			8	K03275	2
HAV_00736					
HAV_00737	K01992	ABC-2.P; ABC-2 type transport system permease protein	101		
HAV_00738	K03565	recX; regulatory protein	24		
HAV_00739			26		
HAV_00740	K00919	ispE; 4-diphosphocytidyl-2-C-methyl-D-erythritol kinase [EC:2.7.1.148]	109		
HAV_00741			79		
HAV_00742	K21929	udg; uracil-DNA glycosylase [EC:3.2.2.27]	80		
HAV_00743	K08309	slt; soluble lytic murein transglycosylase [EC:4.2.2.-]	69		
HAV_00744			1	K15987	1
HAV_00745			10	K03611	7
HAV_00746					
HAV_00747			1	K00325	1
HAV_00748			73		
HAV_00749	K00927	PGK, pgk; phosphoglycerate kinase [EC:2.7.2.3]	271		
HAV_00750	K01174	nuc; micrococcal nuclease [EC:3.1.31.1]	33		
HAV_00751					
HAV_00752			58		
HAV_00753			76		
HAV_00754			14	K03415	1
HAV_00755			34		
HAV_00756	K02116	atpI; ATP synthase protein I	18		
HAV_00757	K02108	ATPF0A, atpB; F-type H+-transporting ATPase subunit a	132		
HAV_00758					
HAV_00759	K02109	ATPF0B, atpF; F-type H+-transporting ATPase subunit b	11		
HAV_00760	K02109	ATPF0B, atpF; F-type H+-transporting ATPase subunit b	11		
HAV_00761	K01736	aroC; chorismate synthase [EC:4.2.3.5]	228		
HAV_00762	K12542	lapC; membrane fusion protein, adhesin transport system	132		
HAV_00763	K12541	lapB; ATP-binding cassette, subfamily C, bacterial LapB	168		
HAV_00764					
HAV_00765			2	K21449	1
HAV_00766	K12543	lapE; outer membrane protein, adhesin transport system	38	K12340	2
HAV_00767					
HAV_00768			2		
HAV_00769					
HAV_00770	K00769	gpt; xanthine phosphoribosyltransferase [EC:2.4.2.22]	52		
HAV_00773	K02238	comEC; competence protein ComEC	72		
HAV_00775	K06911	PIR; quercetin 2,3-dioxygenase [EC:1.13.11.24]	102		
HAV_00776	K05592	deaD, cshA; ATP-dependent RNA helicase DeaD [EC:3.6.4.13]	55	K11927	42

HAV_00777	K09760	rmuC; DNA recombination protein RmuC	104		
HAV_00778	K04750	phnB; PhnB protein	41		
HAV_00779	K04750	phnB; PhnB protein	21		
HAV_00780			2	K04080	1
HAV_00781	K04080	ibpA; molecular chaperone IbpA	11		
HAV_00782	K04758	feoA; ferrous iron transport protein A	16		
HAV_00783	K04759	feoB; ferrous iron transport protein B	183		
HAV_00785	K03431	glmM; phosphoglucosamine mutase [EC:5.4.2.10]	238		
HAV_00786	K03798	ftsH, hflB; cell division protease FtsH [EC:3.4.24.-]	283		
HAV_00787	K04075	tilS, mesJ; tRNA(Ile)-lysidine synthase [EC:6.3.4.19]	60		
HAV_00788			2	K01423	1
HAV_00789	K03640	pal; peptidoglycan-associated lipoprotein	51		
HAV_00790	K03641	tolB; TolB protein	77		
HAV_00791			2	K02482	1
HAV_00792	K03560	tolR; biopolymer transport protein TolR	41	K03559	1
HAV_00793	K03562	tolQ; biopolymer transport protein TolQ	48		
HAV_00794	K07107	ybgC; acyl-CoA thioester hydrolase [EC:3.1.2.-]	28	K01075	3
HAV_00795	K03551	ruvB; holliday junction DNA helicase RuvB [EC:5.6.2.4]	194		
HAV_00796	K03550	ruvA; holliday junction DNA helicase RuvA [EC:5.6.2.4]	62		
HAV_00797	K01159	ruvC; crossover junction endodeoxyribonuclease RuvC [EC:3.1.21.10]	63		
HAV_00798			133		
HAV_00799			24		
HAV_00800	K01934	MTHFS; 5-formyltetrahydrofolate cyclo-ligase [EC:6.3.3.2]	58		
HAV_00801	K09888	zapA; cell division protein ZapA	10		
HAV_00802			2	K21826	1
HAV_00803	K00134	GAPDH, gapA; glyceraldehyde 3-phosphate dehydrogenase (phosphorylating) [EC:1	236		
HAV_00804					
HAV_00805	K02356	efp; elongation factor P	110		
HAV_00806	K01092	E3.1.3.25, IMPA, suhB; myo-inositol-1(or 4)-monophosphatase [EC:3.1.3.25]	70		
HAV_00807			2	K13890	1
HAV_00808	K08151	tetA; MFS transporter, DHA1 family, tetracycline resistance protein	37		
HAV_00809	K02909	RP-L31, rpmE; large subunit ribosomal protein L31	47		
HAV_00810	K08151	tetA; MFS transporter, DHA1 family, tetracycline resistance protein	33	K08153	16
HAV_00811	K08151	tetA; MFS transporter, DHA1 family, tetracycline resistance protein	24	K08153	17
HAV_00812	K09987	K09987; uncharacterized protein	43		
HAV_00813	K21071	pfk, pfp; ATP-dependent phosphofructokinase / diphosphate-dependent phosphof	188		
HAV_00814	K02970	RP-S21, MRPS21, rpsU; small subunit ribosomal protein S21	46		
HAV_00815			33	K08153	1
HAV_00816	K03797	E3.4.21.102, prc, ctpA; carboxyl-terminal processing protease [EC:3.4.21.102]	63		
HAV_00817	K00275	pdxH, PNPO; pyridoxamine 5'-phosphate oxidase [EC:1.4.3.5]	73		
HAV_00818			2	K08963	1
HAV_00819	K00208	fabI; enoyl-[acyl-carrier protein] reductase I [EC:1.3.1.9 1.3.1.10]	161		
HAV_00820	K04771	degP, htrA; serine protease Do [EC:3.4.21.107]	123		
HAV_00821	K03593	mrp, NUBPL; ATP-binding protein involved in chromosome partitioning	79		
HAV_00822			293		
HAV_00823	K11085	msbA; ATP-binding cassette, subfamily B, bacterial MsbA [EC:7.5.2.6]	200		
HAV_00824			40		
HAV_00825	K02527	kdtA, waaA; 3-deoxy-D-manno-octulosonic-acid transferase [EC:2.4.99.12 2.4.99.1	84		
HAV_00826	K00912	lpxK; tetraacyldisaccharide 4'-kinase [EC:2.7.1.130]	117		
HAV_00827	K02517	lpxL, htrB; Kdo2-lipid IVA lauroyltransferase/acyltransferase [EC:2.3.1.241 2.3.1.-]	72		
HAV_00828	K03601	xseA; exodeoxyribonuclease VII large subunit [EC:3.1.11.6]	91		
HAV_00829	K01810	GPI, pgi; glucose-6-phosphate isomerase [EC:5.3.1.9]	288		
HAV_00830	K03572	mutL; DNA mismatch repair protein MutL	187		
HAV_00831	K06890	K06890; uncharacterized protein	152		
HAV_00832	K06173	truA, PUS1; tRNA pseudouridine38-40 synthase [EC:5.4.99.12]	168		
HAV_00833	K03466	ftsK, spoIIIE; DNA segregation ATPase FtsK/SpoIIIE, S-DNA-T family	177		
HAV_00834			27		
HAV_00835			9	K07658	2
HAV_00836	K06997	yggS, PROSC; PLP dependent protein	66		
HAV_00837			2	K01937	1
HAV_00838			40		
HAV_00839	K01869	LARS, leuS; leucyl-tRNA synthetase [EC:6.1.1.4]	429		
HAV_00840					
HAV_00841	K02340	holA; DNA polymerase III subunit delta [EC:2.7.7.7]	74		
HAV_00842	K00798	MMAB, pduO; cob(I)alamin adenosyltransferase [EC:2.5.1.17]	47		
HAV_00843	K01262	pepP; Xaa-Pro aminopeptidase [EC:3.4.11.9]	179		
HAV_00844			3	K13051	1
HAV_00845					

HAV_00846	K09812	ftsE; cell division transport system ATP-binding protein	97		
HAV_00847					
HAV_00848	K00655	plsC; 1-acyl-sn-glycerol-3-phosphate acyltransferase [EC:2.3.1.51]	71		
HAV_00849	K03465	thyX, thy1; thymidylate synthase (FAD) [EC:2.1.1.148]	206		
HAV_00854	K09985	K09985; uncharacterized protein	31		
HAV_00855			1	K01613	1
HAV_00856			2	K17759	1
HAV_00857					
HAV_00858	K02919	RP-L36, MRPL36, rpmJ; large subunit ribosomal protein L36	41		
HAV_00859	K03839	fldA, nifF, isiB; flavodoxin I	26		
HAV_00860	K07568	queA; S-adenosylmethionine:tRNA ribosyltransferase-isomerase [EC:2.4.99.17]	165		
HAV_00861	K00773	tgt; queuine tRNA-ribosyltransferase [EC:2.4.2.29]	260		
HAV_00862	K18979	queG; epoxyqueuosine reductase [EC:1.17.99.6]	90		
HAV_00863			1	K06397	1
HAV_00865	K01990	ABC-2.A; ABC-2 type transport system ATP-binding protein	131		
HAV_00866			8		
HAV_00867	K02335	polA; DNA polymerase I [EC:2.7.7.7]	315		
HAV_00868			15	K02242	1
HAV_00869	K09791	K09791; uncharacterized protein	23		
HAV_00871	K09805	K09805; uncharacterized protein	88		
HAV_00872			7	K07402	1
HAV_00873			42		
HAV_00874	K01520	dut, DUT; dUTP pyrophosphatase [EC:3.6.1.23]	114		
HAV_00875	K03688	ubiB, aarF; ubiquinone biosynthesis protein	128		
HAV_00876	K03183	ubiE; demethylmenaquinone methyltransferase / 2-methoxy-6-polyprenyl-1,4-ben	124		
HAV_00877	K10563	mutM, fpg; formamidopyrimidine-DNA glycosylase [EC:3.2.2.23 4.2.99.18]	107		
HAV_00878	K02968	RP-S20, rpsT; small subunit ribosomal protein S20	42		
HAV_00879	K02313	dnaA; chromosomal replication initiator protein	199		
HAV_00880	K02338	dnaN; DNA polymerase III subunit beta [EC:2.7.7.7]	145		
HAV_00881	K03629	recF; DNA replication and repair protein RecF	90		
HAV_00882			185		
HAV_00883	K02470	gyrB; DNA gyrase subunit B [EC:5.6.2.2]	475		
HAV_00884			4	K05366	1
HAV_00885	K06861	lptB; lipopolysaccharide export system ATP-binding protein [EC:7.5.2.5]	139		
HAV_00886					
HAV_00887	K06041	kdsD, kpsF; arabinose-5-phosphate isomerase [EC:5.3.1.13]	141		
HAV_00888	K03684	rnd; ribonuclease D [EC:3.1.13.5]	113		
HAV_00889			4	K07071	1
HAV_00890	K01626	E2.5.1.54, aroF, aroG, aroH; 3-deoxy-7-phosphoheptulonate synthase [EC:2.5.1.54]	114		
HAV_00891	K00763	pncB, NAPRT1; nicotinate phosphoribosyltransferase [EC:6.3.4.21]	185		
HAV_00892	K01916	nadE; NAD+ synthase [EC:6.3.1.5]	149	K01950	34
HAV_00893	K01885	EARS, gltX; glutamyl-tRNA synthetase [EC:6.1.1.17]	197		
HAV_00894	K01883	CARS, cysS; cysteinyl-tRNA synthetase [EC:6.1.1.16]	213		
HAV_00895	K09160	K09160; uncharacterized protein	75		
HAV_00896			48		
HAV_00897	K03799	htpX; heat shock protein HtpX [EC:3.4.24.-]	132		
HAV_00898			85		
HAV_00899	K09780	K09780; uncharacterized protein	44		
HAV_00900	K00057	gpsA; glycerol-3-phosphate dehydrogenase (NAD(P)+) [EC:1.1.1.94]	120		
HAV_00901	K01409	KAE1, tsaD, QRI7; N6-L-threonylcarbamoyladenine synthase [EC:2.3.1.234]	162		
HAV_00902			2	K00785	1
HAV_00903			2	K02498	2
HAV_00904			32		
HAV_00905	K00459	ncd2, npd; nitronate monooxygenase [EC:1.13.12.16]	236		
HAV_00906	K00962	pnp, PNPT1; polyribonucleotide nucleotidyltransferase [EC:2.7.7.8]	378		
HAV_00907	K02956	RP-S15, MRPS15, rpsO; small subunit ribosomal protein S15	64		
HAV_00908	K03177	truB, PUS4, TRUB1; tRNA pseudouridine55 synthase [EC:5.4.99.25]	101		
HAV_00909	K02834	rbfA; ribosome-binding factor A	45		
HAV_00910	K02519	infB, MTIF2; translation initiation factor IF-2	192		
HAV_00911	K02600	nusA; transcription termination/antitermination protein NusA	196		
HAV_00912	K09748	rimP; ribosome maturation factor RimP	32		
HAV_00913	K03439	trmB, METTL1, TRM8; tRNA (guanine-N7-)-methyltransferase [EC:2.1.1.33]	65		
HAV_00914	K00789	metK, MAT; S-adenosylmethionine synthetase [EC:2.5.1.6]	220		
HAV_00915			37	K03820	1
HAV_00916	K03820	lnt; apolipoprotein N-acyltransferase [EC:2.3.1.269]	73		
HAV_00917	K06189	corC, tlyC; hemolysin (HlyC) family protein	79		
HAV_00918	K07042	ybeY, yqfG; probable rRNA maturation factor	19		
HAV_00919	K06168	miaB; tRNA-2-methylthio-N6-dimethylallyladenosine synthase [EC:2.8.4.3]	240		

HAV_00920	K14742	tsaB; tRNA threonylcarbamoyladenosine biosynthesis protein TsaB	21		
HAV_00921	K03796	bax; Bax protein	33		
HAV_00922			77	K22074	5
HAV_00923	K01867	WARS, trpS; tryptophanyl-tRNA synthetase [EC:6.1.1.2]	196		
HAV_00924	K03980	murJ, mviN; putative peptidoglycan lipid II flippase	128		
HAV_00925	K03555	mutS; DNA mismatch repair protein MutS	312		
HAV_00926					
HAV_00927			5	K03286	2
HAV_00928					
HAV_00929	K01507	ppa; inorganic pyrophosphatase [EC:3.6.1.1]	102		
HAV_00930					
HAV_00931			3	K18889	1
HAV_00932			34		
HAV_00933			51		
HAV_00934	K02342	dnaQ; DNA polymerase III subunit epsilon [EC:2.7.7.7]	83		
HAV_00935	K00859	coaE; dephospho-CoA kinase [EC:2.7.1.24]	48		
HAV_00936	K00014	aroE; shikimate dehydrogenase [EC:1.1.1.25]	55		
HAV_00937	K03628	rho; transcription termination factor Rho	298		
HAV_00938	K03650	mnmE, trmE, MSS1; tRNA modification GTPase [EC:3.6.-.-]	145		
HAV_00939			31	K03321	1
HAV_00940	K20534	gtrB, csbB; polyisoprenyl-phosphate glycosyltransferase [EC:2.4.-.-]	134		
HAV_00941			15	K07152	1
HAV_00942			15	K18697	1
HAV_00943	K03495	gidA, mnmG, MTO1; tRNA uridine 5-carboxymethylaminomethyl modification enzy	320		
HAV_00944			3	K00057	1
HAV_00945	K03501	gidB, rsmG; 16S rRNA (guanine527-N7)-methyltransferase [EC:2.1.1.170]	32		
HAV_00946	K03497	parB, spo0J; ParB family transcriptional regulator, chromosome partitioning protei	84		
HAV_00947	K03496	parA, soj; chromosome partitioning protein	146		
HAV_00948	K11991	tadA; tRNA(adenine34) deaminase [EC:3.5.4.33]	61		
HAV_00949	K06178	rluB; 23S rRNA pseudouridine2605 synthase [EC:5.4.99.22]	54		
HAV_00950	K08316	rsmD; 16S rRNA (guanine966-N2)-methyltransferase [EC:2.1.1.171]	56		
HAV_00951			2	K00943	2
HAV_00952			6		
HAV_00953			22	K01939	1
HAV_00954			14	K21686	1
HAV_00955			1	K03091	1
HAV_00956	K02503	HINT1, hinT, hit; histidine triad (HIT) family protein	58		
HAV_00957	K01419	hslV, clpQ; ATP-dependent HslUV protease, peptidase subunit HslV [EC:3.4.25.2]	112		
HAV_00958	K03667	hslU; ATP-dependent HslUV protease ATP-binding subunit HslU	252		
HAV_00959	K07391	comM; magnesium chelatase family protein	241		
HAV_00960			15		
HAV_00961	K02040	pstS; phosphate transport system substrate-binding protein	137		
HAV_00962	K02037	pstC; phosphate transport system permease protein	162		
HAV_00963	K02038	pstA; phosphate transport system permease protein	152		
HAV_00964	K02036	pstB; phosphate transport system ATP-binding protein [EC:7.3.2.1]	165		
HAV_00965	K02039	phoU; phosphate transport system protein	46		
HAV_00966			1	K09800	1
HAV_00967	K00625	E2.3.1.8, pta; phosphate acetyltransferase [EC:2.3.1.8]	97		
HAV_00968	K00925	ackA; acetate kinase [EC:2.7.2.1]	170		
HAV_00969					
HAV_00970			11	K21402	1
HAV_00971	K01784	galE, GALE; UDP-glucose 4-epimerase [EC:5.1.3.2]	126	K12448	29
HAV_00972	K10773	NTH; endonuclease III [EC:4.2.99.18]	109		
HAV_00973	K03686	dnaJ; molecular chaperone DnaJ	145		
HAV_00974	K04043	dnaK, HSPA9; molecular chaperone DnaK	433		
HAV_00975	K03687	GRPE; molecular chaperone GrpE	36		
HAV_00976	K01295	cpg; glutamate carboxypeptidase [EC:3.4.17.11]	52		
HAV_00977	K00989	rph; ribonuclease PH [EC:2.7.7.56]	183		
HAV_00978	K01519	rdgB, ITPA; XTP/dITP diphosphohydrolase [EC:3.6.1.66]	96		
HAV_00979			121		
HAV_00980	K14623	dinD; DNA-damage-inducible protein D	125		
HAV_00981			125		
HAV_00982			39		
HAV_00983	K07056	rsmI; 16S rRNA (cytidine1402-2'-O)-methyltransferase [EC:2.1.1.198]	106		
HAV_00984	K07460	yraN; putative endonuclease	30		
HAV_00985	K01920	gshB; glutathione synthase [EC:6.3.2.3]	150		
HAV_00986			43		
HAV_00987	K00604	MTFMT, fmt; methionyl-tRNA formyltransferase [EC:2.1.2.9]	119		

HAV_00988	K01462	PDF, def; peptide deformylase [EC:3.5.1.88]	99		
HAV_00989	K06187	recR; recombination protein RecR	88		
HAV_00990	K09747	ebfC; nucleoid-associated protein EbfC	45		
HAV_00991	K09125	yhhQ; queuosine precursor transporter	21		
HAV_00992	K23993	fruB; multiphosphoryl transfer protein [EC:2.7.3.9 2.7.1.202]	229		
HAV_00993	K02804	nagE; N-acetylglucosamine PTS system EIICBA or EIICB component [EC:2.7.1.193]	210		
HAV_00994	K02564	nagB, GNPDA; glucosamine-6-phosphate deaminase [EC:3.5.99.6]	190		
HAV_00995					
HAV_00996	K01443	nagA, AMDHD2; N-acetylglucosamine-6-phosphate deacetylase [EC:3.5.1.25]	218		
HAV_00997	K07501	K07501; 3'-5' exonuclease	118		
HAV_00998			141		
HAV_00999	K01895	ACSS1_2, acs; acetyl-CoA synthetase [EC:6.2.1.1]	313		
HAV_01000			18	K12342	1
HAV_01001	K02343	dnaX; DNA polymerase III subunit gamma/tau [EC:2.7.7.7]	121		
HAV_01003			4	K03497	1
HAV_01004	K06223	dam; DNA adenine methylase [EC:2.1.1.72]	125		
HAV_01005	K06941	rlmN; 23S rRNA (adenine2503-C2)-methyltransferase [EC:2.1.1.192]	155		
HAV_01006	K00979	kdsB; 3-deoxy-manno-octulosonate cytidylyltransferase (CMP-KDO synthetase) [EC	117		
HAV_01007	K13893	yejA; microcin C transport system substrate-binding protein	62		
HAV_01008	K13894	yejB; microcin C transport system permease protein	127		
HAV_01009	K13895	yejE; microcin C transport system permease protein	72		
HAV_01010	K02031	ddpD; peptide/nickel transport system ATP-binding protein	30	K13896	19
	K02032	ddpF; peptide/nickel transport system ATP-binding protein			
HAV_01011	K06153	bacA; undecaprenyl-diphosphatase [EC:3.6.1.27]	105		
HAV_01012	K14170	pheA; chorismate mutase / prephenate dehydratase [EC:5.4.99.5 4.2.1.51]	12		
HAV_01013	K01069	gloB, gloC, HAGH; hydroxyacylglutathione hydrolase [EC:3.1.2.6]	73		
HAV_01014	K03784	deoD; purine-nucleoside phosphorylase [EC:2.4.2.1]	140		
HAV_01015			27	K09809	3
HAV_01016	K01839	deoB; phosphopentomutase [EC:5.4.2.7]	222		
HAV_01017			248		
HAV_01018	K01868	TARS, thrS; threonyl-tRNA synthetase [EC:6.1.1.3]	351		
HAV_01019	K02520	infC, MTIF3; translation initiation factor IF-3	70		
HAV_01020	K02916	RP-L35, MRPL35, rpmI; large subunit ribosomal protein L35	37		
HAV_01021	K02887	RP-L20, MRPL20, rplT; large subunit ribosomal protein L20	78		
HAV_01022	K01889	FARSA, pheS; phenylalanyl-tRNA synthetase alpha chain [EC:6.1.1.20]	150		
HAV_01023	K01890	FARSB, pheT; phenylalanyl-tRNA synthetase beta chain [EC:6.1.1.20]	259		
HAV_01024			128		
HAV_01025			8	K02237	1
HAV_01026	K01243	mtnN, mtn, pfs; adenosylhomocysteine nucleosidase [EC:3.2.2.9]	87		
HAV_01027	K01183	E3.2.1.14; chitinase [EC:3.2.1.14]	75		
HAV_01028	K01183	E3.2.1.14; chitinase [EC:3.2.1.14]	10		
HAV_01029	K04077	groEL, HSPD1; chaperonin GroEL	409		
HAV_01030	K04078	groES, HSPE1; chaperonin GroES	86		
HAV_01031			133	K19349	8
HAV_01032	K03657	uvrD, pcrA; ATP-dependent DNA helicase UvrD/PcrA [EC:5.6.2.4]	295		
HAV_01033	K00525	E1.17.4.1A, nrdA, nrdE; ribonucleoside-diphosphate reductase alpha chain [EC:1.17	347		
HAV_01034	K00526	E1.17.4.1B, nrdB, nrdF; ribonucleoside-diphosphate reductase beta chain [EC:1.17.	187		
HAV_01035	K03647	nrdI; protein involved in ribonucleotide reduction	34		
HAV_01036	K02687	prmA; ribosomal protein L11 methyltransferase [EC:2.1.1.-]	38		
HAV_01037	K08281	pncA; nicotinamidase/pyrazinamidase [EC:3.5.1.19 3.5.1.-]	112		
HAV_01038	K03116	tatA; sec-independent protein translocase protein TatA	19		
HAV_01039	K04042	glmU; bifunctional UDP-N-acetylglucosamine pyrophosphorylase / glucosamine-1-p	182		
HAV_01040					
HAV_01041			3	K00343	1
HAV_01042	K03470	rnhB; ribonuclease HII [EC:3.1.26.4]	86		
HAV_01043	K01129	dgt; dGTPase [EC:3.1.5.1]	155		
HAV_01044	K16264	czcD, zitB; cobalt-zinc-cadmium efflux system protein	87		
HAV_01045	K03596	lepA; GTP-binding protein LepA	433		
HAV_01046	K01835	pgm; phosphoglucomutase [EC:5.4.2.2]	227		
HAV_01047	K01491	folD; methylenetetrahydrofolate dehydrogenase (NADP+) / methenyltetrahydrofol	118		
HAV_01048	K02221	yggT; YggT family protein	14	K02031	1
HAV_01049	K00942	gmk, GUK1; guanylate kinase [EC:2.7.4.8]	84		
HAV_01050	K02528	ksgA; 16S rRNA (adenine1518-N6/adenine1519-N6)-dimethyltransferase [EC:2.1.1.	82		
HAV_01051	K00097	pdxA; 4-hydroxythreonine-4-phosphate dehydrogenase [EC:1.1.1.262]	105		
HAV_01052	K03771	surA; peptidyl-prolyl cis-trans isomerase SurA [EC:5.2.1.8]	23		
HAV_01053	K04744	lptD, imp, ostA; LPS-assembly protein	51		
HAV_01054	K11720	lptG; lipopolysaccharide export system permease protein	57		
HAV_01055			178		

HAV_01056	K07566	tsaC, rimN, SUA5; L-threonylcarbamoyladenylate synthase [EC:2.7.7.87]	109		
HAV_01057	K01082	cysQ, MET22, BPNT1; 3'(2'), 5'-bisphosphate nucleotidase [EC:3.1.3.7]	71		
HAV_01058	K01255	CARP, pepA; leucyl aminopeptidase [EC:3.4.11.1]	246		
HAV_01059	K02339	holC; DNA polymerase III subunit chi [EC:2.7.7.7]	43		
HAV_01060			4	K00919	1
HAV_01061			41		
HAV_01062					
HAV_01063			11	K06338	2
HAV_01064			32	K19427	2
HAV_01065	K13581	ccrM; modification methylase [EC:2.1.1.72]	178		
HAV_01066			147		
HAV_01067			21	K19591	1
HAV_01068	K09859	K09859; uncharacterized protein	37		
HAV_01069	K07337	K07337; penicillin-binding protein activator	40		
HAV_01070			4	K01524	1
HAV_01071			3	K23743	1
HAV_01072			2	K23743	1
HAV_01073	K06905	K06905; uncharacterized protein	20		
HAV_01074			4	K02443	1
HAV_01075					
HAV_01076	K06903	K06903; uncharacterized protein	20		
HAV_01077			1	K02313	1
HAV_01078			11	K01875	1
HAV_01079	K06908	K06908; uncharacterized protein	54		
HAV_01080	K06907	K06907; uncharacterized protein	103		
HAV_01081			8	K11904	1
HAV_01082			38		
HAV_01083			70		
HAV_01084					
HAV_01085			4	K00382	1
HAV_01086			3	K00627	1
HAV_01087			22	K03279	2
HAV_01089	K24845	ubiV; O2-independent ubiquinone biosynthesis protein UbiV	155		
HAV_01090	K24844	ubiU; O2-independent ubiquinone biosynthesis protein UbiU	186		
HAV_01091	K24843	ubiT; O2-independent ubiquinone biosynthesis accessory factor UbiT	18	K24843/K24844	1
HAV_01092					
HAV_01094			2	K02030	1
HAV_01095	K02914	RP-L34, MRPL34, rpmH; large subunit ribosomal protein L34	35		
HAV_01096	K03536	rnpA; ribonuclease P protein component [EC:3.1.26.5]	23		
HAV_01097	K08998	K08998; uncharacterized protein	43		
HAV_01098	K03217	yidC, spoIIIJ, OXA1, ccfA; YidC/Oxa1 family membrane protein insertase	223		
HAV_01099	K03978	engB; GTP-binding protein	84		
HAV_01100	K03299	TC.GNTP; gluconate:H+ symporter, GntP family	167		
HAV_01101			54		
HAV_01102	K13292	lgt, umpA; phosphatidylglycerol---prolipoprotein diacylglyceryl transferase [EC:2.5.	145		
HAV_01103	K18164	NDUFAF7; NADH dehydrogenase [ubiquinone] 1 alpha subcomplex assembly facto	74		
HAV_01104	K06942	ychF; ribosome-binding ATPase	228		
HAV_01105	K01056	PTH1, pth, spoVC; peptidyl-tRNA hydrolase, PTH1 family [EC:3.1.1.29]	85		
HAV_01106	K02897	RP-L25, rplY; large subunit ribosomal protein L25	75		
HAV_01107	K00948	PRPS, prsA; ribose-phosphate pyrophosphokinase [EC:2.7.6.1]	189		
HAV_01108	K05810	yfiH; polyphenol oxidase [EC:1.10.3.-]	126		
HAV_01109	K07638	envZ; two-component system, OmpR family, osmolarity sensor histidine kinase Env	94		
HAV_01110	K07659	ompR; two-component system, OmpR family, phosphate regulon response regulat	71	K02483	5
HAV_01111			106		
HAV_01112	K08744	CRLS; cardiolipin synthase (CMP-forming) [EC:2.7.8.41]	71	K00995	5
HAV_01113	K03703	uvrC; excinuclease ABC subunit C	220		
HAV_01114	K08311	nudH; putative (di)nucleoside polyphosphate hydrolase [EC:3.6.1.-]	86		
HAV_01115	K03797	E3.4.21.102, prc, ctpA; carboxyl-terminal processing protease [EC:3.4.21.102]	97		
HAV_01116	K22719	envC; murein hydrolase activator	15		
HAV_01117	K15633	gpmI; 2,3-bisphosphoglycerate-independent phosphoglycerate mutase [EC:5.4.2.1	250		
HAV_01118	K00783	rlmH; 23S rRNA (pseudouridine1915-N3)-methyltransferase [EC:2.1.1.177]	45		
HAV_01119	K09710	ybeB; ribosome-associated protein	37		
HAV_01120	K00969	nadD; nicotinate-nucleotide adenylyltransferase [EC:2.7.7.18]	62		
HAV_01121					
HAV_01122	K03979	obgE, cgtA, MTG2; GTPase [EC:3.6.5.-]	145		
HAV_01123	K02899	RP-L27, MRPL27, rpmA; large subunit ribosomal protein L27	71		
HAV_01124	K02888	RP-L21, MRPL21, rplU; large subunit ribosomal protein L21	54		
HAV_01125	K01295	cpg; glutamate carboxypeptidase [EC:3.4.17.11]	128		

HAV_01126			15	K08973	1
HAV_01127			10	K03597	1
HAV_01128			7	K03074	1
HAV_01129					
HAV_01132	K00800	aroA; 3-phosphoshikimate 1-carboxyvinyltransferase [EC:2.5.1.19]	135	K24018	10
HAV_01133	K00945	cmk; CMP/dCMP kinase [EC:2.7.4.25]	71		
HAV_01134	K02945	RP-S1, rpsA; small subunit ribosomal protein S1	255		
HAV_01135	K01963	accD; acetyl-CoA carboxylase carboxyl transferase subunit beta [EC:6.4.1.2 2.1.3.15	150		
HAV_01136	K11754	folC; dihydrofolate synthase / folylpolyglutamate synthase [EC:6.3.2.12 6.3.2.17]	123		
HAV_01137	K03671	trxA; thioredoxin 1	53		
HAV_01138	K16898	addA; ATP-dependent helicase/nuclease subunit A [EC:5.6.2.4 3.1.-.-]	257		
HAV_01139	K16899	addB; ATP-dependent helicase/nuclease subunit B [EC:5.6.2.4 3.1.-.-]	161		
HAV_01140	K06925	tsaE; tRNA threonylcarbamoyladenosine biosynthesis protein TsaE	31	K07102	1
HAV_01141	K00384	trxB, TRR; thioredoxin reductase (NADPH) [EC:1.8.1.9]	157		
HAV_01142	K03498	trkH, trkG, ktrB, ktrD; trk/ktr system potassium uptake protein	218		
HAV_01143					
HAV_01144			1	K21449	1
HAV_01145			2	K01255	1
HAV_01146					
HAV_01147			7	K03798	1
HAV_01149	K03322	mntH; manganese transport protein	279		
HAV_01150			19	K01356	3
HAV_01151			3	K18555	1
HAV_01152			11	K01447	1
HAV_01153			22	K02081	17
HAV_01154	K01424	E3.5.1.1, ansA, ansB; L-asparaginase [EC:3.5.1.1]	88		
HAV_01155	K03218	rlmB; 23S rRNA (guanosine2251-2'-O)-methyltransferase [EC:2.1.1.185]	74		
HAV_01158	K02358	tuf, TUFM; elongation factor Tu	329		
HAV_01160	K02601	nusG; transcription termination/antitermination protein NusG	76		
HAV_01161	K02867	RP-L11, MRPL11, rplK; large subunit ribosomal protein L11	93		
HAV_01162	K02863	RP-L1, MRPL1, rplA; large subunit ribosomal protein L1	172		
HAV_01163	K02864	RP-L10, MRPL10, rplJ; large subunit ribosomal protein L10	67		
HAV_01164	K02935	RP-L7, MRPL12, rplL; large subunit ribosomal protein L7/L12	79		
HAV_01165	K03043	rpoB; DNA-directed RNA polymerase subunit beta [EC:2.7.7.6]	785		
HAV_01166	K03046	rpoC; DNA-directed RNA polymerase subunit beta' [EC:2.7.7.6]	753	K13797	43
HAV_01167	K02950	RP-S12, MRPS12, rpsL; small subunit ribosomal protein S12	141		
HAV_01168	K02992	RP-S7, MRPS7, rpsG; small subunit ribosomal protein S7	116		
HAV_01169	K02355	fusA, GFM, EFG; elongation factor G	521		
HAV_01170	K02358	tuf, TUFM; elongation factor Tu	329		
HAV_01171	K02946	RP-S10, MRPS10, rpsJ; small subunit ribosomal protein S10	112		
HAV_01172	K02906	RP-L3, MRPL3, rplC; large subunit ribosomal protein L3	175		
HAV_01173	K02926	RP-L4, MRPL4, rplD; large subunit ribosomal protein L4	106		
HAV_01174	K02892	RP-L23, MRPL23, rplW; large subunit ribosomal protein L23	48	K02906	1
HAV_01175	K02886	RP-L2, MRPL2, rplB; large subunit ribosomal protein L2	190		
HAV_01176	K02965	RP-S19, rpsS; small subunit ribosomal protein S19	94		
HAV_01177	K02890	RP-L22, MRPL22, rplV; large subunit ribosomal protein L22	62		
HAV_01178	K02982	RP-S3, rpsC; small subunit ribosomal protein S3	143		
HAV_01179	K02878	RP-L16, MRPL16, rplP; large subunit ribosomal protein L16	148		
HAV_01180	K02904	RP-L29, rpmC; large subunit ribosomal protein L29	32		
HAV_01181	K02961	RP-S17, MRPS17, rpsQ; small subunit ribosomal protein S17	42		
HAV_01182	K02874	RP-L14, MRPL14, rplN; large subunit ribosomal protein L14	98		
HAV_01183	K02895	RP-L24, MRPL24, rplX; large subunit ribosomal protein L24	56		
HAV_01184	K02931	RP-L5, MRPL5, rplE; large subunit ribosomal protein L5	118		
HAV_01185	K02954	RP-S14, MRPS14, rpsN; small subunit ribosomal protein S14	62		
HAV_01186	K02994	RP-S8, rpsH; small subunit ribosomal protein S8	66		
HAV_01187	K02933	RP-L6, MRPL6, rplF; large subunit ribosomal protein L6	137		
HAV_01188	K02881	RP-L18, MRPL18, rplR; large subunit ribosomal protein L18	57		
HAV_01189	K02988	RP-S5, MRPS5, rpsE; small subunit ribosomal protein S5	114		
HAV_01190	K02907	RP-L30, MRPL30, rpmD; large subunit ribosomal protein L30	58		
HAV_01191	K02876	RP-L15, MRPL15, rplO; large subunit ribosomal protein L15	81		
HAV_01192	K03076	secY; preprotein translocase subunit SecY	301		
HAV_01193	K00939	adk, AK; adenylate kinase [EC:2.7.4.3]	88		
HAV_01194	K02952	RP-S13, rpsM; small subunit ribosomal protein S13	89		
HAV_01195	K02948	RP-S11, MRPS11, rpsK; small subunit ribosomal protein S11	91		
HAV_01196	K03040	rpoA; DNA-directed RNA polymerase subunit alpha [EC:2.7.7.6]	229		
HAV_01197	K02879	RP-L17, MRPL17, rplQ; large subunit ribosomal protein L17	76		
HAV_01198			2		
HAV_01199			85	K04771	5

HAV_01200			2	K02014	1
HAV_01201	K06179	rluC; 23S rRNA pseudouridine955/2504/2580 synthase [EC:5.4.99.24]	79		
HAV_01202	K06190	ispZ; intracellular septation protein	88		
HAV_01203	K03801	lipB; lipoyl(octanoyl) transferase [EC:2.3.1.181]	79		
HAV_01205	K06213	mgtE; magnesium transporter	118		
HAV_01206	K07091	lptF; lipopolysaccharide export system permease protein	34		
HAV_01207	K00858	ppnK, NADK; NAD+ kinase [EC:2.7.1.23]	81		
HAV_01208	K13588	chpT; histidine phosphotransferase ChpT	41		
HAV_01209	K13584	ctrA; two-component system, cell cycle response regulator CtrA	105		
HAV_01210	K01611	speD, AMD1; S-adenosylmethionine decarboxylase [EC:4.1.1.50]	83		
HAV_01211	K00797	speE, SRM, SPE3; spermidine synthase [EC:2.5.1.16]	133		
HAV_01212	K13587	cckA; two-component system, cell cycle sensor histidine kinase and response regul	87		
HAV_01213	K03553	recA; recombination protein RecA	214		
HAV_01214	K01872	AARS, alaS; alanyl-tRNA synthetase [EC:6.1.1.7]	360		
HAV_01215			4	K03768	2
HAV_01216	K00873	PK, pyk; pyruvate kinase [EC:2.7.1.40]	237		
HAV_01217	K03585	acrA, mexA, adeI, smeD, mtrC, cmeA; membrane fusion protein, multidrug efflux sy	23		
HAV_01218			272	K03296	72
HAV_01219	K12340	tolC, bepC, cyaE, raxC, sapF, rsaF, hasF; outer membrane protein	63		
HAV_01221	K02770	fruA; fructose PTS system EIIBC or EIIC component [EC:2.7.1.202]	217		
HAV_01222	K03702	uvrB; excinuclease ABC subunit B	339		
HAV_01223	K01095	pgpA; phosphatidylglycerophosphatase A [EC:3.1.3.27]	37		
HAV_01224	K03624	greA; transcription elongation factor GreA	90		
HAV_01225	K09117	K09117; uncharacterized protein	60		
HAV_01226	K02316	dnaG; DNA primase [EC:2.7.7.101]	123		
HAV_01227	K03086	rpoD; RNA polymerase primary sigma factor	183		
HAV_01229			8	K18678	4
HAV_01230	K11041	eta; exfoliative toxin A/B	99		
HAV_01231	K03760	eptA, pmrC; lipid A ethanolaminephosphotransferase [EC:2.7.8.43]	88		
HAV_01232	K03760	eptA, pmrC; lipid A ethanolaminephosphotransferase [EC:2.7.8.43]	77		
HAV_01233			104		
HAV_01234			2	K23743	2
HAV_01235			2	K23743	2

Query	KO	Definition	Score	Second best	Score
HPDP_00001			1	K03695	1
HPDP_00002					
HPDP_00003					
HPDP_00004			2	K23743	1
HPDP_00005			3	K23743	1
HPDP_00006			250		
HPDP_00007	K01652	E2.2.1.6L, ilvB, ilvG, ilvI; acetolactate synthase I/II/III large subunit [EC:2.2.1.6]	316		
HPDP_00008	K01647	CS, gltA; citrate synthase [EC:2.3.3.1]	217		
HPDP_00009	K00031	IDH1, IDH2, icd; isocitrate dehydrogenase [EC:1.1.1.42]	247		
HPDP_00010	K01681	ACO, acnA; aconitate hydratase [EC:4.2.1.3]	760		
HPDP_00011	K00024	mdh; malate dehydrogenase [EC:1.1.1.37]	195		
HPDP_00012	K13821	putA; RHH-type transcriptional regulator, proline utilization regulon repressor / prol	395		
HPDP_00013	K11928	putP; sodium/proline symporter	151	K03307	17
HPDP_00014	K03325	ACR3, arsB; arsenite transporter	256		
HPDP_00015	K14155	patB, malY; cysteine-S-conjugate beta-lyase [EC:4.4.1.13]	144		
HPDP_00016	K01489	cdd, CDA; cytidine deaminase [EC:3.5.4.5]	79		
HPDP_00017	K00758	deoA, TYMP; thymidine phosphorylase [EC:2.4.2.4]	218		
HPDP_00018	K01619	deoC, DERA; deoxyribose-phosphate aldolase [EC:4.1.2.4]	97		
HPDP_00019	K03317	TC.CNT; concentrative nucleoside transporter, CNT family	212		
HPDP_00022			1	K07258	1
HPDP_00023			3		
HPDP_00024					
HPDP_00025			4	K03286	3
HPDP_00026	K06207	typA, bipA; GTP-binding protein	373		
HPDP_00027			33	K15270	3
HPDP_00028			34	K15270	6
HPDP_00029			40	K15270	2
HPDP_00030	K06867	K06867; uncharacterized protein	10		
HPDP_00031	K09815	znuA; zinc transport system substrate-binding proteir	10	K02077	1
HPDP_00032	K09815	znuA; zinc transport system substrate-binding proteir	52		
HPDP_00033	K03089	rpoH; RNA polymerase sigma-32 factor	114		
HPDP_00036			18	K07678	1
HPDP_00037	K03923	mdaB; NADPH dehydrogenase (quinone) [EC:1.6.5.10]	124		
HPDP_00038					
HPDP_00039			2	K01686	1
HPDP_00040			9	K12996	1
HPDP_00041			122		
HPDP_00042	K17686	copA, ctpA, ATP7; P-type Cu+ transporter [EC:7.2.2.8]	292		
HPDP_00044	K00022	HADH; 3-hydroxyacyl-CoA dehydrogenase [EC:1.1.1.35]	93		
HPDP_00045	K02440	GLPF; glycerol uptake facilitator protein	131		
HPDP_00046	K00864	glpK, GK; glycerol kinase [EC:2.7.1.30]	322		
HPDP_00047	K00111	glpA, glpD; glycerol-3-phosphate dehydrogenase [EC:1.1.5.3]	217		
HPDP_00048	K03564	BCP, PRXQ, DOT5; thioredoxin-dependent peroxiredoxin [EC:1.11.1.24]	67		
HPDP_00049	K03564	BCP, PRXQ, DOT5; thioredoxin-dependent peroxiredoxin [EC:1.11.1.24]	56		
HPDP_00050			18	K09809	2
HPDP_00051	K06895	lysE, argO; L-lysine exporter family protein LysE/ArgC	144		
HPDP_00052	K07266	kpsC, lipA; capsular polysaccharide export protein	30		
HPDP_00053	K08344	scsB; suppressor for copper-sensitivity B	123		
HPDP_00054			101		
HPDP_00055	K22044	ybiO; moderate conductance mechanosensitive channe	41		
HPDP_00056	K00759	APRT, apt; adenine phosphoribosyltransferase [EC:2.4.2.7]	104		
HPDP_00057					
HPDP_00058	K02523	ispB; octaprenyl-diphosphate synthase [EC:2.5.1.90]	132		
HPDP_00059			52	K15460	14
HPDP_00060			112		
HPDP_00061	K02902	RP-L28, MRPL28, rpmB; large subunit ribosomal protein L28	68		
HPDP_00062					
HPDP_00063	K03699	tlyC; magnesium and cobalt exporter, CNNM family	138		
HPDP_00064	K01735	aroB; 3-dehydroquinate synthase [EC:4.2.3.4]	89	K13829	12
HPDP_00065	K00891	aroK, aroL; shikimate kinase [EC:2.7.1.71]	45		
HPDP_00066					
HPDP_00067	K04763	xerD; integrase/recombinase XerD	118		
HPDP_00068	K01962	accA; acetyl-CoA carboxylase carboxyl transferase subunit alpha [EC:6.4.1.2 2.1.3.15	173		
HPDP_00070	K07552	bcr, tcaB; MFS transporter, DHA1 family, multidrug resistance proteir	126		
HPDP_00071	K07334	higB-1; toxin HigB-1	79		
HPDP_00072	K21498	higA-1; antitoxin HigA-1	35		
HPDP_00073			1	K21572	1
HPDP_00074	K02257	COX10, ctaB, cyoE; heme o synthase [EC:2.5.1.141]	99		

HPDP_00075	K02297	cyoA; cytochrome o ubiquinol oxidase subunit II [EC:7.1.1.3]	123		
HPDP_00076	K02298	cyoB; cytochrome o ubiquinol oxidase subunit I [EC:7.1.1.3]	490		
HPDP_00077	K02299	cyoC; cytochrome o ubiquinol oxidase subunit III	118		
HPDP_00078	K02300	cyoD; cytochrome o ubiquinol oxidase subunit IV	35		
HPDP_00079	K03305	TC.POT; proton-dependent oligopeptide transporter, POT family	161		
HPDP_00080					
HPDP_00081	K19303	mepH; murein DD-endopeptidase [EC:3.4.-.-]	21		
HPDP_00082			28	K03187	1
HPDP_00083	K00571	E2.1.1.72; site-specific DNA-methyltransferase (adenine-specific) [EC:2.1.1.72]	47	K13581	30
HPDP_00084			58		
HPDP_00085			7		
HPDP_00086			1	K08363	1
HPDP_00087	K03545	tig; trigger factor	123		
HPDP_00088	K01358	clpP, CLPP; ATP-dependent Clp protease, protease subunit [EC:3.4.21.92]	176		
HPDP_00089	K03544	clpX, CLPX; ATP-dependent Clp protease ATP-binding subunit ClpX	200		
HPDP_00090	K01338	lon; ATP-dependent Lon protease [EC:3.4.21.53]	362		
HPDP_00091	K03530	hupB; DNA-binding protein HU-beta	41		
HPDP_00095	K01752	E4.3.1.17, sdaA, sdaB, tdcG; L-serine dehydratase [EC:4.3.1.17]	205		
HPDP_00096	K07240	chrA; chromate transporter	39		
HPDP_00097	K07240	chrA; chromate transporter	32		
HPDP_00098	K01207	nagZ; beta-N-acetylhexosaminidase [EC:3.2.1.52]	126		
HPDP_00099			71		
HPDP_00100	K05304	NANS, SAS; sialic acid synthase [EC:2.5.1.56 2.5.1.57 2.5.1.132]	165	K01654	32
HPDP_00101			9	K02030	1
HPDP_00102			1	K00566	1
HPDP_00103	K03778	ldhA; D-lactate dehydrogenase [EC:1.1.1.28]	170		
HPDP_00104	K01915	glnA, GLUL; glutamine synthetase [EC:6.3.1.2]	18		
HPDP_00105	K01961	accC; acetyl-CoA carboxylase, biotin carboxylase subunit [EC:6.4.1.2 6.3.4.14]	282		
HPDP_00106	K02160	accB, bccP; acetyl-CoA carboxylase biotin carboxyl carrier protein	40		
HPDP_00107	K03786	aroQ, qutE; 3-dehydroquinate dehydratase II [EC:4.2.1.10]	65		
HPDP_00108	K01581	E4.1.1.17, ODC1, speC, speF; ornithine decarboxylase [EC:4.1.1.17]	190		
HPDP_00109			37		
HPDP_00110	K03308	TC.NSS; neurotransmitter:Na+ symporter, NSS family	180		
HPDP_00111	K03308	TC.NSS; neurotransmitter:Na+ symporter, NSS family	134		
HPDP_00112			59		
HPDP_00113			5	K03286	3
HPDP_00114	K07462	recJ; single-stranded-DNA-specific exonuclease [EC:3.1.-.-]	172		
HPDP_00116					
HPDP_00117					
HPDP_00118			5	K07755	1
HPDP_00119	K00820	glmS, GFPT; glutamine---fructose-6-phosphate transaminase (isomerizing) [EC:2.6.1.	293		
HPDP_00120	K11746	kefF; glutathione-regulated potassium-efflux system ancillary protein KefF	21		
HPDP_00121			3	K10680	1
HPDP_00122	K06199	crcB, FEX; fluoride exporter	36		
HPDP_00123			101		
HPDP_00124					
HPDP_00125	K03595	era, ERAL1; GTPase	109		
HPDP_00126	K03584	recO; DNA repair protein RecO (recombination protein O)	101		
HPDP_00127	K00573	E2.1.1.77, pcm; protein-L-isoaspartate(D-aspartate) O-methyltransferase [EC:2.1.1.7	37		
HPDP_00128					
HPDP_00129	K01873	VARS, valS; valyl-tRNA synthetase [EC:6.1.1.9]	414		
HPDP_00130	K00330	nuoA; NADH-quinone oxidoreductase subunit A [EC:7.1.1.2]	72		
HPDP_00131	K00331	nuoB; NADH-quinone oxidoreductase subunit B [EC:7.1.1.2]	163		
HPDP_00132	K00332	nuoC; NADH-quinone oxidoreductase subunit C [EC:7.1.1.2]	65		
HPDP_00133	K00333	nuoD; NADH-quinone oxidoreductase subunit D [EC:7.1.1.2]	296		
HPDP_00134	K00334	nuoE; NADH-quinone oxidoreductase subunit E [EC:7.1.1.2]	46	K03943	7
HPDP_00135	K00335	nuoF; NADH-quinone oxidoreductase subunit F [EC:7.1.1.2]	244		
HPDP_00136	K00336	nuoG; NADH-quinone oxidoreductase subunit G [EC:7.1.1.2]	298		
HPDP_00137	K00337	nuoH; NADH-quinone oxidoreductase subunit H [EC:7.1.1.2]	217		
HPDP_00138	K00338	nuoI; NADH-quinone oxidoreductase subunit I [EC:7.1.1.2]	101		
HPDP_00139	K00339	nuoJ; NADH-quinone oxidoreductase subunit J [EC:7.1.1.2]	76		
HPDP_00140	K00340	nuoK; NADH-quinone oxidoreductase subunit K [EC:7.1.1.2]	52		
HPDP_00141	K00341	nuoL; NADH-quinone oxidoreductase subunit L [EC:7.1.1.2]	228		
HPDP_00142	K00342	nuoM; NADH-quinone oxidoreductase subunit M [EC:7.1.1.2]	164		
HPDP_00143	K00343	nuoN; NADH-quinone oxidoreductase subunit N [EC:7.1.1.2]	140		
HPDP_00144	K03524	birA; BirA family transcriptional regulator, biotin operon repressor / biotin---[acetyl-	45		
HPDP_00145	K03525	coaX; type III pantothenate kinase [EC:2.7.1.33]	36		
HPDP_00146	K12574	rnj; ribonuclease J [EC:3.1.-.-]	154		
HPDP_00147			45	K09809	1

HPDP_00148	K01791	wecB; UDP-N-acetylglucosamine 2-epimerase (non-hydrolysing) [EC:5.1.3.14]	102		
HPDP_00149			9	K06889	1
HPDP_00150	K25227	sdsA1; linear primary-alkylsulfatase [EC:3.1.6.21]	310		
HPDP_00151	K06901	pbuG, azgA, ghxP, ghxQ, adeQ; adenine/guanine/hypoxanthine permease	157		
HPDP_00152	K00459	ncd2, npd; nitronate monooxygenase [EC:1.13.12.16]	89		
HPDP_00153	K01881	PARS, proS; prolyl-tRNA synthetase [EC:6.1.1.15]	246		
HPDP_00154	K09808	lolC_E; lipoprotein-releasing system permease protein	169		
HPDP_00155	K09810	lolD; lipoprotein-releasing system ATP-binding protein [EC:7.6.2.-]	98		
HPDP_00156	K02337	dnaE; DNA polymerase III subunit alpha [EC:2.7.7.7]	385		
HPDP_00157	K02232	cobQ, cbiP; adenosylcobyric acid synthase [EC:6.3.5.10]	172		
HPDP_00158			2	K09924	1
HPDP_00159	K06949	rsgA, engC; ribosome biogenesis GTPase / thiamine phosphate phosphatase [EC:3.6	103		
HPDP_00160			6		
HPDP_00161			18	K07006	2
HPDP_00162			10	K00937	1
HPDP_00163	K04564	SOD2; superoxide dismutase, Fe-Mn family [EC:1.15.1.1]	113		
HPDP_00164					
HPDP_00165			2		
HPDP_00166					
HPDP_00167	K03642	rlpA; rare lipoprotein A	76		
HPDP_00168	K07258	dacC, dacA, dacD; serine-type D-Ala-D-Ala carboxypeptidase (penicillin-binding prot	104		
HPDP_00169	K00943	tmk, DTYMK; dTMP kinase [EC:2.7.4.9]	26		
HPDP_00170	K02341	holB; DNA polymerase III subunit delta' [EC:2.7.7.7]	45		
HPDP_00171			11	K14654	4
HPDP_00172					
HPDP_00173			1	K09013	1
HPDP_00174	K00374	narI, narV; nitrate reductase gamma subunit [EC:1.7.5.1 1.7.99.-]	14		
HPDP_00175					
HPDP_00176	K01286	E3.4.16.4; D-alanyl-D-alanine carboxypeptidase [EC:3.4.16.4]	66	K07258	19
HPDP_00177	K00525	E1.17.4.1A, nrdA, nrdE; ribonucleoside-diphosphate reductase alpha chain [EC:1.17.	653		
HPDP_00178			26		
HPDP_00179	K02067	mlaD, linM; phospholipid/cholesterol/gamma-HCH transport system substrate-bind	14		
HPDP_00180	K02067	mlaD, linM; phospholipid/cholesterol/gamma-HCH transport system substrate-bind	45		
HPDP_00181			18		
HPDP_00182			1	K07447	1
HPDP_00183			92		
HPDP_00184	K03701	uvrA; excinuclease ABC subunit A	591		
HPDP_00185	K03111	ssb; single-strand DNA-binding protein	72		
HPDP_00186	K03270	kdsC; 3-deoxy-D-manno-octulosonate 8-phosphate phosphatase (KDO 8-P phosphat	55		
HPDP_00187	K03811	pnuC; nicotinamide mononucleotide transporter	30		
HPDP_00188	K03811	pnuC; nicotinamide mononucleotide transporter	28		
HPDP_00189	K02967	RP-S2, MRPS2, rpsB; small subunit ribosomal protein S2	132		
HPDP_00190	K02357	tsf, TSFM; elongation factor Ts	147		
HPDP_00191	K09903	pyrH; uridylate kinase [EC:2.7.4.22]	127		
HPDP_00192	K02838	frr, MRRF, RRF; ribosome recycling factor	87		
HPDP_00193	K00806	uppS; undecaprenyl diphosphate synthase [EC:2.5.1.31]	93		
HPDP_00194			7	K01356	1
HPDP_00195	K07724	ner, nlp, sfsB; Ner family transcriptional regulator	11		
HPDP_00196	K07497	K07497; putative transposase	85		
HPDP_00197			80		
HPDP_00198			3	K10254	1
HPDP_00199					
HPDP_00200					
HPDP_00201					
HPDP_00202					
HPDP_00203			8	K07482	1
HPDP_00204			39		
HPDP_00205			3	K04100	1
HPDP_00206			3	K13525	1
HPDP_00207			6	K03612	1
HPDP_00208			8	K08151	1
HPDP_00209			10	K11751	1
HPDP_00210			10	K20269	1
HPDP_00211			10	K01775	1
HPDP_00212	K06907	K06907; uncharacterized protein	141		
HPDP_00213	K06908	K06908; uncharacterized protein	59		
HPDP_00214			12	K01875	1
HPDP_00215					
HPDP_00216	K06903	K06903; uncharacterized protein	15		

HPDP_00217			2	K01803	1
HPDP_00218			5	K20074	1
HPDP_00219	K06905	K06905; uncharacterized protein	29		
HPDP_00220					
HPDP_00221					
HPDP_00222			8	K01921	1
HPDP_00223			2	K20971	1
HPDP_00224	K03664	smpB; SsrA-binding protein	59		
HPDP_00225	K00981	E2.7.7.41, CDS1, CDS2, cdsA; phosphatidate cytidylyltransferase [EC:2.7.7.41]	28		
HPDP_00226	K17837	bla2, blm, ccrA, blaB; metallo-beta-lactamase class B [EC:3.5.2.6]	56	K18782	11
HPDP_00227			223		
HPDP_00228			94		
HPDP_00229			31	K22847	6
HPDP_00230			11	K06992	1
HPDP_00231	K01921	ddl; D-alanine-D-alanine ligase [EC:6.3.2.4]	46	K00075/K01921	1
HPDP_00232	K03303	lctP; lactate permease	197		
HPDP_00233	K01095	pgpA; phosphatidylglycerophosphatase A [EC:3.1.3.27]	15		
HPDP_00234			2	K02014	1
HPDP_00235	K03060	rpoZ; DNA-directed RNA polymerase subunit omega [EC:2.7.7.6]	21		
HPDP_00236	K01139	spoT; GTP diphosphokinase / guanosine-3',5'-bis(diphosphate) 3'-diphosphatase [EC	207		
HPDP_00237	K03474	pdxJ; pyridoxine 5-phosphate synthase [EC:2.6.99.2]	115		
HPDP_00238	K00997	acpS; holo-[acyl-carrier protein] synthase [EC:2.7.8.7]	39	K17758	1
HPDP_00239	K03100	lepB; signal peptidase I [EC:3.4.21.89]	87		
HPDP_00240	K03685	rnc, DROSHA, RNT1; ribonuclease III [EC:3.1.26.3]	62	K03595	2
HPDP_00241			19	K21687	1
HPDP_00242	K01624	FBA, fbaA; fructose-bisphosphate aldolase, class II [EC:4.1.2.13]	208		
HPDP_00243			28	K02199	3
HPDP_00244	K01478	arcA; arginine deiminase [EC:3.5.3.6]	164		
HPDP_00245	K00611	OTC, argF, argI; ornithine carbamoyltransferase [EC:2.1.3.3]	277		
HPDP_00246			202		
HPDP_00247	K00926	arcC; carbamate kinase [EC:2.7.2.2]	221		
HPDP_00248			3	K24967	1
HPDP_00249					
HPDP_00250			1	K17723	1
HPDP_00251			10	K19334	7
HPDP_00252	K03655	recG; ATP-dependent DNA helicase RecG [EC:5.6.2.4]	228		
HPDP_00253	K03723	mfd; transcription-repair coupling factor (superfamily II helicase) [EC:3.6.4.-	374		
HPDP_00254			102		
HPDP_00255					
HPDP_00256	K01476	E3.5.3.1, rocF, arg; arginase [EC:3.5.3.1]	102		
HPDP_00257					
HPDP_00258	K16092	btuB; vitamin B12 transporter	66		
HPDP_00260			21	K03327	2
HPDP_00261	K02073	metQ; D-methionine transport system substrate-binding proteir	118		
HPDP_00262	K02073	metQ; D-methionine transport system substrate-binding proteir	115		
HPDP_00263	K02072	metI; D-methionine transport system permease proteir	114		
HPDP_00264	K02071	metN; D-methionine transport system ATP-binding protein	146		
HPDP_00265	K00615	E2.2.1.1, tktA, tktB; transketolase [EC:2.2.1.1]	269		
HPDP_00266	K01783	rpe, RPE; ribulose-phosphate 3-epimerase [EC:5.1.3.1]	113		
HPDP_00267			75		
HPDP_00268			2	K03286	1
HPDP_00269			2	K24846	1
HPDP_00270	K19286	nfrA2; FMN reductase [NAD(P)H] [EC:1.5.1.39]	77		
HPDP_00271			22	K02838	1
HPDP_00272	K02804	nagE; N-acetylglucosamine PTS system EIICBA or EIICB component [EC:2.7.1.193]	200		
HPDP_00273			2	K03406	1
HPDP_00274	K03652	MPG; DNA-3-methyladenine glycosylase [EC:3.2.2.21]	16		
HPDP_00275			15	K07645	1
HPDP_00276	K03308	TC.NSS; neurotransmitter:Na+ symporter, NSS family	311		
HPDP_00277	K00259	ald; alanine dehydrogenase [EC:1.4.1.1]	196		
HPDP_00278	K03770	ppiD; peptidyl-prolyl cis-trans isomerase D [EC:5.2.1.8]	47		
HPDP_00279	K01803	TPI, tpiA; triosephosphate isomerase (TIM) [EC:5.3.1.1]	123		
HPDP_00280	K03075	secG; preprotein translocase subunit SecG	14	K21687	1
HPDP_00281	K01937	pyrG, CTPS; CTP synthase [EC:6.3.4.2]	319		
HPDP_00282	K01627	kdsA; 2-dehydro-3-deoxyphosphooctonate aldolase (KDO 8-P synthase) [EC:2.5.1.55	186		
HPDP_00283	K01689	ENO, eno; enolase [EC:4.2.1.11]	294		
HPDP_00284			1	K02346	1
HPDP_00285			5		
HPDP_00286					

HPDP_00287			2	K03686	1
HPDP_00288			9	K18918	2
HPDP_00289	K19092	parE1_3_4; toxin ParE1/3/4	21		
HPDP_00290					
HPDP_00291	K02445	glpT; MFS transporter, OPA family, glycerol-3-phosphate transporter	234		
HPDP_00292			7	K03586	1
HPDP_00293	K00161	PDHA, pdhA; pyruvate dehydrogenase E1 component alpha subunit [EC:1.2.4.1]	160		
HPDP_00294	K00627	DLAT, aceF, pdhC; pyruvate dehydrogenase E2 component (dihydrolipoamide acetyl	106		
HPDP_00295	K00382	DLD, lpd, pdhD; dihydrolipoamide dehydrogenase [EC:1.8.1.4]	298		
HPDP_00296					
HPDP_00297			2	K01814	1
HPDP_00298			4	K06919	1
HPDP_00299	K01750	E4.3.1.12, ocd; ornithine cyclodeaminase [EC:4.3.1.12]	161		
HPDP_00300	K07504	K07504; predicted type IV restriction endonuclease	164		
HPDP_00301			31	K06867	1
HPDP_00302			5	K00341	1
HPDP_00303			6	K02035	1
HPDP_00304			11	K00799	4
HPDP_00305	K01679	E4.2.1.2B, fumC, FH; fumarate hydratase, class II [EC:4.2.1.2]	259		
HPDP_00306	K14445	SLC13A2_3_5; solute carrier family 13 (sodium-dependent dicarboxylate transporte	156		
HPDP_00307	K03644	lipA, LIAS, LIP1, LIP5; lipoyl synthase [EC:2.8.1.8]	149		
HPDP_00308	K18588	COQ10; coenzyme Q-binding protein COQ10	49		
HPDP_00309			34		
HPDP_00310	K01874	MARS, metG; methionyl-tRNA synthetase [EC:6.1.1.10]	275		
HPDP_00311					
HPDP_00312			5		
HPDP_00313					
HPDP_00314			2	K03406	1
HPDP_00315					
HPDP_00316			2	K16211	1
HPDP_00317	K03424	tatD; TatD DNase family protein [EC:3.1.21.-]	152		
HPDP_00318	K06167	phnP; phosphoribosyl 1,2-cyclic phosphate phosphodiesterase [EC:3.1.4.55]	87		
HPDP_00319			2	K06217	1
HPDP_00320			2	K10206	1
HPDP_00321			15	K00010	1
HPDP_00322	K03665	hflX; GTPase	89		
HPDP_00323	K03666	hfq; host factor-I protein	37		
HPDP_00324	K03499	trkA, ktrA, ktrC; trk/ktr system potassium uptake protein	77		
HPDP_00325	K09159	cptB; antitoxin CptB	22		
HPDP_00326			2	K07705	1
HPDP_00327					
HPDP_00328			1	K09808	1
HPDP_00329			4	K07497	1
HPDP_00330			2	K02338	1
HPDP_00331	K00240	sdhB, frdB; succinate dehydrogenase / fumarate reductase, iron-sulfur subunit [EC:1	181		
HPDP_00332	K00239	sdhA, frdA; succinate dehydrogenase / fumarate reductase, flavoprotein subunit [EC	408		
HPDP_00333	K00242	sdhD, frdD; succinate dehydrogenase / fumarate reductase, membrane anchor subu	26		
HPDP_00334	K00241	sdhC, frdC; succinate dehydrogenase / fumarate reductase, cytochrome b subunit	38		
HPDP_00335	K07791	dcuA; anaerobic C4-dicarboxylate transporter DcuA	232		
HPDP_00336	K07791	dcuA; anaerobic C4-dicarboxylate transporter DcuA	185		
HPDP_00337	K01744	aspA; aspartate ammonia-lyase [EC:4.3.1.1]	226		
HPDP_00338	K05540	dusB; tRNA-dihydrouridine synthase B [EC:1.-.-.-]	158		
HPDP_00339	K12506	ispDF; 2-C-methyl-D-erythritol 4-phosphate cytidylyltransferase / 2-C-methyl-D-eryt	139		
HPDP_00340	K00748	lpxB; lipid-A-disaccharide synthase [EC:2.4.1.182]	159		
HPDP_00341	K01759	GLO1, gloA; lactoylglutathione lyase [EC:4.4.1.5]	77		
HPDP_00342	K06867	K06867; uncharacterized protein	11		
HPDP_00343			80		
HPDP_00344	K01607	pcaC; 4-carboxymuconolactone decarboxylase [EC:4.1.1.44]	66		
HPDP_00345			21	K01928	1
HPDP_00346	K00963	UGP2, galU, galF; UTP--glucose-1-phosphate uridylyltransferase [EC:2.7.7.9]	139		
HPDP_00347	K01256	pepN; aminopeptidase N [EC:3.4.11.2]	320		
HPDP_00348			48		
HPDP_00349			1	K02020	1
HPDP_00350	K00088	IMPDH, guaB; IMP dehydrogenase [EC:1.1.1.205]	109		
HPDP_00351			1	K15580	1
HPDP_00352	K01756	purB, ADSL; adenylosuccinate lyase [EC:4.3.2.2]	241		
HPDP_00353	K01939	purA, ADSS; adenylosuccinate synthase [EC:6.3.4.4]	251		
HPDP_00354	K03186	ubiX, bsdB, PAD1; flavin prenyltransferase [EC:2.5.1.129]	93		
HPDP_00355					

HPDP_00356			3	K01756	1
HPDP_00357	K04094	trmFO, gid; methylenetetrahydrofolate--tRNA-(uracil-5-)-methyltransferase [EC:2.1.	212		
HPDP_00358	K00648	fabH; 3-oxoacyl-[acyl-carrier-protein] synthase III [EC:2.3.1.180]	182		
HPDP_00359	K03621	plsX; phosphate acyltransferase [EC:2.3.1.274]	149		
HPDP_00360	K02911	RP-L32, MRPL32, rpmF; large subunit ribosomal protein L32	48		
HPDP_00361			13	K01092	1
HPDP_00362			232		
HPDP_00363			220		
HPDP_00364			4	K13652	1
HPDP_00365			67	K01607	2
HPDP_00366			10	K00831	1
HPDP_00367			11	K06186	5
HPDP_00368	K00946	thiL; thiamine-monophosphate kinase [EC:2.7.4.16]	81		
HPDP_00369	K00600	glyA, SHMT; glycine hydroxymethyltransferase [EC:2.1.2.1]	243		
HPDP_00370	K01808	rpiB; ribose 5-phosphate isomerase B [EC:5.3.1.6]	59		
HPDP_00371	K00761	upp, UPRT; uracil phosphoribosyltransferase [EC:2.4.2.9]	83		
HPDP_00372	K02824	uraA, pyrP; uracil permease	212		
HPDP_00373			37		
HPDP_00374			2		
HPDP_00375	K09949	lpxI; UDP-2,3-diacylglucosamine hydrolase [EC:3.6.1.54]	89		
HPDP_00376	K00677	lpxA; UDP-N-acetylglucosamine acyltransferase [EC:2.3.1.129]	127		
HPDP_00377	K02372	fabZ; 3-hydroxyacyl-[acyl-carrier-protein] dehydratase [EC:4.2.1.59]	57	K16363	5
HPDP_00378	K02536	lpxD; UDP-3-O-[3-hydroxymyristoyl] glucosamine N-acyltransferase [EC:2.3.1.191]	115		
HPDP_00379	K06142	hlpA, ompH; outer membrane protein	14		
HPDP_00380	K01809	manA, MPI; mannose-6-phosphate isomerase [EC:5.3.1.8]	58		
HPDP_00381	K07277	SAM50, TOB55, bamA; outer membrane protein insertion porin family	211		
HPDP_00382	K11749	rseP; regulator of sigma E protease [EC:3.4.24.-]	111		
HPDP_00383	K00099	dxr; 1-deoxy-D-xylulose-5-phosphate reductoisomerase [EC:1.1.1.267]	132		
HPDP_00384			10	K07251	4
HPDP_00385	K03811	pnuC; nicotinamide mononucleotide transporter	45		
HPDP_00386	K01885	EARS, gltX; glutamyl-tRNA synthetase [EC:6.1.1.17]	234		
HPDP_00387			55		
HPDP_00388			3	K03088	1
HPDP_00389	K03118	tatC; sec-independent protein translocase protein TatC	81		
HPDP_00390			3	K01286	1
HPDP_00391	K01887	RARS, argS; arginyl-tRNA synthetase [EC:6.1.1.19]	254		
HPDP_00392	K15724	erpA; iron-sulfur cluster insertion protein	29		
HPDP_00393	K01142	E3.1.11.2, xthA; exodeoxyribonuclease III [EC:3.1.11.2]	114		
HPDP_00394					
HPDP_00396					
HPDP_00397					
HPDP_00398	K01662	dxs; 1-deoxy-D-xylulose-5-phosphate synthase [EC:2.2.1.7]	238		
HPDP_00399	K13789	GGPS; geranylgeranyl diphosphate synthase, type II [EC:2.5.1.1 2.5.1.10 2.5.1.29]	36	K00795	22
HPDP_00400	K03602	xseB; exodeoxyribonuclease VII small subunit [EC:3.1.11.6]	20		
HPDP_00401	K01876	DARS2, aspS; aspartyl-tRNA synthetase [EC:6.1.1.12]	372		
HPDP_00402			15		
HPDP_00403			6	K02020	1
HPDP_00404			2		
HPDP_00405			2	K06286	1
HPDP_00407			19		
HPDP_00408			8	K01552	1
HPDP_00409			10	K00544	1
HPDP_00411			15	K03972	3
HPDP_00412			30	K03671	1
HPDP_00413					
HPDP_00414			209		
HPDP_00415			3	K00525	1
HPDP_00416			2	K00656	1
HPDP_00417			17	K01130	1
HPDP_00418			14	K00703	1
HPDP_00419			1	K00762	1
HPDP_00420			20	K13924	1
HPDP_00421			2	K03436	1
HPDP_00422			3	K07459	1
HPDP_00423			106		
HPDP_00424			28	K06909	3
HPDP_00425					
HPDP_00426					
HPDP_00427			2	K01560	1

HPDP_00428			24	K02200	1
HPDP_00429			7	K03719	1
HPDP_00430	K00558	DNMT1, dcm; DNA (cytosine-5)-methyltransferase 1 [EC:2.1.1.37]	86		
HPDP_00431	K05569	mnhE, mrpE; multicomponent Na+:H+ antiporter subunit E	32		
HPDP_00432	K05570	mnhF, mrpF; multicomponent Na+:H+ antiporter subunit F	13		
HPDP_00433	K05571	mnhG, mrpG; multicomponent Na+:H+ antiporter subunit G	14	K05564	1
HPDP_00434	K05566	mnhB, mrpB; multicomponent Na+:H+ antiporter subunit B	71		
HPDP_00435	K05567	mnhC, mrpC; multicomponent Na+:H+ antiporter subunit C	33		
HPDP_00436	K05568	mnhD, mrpD; multicomponent Na+:H+ antiporter subunit D	108		
HPDP_00437	K05568	mnhD, mrpD; multicomponent Na+:H+ antiporter subunit D	215		
HPDP_00438	K05568	mnhD, mrpD; multicomponent Na+:H+ antiporter subunit D	61		
HPDP_00439			36		
HPDP_00440					
HPDP_00441			1	K11959	1
HPDP_00442					
HPDP_00443			4	K03317	1
HPDP_00444			9	K08978	4
HPDP_00445					
HPDP_00446	K01610	E4.1.1.49, pckA; phosphoenolpyruvate carboxykinase (ATP) [EC:4.1.1.49]	272		
HPDP_00447			1	K15532	1
HPDP_00448			10	K07192	1
HPDP_00449	K04047	dps; starvation-inducible DNA-binding protein	62		
HPDP_00450					
HPDP_00451					
HPDP_00452	K00009	mtlD; mannitol-1-phosphate 5-dehydrogenase [EC:1.1.1.17]	158		
HPDP_00453	K02800	mtlA, cmtA; mannitol PTS system EIICBA or EIICB component [EC:2.7.1.197]	348		
HPDP_00454			2	K10040	1
HPDP_00455			1	K03679	1
HPDP_00456			4	K07740	2
HPDP_00457			2	K01953	1
HPDP_00458			2	K00927	1
HPDP_00459	K04095	fic; cell filamentation protein	60		
HPDP_00460			6	K05606	4
HPDP_00461	K03837	sdaC; serine transporter	187		
HPDP_00462					
HPDP_00463					
HPDP_00464	K07552	bcr, tcaB; MFS transporter, DHA1 family, multidrug resistance protein	132		
HPDP_00465	K03788	aphA; acid phosphatase (class B) [EC:3.1.3.2]	55		
HPDP_00466			3		
HPDP_00468			2		
HPDP_00469	K00954	E2.7.7.3A, coaD, kdtB; pantetheine-phosphate adenylyltransferase [EC:2.7.7.3]	73		
HPDP_00470	K02469	gyrA; DNA gyrase subunit A [EC:5.6.2.2]	464		
HPDP_00471	K07276	K07276; uncharacterized protein	42		
HPDP_00472	K03527	ispH, lytB; 4-hydroxy-3-methylbut-2-en-1-yl diphosphate reductase [EC:1.17.7.4]	154		
HPDP_00473	K07323	mlaC; phospholipid transport system substrate-binding protein	10	K01695	1
HPDP_00474	K00970	pcnB; poly(A) polymerase [EC:2.7.7.19]	94		
HPDP_00475	K07082	K07082; UPF0755 protein	88		
HPDP_00476	K09458	fabF, OXSM, CEM1; 3-oxoacyl-[acyl-carrier-protein] synthase II [EC:2.3.1.179]	233		
HPDP_00477	K02078	acpP; acyl carrier protein	46		
HPDP_00478	K00059	fabG, OAR1; 3-oxoacyl-[acyl-carrier protein] reductase [EC:1.1.1.100]	138		
HPDP_00479	K00645	fabD, MCAT, MCT1; [acyl-carrier-protein] S-malonyltransferase [EC:2.3.1.39]	134		
HPDP_00480	K02990	RP-S6, MRPS6, rpsF; small subunit ribosomal protein S6	38		
HPDP_00481	K02963	RP-S18, MRPS18, rpsR; small subunit ribosomal protein S18	21		
HPDP_00482			2	K23509	1
HPDP_00483	K02939	RP-L9, MRPL9, rplI; large subunit ribosomal protein L9	75		
HPDP_00484	K02314	dnaB; replicative DNA helicase [EC:5.6.2.3]	183		
HPDP_00485					
HPDP_00486	K01775	alr; alanine racemase [EC:5.1.1.1]	126		
HPDP_00487	K02427	rlmE, rrmJ, ftsJ; 23S rRNA (uridine2552-2'-O)-methyltransferase [EC:2.1.1.166]	98		
HPDP_00488			3		
HPDP_00489	K03722	dinG; ATP-dependent DNA helicase DinG [EC:5.6.2.3]	177		
HPDP_00490	K02065	mlaF, linL, mkl; phospholipid/cholesterol/gamma-HCH transport system ATP-binding	109		
HPDP_00491	K02066	mlaE, linK; phospholipid/cholesterol/gamma-HCH transport system permease prote	194		
HPDP_00492			28		
HPDP_00493	K04755	fdx; ferredoxin, 2Fe-2S	33	K22071	11
HPDP_00494	K04044	hscA; molecular chaperone HscA	253		
HPDP_00495	K04082	hscB, HSCB, HSC20; molecular chaperone HscB	20		
HPDP_00496	K13628	iscA; iron-sulfur cluster assembly protein	71		
HPDP_00497	K04488	iscU, nifU; nitrogen fixation protein NifU and related proteins	95		

HPDP_00498	K04487	iscS, NFS1; cysteine desulfurase [EC:2.8.1.7]	253		
HPDP_00499	K13643	iscR; Rrf2 family transcriptional regulator, iron-sulfur cluster assembly transcription	58		
HPDP_00500	K07018	K07018; uncharacterized protein	87		
HPDP_00501	K01866	YARS, tyrS; tyrosyl-tRNA synthetase [EC:6.1.1.1]	208		
HPDP_00502			3	K07163	
HPDP_00503	K02836	prfB; peptide chain release factor 2	145		
HPDP_00504	K05366	mrcA; penicillin-binding protein 1A [EC:2.4.1.129 3.4.16.4]	227		
HPDP_00505	K01448	amiABC; N-acetylmuramoyl-L-alanine amidase [EC:3.5.1.28]	63		
HPDP_00506	K08300	rne; ribonuclease E [EC:3.1.26.12]	192		
HPDP_00507			81		
HPDP_00508					
HPDP_00509			3	K03286	1
HPDP_00511	K02434	gatB, PET112; aspartyl-tRNA(Asn)/glutamyl-tRNA(Gln) amidotransferase subunit B [	273		
HPDP_00512	K02433	gatA, QRSL1; aspartyl-tRNA(Asn)/glutamyl-tRNA(Gln) amidotransferase subunit A [E	233		
HPDP_00513	K02435	gatC, GATC; aspartyl-tRNA(Asn)/glutamyl-tRNA(Gln) amidotransferase subunit C [EC	28		
HPDP_00514	K07447	ruvX; putative pre-16S rRNA nuclease [EC:3.1.-.-]	58		
HPDP_00515	K08591	plsY; acyl phosphate:glycerol-3-phosphate acyltransferase [EC:2.3.1.275]	101		
HPDP_00516	K04096	smf; DNA processing protein	135		
HPDP_00517	K03168	topA; DNA topoisomerase I [EC:5.6.2.1]	337		
HPDP_00518	K12573	rnr, vacB; ribonuclease R [EC:3.1.13.1]	117		
HPDP_00519	K02913	RP-L33, MRPL33, rpmG; large subunit ribosomal protein L33	52		
HPDP_00520			1	K05936	1
HPDP_00521	K06180	rluD; 23S rRNA pseudouridine1911/1915/1917 synthase [EC:5.4.99.23]	102		
HPDP_00522			2	K21555	1
HPDP_00523			20		
HPDP_00524	K08303	prtC, trhP; U32 family peptidase [EC:3.4.-.-]	355		
HPDP_00525	K01493	comEB; dCMP deaminase [EC:3.5.4.12]	75		
HPDP_00526			2	K11717	1
HPDP_00527	K03469	rnhA, RNASEH1; ribonuclease HI [EC:3.1.26.4]	95		
HPDP_00528	K07735	algH; putative transcriptional regulator	49		
HPDP_00529			2	K10201	1
HPDP_00530	K03760	eptA, pmrC; lipid A ethanolaminephosphotransferase [EC:2.7.8.43]	25		
HPDP_00531			30		
HPDP_00532	K03215	rumA; 23S rRNA (uracil1939-C5)-methyltransferase [EC:2.1.1.190]	75		
HPDP_00533			134		
HPDP_00534					
HPDP_00535	K01972	E6.5.1.2, ligA, ligB; DNA ligase (NAD+) [EC:6.5.1.2]	202		
HPDP_00536	K03631	recN; DNA repair protein RecN (Recombination protein N)	180		
HPDP_00537	K05807	bamD; outer membrane protein assembly factor BamD	59		
HPDP_00538	K02535	lpxC; UDP-3-O-[3-hydroxymyristoyl] N-acetylglucosamine deacetylase [EC:3.5.1.108]	170		
HPDP_00539			65		
HPDP_00540	K03531	ftsZ; cell division protein FtsZ	94		
HPDP_00541	K03590	ftsA; cell division protein FtsA	10		
HPDP_00542	K03589	ftsQ; cell division protein FtsQ	20		
HPDP_00543	K01921	ddl; D-alanine-D-alanine ligase [EC:6.3.2.4]	112		
HPDP_00544	K00075	murB; UDP-N-acetylmuramate dehydrogenase [EC:1.3.1.98]	122		
HPDP_00545	K01924	murC; UDP-N-acetylmuramate--alanine ligase [EC:6.3.2.8]	193		
HPDP_00546	K02563	murG; UDP-N-acetylglucosamine--N-acetylmuramyl-(pentapeptide) pyrophosphoryl	77		
HPDP_00547	K03588	ftsW, spoVE; cell division protein FtsW	169		
HPDP_00548	K01925	murD; UDP-N-acetylmuramoylalanine--D-glutamate ligase [EC:6.3.2.9]	118		
HPDP_00549	K01000	mraY; phospho-N-acetylmuramoyl-pentapeptide-transferase [EC:2.7.8.13]	133		
HPDP_00550	K01929	murF; UDP-N-acetylmuramoyl-tripeptide--D-alanyl-D-alanine ligase [EC:6.3.2.10]	106		
HPDP_00551	K01928	murE; UDP-N-acetylmuramoyl-L-alanyl-D-glutamate--2,6-diaminopimelate ligase [EC	131	K15792	8
HPDP_00552	K03587	ftsI; cell division protein FtsI (penicillin-binding protein 3) [EC:3.4.16.4]	152		
HPDP_00553			7	K03420	1
HPDP_00554	K03438	mraW, rsmH; 16S rRNA (cytosine1402-N4)-methyltransferase [EC:2.1.1.199]	110		
HPDP_00555					
HPDP_00558					
HPDP_00559			7	K09809	2
HPDP_00560			15	K16868	4
HPDP_00562	K08996	yagU; putative membrane protein	146		
HPDP_00563	K08996	yagU; putative membrane protein	103		
HPDP_00564	K09862	K09862; uncharacterized protein	13		
HPDP_00565	K08301	rng, cafA; ribonuclease G [EC:3.1.26.-]	37		
HPDP_00566	K06287	yhdE; nucleoside triphosphate pyrophosphatase [EC:3.6.1.-]	91		
HPDP_00567	K02518	infA; translation initiation factor IF-1	22		
HPDP_00568	K00790	murA; UDP-N-acetylglucosamine 1-carboxyvinyltransferase [EC:2.5.1.7]	269		
HPDP_00569			7	K01991	
HPDP_00571	K03216	trmL, cspR; tRNA (cytidine/uridine-2'-O-)-methyltransferase [EC:2.1.1.207]	99		

HPDP_00572	K00791	miaA, TRIT1; tRNA dimethylallyltransferase [EC:2.5.1.75]	77		
HPDP_00573	K03569	mreB; rod shape-determining protein MreB and related proteins	198		
HPDP_00574	K03570	mreC; rod shape-determining protein MreC	26		
HPDP_00575					
HPDP_00576	K05515	mrdA; penicillin-binding protein 2 [EC:3.4.16.4]	183		
HPDP_00577	K05837	rodA, mrdB; rod shape determining protein RodA	189		
HPDP_00578	K07263	pqqL; zinc protease [EC:3.4.24.-]	49		
HPDP_00579	K07263	pqqL; zinc protease [EC:3.4.24.-]	95		
HPDP_00580			6	K10441	1
HPDP_00581	K03101	lspA; signal peptidase II [EC:3.4.23.36]	51		
HPDP_00582	K01870	IARS, ileS; isoleucyl-tRNA synthetase [EC:6.1.1.5]	427		
HPDP_00583	K11753	ribF; riboflavin kinase / FMN adenylyltransferase [EC:2.7.1.26 2.7.7.2]	93		
HPDP_00584					
HPDP_00585			1	K00648	1
HPDP_00586	K06020	ettA; energy-dependent translational throttle protein EttA	314		
HPDP_00587			1		
HPDP_00588			68		
HPDP_00589					
HPDP_00590			15	K17278	1
HPDP_00591			3	K00158	1
HPDP_00592	K06956	K06956; uncharacterized protein	235		
HPDP_00593			138	K18367	35
HPDP_00594			61		
HPDP_00595	K00384	trxB, TRR; thioredoxin reductase (NADPH) [EC:1.8.1.9]	162		
HPDP_00596	K02114	ATPF1E, atpC; F-type H+-transporting ATPase subunit epsilon	14	K07263	1
HPDP_00597	K02112	ATPF1B, atpD; F-type H+/Na+-transporting ATPase subunit beta [EC:7.1.2.2 7.2.2.1]	323		
HPDP_00598	K02115	ATPF1G, atpG; F-type H+-transporting ATPase subunit gamma	62		
HPDP_00599	K02111	ATPF1A, atpA; F-type H+/Na+-transporting ATPase subunit alpha [EC:7.1.2.2 7.2.2.1]	313		
HPDP_00600	K02113	ATPF1D, atpH; F-type H+-transporting ATPase subunit delta	38		
HPDP_00601	K04066	priA; primosomal protein N' (replication factor Y) (superfamily II helicase) [EC:3.6.4.-	285		
HPDP_00602	K03733	xerC; integrase/recombinase XerC	110		
HPDP_00603			12	K17837	3
HPDP_00604					
HPDP_00605	K00426	cydB; cytochrome bd ubiquinol oxidase subunit II [EC:7.1.1.7]	181		
HPDP_00606	K00425	cydA; cytochrome bd ubiquinol oxidase subunit I [EC:7.1.1.7]	317		
HPDP_00607	K00133	asd; aspartate-semialdehyde dehydrogenase [EC:1.2.1.11]	147		
HPDP_00608	K02884	RP-L19, MRPL19, rplS; large subunit ribosomal protein L19	82		
HPDP_00609	K00554	trmD; tRNA (guanine37-N1)-methyltransferase [EC:2.1.1.228]	104		
HPDP_00610	K02860	rimM; 16S rRNA processing protein RimM	51		
HPDP_00611	K02959	RP-S16, MRPS16, rpsP; small subunit ribosomal protein S16	34		
HPDP_00612	K03106	SRP54, ffh; signal recognition particle subunit SRP54 [EC:3.6.5.4]	173		
HPDP_00613	K01778	dapF; diaminopimelate epimerase [EC:5.1.1.7]	47		
HPDP_00614	K18707	mtaB; threonylcarbamoyladenosine tRNA methylthiotransferase MtaB [EC:2.8.4.5]	166		
HPDP_00615			125		
HPDP_00616	K00567	ogt, MGMT; methylated-DNA-[protein]-cysteine S-methyltransferase [EC:2.1.1.63]	29		
HPDP_00617			77		
HPDP_00618			17		
HPDP_00619			42	K08217	6
HPDP_00620	K13529	ada-alkA; AraC family transcriptional regulator, regulatory protein of adaptative res	14	K01247	6
HPDP_00621	K01854	glf; UDP-galactopyranose mutase [EC:5.4.99.9]	161		
HPDP_00622	K03110	ftsY; fused signal recognition particle receptor	92		
HPDP_00623	K03559	exbD; biopolymer transport protein ExbD	19	K03560	3
HPDP_00624	K03561	exbB; biopolymer transport protein ExbB	19		
HPDP_00625	K03832	tonB; periplasmic protein TonB	10		
HPDP_00626	K16087	TC.FEV.OM3, tbpA, hemR, lbpA, hpuB, bhuR, hugA, hmbR; hemoglobin/transferrin/l	106		
HPDP_00627					
HPDP_00628					
HPDP_00629	K01591	pyrF; orotidine-5'-phosphate decarboxylase [EC:4.1.1.23]	58		
HPDP_00630	K17828	pyrDI; dihydroorotate dehydrogenase (NAD+) catalytic subunit [EC:1.3.1.14]	21	K00254	5
HPDP_00631			3	K16868	1
HPDP_00632			282		
HPDP_00633	K07316	mod; adenine-specific DNA-methyltransferase [EC:2.1.1.72]	36		
HPDP_00634	K07316	mod; adenine-specific DNA-methyltransferase [EC:2.1.1.72]	37		
HPDP_00635	K01156	res; type III restriction enzyme [EC:3.1.21.5]	307		
HPDP_00636			17	K11530	3
HPDP_00637			10	K04773	1
HPDP_00638	K07341	doc; death on curing protein	51		
HPDP_00640			12	K03275	3
HPDP_00642					

HPDP_00643	K01992	ABC-2.P; ABC-2 type transport system permease protein	99		
HPDP_00644	K03565	recX; regulatory protein	23		
HPDP_00645			27		
HPDP_00646	K00919	ispE; 4-diphosphocytidyl-2-C-methyl-D-erythritol kinase [EC:2.7.1.148]	106		
HPDP_00647			79		
HPDP_00648	K21929	udg; uracil-DNA glycosylase [EC:3.2.2.27]	77		
HPDP_00649	K08309	slt; soluble lytic murein transglycosylase [EC:4.2.2.-]	63		
HPDP_00650			1	K15987	1
HPDP_00651			13	K03611	5
HPDP_00652			1	K03076	1
HPDP_00653			1	K00325	1
HPDP_00654			72		
HPDP_00655	K00927	PGK, pgk; phosphoglycerate kinase [EC:2.7.2.3]	280		
HPDP_00656	K01174	nuc; micrococcal nuclease [EC:3.1.31.1]	26		
HPDP_00657					
HPDP_00658			58		
HPDP_00659			74		
HPDP_00660			14	K00799	1
HPDP_00661			33		
HPDP_00662	K02116	atpI; ATP synthase protein I	18		
HPDP_00663	K02108	ATPF0A, atpB; F-type H+-transporting ATPase subunit a	137		
HPDP_00664					
HPDP_00665	K02109	ATPF0B, atpF; F-type H+-transporting ATPase subunit b	11		
HPDP_00666	K02109	ATPF0B, atpF; F-type H+-transporting ATPase subunit b	11		
HPDP_00667	K01736	aroC; chorismate synthase [EC:4.2.3.5]	219		
HPDP_00668	K12542	lapC; membrane fusion protein, adhesin transport system	131		
HPDP_00669	K12541	lapB; ATP-binding cassette, subfamily C, bacterial LapB	169		
HPDP_00670					
HPDP_00671			2	K21449	1
HPDP_00672	K12543	lapE; outer membrane protein, adhesin transport system	38	K12340	2
HPDP_00673					
HPDP_00674			2		
HPDP_00675			2	K04034	1
HPDP_00676	K00769	gpt; xanthine phosphoribosyltransferase [EC:2.4.2.22]	53		
HPDP_00677					
HPDP_00678	K02238	comEC; competence protein ComEC	73		
HPDP_00680			173		
HPDP_00681	K06911	PIR; quercetin 2,3-dioxygenase [EC:1.13.11.24]	105		
HPDP_00682	K05592	deaD, cshA; ATP-dependent RNA helicase DeaD [EC:3.6.4.13]	55	K11927	42
HPDP_00683	K09760	rmuC; DNA recombination protein RmuC	104		
HPDP_00684	K04750	phnB; PhnB protein	42		
HPDP_00685	K04750	phnB; PhnB protein	18		
HPDP_00686			1	K04080	1
HPDP_00687			11	K04080	10
HPDP_00688	K04758	feoA; ferrous iron transport protein A	16		
HPDP_00689	K04759	feoB; ferrous iron transport protein B	183		
HPDP_00690			8	K19784	1
HPDP_00692	K03431	glmM; phosphoglucosamine mutase [EC:5.4.2.10]	236		
HPDP_00693	K03798	ftsH, hflB; cell division protease FtsH [EC:3.4.24.-]	284		
HPDP_00694	K04075	tilS, mesJ; tRNA(Ile)-lysidine synthase [EC:6.3.4.19]	59		
HPDP_00695			2	K01423	1
HPDP_00696	K03640	pal; peptidoglycan-associated lipoprotein	51		
HPDP_00697	K03641	tolB; TolB protein	78		
HPDP_00698					
HPDP_00699	K03560	tolR; biopolymer transport protein TolR	52		
HPDP_00700	K03562	tolQ; biopolymer transport protein TolQ	46	K03561	2
HPDP_00701	K07107	ybgC; acyl-CoA thioester hydrolase [EC:3.1.2.-]	30		
HPDP_00702	K03551	ruvB; holliday junction DNA helicase RuvB [EC:5.6.2.4]	193		
HPDP_00703	K03550	ruvA; holliday junction DNA helicase RuvA [EC:5.6.2.4]	62		
HPDP_00704	K01159	ruvC; crossover junction endodeoxyribonuclease RuvC [EC:3.1.21.10]	63		
HPDP_00705			133		
HPDP_00706			24		
HPDP_00707	K01934	MTHFS; 5-formyltetrahydrofolate cyclo-ligase [EC:6.3.3.2]	59		
HPDP_00708	K09888	zapA; cell division protein ZapA	10		
HPDP_00709			2	K21826	1
HPDP_00710	K00134	GAPDH, gapA; glyceraldehyde 3-phosphate dehydrogenase (phosphorylating) [EC:1.	236		
HPDP_00711					
HPDP_00712	K02356	efp; elongation factor P	110		
HPDP_00713	K01092	E3.1.3.25, IMPA, suhB; myo-inositol-1(or 4)-monophosphatase [EC:3.1.3.25]	71		

HPDP_00714			3	K13890	1
HPDP_00715	K08151	tetA; MFS transporter, DHA1 family, tetracycline resistance proteir	37		
HPDP_00716	K02909	RP-L31, rpmE; large subunit ribosomal protein L31	47		
HPDP_00717	K08151	tetA; MFS transporter, DHA1 family, tetracycline resistance proteir	32	K08153	15
HPDP_00718	K08151	tetA; MFS transporter, DHA1 family, tetracycline resistance proteir	22	K08153	20
HPDP_00719	K09987	K09987; uncharacterized protein	44		
HPDP_00720	K21071	pfk, pfp; ATP-dependent phosphofructokinase / diphosphate-dependent phosphofr	243		
HPDP_00721	K02970	RP-S21, MRPS21, rpsU; small subunit ribosomal protein S21	46		
HPDP_00722			33	K08153	1
HPDP_00723	K03797	E3.4.21.102, prc, ctpA; carboxyl-terminal processing protease [EC:3.4.21.102]	62		
HPDP_00724	K00275	pdxH, PNPO; pyridoxamine 5'-phosphate oxidase [EC:1.4.3.5]	74		
HPDP_00725			2	K03641	1
HPDP_00726	K00208	fabI; enoyl-[acyl-carrier protein] reductase I [EC:1.3.1.9 1.3.1.10]	162		
HPDP_00727	K04771	degP, htrA; serine protease Do [EC:3.4.21.107]	124		
HPDP_00728	K03593	mrp, NUBPL; ATP-binding protein involved in chromosome partitioning	80		
HPDP_00729			292		
HPDP_00730	K11085	msbA; ATP-binding cassette, subfamily B, bacterial MsbA [EC:7.5.2.6]	199		
HPDP_00731			31		
HPDP_00732	K02527	kdtA, waaA; 3-deoxy-D-manno-octulosonic-acid transferase [EC:2.4.99.12 2.4.99.13	84		
HPDP_00733	K00912	lpxK; tetraacyldisaccharide 4'-kinase [EC:2.7.1.130]	115		
HPDP_00734	K02517	lpxL, htrB; Kdo2-lipid IVA lauroyltransferase/acyltransferase [EC:2.3.1.241 2.3.1.-]	72		
HPDP_00735	K03601	xseA; exodeoxyribonuclease VII large subunit [EC:3.1.11.6]	90		
HPDP_00736	K01810	GPI, pgi; glucose-6-phosphate isomerase [EC:5.3.1.9]	298		
HPDP_00737	K03572	mutL; DNA mismatch repair protein MutL	217		
HPDP_00738	K06890	K06890; uncharacterized protein	152		
HPDP_00739	K06173	truA, PUS1; tRNA pseudouridine38-40 synthase [EC:5.4.99.12]	166		
HPDP_00740	K03466	ftsK, spoIIIE; DNA segregation ATPase FtsK/SpoIIIE, S-DNA-T family	166		
HPDP_00741			27		
HPDP_00742			8	K02483	2
HPDP_00743	K06997	yggS, PROSC; PLP dependent protein	66		
HPDP_00744			2	K00831	1
HPDP_00745			39		
HPDP_00746	K01869	LARS, leuS; leucyl-tRNA synthetase [EC:6.1.1.4]	431		
HPDP_00747					
HPDP_00748	K02340	holA; DNA polymerase III subunit delta [EC:2.7.7.7]	75		
HPDP_00749	K00798	MMAB, pduO; cob(I)alamin adenosyltransferase [EC:2.5.1.17]	46		
HPDP_00750	K01262	pepP; Xaa-Pro aminopeptidase [EC:3.4.11.9]	180		
HPDP_00751			3	K15727	1
HPDP_00752					
HPDP_00753	K09812	ftsE; cell division transport system ATP-binding proteir	97		
HPDP_00754					
HPDP_00755	K00655	plsC; 1-acyl-sn-glycerol-3-phosphate acyltransferase [EC:2.3.1.51]	78		
HPDP_00756	K03465	thyX, thy1; thymidylate synthase (FAD) [EC:2.1.1.148]	154		
HPDP_00761	K09985	K09985; uncharacterized protein	34		
HPDP_00762			1	K01613	1
HPDP_00763					
HPDP_00764	K02919	RP-L36, MRPL36, rpmJ; large subunit ribosomal protein L36	41		
HPDP_00765	K03839	fldA, nifF, isiB; flavodoxin I	26		
HPDP_00766	K07568	queA; S-adenosylmethionine:tRNA ribosyltransferase-isomerase [EC:2.4.99.17]	165		
HPDP_00767	K00773	tgt; queuine tRNA-ribosyltransferase [EC:2.4.2.29]	265		
HPDP_00768	K18979	queG; epoxyqueuosine reductase [EC:1.17.99.6]	90		
HPDP_00769			1	K01448	1
HPDP_00771	K01990	ABC-2.A; ABC-2 type transport system ATP-binding protein	130		
HPDP_00772			7	K03045	1
HPDP_00773	K02335	polA; DNA polymerase I [EC:2.7.7.7]	314		
HPDP_00774			15	K03824	1
HPDP_00775	K09791	K09791; uncharacterized protein	23		
HPDP_00777	K09805	K09805; uncharacterized protein	88		
HPDP_00778			9	K07402	1
HPDP_00779			42		
HPDP_00780	K01520	dut, DUT; dUTP pyrophosphatase [EC:3.6.1.23]	114		
HPDP_00781	K03688	ubiB, aarF; ubiquinone biosynthesis protein	125		
HPDP_00782	K03183	ubiE; demethylmenaquinone methyltransferase / 2-methoxy-6-polyprenyl-1,4-benz	124		
HPDP_00783	K10563	mutM, fpg; formamidopyrimidine-DNA glycosylase [EC:3.2.2.23 4.2.99.18]	106		
HPDP_00784	K02968	RP-S20, rpsT; small subunit ribosomal protein S20	42		
HPDP_00785	K02313	dnaA; chromosomal replication initiator proteir	198		
HPDP_00786	K02338	dnaN; DNA polymerase III subunit beta [EC:2.7.7.7]	148		
HPDP_00787	K03629	recF; DNA replication and repair protein RecF	90		
HPDP_00788			184		

HPDP_00789	K02470	gyrB; DNA gyrase subunit B [EC:5.6.2.2]	474		
HPDP_00790			4	K05366	1
HPDP_00791	K06861	lptB; lipopolysaccharide export system ATP-binding protein [EC:7.5.2.5]	140		
HPDP_00792					
HPDP_00793	K06041	kdsD, kpsF; arabinose-5-phosphate isomerase [EC:5.3.1.13]	144		
HPDP_00794	K03684	rnd; ribonuclease D [EC:3.1.13.5]	113		
HPDP_00795			4	K07071	1
HPDP_00796	K01626	E2.5.1.54, aroF, aroG, aroH; 3-deoxy-7-phosphoheptulonate synthase [EC:2.5.1.54]	113		
HPDP_00797	K00763	pncB, NAPRT1; nicotinate phosphoribosyltransferase [EC:6.3.4.21]	185		
HPDP_00798	K01916	nadE; NAD+ synthase [EC:6.3.1.5]	146	K01950	35
HPDP_00799	K01885	EARS, gltX; glutamyl-tRNA synthetase [EC:6.1.1.17]	196		
HPDP_00800	K01883	CARS, cysS; cysteinyl-tRNA synthetase [EC:6.1.1.16]	212		
HPDP_00801	K09160	K09160; uncharacterized protein	73		
HPDP_00802			48		
HPDP_00803	K03799	htpX; heat shock protein HtpX [EC:3.4.24.-]	133		
HPDP_00804			85		
HPDP_00805	K09780	K09780; uncharacterized protein	44		
HPDP_00806	K00057	gpsA; glycerol-3-phosphate dehydrogenase (NAD(P)+) [EC:1.1.1.94]	120		
HPDP_00807	K01409	KAE1, tsaD, QRI7; N6-L-threonylcarbamoyladenine synthase [EC:2.3.1.234]	162		
HPDP_00808			1	K02496	1
HPDP_00809			2	K02498	2
HPDP_00810			31		
HPDP_00811	K00459	ncd2, npd; nitronate monooxygenase [EC:1.13.12.16]	210		
HPDP_00812	K00962	pnp, PNPT1; polyribonucleotide nucleotidyltransferase [EC:2.7.7.8]	379		
HPDP_00813	K02956	RP-S15, MRPS15, rpsO; small subunit ribosomal protein S15	64		
HPDP_00814	K03177	truB, PUS4, TRUB1; tRNA pseudouridine55 synthase [EC:5.4.99.25]	102		
HPDP_00815	K02834	rbfA; ribosome-binding factor A	46		
HPDP_00816	K02519	infB, MTIF2; translation initiation factor IF-2	194		
HPDP_00817	K02600	nusA; transcription termination/antitermination protein NusA	197		
HPDP_00818	K09748	rimP; ribosome maturation factor RimP	33		
HPDP_00819	K03439	trmB, METTL1, TRM8; tRNA (guanine-N7-)-methyltransferase [EC:2.1.1.33]	70		
HPDP_00820	K00789	metK, MAT; S-adenosylmethionine synthetase [EC:2.5.1.6]	219		
HPDP_00821			37	K03820	1
HPDP_00822	K03820	lnt; apolipoprotein N-acyltransferase [EC:2.3.1.269]	73		
HPDP_00823	K06189	corC, tlyC; hemolysin (HlyC) family protein	82		
HPDP_00824	K07042	ybeY, yqfG; probable rRNA maturation factor	18		
HPDP_00825	K06168	miaB; tRNA-2-methylthio-N6-dimethylallyladenosine synthase [EC:2.8.4.3]	240		
HPDP_00826	K14742	tsaB; tRNA threonylcarbamoyladenosine biosynthesis protein TsaB	23		
HPDP_00827	K03796	bax; Bax protein	33		
HPDP_00828			77	K22074	5
HPDP_00829	K01867	WARS, trpS; tryptophanyl-tRNA synthetase [EC:6.1.1.2]	197		
HPDP_00830	K03980	murJ, mviN; putative peptidoglycan lipid II flippase	125		
HPDP_00831	K03555	mutS; DNA mismatch repair protein MutS	315		
HPDP_00832					
HPDP_00833					
HPDP_00834			4	K07275	2
HPDP_00835			1	K01463	1
HPDP_00836	K01507	ppa; inorganic pyrophosphatase [EC:3.6.1.1]	102		
HPDP_00837					
HPDP_00838			2	K18889	1
HPDP_00839			34		
HPDP_00840			40		
HPDP_00841	K02342	dnaQ; DNA polymerase III subunit epsilon [EC:2.7.7.7]	84		
HPDP_00842	K00859	coaE; dephospho-CoA kinase [EC:2.7.1.24]	48		
HPDP_00843	K00014	aroE; shikimate dehydrogenase [EC:1.1.1.25]	55		
HPDP_00844	K03628	rho; transcription termination factor Rho	299		
HPDP_00845	K03650	mnmE, trmE, MSS1; tRNA modification GTPase [EC:3.6.-.-]	147		
HPDP_00846			14	K18697	1
HPDP_00847	K03495	gidA, mnmG, MTO1; tRNA uridine 5-carboxymethylaminomethyl modification enzyr	322		
HPDP_00848			5		
HPDP_00849	K03501	gidB, rsmG; 16S rRNA (guanine527-N7)-methyltransferase [EC:2.1.1.170]	35		
HPDP_00850	K03497	parB, spo0J; ParB family transcriptional regulator, chromosome partitioning proteir	84		
HPDP_00851	K03496	parA, soj; chromosome partitioning protein	145		
HPDP_00852					
HPDP_00853	K11991	tadA; tRNA(adenine34) deaminase [EC:3.5.4.33]	60		
HPDP_00854	K06178	rluB; 23S rRNA pseudouridine2605 synthase [EC:5.4.99.22]	52		
HPDP_00855	K08316	rsmD; 16S rRNA (guanine966-N2)-methyltransferase [EC:2.1.1.171]	49		
HPDP_00856			2	K00943	2
HPDP_00857			5	K02986	1

HPDP_00858			23	K01867	1
HPDP_00859			15	K21686	1
HPDP_00860			2	K06395	1
HPDP_00861	K02503	HINT1, hinT, hit; histidine triad (HIT) family protein	53		
HPDP_00862	K01419	hslV, clpQ; ATP-dependent HslUV protease, peptidase subunit HslV [EC:3.4.25.2]	109		
HPDP_00863	K03667	hslU; ATP-dependent HslUV protease ATP-binding subunit HslU	253		
HPDP_00864	K07391	comM; magnesium chelatase family protein	241		
HPDP_00865	K14623	dinD; DNA-damage-inducible protein D	63		
HPDP_00866			15		
HPDP_00867	K02040	pstS; phosphate transport system substrate-binding protein	134		
HPDP_00868	K02037	pstC; phosphate transport system permease protein	162		
HPDP_00869	K02038	pstA; phosphate transport system permease protein	153		
HPDP_00870	K02036	pstB; phosphate transport system ATP-binding protein [EC:7.3.2.1]	167		
HPDP_00871	K02039	phoU; phosphate transport system protein	46		
HPDP_00872			1	K07275	1
HPDP_00873	K00625	E2.3.1.8, pta; phosphate acetyltransferase [EC:2.3.1.8]	97		
HPDP_00874	K00925	ackA; acetate kinase [EC:2.7.2.1]	170		
HPDP_00875					
HPDP_00876	K06218	relE, stbE; mRNA interferase RelE/StbE	26		
HPDP_00877	K01784	galE, GALE; UDP-glucose 4-epimerase [EC:5.1.3.2]	129	K12448	29
HPDP_00878	K10773	NTH; endonuclease III [EC:4.2.99.18]	107		
HPDP_00879	K03686	dnaJ; molecular chaperone DnaJ	144		
HPDP_00880	K04043	dnaK, HSPA9; molecular chaperone DnaK	433		
HPDP_00881	K03687	GRPE; molecular chaperone GrpE	34		
HPDP_00882	K01295	cpg; glutamate carboxypeptidase [EC:3.4.17.11]	52		
HPDP_00883	K00989	rph; ribonuclease PH [EC:2.7.7.56]	134		
HPDP_00884	K01519	rdgB, ITPA; XTP/dITP diphosphohydrolase [EC:3.6.1.66]	94		
HPDP_00885			123		
HPDP_00886	K14623	dinD; DNA-damage-inducible protein D	129		
HPDP_00887			125		
HPDP_00888			39		
HPDP_00889	K07056	rsmI; 16S rRNA (cytidine1402-2'-O)-methyltransferase [EC:2.1.1.198]	106		
HPDP_00890	K07460	yraN; putative endonuclease	29		
HPDP_00891	K01920	gshB; glutathione synthase [EC:6.3.2.3]	150		
HPDP_00892	K00604	MTFMT, fmt; methionyl-tRNA formyltransferase [EC:2.1.2.9]	123		
HPDP_00893	K01462	PDF, def; peptide deformylase [EC:3.5.1.88]	97		
HPDP_00894	K06187	recR; recombination protein RecR	87		
HPDP_00895	K09747	ebfC; nucleoid-associated protein EbfC	45		
HPDP_00896	K09125	yhhQ; queuosine precursor transporter	21		
HPDP_00897	K23993	fruB; multiphosphoryl transfer protein [EC:2.7.3.9 2.7.1.202]	239		
HPDP_00898			47	K01952	1
HPDP_00899	K00571	E2.1.1.72; site-specific DNA-methyltransferase (adenine-specific) [EC:2.1.1.72]	122		
HPDP_00900	K02804	nagE; N-acetylglucosamine PTS system EIICBA or EIICB component [EC:2.7.1.193]	198		
HPDP_00901	K02564	nagB, GNPDA; glucosamine-6-phosphate deaminase [EC:3.5.99.6]	141		
HPDP_00902			3	K07991	1
HPDP_00903	K01443	nagA, AMDHD2; N-acetylglucosamine-6-phosphate deacetylase [EC:3.5.1.25]	155		
HPDP_00904	K07501	K07501; 3'-5' exonuclease	118		
HPDP_00905			107		
HPDP_00906	K01895	ACSS1_2, acs; acetyl-CoA synthetase [EC:6.2.1.1]	309		
HPDP_00907			14	K16087	1
HPDP_00908	K02343	dnaX; DNA polymerase III subunit gamma/tau [EC:2.7.7.7]	101		
HPDP_00910			4	K18940	1
HPDP_00911	K06223	dam; DNA adenine methylase [EC:2.1.1.72]	126		
HPDP_00912	K06941	rlmN; 23S rRNA (adenine2503-C2)-methyltransferase [EC:2.1.1.192]	155		
HPDP_00913	K00979	kdsB; 3-deoxy-manno-octulosonate cytidylyltransferase (CMP-KDO synthetase) [EC:	116		
HPDP_00914	K13893	yejA; microcin C transport system substrate-binding protein	81	K02035	11
HPDP_00915	K13894	yejB; microcin C transport system permease protein	127		
HPDP_00916	K13895	yejE; microcin C transport system permease protein	73		
HPDP_00917	K02031	ddpD; peptide/nickel transport system ATP-binding protein	26	K13896	23
	K02032	ddpF; peptide/nickel transport system ATP-binding protein			
HPDP_00918	K06153	bacA; undecaprenyl-diphosphatase [EC:3.6.1.27]	107		
HPDP_00919	K14170	pheA; chorismate mutase / prephenate dehydratase [EC:5.4.99.5 4.2.1.51]	13		
HPDP_00920	K01069	gloB, gloC, HAGH; hydroxyacylglutathione hydrolase [EC:3.1.2.6]	72		
HPDP_00921	K03784	deoD; purine-nucleoside phosphorylase [EC:2.4.2.1]	140		
HPDP_00922			33	K09809	3
HPDP_00923	K01839	deoB; phosphopentomutase [EC:5.4.2.7]	222		
HPDP_00924			246		
HPDP_00925	K01868	TARS, thrS; threonyl-tRNA synthetase [EC:6.1.1.3]	351		
HPDP_00926	K02520	infC, MTIF3; translation initiation factor IF-3	70		

HPDP_00927	K02916	RP-L35, MRPL35, rpmI; large subunit ribosomal protein L35	37		
HPDP_00928	K02887	RP-L20, MRPL20, rplT; large subunit ribosomal protein L20	78		
HPDP_00929	K01889	FARSA, pheS; phenylalanyl-tRNA synthetase alpha chain [EC:6.1.1.20]	169		
HPDP_00930	K01890	FARSB, pheT; phenylalanyl-tRNA synthetase beta chain [EC:6.1.1.20]	260		
HPDP_00931			128		
HPDP_00932			8	K02237	1
HPDP_00933	K01243	mtnN, mtn, pfs; adenosylhomocysteine nucleosidase [EC:3.2.2.9]	86		
HPDP_00934	K01183	E3.2.1.14; chitinase [EC:3.2.1.14]	73		
HPDP_00935	K01183	E3.2.1.14; chitinase [EC:3.2.1.14]	11		
HPDP_00936			1	K04759	1
HPDP_00937	K04077	groEL, HSPD1; chaperonin GroEL	408		
HPDP_00938	K04078	groES, HSPE1; chaperonin GroES	85		
HPDP_00939			152		
HPDP_00940	K03657	uvrD, pcrA; ATP-dependent DNA helicase UvrD/PcrA [EC:5.6.2.4]	293		
HPDP_00941	K00525	E1.17.4.1A, nrdA, nrdE; ribonucleoside-diphosphate reductase alpha chain [EC:1.17.	348		
HPDP_00942	K00526	E1.17.4.1B, nrdB, nrdF; ribonucleoside-diphosphate reductase beta chain [EC:1.17.4	187		
HPDP_00943	K03647	nrdI; protein involved in ribonucleotide reductior	34		
HPDP_00944	K02687	prmA; ribosomal protein L11 methyltransferase [EC:2.1.1.-]	37		
HPDP_00945	K08281	pncA; nicotinamidase/pyrazinamidase [EC:3.5.1.19 3.5.1.-]	112		
HPDP_00946	K03116	tatA; sec-independent protein translocase protein TatA	19		
HPDP_00947	K04042	glmU; bifunctional UDP-N-acetylglucosamine pyrophosphorylase / glucosamine-1-p	180		
HPDP_00948					
HPDP_00949			3	K03561	1
HPDP_00950	K03470	rnhB; ribonuclease HII [EC:3.1.26.4]	88		
HPDP_00951	K01129	dgt; dGTPase [EC:3.1.5.1]	145		
HPDP_00952	K16264	czcD, zitB; cobalt-zinc-cadmium efflux system proteir	87		
HPDP_00953	K03596	lepA; GTP-binding protein LepA	433		
HPDP_00954	K01835	pgm; phosphoglucomutase [EC:5.4.2.2]	226		
HPDP_00955	K01491	folD; methylenetetrahydrofolate dehydrogenase (NADP+) / methenyltetrahydrofola	118		
HPDP_00956	K02221	yggT; YggT family protein	14	K02031	1
HPDP_00957	K00942	gmk, GUK1; guanylate kinase [EC:2.7.4.8]	84		
HPDP_00958	K02528	ksgA; 16S rRNA (adenine1518-N6/adenine1519-N6)-dimethyltransferase [EC:2.1.1.1	83		
HPDP_00959	K00097	pdxA; 4-hydroxythreonine-4-phosphate dehydrogenase [EC:1.1.1.262]	104		
HPDP_00960	K03771	surA; peptidyl-prolyl cis-trans isomerase SurA [EC:5.2.1.8]	23		
HPDP_00961	K04744	lptD, imp, ostA; LPS-assembly protein	51		
HPDP_00962	K11720	lptG; lipopolysaccharide export system permease proteir	57		
HPDP_00963			176		
HPDP_00964	K07566	tsaC, rimN, SUA5; L-threonylcarbamoyladenylate synthase [EC:2.7.7.87]	109		
HPDP_00965	K01082	cysQ, MET22, BPNT1; 3'(2'), 5'-bisphosphate nucleotidase [EC:3.1.3.7]	71		
HPDP_00966	K01255	CARP, pepA; leucyl aminopeptidase [EC:3.4.11.1]	248		
HPDP_00967	K02339	holC; DNA polymerase III subunit chi [EC:2.7.7.7]	43		
HPDP_00968			5	K12524	1
HPDP_00969			42		
HPDP_00970					
HPDP_00971			12	K06338	2
HPDP_00972			33	K19427	2
HPDP_00973	K13581	ccrM; modification methylase [EC:2.1.1.72]	179		
HPDP_00974			146		
HPDP_00975			21	K19591	1
HPDP_00976	K09859	K09859; uncharacterized protein	36		
HPDP_00977	K07337	K07337; penicillin-binding protein activator	40		
HPDP_00978			5	K03151	1
HPDP_00979			2	K23743	2
HPDP_00980			2	K23743	2
HPDP_00981	K06905	K06905; uncharacterized protein	20		
HPDP_00982			3	K02443	1
HPDP_00983					
HPDP_00984	K06903	K06903; uncharacterized protein	16		
HPDP_00985			2	K16200	1
HPDP_00986			8	K00390	1
HPDP_00987	K06908	K06908; uncharacterized protein	54		
HPDP_00988	K06907	K06907; uncharacterized protein	101		
HPDP_00989			8	K08086	1
HPDP_00990			37		
HPDP_00991			51		
HPDP_00992					
HPDP_00993			4	K23997	1
HPDP_00994			3	K02067	1
HPDP_00995			20	K03279	2

HPDP_00996			19		
HPDP_00997			6	K01933	1
HPDP_00999	K21498	higA-1; antitoxin HigA-1	29		
HPDP_01000	K07334	higB-1; toxin HigB-1	54		
HPDP_01001	K24845	ubiV; O2-independent ubiquinone biosynthesis protein UbiV	155		
HPDP_01002	K24844	ubiU; O2-independent ubiquinone biosynthesis protein UbiU	186		
HPDP_01003	K24843	ubiT; O2-independent ubiquinone biosynthesis accessory factor UbiT	18	K24843/K24844	1
HPDP_01004					
HPDP_01006			1	K10747	1
HPDP_01007	K02914	RP-L34, MRPL34, rpmH; large subunit ribosomal protein L34	35		
HPDP_01008	K03536	rnpA; ribonuclease P protein component [EC:3.1.26.5]	23		
HPDP_01009	K08998	K08998; uncharacterized protein	43		
HPDP_01010	K03217	yidC, spoIIIJ, OXA1, ccfA; YidC/Oxa1 family membrane protein insertase	219		
HPDP_01011	K03978	engB; GTP-binding protein	83		
HPDP_01012	K03299	TC.GNTP; gluconate:H+ symporter, GntP family	168		
HPDP_01013			54		
HPDP_01014	K13292	lgt, umpA; phosphatidylglycerol---prolipoprotein diacylglyceryl transferase [EC:2.5.1	140		
HPDP_01015	K18164	NDUFAF7; NADH dehydrogenase [ubiquinone] 1 alpha subcomplex assembly factor	74		
HPDP_01016	K06942	ychF; ribosome-binding ATPase	227		
HPDP_01017	K01056	PTH1, pth, spoVC; peptidyl-tRNA hydrolase, PTH1 family [EC:3.1.1.29]	83		
HPDP_01018	K02897	RP-L25, rplY; large subunit ribosomal protein L25	72		
HPDP_01019	K00948	PRPS, prsA; ribose-phosphate pyrophosphokinase [EC:2.7.6.1]	189		
HPDP_01020	K05810	yfiH; polyphenol oxidase [EC:1.10.3.-]	125		
HPDP_01021	K07638	envZ; two-component system, OmpR family, osmolarity sensor histidine kinase EnvZ	95		
HPDP_01022	K07659	ompR; two-component system, OmpR family, phosphate regulon response regulato	71	K02483	5
HPDP_01023			106		
HPDP_01024	K08744	CRLS; cardiolipin synthase (CMP-forming) [EC:2.7.8.41]	71	K00995	5
HPDP_01025	K03703	uvrC; excinuclease ABC subunit C	221		
HPDP_01026	K08311	nudH; putative (di)nucleoside polyphosphate hydrolase [EC:3.6.1.-]	86		
HPDP_01027	K03797	E3.4.21.102, prc, ctpA; carboxyl-terminal processing protease [EC:3.4.21.102]	104		
HPDP_01028	K22719	envC; murein hydrolase activator	16		
HPDP_01029	K15633	gpmI; 2,3-bisphosphoglycerate-independent phosphoglycerate mutase [EC:5.4.2.12	251		
HPDP_01030	K00783	rlmH; 23S rRNA (pseudouridine1915-N3)-methyltransferase [EC:2.1.1.177]	46		
HPDP_01031	K09710	ybeB; ribosome-associated protein	36		
HPDP_01032	K00969	nadD; nicotinate-nucleotide adenylyltransferase [EC:2.7.7.18]	68		
HPDP_01033					
HPDP_01034	K03979	obgE, cgtA, MTG2; GTPase [EC:3.6.5.-]	146		
HPDP_01035	K02899	RP-L27, MRPL27, rpmA; large subunit ribosomal protein L27	69		
HPDP_01036	K02888	RP-L21, MRPL21, rplU; large subunit ribosomal protein L21	54		
HPDP_01037	K01295	cpg; glutamate carboxypeptidase [EC:3.4.17.11]	128		
HPDP_01038			15	K08973	1
HPDP_01039			9	K09761	1
HPDP_01040			7	K03074	1
HPDP_01043	K00800	aroA; 3-phosphoshikimate 1-carboxyvinyltransferase [EC:2.5.1.19]	207		
HPDP_01044	K00945	cmk; CMP/dCMP kinase [EC:2.7.4.25]	71		
HPDP_01045	K02945	RP-S1, rpsA; small subunit ribosomal protein S1	255		
HPDP_01046	K01963	accD; acetyl-CoA carboxylase carboxyl transferase subunit beta [EC:6.4.1.2 2.1.3.15]	147		
HPDP_01047	K11754	folC; dihydrofolate synthase / folylpolyglutamate synthase [EC:6.3.2.12 6.3.2.17]	124		
HPDP_01048	K03671	trxA; thioredoxin 1	53		
HPDP_01049	K16898	addA; ATP-dependent helicase/nuclease subunit A [EC:5.6.2.4 3.1.-.-]	258		
HPDP_01050	K16899	addB; ATP-dependent helicase/nuclease subunit B [EC:5.6.2.4 3.1.-.-]	161		
HPDP_01051	K06925	tsaE; tRNA threonylcarbamoyladenosine biosynthesis protein TsaE	31	K07102	1
HPDP_01052	K00384	trxB, TRR; thioredoxin reductase (NADPH) [EC:1.8.1.9]	155		
HPDP_01053	K03498	trkH, trkG, ktrB, ktrD; trk/ktr system potassium uptake protein	226		
HPDP_01054					
HPDP_01055			1	K21449	1
HPDP_01056					
HPDP_01057					
HPDP_01058			6	K03798	1
HPDP_01060	K03322	mntH; manganese transport protein	277		
HPDP_01061			19	K01356	3
HPDP_01062			3	K18555	1
HPDP_01063			12	K01447	1
HPDP_01064			24	K02081	15
HPDP_01065					
HPDP_01066	K01424	E3.5.1.1, ansA, ansB; L-asparaginase [EC:3.5.1.1]	10	K05597	2
HPDP_01067					
HPDP_01068	K03218	rlmB; 23S rRNA (guanosine2251-2'-O)-methyltransferase [EC:2.1.1.185]	75		
HPDP_01071					

HPDP_01073	K03073	secE; preprotein translocase subunit SecE	23		
HPDP_01074	K02601	nusG; transcription termination/antitermination protein NusG	76		
HPDP_01075	K02867	RP-L11, MRPL11, rplK; large subunit ribosomal protein L11	93		
HPDP_01076	K02863	RP-L1, MRPL1, rplA; large subunit ribosomal protein L1	172		
HPDP_01077	K02864	RP-L10, MRPL10, rplJ; large subunit ribosomal protein L10	67		
HPDP_01078	K02935	RP-L7, MRPL12, rplL; large subunit ribosomal protein L7/L12	79		
HPDP_01079	K03043	rpoB; DNA-directed RNA polymerase subunit beta [EC:2.7.7.6]	784		
HPDP_01080	K03046	rpoC; DNA-directed RNA polymerase subunit beta' [EC:2.7.7.6]	752	K13797	43
HPDP_01081	K02950	RP-S12, MRPS12, rpsL; small subunit ribosomal protein S12	142		
HPDP_01082	K02992	RP-S7, MRPS7, rpsG; small subunit ribosomal protein S7	116		
HPDP_01083	K02355	fusA, GFM, EFG; elongation factor G	522		
HPDP_01084	K02358	tuf, TUFM; elongation factor Tu	60		
HPDP_01085	K02946	RP-S10, MRPS10, rpsJ; small subunit ribosomal protein S10	112		
HPDP_01086	K02906	RP-L3, MRPL3, rplC; large subunit ribosomal protein L3	173		
HPDP_01087	K02926	RP-L4, MRPL4, rplD; large subunit ribosomal protein L4	106		
HPDP_01088	K02892	RP-L23, MRPL23, rplW; large subunit ribosomal protein L23	47	K02926	1
HPDP_01089	K02886	RP-L2, MRPL2, rplB; large subunit ribosomal protein L2	190		
HPDP_01090	K02965	RP-S19, rpsS; small subunit ribosomal protein S19	93		
HPDP_01091	K02890	RP-L22, MRPL22, rplV; large subunit ribosomal protein L22	74		
HPDP_01092	K02982	RP-S3, rpsC; small subunit ribosomal protein S3	143		
HPDP_01093	K02878	RP-L16, MRPL16, rplP; large subunit ribosomal protein L16	148		
HPDP_01094	K02904	RP-L29, rpmC; large subunit ribosomal protein L29	32		
HPDP_01095	K02961	RP-S17, MRPS17, rpsQ; small subunit ribosomal protein S17	42		
HPDP_01096	K02874	RP-L14, MRPL14, rplN; large subunit ribosomal protein L14	98		
HPDP_01097	K02895	RP-L24, MRPL24, rplX; large subunit ribosomal protein L24	56		
HPDP_01098	K02931	RP-L5, MRPL5, rplE; large subunit ribosomal protein L5	118		
HPDP_01099	K02954	RP-S14, MRPS14, rpsN; small subunit ribosomal protein S14	62		
HPDP_01100	K02994	RP-S8, rpsH; small subunit ribosomal protein S8	66		
HPDP_01101	K02933	RP-L6, MRPL6, rplF; large subunit ribosomal protein L6	137		
HPDP_01102	K02881	RP-L18, MRPL18, rplR; large subunit ribosomal protein L18	57		
HPDP_01103	K02988	RP-S5, MRPS5, rpsE; small subunit ribosomal protein S5	114		
HPDP_01104	K02907	RP-L30, MRPL30, rpmD; large subunit ribosomal protein L30	58		
HPDP_01105	K02876	RP-L15, MRPL15, rplO; large subunit ribosomal protein L15	80		
HPDP_01106	K03076	secY; preprotein translocase subunit SecY	301		
HPDP_01107	K00939	adk, AK; adenylate kinase [EC:2.7.4.3]	87		
HPDP_01108	K02952	RP-S13, rpsM; small subunit ribosomal protein S13	89		
HPDP_01109	K02948	RP-S11, MRPS11, rpsK; small subunit ribosomal protein S11	91		
HPDP_01110	K03040	rpoA; DNA-directed RNA polymerase subunit alpha [EC:2.7.7.6]	229		
HPDP_01111	K02879	RP-L17, MRPL17, rplQ; large subunit ribosomal protein L17	102		
HPDP_01112			2		
HPDP_01113			84	K04771	5
HPDP_01114			2	K07496	2
HPDP_01115	K06179	rluC; 23S rRNA pseudouridine955/2504/2580 synthase [EC:5.4.99.24]	80		
HPDP_01116	K06190	ispZ; intracellular septation protein	88		
HPDP_01117	K03801	lipB; lipoyl(octanoyl) transferase [EC:2.3.1.181]	80		
HPDP_01119	K06213	mgtE; magnesium transporter	118		
HPDP_01120	K07091	lptF; lipopolysaccharide export system permease protein	34		
HPDP_01121	K00858	ppnK, NADK; NAD+ kinase [EC:2.7.1.23]	81		
HPDP_01122	K13588	chpT; histidine phosphotransferase ChpT	42		
HPDP_01123	K13584	ctrA; two-component system, cell cycle response regulator CtrA	105		
HPDP_01124	K01611	speD, AMD1; S-adenosylmethionine decarboxylase [EC:4.1.1.50]	83		
HPDP_01125	K00797	speE, SRM, SPE3; spermidine synthase [EC:2.5.1.16]	136		
HPDP_01126	K13587	cckA; two-component system, cell cycle sensor histidine kinase and response regula	87		
HPDP_01127	K03553	recA; recombination protein RecA	214		
HPDP_01128	K01872	AARS, alaS; alanyl-tRNA synthetase [EC:6.1.1.7]	370		
HPDP_01129			4	K03768	2
HPDP_01130	K00873	PK, pyk; pyruvate kinase [EC:2.7.1.40]	237		
HPDP_01131	K03585	acrA, mexA, adeI, smeD, mtrC, cmeA; membrane fusion protein, multidrug efflux sys	24		
HPDP_01132			275	K03296	72
HPDP_01133	K12340	tolC, bepC, cyaE, raxC, sapF, rsaF, hasF; outer membrane protein	84		
HPDP_01135	K02770	fruA; fructose PTS system EIIBC or EIIC component [EC:2.7.1.202]	223		
HPDP_01136	K03702	uvrB; excinuclease ABC subunit B	347		
HPDP_01137	K01095	pgpA; phosphatidylglycerophosphatase A [EC:3.1.3.27]	37		
HPDP_01138	K03624	greA; transcription elongation factor GreA	91		
HPDP_01139	K09117	K09117; uncharacterized protein	59		
HPDP_01140	K02316	dnaG; DNA primase [EC:2.7.7.101]	122		
HPDP_01141	K03086	rpoD; RNA polymerase primary sigma factor	185		
HPDP_01143			9	K18678	4
HPDP_01144	K11041	eta; exfoliative toxin A/B	98		

HPDP_01145	K03760	eptA, pmrC; lipid A ethanolaminephosphotransferase [EC:2.7.8.43]	77		
HPDP_01146	K03760	eptA, pmrC; lipid A ethanolaminephosphotransferase [EC:2.7.8.43]	33	K12975	13

Query	KO	Definition	Score	Second best	Score
HPPR_00001					
HPPR_00002					
HPPR_00003					
HPPR_00004					
HPPR_00005					
HPPR_00006			2	K23743	2
HPPR_00007			1	K23743	1
HPPR_00008			2	K23743	1
HPPR_00009			1	K23743	1
HPPR_00010			2	K23743	1
HPPR_00011			239		
HPPR_00012	K01652	E2.2.1.6L, ilvB, ilvG, ilvI; acetolactate synthase I/II/III large subunit [EC:2.2.1.6]	317		
HPPR_00013	K01647	CS, gltA; citrate synthase [EC:2.3.3.1]	217		
HPPR_00014	K00031	IDH1, IDH2, icd; isocitrate dehydrogenase [EC:1.1.1.42]	250		
HPPR_00015	K01681	ACO, acnA; aconitate hydratase [EC:4.2.1.3]	800		
HPPR_00016	K00024	mdh; malate dehydrogenase [EC:1.1.1.37]	197		
HPPR_00017	K13821	putA; RHH-type transcriptional regulator, proline utilization regulon repressor / pro	383		
HPPR_00018	K11928	putP; sodium/proline symporter	211		
HPPR_00019	K14155	patB, malY; cysteine-S-conjugate beta-lyase [EC:4.4.1.13]	141		
HPPR_00020	K01489	cdd, CDA; cytidine deaminase [EC:3.5.4.5]	55		
HPPR_00021	K00758	deoA, TYMP; thymidine phosphorylase [EC:2.4.2.4]	216		
HPPR_00022	K01619	deoC, DERA; deoxyribose-phosphate aldolase [EC:4.1.2.4]	103		
HPPR_00023	K03317	TC.CNT; concentrative nucleoside transporter, CNT family	217		
HPPR_00026	K01666	mhpE; 4-hydroxy 2-oxovalerate aldolase [EC:4.1.3.39]	60	K18365	3
HPPR_00027			5	K01055	1
HPPR_00028			141	K00030	9
HPPR_00029	K03742	pncC; nicotinamide-nucleotide amidase [EC:3.5.1.42]	29		
HPPR_00030			101		
HPPR_00031			33		
HPPR_00032	K07552	bcr, tcaB; MFS transporter, DHA1 family, multidrug resistance protein	126		
HPPR_00033			2	K03550	1
HPPR_00034			2	K00674	1
HPPR_00035					
HPPR_00036					
HPPR_00037	K06207	typA, bipA; GTP-binding protein	374		
HPPR_00038			39	K15270	7
HPPR_00039			31	K15270	7
HPPR_00040			39	K15270	6
HPPR_00043			39	K09809	1
HPPR_00044	K00831	serC, PSAT1; phosphoserine aminotransferase [EC:2.6.1.52]	128		
HPPR_00046			38	K09809	1
HPPR_00047	K03070	secA; preprotein translocase subunit SecA [EC:7.4.2.8]	448		
HPPR_00048	K03558	cvpA; membrane protein required for colicin V production	16		
HPPR_00049			14	K02673	1
HPPR_00050	K17713	bamB; outer membrane protein assembly factor BamB	30		
HPPR_00051	K03977	engA, der; GTPase	164		
HPPR_00052	K04485	radA, sms; DNA repair protein RadA/Sms	222		
HPPR_00053					
HPPR_00054	K17103	CHO1, pssA; CDP-diacylglycerol---serine O-phosphatidyltransferase [EC:2.7.8.8]	60		
HPPR_00055	K01613	psd, PISD; phosphatidylserine decarboxylase [EC:4.1.1.65]	147		
HPPR_00056	K01878	glyQ; glycyl-tRNA synthetase alpha chain [EC:6.1.1.14]	175		
HPPR_00057	K01879	glyS; glycyl-tRNA synthetase beta chain [EC:6.1.1.14]	172		
HPPR_00058	K02779	ptsG; glucose PTS system EIICB or EIICBA component [EC:2.7.1.199]	214		
HPPR_00060			2	K02483	1
HPPR_00061			2	K01183	1
HPPR_00062			3	K02357	1
HPPR_00063	K06980	ygfZ; tRNA-modifying protein YgfZ	57	K22073	11
HPPR_00064	K01919	gshA; glutamate--cysteine ligase [EC:6.3.2.2]	215		
HPPR_00065	K09761	rsmE; 16S rRNA (uracil1498-N3)-methyltransferase [EC:2.1.1.193]	58		
HPPR_00066	K03179	ubiA; 4-hydroxybenzoate polyprenyltransferase [EC:2.5.1.39]	73		
HPPR_00067	K03182	ubiD; 4-hydroxy-3-polyprenylbenzoate decarboxylase [EC:4.1.1.98]	302		
HPPR_00068	K04566	lysK; lysyl-tRNA synthetase, class I [EC:6.1.1.6]	289		
HPPR_00069	K13583	gcrA; GcrA cell cycle regulator	18		
HPPR_00070			66		

HPPR_00071			23	K02004	17
HPPR_00072			23	K09808	14
HPPR_00073	K02003	ABC.CD.A; putative ABC transport system ATP-binding protein	101		
HPPR_00074			18	K21573	1
HPPR_00075			3	K25152	1
HPPR_00076			28	K22708	1
HPPR_00077			15	K18704	1
HPPR_00078			2	K03286	1
HPPR_00079			13	K01183	1
HPPR_00080			2	K05845	1
HPPR_00081			11	K07079	1
HPPR_00082	K02986	RP-S4, rpsD; small subunit ribosomal protein S4	145		
HPPR_00083	K21469	pbp4b; serine-type D-Ala-D-Ala carboxypeptidase [EC:3.4.16.4]	138		
HPPR_00084					
HPPR_00085			2	K08997	1
HPPR_00086	K06999	K06999; phospholipase/carboxylesterase	45		
HPPR_00087			2	K01092	1
HPPR_00088	K07390	grxD, GLRX5; monothiol glutaredoxin	20		
HPPR_00089	K03630	radC; DNA repair protein RadC	95		
HPPR_00090	K01265	map; methionyl aminopeptidase [EC:3.4.11.18]	126		
HPPR_00091	K06206	sfsA; sugar fermentation stimulation protein A	90		
HPPR_00092			32	K18162	14
HPPR_00093			58	K02242	4
HPPR_00094	K03676	grxC, GLRX, GLRX2; glutaredoxin 3	46		
HPPR_00095	K00568	ubiG; 2-polyprenyl-6-hydroxyphenyl methylase / 3-demethylubiquinone-9 3-methyl	84		
HPPR_00096	K02371	fabK; enoyl-[acyl-carrier protein] reductase II [EC:1.3.1.9]	188		
HPPR_00097					
HPPR_00098	K03526	gcpE, ispG; (E)-4-hydroxy-3-methylbut-2-enyl-diphosphate synthase [EC:1.17.7.1 1.1	218		
HPPR_00099	K01892	HARS, hisS; histidyl-tRNA synthetase [EC:6.1.1.21]	174		
HPPR_00100	K02835	prfA, MTRF1, MRF1; peptide chain release factor 1	217		
HPPR_00101	K02493	hemK, prmC, HEMK; release factor glutamine methyltransferase [EC:2.1.1.297]	82		
HPPR_00102	K02871	RP-L13, MRPL13, rplM; large subunit ribosomal protein L13	135		
HPPR_00103	K02996	RP-S9, MRPS9, rpsI; small subunit ribosomal protein S9	96		
HPPR_00104			2	K00342	1
HPPR_00105			3	K07273	1
HPPR_00106	K09816	znuB; zinc transport system permease protein	62		
HPPR_00107	K09817	znuC; zinc transport system ATP-binding protein [EC:7.2.2.20]	78		
HPPR_00108	K01999	livK; branched-chain amino acid transport system substrate-binding protein	41		
HPPR_00109			69		
HPPR_00110	K00566	mnmA, trmU; tRNA-uridine 2-sulfurtransferase [EC:2.8.1.13]	127		
HPPR_00111			74		
HPPR_00112	K01875	SARS, serS; seryl-tRNA synthetase [EC:6.1.1.11]	191		
HPPR_00113			28	K06194	1
HPPR_00114	K03210	yajC; preprotein translocase subunit YajC	35		
HPPR_00115	K03072	secD; preprotein translocase subunit SecD	93	K12257	45
HPPR_00116	K03074	secF; preprotein translocase subunit SecF	79	K12257	8
HPPR_00117			12	K09008	1
HPPR_00118	K08305	mltB; membrane-bound lytic murein transglycosylase B [EC:4.2.2.-]	27		
HPPR_00119	K06867	K06867; uncharacterized protein	10		
HPPR_00120	K09815	znuA; zinc transport system substrate-binding protein	10	K19975	1
HPPR_00121	K09815	znuA; zinc transport system substrate-binding protein	42		
HPPR_00122	K03089	rpoH; RNA polymerase sigma-32 factor	112		
HPPR_00125			289		
HPPR_00126	K03923	mdaB; NADPH dehydrogenase (quinone) [EC:1.6.5.10]	128		
HPPR_00127			2	K08311	1
HPPR_00128			4	K03427	3
HPPR_00129			110		
HPPR_00130	K17686	copA, ctpA, ATP7; P-type Cu+ transporter [EC:7.2.2.8]	288		
HPPR_00131			3	K07727	1
HPPR_00133	K00022	HADH; 3-hydroxyacyl-CoA dehydrogenase [EC:1.1.1.35]	103		
HPPR_00134	K02440	GLPF; glycerol uptake facilitator protein	112		
HPPR_00135	K00864	glpK, GK; glycerol kinase [EC:2.7.1.30]	321		
HPPR_00136	K00111	glpA, glpD; glycerol-3-phosphate dehydrogenase [EC:1.1.5.3]	217		
HPPR_00137	K03564	BCP, PRXQ, DOT5; thioredoxin-dependent peroxiredoxin [EC:1.11.1.24]	66		
HPPR_00138	K03564	BCP, PRXQ, DOT5; thioredoxin-dependent peroxiredoxin [EC:1.11.1.24]	55		

HPPR_00139			19	K19427	2
HPPR_00140	K08344	scsB; suppressor for copper-sensitivity B	121		
HPPR_00141			95		
HPPR_00142	K22044	ybiO; moderate conductance mechanosensitive channel	35		
HPPR_00143	K00759	APRT, apt; adenine phosphoribosyltransferase [EC:2.4.2.7]	108		
HPPR_00144			1	K20382	1
HPPR_00145	K02523	ispB; octaprenyl-diphosphate synthase [EC:2.5.1.90]	167		
HPPR_00146			52	K15460	10
HPPR_00147			113		
HPPR_00148	K02902	RP-L28, MRPL28, rpmB; large subunit ribosomal protein L28	68		
HPPR_00149					
HPPR_00150	K03699	tlyC; magnesium and cobalt exporter, CNNM family	139		
HPPR_00151	K01735	aroB; 3-dehydroquinate synthase [EC:4.2.3.4]	86	K13829	13
HPPR_00152	K00891	aroK, aroL; shikimate kinase [EC:2.7.1.71]	47		
HPPR_00153			4		
HPPR_00154	K04763	xerD; integrase/recombinase XerD	117		
HPPR_00155	K01962	accA; acetyl-CoA carboxylase carboxyl transferase subunit alpha [EC:6.4.1.2 2.1.3.15	173		
HPPR_00157	K02257	COX10, ctaB, cyoE; heme o synthase [EC:2.5.1.141]	123		
HPPR_00158	K02297	cyoA; cytochrome o ubiquinol oxidase subunit II [EC:7.1.1.3]	118		
HPPR_00159	K02298	cyoB; cytochrome o ubiquinol oxidase subunit I [EC:7.1.1.3]	489		
HPPR_00160	K02299	cyoC; cytochrome o ubiquinol oxidase subunit III	123		
HPPR_00161	K02300	cyoD; cytochrome o ubiquinol oxidase subunit IV	37	K02299	1
HPPR_00162			7	K05606	5
HPPR_00163	K03305	TC.POT; proton-dependent oligopeptide transporter, POT family	162		
HPPR_00164					
HPPR_00165	K19303	mepH; murein DD-endopeptidase [EC:3.4.-.-]	21		
HPPR_00166			36	K02404	1
HPPR_00167	K00571	E2.1.1.72; site-specific DNA-methyltransferase (adenine-specific) [EC:2.1.1.72]	111		
HPPR_00168			60		
HPPR_00169			8		
HPPR_00170			2	K08369	1
HPPR_00171	K03545	tig; trigger factor	126		
HPPR_00172	K01358	clpP, CLPP; ATP-dependent Clp protease, protease subunit [EC:3.4.21.92]	191		
HPPR_00173	K03544	clpX, CLPX; ATP-dependent Clp protease ATP-binding subunit ClpX	202		
HPPR_00174	K01338	lon; ATP-dependent Lon protease [EC:3.4.21.53]	368		
HPPR_00175	K03530	hupB; DNA-binding protein HU-beta	52		
HPPR_00179	K01752	E4.3.1.17, sdaA, sdaB, tdcG; L-serine dehydratase [EC:4.3.1.17]	207		
HPPR_00180	K07240	chrA; chromate transporter	34		
HPPR_00181	K07240	chrA; chromate transporter	31		
HPPR_00182	K01207	nagZ; beta-N-acetylhexosaminidase [EC:3.2.1.52]	122		
HPPR_00183			64		
HPPR_00184	K05304	NANS, SAS; sialic acid synthase [EC:2.5.1.56 2.5.1.57 2.5.1.132]	167	K01654	32
HPPR_00185			9	K11085	1
HPPR_00186			1	K01270	1
HPPR_00187	K03778	ldhA; D-lactate dehydrogenase [EC:1.1.1.28]	169		
HPPR_00188					
HPPR_00189	K01961	accC; acetyl-CoA carboxylase, biotin carboxylase subunit [EC:6.4.1.2 6.3.4.14]	280		
HPPR_00190	K02160	accB, bccP; acetyl-CoA carboxylase biotin carboxyl carrier protein	41		
HPPR_00191	K03786	aroQ, qutE; 3-dehydroquinate dehydratase II [EC:4.2.1.10]	76		
HPPR_00192	K01581	E4.1.1.17, ODC1, speC, speF; ornithine decarboxylase [EC:4.1.1.17]	201		
HPPR_00193			37		
HPPR_00194	K03308	TC.NSS; neurotransmitter:Na+ symporter, NSS family	175		
HPPR_00195	K03308	TC.NSS; neurotransmitter:Na+ symporter, NSS family	139		
HPPR_00196			57		
HPPR_00197			5	K03286	3
HPPR_00198	K07462	recJ; single-stranded-DNA-specific exonuclease [EC:3.1.-.-]	170		
HPPR_00200			4	K00615	1
HPPR_00201					
HPPR_00202			4	K03286	2
HPPR_00203			5	K07646	1
HPPR_00204	K00820	glmS, GFPT; glutamine---fructose-6-phosphate transaminase (isomerizing) [EC:2.6.1	295		
HPPR_00205	K11746	kefF; glutathione-regulated potassium-efflux system ancillary protein KefF	23		
HPPR_00206					
HPPR_00207	K06199	crcB, FEX; fluoride exporter	36		
HPPR_00208			101		

HPPR_00209			3	K03286	2
HPPR_00210	K03595	era, ERAL1; GTPase	105		
HPPR_00211	K03584	recO; DNA repair protein RecO (recombination protein O)	104		
HPPR_00212	K00573	E2.1.1.77, pcm; protein-L-isoaspartate(D-aspartate) O-methyltransferase [EC:2.1.1.7	38		
HPPR_00213					
HPPR_00214	K01873	VARS, valS; valyl-tRNA synthetase [EC:6.1.1.9]	414		
HPPR_00215	K00330	nuoA; NADH-quinone oxidoreductase subunit A [EC:7.1.1.2]	59		
HPPR_00216	K00331	nuoB; NADH-quinone oxidoreductase subunit B [EC:7.1.1.2]	150		
HPPR_00217	K00332	nuoC; NADH-quinone oxidoreductase subunit C [EC:7.1.1.2]	89		
HPPR_00218	K00333	nuoD; NADH-quinone oxidoreductase subunit D [EC:7.1.1.2]	315		
HPPR_00219	K00334	nuoE; NADH-quinone oxidoreductase subunit E [EC:7.1.1.2]	61	K03943	14
HPPR_00220	K00335	nuoF; NADH-quinone oxidoreductase subunit F [EC:7.1.1.2]	245		
HPPR_00221	K00336	nuoG; NADH-quinone oxidoreductase subunit G [EC:7.1.1.2]	304		
HPPR_00222	K00337	nuoH; NADH-quinone oxidoreductase subunit H [EC:7.1.1.2]	215		
HPPR_00223	K00338	nuoI; NADH-quinone oxidoreductase subunit I [EC:7.1.1.2]	101		
HPPR_00224	K00339	nuoJ; NADH-quinone oxidoreductase subunit J [EC:7.1.1.2]	65		
HPPR_00225	K00340	nuoK; NADH-quinone oxidoreductase subunit K [EC:7.1.1.2]	52		
HPPR_00226	K00341	nuoL; NADH-quinone oxidoreductase subunit L [EC:7.1.1.2]	221		
HPPR_00227	K00342	nuoM; NADH-quinone oxidoreductase subunit M [EC:7.1.1.2]	168		
HPPR_00228	K00343	nuoN; NADH-quinone oxidoreductase subunit N [EC:7.1.1.2]	128		
HPPR_00229	K03524	birA; BirA family transcriptional regulator, biotin operon repressor / biotin---[acetyl-	45		
HPPR_00230	K03525	coaX; type III pantothenate kinase [EC:2.7.1.33]	37		
HPPR_00231	K12574	rnj; ribonuclease J [EC:3.1.-.-]	159		
HPPR_00232	K00459	ncd2, npd; nitronate monooxygenase [EC:1.13.12.16]	83		
HPPR_00233	K25227	sdsA1; linear primary-alkylsulfatase [EC:3.1.6.21]	304		
HPPR_00234	K06901	pbuG, azgA, ghxP, ghxQ, adeQ; adenine/guanine/hypoxanthine permease	156		
HPPR_00235	K01881	PARS, proS; prolyl-tRNA synthetase [EC:6.1.1.15]	246		
HPPR_00236	K09808	lolC_E; lipoprotein-releasing system permease protein	198		
HPPR_00237	K09810	lolD; lipoprotein-releasing system ATP-binding protein [EC:7.6.2.-]	98		
HPPR_00238	K02337	dnaE; DNA polymerase III subunit alpha [EC:2.7.7.7]	355		
HPPR_00239			1	K06006	1
HPPR_00240	K06949	rsgA, engC; ribosome biogenesis GTPase / thiamine phosphate phosphatase [EC:3.6	103		
HPPR_00241			1	K02313	1
HPPR_00242			14	K07006	3
HPPR_00243			8	K02392	1
HPPR_00244			1	K01799	1
HPPR_00245	K04564	SOD2; superoxide dismutase, Fe-Mn family [EC:1.15.1.1]	110		
HPPR_00246			1	K00215	1
HPPR_00247			2	K03079	1
HPPR_00248					
HPPR_00249	K03642	rlpA; rare lipoprotein A	75		
HPPR_00250	K07258	dacC, dacA, dacD; serine-type D-Ala-D-Ala carboxypeptidase (penicillin-binding prot	101		
HPPR_00251	K00943	tmk, DTYMK; dTMP kinase [EC:2.7.4.9]	16		
HPPR_00252	K02341	holB; DNA polymerase III subunit delta' [EC:2.7.7.7]	48		
HPPR_00253			26	K14654	2
HPPR_00254	K00374	narI, narV; nitrate reductase gamma subunit [EC:1.7.5.1 1.7.99.-]	20		
HPPR_00255			1		
HPPR_00256	K01286	E3.4.16.4; D-alanyl-D-alanine carboxypeptidase [EC:3.4.16.4]	73	K07258	21
HPPR_00257	K00525	E1.17.4.1A, nrdA, nrdE; ribonucleoside-diphosphate reductase alpha chain [EC:1.17.	655		
HPPR_00258			37		
HPPR_00259	K02067	mlaD, linM; phospholipid/cholesterol/gamma-HCH transport system substrate-bind	13		
HPPR_00260	K02067	mlaD, linM; phospholipid/cholesterol/gamma-HCH transport system substrate-bind	52		
HPPR_00261			19		
HPPR_00262			2	K03601	1
HPPR_00263			88		
HPPR_00264	K03701	uvrA; excinuclease ABC subunit A	590		
HPPR_00265	K03111	ssb; single-strand DNA-binding protein	72		
HPPR_00266	K03270	kdsC; 3-deoxy-D-manno-octulosonate 8-phosphate phosphatase (KDO 8-P phosphat	54		
HPPR_00267	K03811	pnuC; nicotinamide mononucleotide transporter	26		
HPPR_00268	K03811	pnuC; nicotinamide mononucleotide transporter	28		
HPPR_00269	K02967	RP-S2, MRPS2, rpsB; small subunit ribosomal protein S2	132		
HPPR_00270	K02357	tsf, TSFM; elongation factor Ts	162		
HPPR_00271	K09903	pyrH; uridylate kinase [EC:2.7.4.22]	132		
HPPR_00272	K02838	frr, MRRF, RRF; ribosome recycling factor	82		
HPPR_00273	K00806	uppS; undecaprenyl diphosphate synthase [EC:2.5.1.31]	95		

HPPR_00274			77		
HPPR_00275					
HPPR_00276			7	K01921	1
HPPR_00277			3	K05832	1
HPPR_00278	K03664	smpB; SsrA-binding protein	59		
HPPR_00279	K00981	E2.7.7.41, CDS1, CDS2, cdsA; phosphatidate cytidylyltransferase [EC:2.7.7.41]	29		
HPPR_00280	K17837	bla2, blm, ccrA, blaB; metallo-beta-lactamase class B [EC:3.5.2.6]	39	K18782	6
HPPR_00281			2	K16363	1
HPPR_00282			1	K02926	1
HPPR_00283					
HPPR_00284	K03303	lctP; lactate permease	144		
HPPR_00285	K01095	pgpA; phosphatidylglycerophosphatase A [EC:3.1.3.27]	13	K05837	1
HPPR_00286			2	K25027	1
HPPR_00287	K03060	rpoZ; DNA-directed RNA polymerase subunit omega [EC:2.7.7.6]	22		
HPPR_00288	K01139	spoT; GTP diphosphokinase / guanosine-3',5'-bis(diphosphate) 3'-diphosphatase [EC	208		
HPPR_00289	K03474	pdxJ; pyridoxine 5-phosphate synthase [EC:2.6.99.2]	116		
HPPR_00290	K00997	acpS; holo-[acyl-carrier protein] synthase [EC:2.7.8.7]	52		
HPPR_00291	K03100	lepB; signal peptidase I [EC:3.4.21.89]	87		
HPPR_00292	K03685	rnc, DROSHA, RNT1; ribonuclease III [EC:3.1.26.3]	61	K03595	2
HPPR_00293			18	K21687	1
HPPR_00294	K01624	FBA, fbaA; fructose-bisphosphate aldolase, class II [EC:4.1.2.13]	208		
HPPR_00295			27	K02199	3
HPPR_00296	K01478	arcA; arginine deiminase [EC:3.5.3.6]	166		
HPPR_00297	K00611	OTC, argF, argI; ornithine carbamoyltransferase [EC:2.1.3.3]	275		
HPPR_00298			203		
HPPR_00299	K00926	arcC; carbamate kinase [EC:2.7.2.2]	183		
HPPR_00300					
HPPR_00301					
HPPR_00302			1	K10206	1
HPPR_00303			13	K19334	8
HPPR_00304	K03655	recG; ATP-dependent DNA helicase RecG [EC:5.6.2.4]	229		
HPPR_00305	K03723	mfd; transcription-repair coupling factor (superfamily II helicase) [EC:5.6.2.4]	374		
HPPR_00306			91	K00721	11
HPPR_00307	K01476	E3.5.3.1, rocF, arg; arginase [EC:3.5.3.1]	76	K00819	5
HPPR_00308	K03832	tonB; periplasmic protein TonB	10		
HPPR_00309	K16092	btuB; vitamin B12 transporter	56		
HPPR_00311			19	K03327	7
HPPR_00312			1	K00625	1
HPPR_00313			3	K00337	1
HPPR_00314	K02073	metQ; D-methionine transport system substrate-binding protein	115		
HPPR_00315	K02073	metQ; D-methionine transport system substrate-binding protein	154		
HPPR_00316	K02072	metI; D-methionine transport system permease protein	112		
HPPR_00317	K02071	metN; D-methionine transport system ATP-binding protein	145		
HPPR_00318	K00615	E2.2.1.1, tktA, tktB; transketolase [EC:2.2.1.1]	272		
HPPR_00319	K01783	rpe, RPE; ribulose-phosphate 3-epimerase [EC:5.1.3.1]	113		
HPPR_00320			78		
HPPR_00321			2	K03286	1
HPPR_00322					
HPPR_00323	K19286	nfrA2; FMN reductase [NAD(P)H] [EC:1.5.1.39]	82		
HPPR_00324	K02804	nagE; N-acetylglucosamine PTS system EIICBA or EIICB component [EC:2.7.1.193]	203		
HPPR_00325			1	K07165	1
HPPR_00326	K03652	MPG; DNA-3-methyladenine glycosylase [EC:3.2.2.21]	27		
HPPR_00327			21	K03724	1
HPPR_00328	K03308	TC.NSS; neurotransmitter:Na+ symporter, NSS family	307		
HPPR_00329	K00259	ald; alanine dehydrogenase [EC:1.4.1.1]	199		
HPPR_00330	K03770	ppiD; peptidyl-prolyl cis-trans isomerase D [EC:5.2.1.8]	48		
HPPR_00331	K01803	TPI, tpiA; triosephosphate isomerase (TIM) [EC:5.3.1.1]	151		
HPPR_00332	K03075	secG; preprotein translocase subunit SecG	11		
HPPR_00333	K01937	pyrG, CTPS; CTP synthase [EC:6.3.4.2]	319		
HPPR_00334	K01627	kdsA; 2-dehydro-3-deoxyphosphooctonate aldolase (KDO 8-P synthase) [EC:2.5.1.55	177		
HPPR_00335	K01689	ENO, eno; enolase [EC:4.2.1.11]	296		
HPPR_00336			2	K01753	1
HPPR_00337					
HPPR_00338					
HPPR_00339	K02445	glpT; MFS transporter, OPA family, glycerol-3-phosphate transporter	231		

HPPR_00340			4	K05589	2
HPPR_00341	K00161	PDHA, pdhA; pyruvate dehydrogenase E1 component alpha subunit [EC:1.2.4.1]	162		
HPPR_00342	K00162	PDHB, pdhB; pyruvate dehydrogenase E1 component beta subunit [EC:1.2.4.1]	278		
HPPR_00343	K00627	DLAT, aceF, pdhC; pyruvate dehydrogenase E2 component (dihydrolipoamide acety	205		
HPPR_00344	K00382	DLD, lpd, pdhD; dihydrolipoamide dehydrogenase [EC:1.8.1.4]	299		
HPPR_00345			13		
HPPR_00346			2	K06075	1
HPPR_00347			2	K13922	1
HPPR_00348	K01750	E4.3.1.12, ocd; ornithine cyclodeaminase [EC:4.3.1.12]	160		
HPPR_00349	K07504	K07504; predicted type IV restriction endonuclease	160		
HPPR_00350			1	K03828	1
HPPR_00351			3	K06889	1
HPPR_00352			9	K23186	1
HPPR_00353			13	K00799	4
HPPR_00354	K01679	E4.2.1.2B, fumC, FH; fumarate hydratase, class II [EC:4.2.1.2]	262		
HPPR_00355	K14445	SLC13A2_3_5; solute carrier family 13 (sodium-dependent dicarboxylate transporte	159		
HPPR_00356	K03644	lipA, LIAS, LIP1, LIP5; lipoyl synthase [EC:2.8.1.8]	179		
HPPR_00357	K18588	COQ10; coenzyme Q-binding protein COQ10	51		
HPPR_00358	K01874	MARS, metG; methionyl-tRNA synthetase [EC:6.1.1.10]	275		
HPPR_00359			23	K03772	17
HPPR_00360			1	K01854	1
HPPR_00361					
HPPR_00362			1	K03639	1
HPPR_00363	K03424	tatD; TatD DNase family protein [EC:3.1.21.-]	148		
HPPR_00364	K06167	phnP; phosphoribosyl 1,2-cyclic phosphate phosphodiesterase [EC:3.1.4.55]	85		
HPPR_00365			8	K02519	1
HPPR_00366			9	K03885	1
HPPR_00367			11	K16381	1
HPPR_00368	K03665	hflX; GTPase	84		
HPPR_00369	K03666	hfq; host factor-I protein	41		
HPPR_00370	K03499	trkA, ktrA, ktrC; trk/ktr system potassium uptake protein	76		
HPPR_00371	K09159	cptB; antitoxin CptB	23		
HPPR_00372			2	K00384	1
HPPR_00373			1	K02004	1
HPPR_00374			2	K02035	1
HPPR_00375			2	K02338	2
HPPR_00376	K00240	sdhB, frdB; succinate dehydrogenase / fumarate reductase, iron-sulfur subunit [EC:1	181		
HPPR_00377	K00239	sdhA, frdA; succinate dehydrogenase / fumarate reductase, flavoprotein subunit [EC	411		
HPPR_00378	K00242	sdhD, frdD; succinate dehydrogenase / fumarate reductase, membrane anchor subu	24		
HPPR_00379	K00241	sdhC, frdC; succinate dehydrogenase / fumarate reductase, cytochrome b subunit	37		
HPPR_00380	K07791	dcuA; anaerobic C4-dicarboxylate transporter DcuA	228		
HPPR_00381	K07791	dcuA; anaerobic C4-dicarboxylate transporter DcuA	184		
HPPR_00382	K01744	aspA; aspartate ammonia-lyase [EC:4.3.1.1]	221		
HPPR_00383	K05540	dusB; tRNA-dihydrouridine synthase B [EC:1.-.-.-]	150		
HPPR_00384	K12506	ispDF; 2-C-methyl-D-erythritol 4-phosphate cytidylyltransferase / 2-C-methyl-D-eryt	139		
HPPR_00385	K00748	lpxB; lipid-A-disaccharide synthase [EC:2.4.1.182]	160		
HPPR_00386	K01759	GLO1, gloA; lactoylglutathione lyase [EC:4.4.1.5]	77		
HPPR_00387	K06867	K06867; uncharacterized protein	12		
HPPR_00388	K25227	sdsA1; linear primary-alkylsulfatase [EC:3.1.6.21]	297		
HPPR_00389			21		
HPPR_00390	K00963	UGP2, galU, galF; UTP--glucose-1-phosphate uridylyltransferase [EC:2.7.7.9]	139		
HPPR_00391	K01256	pepN; aminopeptidase N [EC:3.4.11.2]	322		
HPPR_00392			49		
HPPR_00393			1		
HPPR_00394	K00088	IMPDH, guaB; IMP dehydrogenase [EC:1.1.1.205]	107		
HPPR_00395			1	K03979	1
HPPR_00396	K01756	purB, ADSL; adenylosuccinate lyase [EC:4.3.2.2]	237		
HPPR_00397	K01939	purA, ADSS; adenylosuccinate synthase [EC:6.3.4.4]	242		
HPPR_00398	K03186	ubiX, bsdB, PAD1; flavin prenyltransferase [EC:2.5.1.129]	116		
HPPR_00399					
HPPR_00400			2	K02437	1
HPPR_00401	K04094	trmFO, gid; methylenetetrahydrofolate--tRNA-(uracil-5-)-methyltransferase [EC:2.1.	213		
HPPR_00402			5	K22227	1
HPPR_00403	K00648	fabH; 3-oxoacyl-[acyl-carrier-protein] synthase III [EC:2.3.1.180]	178		
HPPR_00404	K03621	plsX; phosphate acyltransferase [EC:2.3.1.274]	147		

HPPR_00405	K02911	RP-L32, MRPL32, rpmF; large subunit ribosomal protein L32	48		
HPPR_00406			15	K07040	1
HPPR_00407			310		
HPPR_00408			4	K13652	1
HPPR_00409			70	K01607	2
HPPR_00410			13	K08222	1
HPPR_00411			12	K06186	6
HPPR_00412	K00946	thiL; thiamine-monophosphate kinase [EC:2.7.4.16]	125		
HPPR_00413	K00600	glyA, SHMT; glycine hydroxymethyltransferase [EC:2.1.2.1]	246		
HPPR_00414	K01808	rpiB; ribose 5-phosphate isomerase B [EC:5.3.1.6]	61		
HPPR_00415	K00761	upp, UPRT; uracil phosphoribosyltransferase [EC:2.4.2.9]	83		
HPPR_00416	K02824	uraA, pyrP; uracil permease	271		
HPPR_00417			2	K23743	1
HPPR_00418			37		
HPPR_00419			2	K06044	1
HPPR_00420	K09949	lpxI; UDP-2,3-diacylglucosamine hydrolase [EC:3.6.1.54]	102		
HPPR_00421	K00677	lpxA; UDP-N-acetylglucosamine acyltransferase [EC:2.3.1.129]	121		
HPPR_00422	K02372	fabZ; 3-hydroxyacyl-[acyl-carrier-protein] dehydratase [EC:4.2.1.59]	56	K16363	5
HPPR_00423	K02536	lpxD; UDP-3-O-[3-hydroxymyristoyl] glucosamine N-acyltransferase [EC:2.3.1.191]	118		
HPPR_00424	K06142	hlpA, ompH; outer membrane protein	14		
HPPR_00425	K01809	manA, MPI; mannose-6-phosphate isomerase [EC:5.3.1.8]	33		
HPPR_00426	K07277	SAM50, TOB55, bamA; outer membrane protein insertion porin family	216		
HPPR_00427	K11749	rseP; regulator of sigma E protease [EC:3.4.24.-]	107		
HPPR_00428	K00099	dxr; 1-deoxy-D-xylulose-5-phosphate reductoisomerase [EC:1.1.1.267]	134		
HPPR_00429			10	K07251	4
HPPR_00430	K03811	pnuC; nicotinamide mononucleotide transporter	44		
HPPR_00431	K01885	EARS, gltX; glutamyl-tRNA synthetase [EC:6.1.1.17]	236		
HPPR_00432			58		
HPPR_00433	K03118	tatC; sec-independent protein translocase protein TatC	85		
HPPR_00434			2	K01286	1
HPPR_00435	K01887	RARS, argS; arginyl-tRNA synthetase [EC:6.1.1.19]	257		
HPPR_00436	K15724	erpA; iron-sulfur cluster insertion protein	23		
HPPR_00437	K01142	E3.1.11.2, xthA; exodeoxyribonuclease III [EC:3.1.11.2]	109		
HPPR_00438			2	K00150	1
HPPR_00440			1	K02556	1
HPPR_00441			2	K11904	1
HPPR_00442	K01662	dxs; 1-deoxy-D-xylulose-5-phosphate synthase [EC:2.2.1.7]	233		
HPPR_00443	K13789	GGPS; geranylgeranyl diphosphate synthase, type II [EC:2.5.1.1 2.5.1.10 2.5.1.29]	38	K00795	21
HPPR_00444	K03602	xseB; exodeoxyribonuclease VII small subunit [EC:3.1.11.6]	17		
HPPR_00445	K01876	DARS2, aspS; aspartyl-tRNA synthetase [EC:6.1.1.12]	373		
HPPR_00446			25		
HPPR_00447			5	K03183	1
HPPR_00448			6		
HPPR_00449			2	K01875	1
HPPR_00451			20		
HPPR_00452			9	K25153	1
HPPR_00454			18	K03972	4
HPPR_00455			23	K02439	1
HPPR_00456	K05517	tsx; nucleoside-specific channel-forming protein	11		
HPPR_00457			217		
HPPR_00458			2	K01652	1
HPPR_00459			2	K07126	1
HPPR_00460			16	K00948	1
HPPR_00461			13	K00703	1
HPPR_00462					
HPPR_00463			22	K13924	1
HPPR_00464					
HPPR_00465			5	K07459	2
HPPR_00466			107		
HPPR_00467			32	K06909	1
HPPR_00468			1	K18700	1
HPPR_00469			2	K03529	1
HPPR_00470			22	K03657	1
HPPR_00471	K05569	mnhE, mrpE; multicomponent Na+:H+ antiporter subunit E	33		
HPPR_00472	K05570	mnhF, mrpF; multicomponent Na+:H+ antiporter subunit F	11		

HPPR_00473	K05571	mnhG, mrpG; multicomponent Na+:H+ antiporter subunit G	10	K05564	2
HPPR_00474	K05566	mnhB, mrpB; multicomponent Na+:H+ antiporter subunit B	77		
HPPR_00475	K05567	mnhC, mrpC; multicomponent Na+:H+ antiporter subunit C	32		
HPPR_00476	K05568	mnhD, mrpD; multicomponent Na+:H+ antiporter subunit D	112		
HPPR_00477	K05568	mnhD, mrpD; multicomponent Na+:H+ antiporter subunit D	219		
HPPR_00478	K05568	mnhD, mrpD; multicomponent Na+:H+ antiporter subunit D	65		
HPPR_00479			37		
HPPR_00480					
HPPR_00481			4	K07219	1
HPPR_00482			3	K03274	1
HPPR_00483	K01610	E4.1.1.49, pckA; phosphoenolpyruvate carboxykinase (ATP) [EC:4.1.1.49]	270		
HPPR_00484			2	K24160	1
HPPR_00485			10	K21394	1
HPPR_00486	K04047	dps; starvation-inducible DNA-binding protein	61		
HPPR_00487	K03760	eptA, pmrC; lipid A ethanolaminephosphotransferase [EC:2.7.8.43]	154		
HPPR_00488			3	K02380	1
HPPR_00489					
HPPR_00490	K00009	mtlD; mannitol-1-phosphate 5-dehydrogenase [EC:1.1.1.17]	154		
HPPR_00491	K02800	mtlA, cmtA; mannitol PTS system EIICBA or EIICB component [EC:2.7.1.197]	351		
HPPR_00492					
HPPR_00493			3	K17472	1
HPPR_00494			3	K03664	1
HPPR_00495			1	K01042	1
HPPR_00496			3	K01992	1
HPPR_00497	K04095	fic; cell filamentation protein	54		
HPPR_00498	K03837	sdaC; serine transporter	193		
HPPR_00499			3	K00020	1
HPPR_00500	K07552	bcr, tcaB; MFS transporter, DHA1 family, multidrug resistance protein	149		
HPPR_00501	K03788	aphA; acid phosphatase (class B) [EC:3.1.3.2]	58		
HPPR_00502			2	K03638	0
HPPR_00503			2	K21498	1
HPPR_00504			4	K07726	1
HPPR_00505			12	K07219	1
HPPR_00506			31		
HPPR_00507	K19092	parE1_3_4; toxin ParE1/3/4	15		
HPPR_00508			10	K18918	3
HPPR_00509			1	K00847	1
HPPR_00510			1	K04069	1
HPPR_00511			1	K05984	1
HPPR_00512	K01185	E3.2.1.17; lysozyme [EC:3.2.1.17]	42		
HPPR_00513			2	K03296	1
HPPR_00514			2	K07250	1
HPPR_00515			1	K03284	1
HPPR_00516			3	K16918	1
HPPR_00517					
HPPR_00518			1	K10914	1
HPPR_00519					
HPPR_00520			2	K16092	1
HPPR_00521			1	K00941	1
HPPR_00522			2	K03665	1
HPPR_00523			6	K01246	1
HPPR_00524			2	K22186	2
HPPR_00525			5	K01011	1
HPPR_00526			1	K02073	1
HPPR_00527			1	K06889	1
HPPR_00528			1	K02027	1
HPPR_00529					
HPPR_00531	K00954	E2.7.7.3A, coaD, kdtB; pantetheine-phosphate adenylyltransferase [EC:2.7.7.3]	73		
HPPR_00532	K02469	gyrA; DNA gyrase subunit A [EC:5.6.2.2]	460		
HPPR_00533	K07276	K07276; uncharacterized protein	53		
HPPR_00534	K03527	ispH, lytB; 4-hydroxy-3-methylbut-2-en-1-yl diphosphate reductase [EC:1.17.7.4]	174		
HPPR_00535	K07323	mlaC; phospholipid transport system substrate-binding protein	13		
HPPR_00536	K00970	pcnB; poly(A) polymerase [EC:2.7.7.19]	101		
HPPR_00537			26		
HPPR_00538	K07082	K07082; UPF0755 protein	124		

HPPR_00539	K09458	fabF, OXSM, CEM1; 3-oxoacyl-[acyl-carrier-protein] synthase II [EC:2.3.1.179]	232		
HPPR_00540	K02078	acpP; acyl carrier protein	46		
HPPR_00541	K00059	fabG, OAR1; 3-oxoacyl-[acyl-carrier protein] reductase [EC:1.1.1.100]	139		
HPPR_00542	K00645	fabD, MCAT, MCT1; [acyl-carrier-protein] S-malonyltransferase [EC:2.3.1.39]	135		
HPPR_00543	K02990	RP-S6, MRPS6, rpsF; small subunit ribosomal protein S6	38		
HPPR_00544	K02963	RP-S18, MRPS18, rpsR; small subunit ribosomal protein S18	26		
HPPR_00545			2		
HPPR_00546	K02939	RP-L9, MRPL9, rplI; large subunit ribosomal protein L9	70		
HPPR_00547	K02314	dnaB; replicative DNA helicase [EC:5.6.2.3]	186		
HPPR_00548	K01775	alr; alanine racemase [EC:5.1.1.1]	130		
HPPR_00549	K02427	rlmE, rrmJ, ftsJ; 23S rRNA (uridine2552-2'-O)-methyltransferase [EC:2.1.1.166]	94		
HPPR_00550			3		
HPPR_00551	K03722	dinG; ATP-dependent DNA helicase DinG [EC:5.6.2.3]	179		
HPPR_00552	K02065	mlaF, linL, mkl; phospholipid/cholesterol/gamma-HCH transport system ATP-binding	108		
HPPR_00553	K02066	mlaE, linK; phospholipid/cholesterol/gamma-HCH transport system permease prote	190		
HPPR_00554			28		
HPPR_00555	K04755	fdx; ferredoxin, 2Fe-2S	34	K22071	11
HPPR_00556	K04044	hscA; molecular chaperone HscA	244		
HPPR_00557	K04082	hscB, HSCB, HSC20; molecular chaperone HscB	22		
HPPR_00558	K13628	iscA; iron-sulfur cluster assembly protein	68		
HPPR_00559	K04488	iscU, nifU; nitrogen fixation protein NifU and related proteins	95		
HPPR_00560	K04487	iscS, NFS1; cysteine desulfurase [EC:2.8.1.7]	250		
HPPR_00561	K13643	iscR; Rrf2 family transcriptional regulator, iron-sulfur cluster assembly transcription	57		
HPPR_00562	K07018	K07018; uncharacterized protein	86		
HPPR_00563	K01866	YARS, tyrS; tyrosyl-tRNA synthetase [EC:6.1.1.1]	210		
HPPR_00564			3		
HPPR_00565	K02836	prfB; peptide chain release factor 2	144		
HPPR_00566	K05366	mrcA; penicillin-binding protein 1A [EC:2.4.1.129 3.4.16.4]	230		
HPPR_00567	K01448	amiABC; N-acetylmuramoyl-L-alanine amidase [EC:3.5.1.28]	62		
HPPR_00568	K08300	rne; ribonuclease E [EC:3.1.26.12]	190		
HPPR_00569			83		
HPPR_00570			2	K03286	2
HPPR_00572	K02434	gatB, PET112; aspartyl-tRNA(Asn)/glutamyl-tRNA(Gln) amidotransferase subunit B [	276		
HPPR_00573	K02433	gatA, QRSL1; aspartyl-tRNA(Asn)/glutamyl-tRNA(Gln) amidotransferase subunit A [E	310		
HPPR_00574	K02435	gatC, GATC; aspartyl-tRNA(Asn)/glutamyl-tRNA(Gln) amidotransferase subunit C [EC	27		
HPPR_00575	K07447	ruvX; putative pre-16S rRNA nuclease [EC:3.1.-.-]	61		
HPPR_00576	K08591	plsY; acyl phosphate:glycerol-3-phosphate acyltransferase [EC:2.3.1.275]	98		
HPPR_00577	K04096	smf; DNA processing protein	135		
HPPR_00578	K03168	topA; DNA topoisomerase I [EC:5.6.2.1]	338		
HPPR_00579	K12573	rnr, vacB; ribonuclease R [EC:3.1.13.1]	103		
HPPR_00580	K02913	RP-L33, MRPL33, rpmG; large subunit ribosomal protein L33	52		
HPPR_00581			5	K06919	1
HPPR_00582			1	K01091	1
HPPR_00583	K06180	rluD; 23S rRNA pseudouridine1911/1915/1917 synthase [EC:5.4.99.23]	102		
HPPR_00584			2	K03973	1
HPPR_00585			20		
HPPR_00586	K08303	prtC, trhP; U32 family peptidase [EC:3.4.-.-]	357		
HPPR_00587	K01493	comEB; dCMP deaminase [EC:3.5.4.12]	76		
HPPR_00588			4	K00001	1
HPPR_00589	K03469	rnhA, RNASEH1; ribonuclease HI [EC:3.1.26.4]	89		
HPPR_00590	K07735	algH; putative transcriptional regulator	47		
HPPR_00591					
HPPR_00592			29		
HPPR_00593	K03215	rumA; 23S rRNA (uracil1939-C5)-methyltransferase [EC:2.1.1.190]	78		
HPPR_00594			139		
HPPR_00595	K01972	E6.5.1.2, ligA, ligB; DNA ligase (NAD+) [EC:6.5.1.2]	201		
HPPR_00596	K03631	recN; DNA repair protein RecN (Recombination protein N)	179		
HPPR_00597	K05807	bamD; outer membrane protein assembly factor BamD	59		
HPPR_00598	K02535	lpxC; UDP-3-O-[3-hydroxymyristoyl] N-acetylglucosamine deacetylase [EC:3.5.1.108	175		
HPPR_00599			60		
HPPR_00600	K03531	ftsZ; cell division protein FtsZ	93		
HPPR_00601					
HPPR_00602	K03589	ftsQ; cell division protein FtsQ	20		
HPPR_00603	K01921	ddl; D-alanine-D-alanine ligase [EC:6.3.2.4]	116		
HPPR_00604	K00075	murB; UDP-N-acetylmuramate dehydrogenase [EC:1.3.1.98]	121		

HPPR_00605	K01924	murC; UDP-N-acetylmuramate--alanine ligase [EC:6.3.2.8]	198		
HPPR_00606	K02563	murG; UDP-N-acetylglucosamine--N-acetylmuramyl-(pentapeptide) pyrophosphoryl	76		
HPPR_00607	K03588	ftsW, spoVE; cell division protein FtsW	170		
HPPR_00608	K01925	murD; UDP-N-acetylmuramoylalanine--D-glutamate ligase [EC:6.3.2.9]	114		
HPPR_00609	K01000	mraY; phospho-N-acetylmuramoyl-pentapeptide-transferase [EC:2.7.8.13]	135		
HPPR_00610	K01929	murF; UDP-N-acetylmuramoyl-tripeptide--D-alanyl-D-alanine ligase [EC:6.3.2.10]	97	K15792	6
HPPR_00611	K01928	murE; UDP-N-acetylmuramoyl-L-alanyl-D-glutamate--2,6-diaminopimelate ligase [E(	135	K15792	8
HPPR_00612	K03587	ftsI; cell division protein FtsI (penicillin-binding protein 3) [EC:3.4.16.4]	147		
HPPR_00613			7	K03695	1
HPPR_00614	K03438	mraW, rsmH; 16S rRNA (cytosine1402-N4)-methyltransferase [EC:2.1.1.199]	117		
HPPR_00618					
HPPR_00619			26	K03217	5
HPPR_00620			16	K16868	4
HPPR_00622					
HPPR_00623	K08301	rng, cafA; ribonuclease G [EC:3.1.26.-]	33		
HPPR_00624	K06287	yhdE; nucleoside triphosphate pyrophosphatase [EC:3.6.1.-]	89		
HPPR_00625	K02518	infA; translation initiation factor IF-1	25		
HPPR_00626	K00790	murA; UDP-N-acetylglucosamine 1-carboxyvinyltransferase [EC:2.5.1.7]	200		
HPPR_00627			9		
HPPR_00629	K03216	trmL, cspR; tRNA (cytidine/uridine-2'-O-)-methyltransferase [EC:2.1.1.207]	93		
HPPR_00630	K00791	miaA, TRIT1; tRNA dimethylallyltransferase [EC:2.5.1.75]	79		
HPPR_00631	K03569	mreB; rod shape-determining protein MreB and related proteins	198		
HPPR_00632	K03570	mreC; rod shape-determining protein MreC	26		
HPPR_00633					
HPPR_00634	K05515	mrdA; penicillin-binding protein 2 [EC:3.4.16.4]	188		
HPPR_00635	K05837	rodA, mrdB; rod shape determining protein RodA	183		
HPPR_00636	K07263	pqqL; zinc protease [EC:3.4.24.-]	55		
HPPR_00637	K07263	pqqL; zinc protease [EC:3.4.24.-]	94		
HPPR_00638			9	K01920	1
HPPR_00639	K03101	lspA; signal peptidase II [EC:3.4.23.36]	46		
HPPR_00640	K01870	IARS, ileS; isoleucyl-tRNA synthetase [EC:6.1.1.5]	425		
HPPR_00641	K11753	ribF; riboflavin kinase / FMN adenylyltransferase [EC:2.7.1.26 2.7.7.2]	94		
HPPR_00642					
HPPR_00643	K06020	ettA; energy-dependent translational throttle protein EttA	314		
HPPR_00644			1		
HPPR_00645			69		
HPPR_00646					
HPPR_00647			14	K17278	1
HPPR_00648	K06956	K06956; uncharacterized protein	233		
HPPR_00649			121	K18367	32
HPPR_00650			61		
HPPR_00651	K00384	trxB, TRR; thioredoxin reductase (NADPH) [EC:1.8.1.9]	160		
HPPR_00652	K02114	ATPF1E, atpC; F-type H+-transporting ATPase subunit epsilon	16		
HPPR_00653	K02112	ATPF1B, atpD; F-type H+/Na+-transporting ATPase subunit beta [EC:7.1.2.2 7.2.2.1]	326		
HPPR_00654	K02115	ATPF1G, atpG; F-type H+-transporting ATPase subunit gamma	57		
HPPR_00655	K02111	ATPF1A, atpA; F-type H+/Na+-transporting ATPase subunit alpha [EC:7.1.2.2 7.2.2.1]	312		
HPPR_00656	K02113	ATPF1D, atpH; F-type H+-transporting ATPase subunit delta	37		
HPPR_00657	K04066	priA; primosomal protein N' (replication factor Y) (superfamily II helicase) [EC:5.6.2.4	277		
HPPR_00658	K03733	xerC; integrase/recombinase XerC	110		
HPPR_00659			12	K17837	4
HPPR_00660					
HPPR_00661	K00426	cydB; cytochrome bd ubiquinol oxidase subunit II [EC:7.1.1.7]	184		
HPPR_00662	K00425	cydA; cytochrome bd ubiquinol oxidase subunit I [EC:7.1.1.7]	250		
HPPR_00663			4	K20344	1
HPPR_00664	K00133	asd; aspartate-semialdehyde dehydrogenase [EC:1.2.1.11]	153		
HPPR_00665	K02884	RP-L19, MRPL19, rplS; large subunit ribosomal protein L19	83		
HPPR_00666	K00554	trmD; tRNA (guanine37-N1)-methyltransferase [EC:2.1.1.228]	136		
HPPR_00667	K02860	rimM; 16S rRNA processing protein RimM	48		
HPPR_00668	K02959	RP-S16, MRPS16, rpsP; small subunit ribosomal protein S16	29		
HPPR_00669	K03106	SRP54, ffh; signal recognition particle subunit SRP54 [EC:3.6.5.4]	175		
HPPR_00670	K01778	dapF; diaminopimelate epimerase [EC:5.1.1.7]	45		
HPPR_00671	K18707	mtaB; threonylcarbamoyladenosine tRNA methylthiotransferase MtaB [EC:2.8.4.5]	166		
HPPR_00672			14		
HPPR_00673			54	K08217	5
HPPR_00674	K13529	ada-alkA; AraC family transcriptional regulator, regulatory protein of adaptative res	14	K01247	7

HPPR_00675	K01854	glf; UDP-galactopyranose mutase [EC:5.4.99.9]	152		
HPPR_00676	K03110	ftsY; fused signal recognition particle receptor	90		
HPPR_00677	K03559	exbD; biopolymer transport protein ExbD	19		
HPPR_00678	K03561	exbB; biopolymer transport protein ExbB	20		
HPPR_00679					
HPPR_00680	K16087	TC.FEV.OM3, tbpA, hemR, lbpA, hpuB, bhuR, hugA, hmbR; hemoglobin/transferrin/l	104		
HPPR_00681			14	K07121	1
HPPR_00682					
HPPR_00683					
HPPR_00684	K01591	pyrF; orotidine-5'-phosphate decarboxylase [EC:4.1.1.23]	54		
HPPR_00685	K17828	pyrDI; dihydroorotate dehydrogenase (NAD+) catalytic subunit [EC:1.3.1.14]	24	K00254	6
HPPR_00686	K09951	cas2; CRISPR-associated protein Cas2	38		
HPPR_00687	K15342	cas1; CRISP-associated protein Cas1	61		
HPPR_00688	K09952	csn1, cas9; CRISPR-associated endonuclease Csn1 [EC:3.1.-.-]	37		
HPPR_00690			15	K03279	1
HPPR_00691					
HPPR_00693			5	K03668	5
HPPR_00694	K01992	ABC-2.P; ABC-2 type transport system permease protein	101		
HPPR_00695	K03565	recX; regulatory protein	21		
HPPR_00696			28		
HPPR_00697	K00919	ispE; 4-diphosphocytidyl-2-C-methyl-D-erythritol kinase [EC:2.7.1.148]	82		
HPPR_00698			74		
HPPR_00699	K21929	udg; uracil-DNA glycosylase [EC:3.2.2.27]	60		
HPPR_00700	K08309	slt; soluble lytic murein transglycosylase [EC:4.2.2.-]	61		
HPPR_00701			2	K24846	1
HPPR_00702			14	K03611	4
HPPR_00703					
HPPR_00704					
HPPR_00705			75		
HPPR_00706	K00927	PGK, pgk; phosphoglycerate kinase [EC:2.7.2.3]	264		
HPPR_00707	K01174	nuc; micrococcal nuclease [EC:3.1.31.1]	26		
HPPR_00708					
HPPR_00709			58		
HPPR_00710			69		
HPPR_00711			13	K00703	1
HPPR_00712			33		
HPPR_00713	K02116	atpI; ATP synthase protein I	18		
HPPR_00714	K02108	ATPF0A, atpB; F-type H+-transporting ATPase subunit a	137		
HPPR_00715					
HPPR_00716	K02109	ATPF0B, atpF; F-type H+-transporting ATPase subunit b	12		
HPPR_00717	K02109	ATPF0B, atpF; F-type H+-transporting ATPase subunit b	14		
HPPR_00718	K01736	aroC; chorismate synthase [EC:4.2.3.5]	222		
HPPR_00719	K12542	lapC; membrane fusion protein, adhesin transport system	131		
HPPR_00720	K12541	lapB; ATP-binding cassette, subfamily C, bacterial LapB	169		
HPPR_00721					
HPPR_00722			3	K21449	1
HPPR_00723	K12543	lapE; outer membrane protein, adhesin transport system	31	K12340	1
HPPR_00724					
HPPR_00725			2		
HPPR_00726					
HPPR_00727	K00769	gpt; xanthine phosphoribosyltransferase [EC:2.4.2.22]	53		
HPPR_00730	K02238	comEC; competence protein ComEC	71		
HPPR_00732	K06911	PIR; quercetin 2,3-dioxygenase [EC:1.13.11.24]	106		
HPPR_00733	K05592	deaD, cshA; ATP-dependent RNA helicase DeaD [EC:3.6.4.13]	64	K11927	32
HPPR_00734			7	K22205	2
HPPR_00735	K09760	rmuC; DNA recombination protein RmuC	111		
HPPR_00736	K04750	phnB; PhnB protein	41		
HPPR_00737	K04750	phnB; PhnB protein	21		
HPPR_00738			2	K04080	1
HPPR_00739	K04080	ibpA; molecular chaperone IbpA	22		
HPPR_00740	K04758	feoA; ferrous iron transport protein A	16		
HPPR_00741	K04759	feoB; ferrous iron transport protein B	186		
HPPR_00743	K03431	glmM; phosphoglucosamine mutase [EC:5.4.2.10]	238		
HPPR_00744	K03798	ftsH, hflB; cell division protease FtsH [EC:3.4.24.-]	287		
HPPR_00745	K04075	tilS, mesJ; tRNA(Ile)-lysidine synthase [EC:6.3.4.19]	60		

HPPR_00746			2	K20480	1
HPPR_00747	K03640	pal; peptidoglycan-associated lipoprotein	47	K03286	1
HPPR_00748	K03641	tolB; TolB protein	77		
HPPR_00749			3	K06919	1
HPPR_00750	K03560	tolR; biopolymer transport protein TolR	53		
HPPR_00751	K03562	tolQ; biopolymer transport protein TolQ	55		
HPPR_00752	K07107	ybgC; acyl-CoA thioester hydrolase [EC:3.1.2.-]	30	K01075	3
HPPR_00753	K03551	ruvB; holliday junction DNA helicase RuvB [EC:5.6.2.4]	196		
HPPR_00754	K03550	ruvA; holliday junction DNA helicase RuvA [EC:5.6.2.4]	60		
HPPR_00755	K01159	ruvC; crossover junction endodeoxyribonuclease RuvC [EC:3.1.21.10]	64		
HPPR_00756			131		
HPPR_00757			22		
HPPR_00758	K01934	MTHFS; 5-formyltetrahydrofolate cyclo-ligase [EC:6.3.3.2]	62		
HPPR_00759	K09888	zapA; cell division protein ZapA	10		
HPPR_00760			1	K01858	1
HPPR_00761	K00134	GAPDH, gapA; glyceraldehyde 3-phosphate dehydrogenase (phosphorylating) [EC:1.	234		
HPPR_00762			3	K06954	1
HPPR_00763	K02356	efp; elongation factor P	111		
HPPR_00764	K01092	E3.1.3.25, IMPA, suhB; myo-inositol-1(or 4)-monophosphatase [EC:3.1.3.25]	45		
HPPR_00765			1	K04041	1
HPPR_00766	K08151	tetA; MFS transporter, DHA1 family, tetracycline resistance protein	38		
HPPR_00767	K02909	RP-L31, rpmE; large subunit ribosomal protein L31	47		
HPPR_00768	K08151	tetA; MFS transporter, DHA1 family, tetracycline resistance protein	32	K08153	17
HPPR_00769	K08151	tetA; MFS transporter, DHA1 family, tetracycline resistance protein	26	K08153	16
HPPR_00770	K09987	K09987; uncharacterized protein	44		
HPPR_00771	K21071	pfk, pfp; ATP-dependent phosphofructokinase / diphosphate-dependent phosphofr	187		
HPPR_00772	K02970	RP-S21, MRPS21, rpsU; small subunit ribosomal protein S21	47		
HPPR_00773			33	K08153	1
HPPR_00774	K03797	E3.4.21.102, prc, ctpA; carboxyl-terminal processing protease [EC:3.4.21.102]	62		
HPPR_00775	K00275	pdxH, PNPO; pyridoxamine 5'-phosphate oxidase [EC:1.4.3.5]	74		
HPPR_00776			2	K01991	1
HPPR_00777	K00208	fabI; enoyl-[acyl-carrier protein] reductase I [EC:1.3.1.9 1.3.1.10]	161		
HPPR_00778	K04771	degP, htrA; serine protease Do [EC:3.4.21.107]	123		
HPPR_00779	K03593	mrp, NUBPL; ATP-binding protein involved in chromosome partitioning	77		
HPPR_00780			291		
HPPR_00781	K11085	msbA; ATP-binding cassette, subfamily B, bacterial MsbA [EC:7.5.2.6]	198		
HPPR_00782			42	K02527	1
HPPR_00783	K02527	kdtA, waaA; 3-deoxy-D-manno-octulosonic-acid transferase [EC:2.4.99.12 2.4.99.13	85		
HPPR_00784	K00912	lpxK; tetraacyldisaccharide 4'-kinase [EC:2.7.1.130]	108		
HPPR_00785	K02517	lpxL, htrB; Kdo2-lipid IVA lauroyltransferase/acyltransferase [EC:2.3.1.241 2.3.1.-]	72		
HPPR_00786	K03601	xseA; exodeoxyribonuclease VII large subunit [EC:3.1.11.6]	91		
HPPR_00787	K01810	GPI, pgi; glucose-6-phosphate isomerase [EC:5.3.1.9]	252		
HPPR_00788	K03572	mutL; DNA mismatch repair protein MutL	219		
HPPR_00789	K06890	K06890; uncharacterized protein	112		
HPPR_00790	K06173	truA, PUS1; tRNA pseudouridine38-40 synthase [EC:5.4.99.12]	158		
HPPR_00791	K03466	ftsK, spoIIIE; DNA segregation ATPase FtsK/SpoIIIE, S-DNA-T family	181		
HPPR_00792			28		
HPPR_00793			10	K02483	2
HPPR_00794	K06997	yggS, PROSC; PLP dependent protein	70		
HPPR_00795			2	K02062	1
HPPR_00796			39		
HPPR_00797	K01869	LARS, leuS; leucyl-tRNA synthetase [EC:6.1.1.4]	430		
HPPR_00798					
HPPR_00799	K02340	holA; DNA polymerase III subunit delta [EC:2.7.7.7]	73		
HPPR_00800	K00798	MMAB, pduO; cob(I)alamin adenosyltransferase [EC:2.5.1.17]	47		
HPPR_00801	K01262	pepP; Xaa-Pro aminopeptidase [EC:3.4.11.9]	177		
HPPR_00802					
HPPR_00803	K09812	ftsE; cell division transport system ATP-binding protein	99		
HPPR_00804					
HPPR_00805	K00655	plsC; 1-acyl-sn-glycerol-3-phosphate acyltransferase [EC:2.3.1.51]	81		
HPPR_00806	K03465	thyX, thy1; thymidylate synthase (FAD) [EC:2.1.1.148]	210		
HPPR_00811	K09985	K09985; uncharacterized protein	31		
HPPR_00812			1	K01613	1
HPPR_00813					
HPPR_00814	K02919	RP-L36, MRPL36, rpmJ; large subunit ribosomal protein L36	41		

HPPR_00815	K03839	fldA, nifF, isiB; flavodoxin I	21		
HPPR_00816	K07568	queA; S-adenosylmethionine:tRNA ribosyltransferase-isomerase [EC:2.4.99.17]	166		
HPPR_00817	K00773	tgt; queuine tRNA-ribosyltransferase [EC:2.4.2.29]	280		
HPPR_00818	K18979	queG; epoxyqueuosine reductase [EC:1.17.99.6]	90		
HPPR_00819			3		
HPPR_00821	K01990	ABC-2.A; ABC-2 type transport system ATP-binding protein	132		
HPPR_00822			8	K03045	1
HPPR_00823	K02335	polA; DNA polymerase I [EC:2.7.7.7]	315		
HPPR_00824			15	K02242	1
HPPR_00825	K09791	K09791; uncharacterized protein	26		
HPPR_00827			8	K01715	1
HPPR_00828			35		
HPPR_00829	K01520	dut, DUT; dUTP pyrophosphatase [EC:3.6.1.23]	115		
HPPR_00830	K03688	ubiB, aarF; ubiquinone biosynthesis protein	124		
HPPR_00831	K03183	ubiE; demethylmenaquinone methyltransferase / 2-methoxy-6-polyprenyl-1,4-benz	124		
HPPR_00832	K10563	mutM, fpg; formamidopyrimidine-DNA glycosylase [EC:3.2.2.23 4.2.99.18]	108		
HPPR_00833	K02968	RP-S20, rpsT; small subunit ribosomal protein S20	42		
HPPR_00834	K02313	dnaA; chromosomal replication initiator protein	203		
HPPR_00835	K02338	dnaN; DNA polymerase III subunit beta [EC:2.7.7.7]	148		
HPPR_00836	K03629	recF; DNA replication and repair protein RecF	90		
HPPR_00837			167	K03327	18
HPPR_00838	K02470	gyrB; DNA gyrase subunit B [EC:5.6.2.2]	474		
HPPR_00839			4	K02073	1
HPPR_00840	K06861	lptB; lipopolysaccharide export system ATP-binding protein [EC:7.5.2.5]	140		
HPPR_00841					
HPPR_00842	K06041	kdsD, kpsF; arabinose-5-phosphate isomerase [EC:5.3.1.13]	148		
HPPR_00843	K03684	rnd; ribonuclease D [EC:3.1.13.5]	114		
HPPR_00844			2	K07071	1
HPPR_00845	K01626	E2.5.1.54, aroF, aroG, aroH; 3-deoxy-7-phosphoheptulonate synthase [EC:2.5.1.54]	141		
HPPR_00846	K00763	pncB, NAPRT1; nicotinate phosphoribosyltransferase [EC:6.3.4.21]	187		
HPPR_00847					
HPPR_00848	K01885	EARS, gltX; glutamyl-tRNA synthetase [EC:6.1.1.17]	196		
HPPR_00849	K01883	CARS, cysS; cysteinyl-tRNA synthetase [EC:6.1.1.16]	215		
HPPR_00850	K09160	K09160; uncharacterized protein	72		
HPPR_00851			49		
HPPR_00852	K03799	htpX; heat shock protein HtpX [EC:3.4.24.-]	133		
HPPR_00853			63		
HPPR_00854	K09780	K09780; uncharacterized protein	40		
HPPR_00855	K00057	gpsA; glycerol-3-phosphate dehydrogenase (NAD(P)+) [EC:1.1.1.94]	119		
HPPR_00856	K25706	tsaD; tRNA N6-adenosine threonylcarbamoyltransferase [EC:2.3.1.234]	161		
HPPR_00857					
HPPR_00858					
HPPR_00859			34		
HPPR_00860	K00459	ncd2, npd; nitronate monooxygenase [EC:1.13.12.16]	301		
HPPR_00861	K00962	pnp, PNPT1; polyribonucleotide nucleotidyltransferase [EC:2.7.7.8]	380		
HPPR_00862	K02956	RP-S15, MRPS15, rpsO; small subunit ribosomal protein S15	66		
HPPR_00863	K03177	truB, PUS4, TRUB1; tRNA pseudouridine55 synthase [EC:5.4.99.25]	102		
HPPR_00864	K02834	rbfA; ribosome-binding factor A	44		
HPPR_00865	K02519	infB, MTIF2; translation initiation factor IF-2	190		
HPPR_00866	K02600	nusA; transcription termination/antitermination protein NusA	197		
HPPR_00867	K09748	rimP; ribosome maturation factor RimP	36		
HPPR_00868	K03439	trmB, METTL1, TRM8; tRNA (guanine-N7-)-methyltransferase [EC:2.1.1.33]	58		
HPPR_00869	K00789	metK, MAT; S-adenosylmethionine synthetase [EC:2.5.1.6]	220		
HPPR_00870			38	K03820	1
HPPR_00871	K03820	lnt; apolipoprotein N-acyltransferase [EC:2.3.1.269]	74		
HPPR_00872	K06189	corC, tlyC; hemolysin (HlyC) family protein	77		
HPPR_00873	K07042	ybeY, yqfG; probable rRNA maturation factor	19	K03474	1
HPPR_00874	K06168	miaB; tRNA-2-methylthio-N6-dimethylallyladenosine synthase [EC:2.8.4.3]	238		
HPPR_00875	K14742	tsaB; tRNA threonylcarbamoyladenosine biosynthesis protein TsaB	22		
HPPR_00876	K03796	bax; Bax protein	42		
HPPR_00877			101		
HPPR_00878	K01867	WARS, trpS; tryptophanyl-tRNA synthetase [EC:6.1.1.2]	215		
HPPR_00879	K03980	murJ, mviN; putative peptidoglycan lipid II flippase	123		
HPPR_00880	K03555	mutS; DNA mismatch repair protein MutS	302		
HPPR_00881					

HPPR_00882			2	K07275	1
HPPR_00883			1	K07407	1
HPPR_00884	K01507	ppa; inorganic pyrophosphatase [EC:3.6.1.1]	100		
HPPR_00885					
HPPR_00886			2	K18889	1
HPPR_00887			28		
HPPR_00888			52		
HPPR_00889	K02342	dnaQ; DNA polymerase III subunit epsilon [EC:2.7.7.7]	82		
HPPR_00890	K00859	coaE; dephospho-CoA kinase [EC:2.7.1.24]	48		
HPPR_00891	K00014	aroE; shikimate dehydrogenase [EC:1.1.1.25]	32		
HPPR_00892	K03628	rho; transcription termination factor Rho	300		
HPPR_00893	K03650	mnmE, trmE, MSS1; tRNA modification GTPase [EC:3.6.-.-]	146		
HPPR_00894			12	K18697	1
HPPR_00895	K03495	gidA, mnmG, MTO1; tRNA uridine 5-carboxymethylaminomethyl modification enzyr	323		
HPPR_00896					
HPPR_00897	K03501	gidB, rsmG; 16S rRNA (guanine527-N7)-methyltransferase [EC:2.1.1.170]	10		
HPPR_00898	K03496	parA, soj; chromosome partitioning protein	145		
HPPR_00899	K03497	parB, spo0J; ParB family transcriptional regulator, chromosome partitioning protein	82		
HPPR_00900			2	K20459	1
HPPR_00901			1	K00648	1
HPPR_00902			9	K09121	1
HPPR_00903	K11991	tadA; tRNA(adenine34) deaminase [EC:3.5.4.33]	60		
HPPR_00904	K06178	rluB; 23S rRNA pseudouridine2605 synthase [EC:5.4.99.22]	56		
HPPR_00905	K08316	rsmD; 16S rRNA (guanine966-N2)-methyltransferase [EC:2.1.1.171]	56		
HPPR_00906			2	K00943	1
HPPR_00907	K02503	HINT1, hinT, hit; histidine triad (HIT) family protein	52		
HPPR_00908	K01419	hslV, clpQ; ATP-dependent HslUV protease, peptidase subunit HslV [EC:3.4.25.2]	110		
HPPR_00909	K03667	hslU; ATP-dependent HslUV protease ATP-binding subunit HslU	254		
HPPR_00910	K07391	comM; magnesium chelatase family protein	243		
HPPR_00911			15		
HPPR_00912	K02040	pstS; phosphate transport system substrate-binding protein	137		
HPPR_00913	K02037	pstC; phosphate transport system permease protein	169		
HPPR_00914	K02038	pstA; phosphate transport system permease protein	150		
HPPR_00915	K02036	pstB; phosphate transport system ATP-binding protein [EC:7.3.2.1]	167		
HPPR_00916	K02039	phoU; phosphate transport system protein	46		
HPPR_00917			1	K07275	1
HPPR_00918	K00625	E2.3.1.8, pta; phosphate acetyltransferase [EC:2.3.1.8]	106		
HPPR_00919	K00925	ackA; acetate kinase [EC:2.7.2.1]	241		
HPPR_00920			16		
HPPR_00922	K01784	galE, GALE; UDP-glucose 4-epimerase [EC:5.1.3.2]	75	K02473	9
HPPR_00923	K01784	galE, GALE; UDP-glucose 4-epimerase [EC:5.1.3.2]	134	K12448	31
HPPR_00924			21	K03646	1
HPPR_00925	K10773	NTH; endonuclease III [EC:4.2.99.18]	106		
HPPR_00926	K03686	dnaJ; molecular chaperone DnaJ	146		
HPPR_00927	K04043	dnaK, HSPA9; molecular chaperone DnaK	435		
HPPR_00928	K03687	GRPE; molecular chaperone GrpE	42		
HPPR_00929	K01295	cpg; glutamate carboxypeptidase [EC:3.4.17.11]	51		
HPPR_00930	K00989	rph; ribonuclease PH [EC:2.7.7.56]	186		
HPPR_00931	K01519	rdgB, ITPA; XTP/dITP diphosphohydrolase [EC:3.6.1.66]	101		
HPPR_00932			129		
HPPR_00933			38	K01999	1
HPPR_00934	K07056	rsmI; 16S rRNA (cytidine1402-2'-O)-methyltransferase [EC:2.1.1.198]	108		
HPPR_00935	K07460	yraN; putative endonuclease	29		
HPPR_00936	K01920	gshB; glutathione synthase [EC:6.3.2.3]	150		
HPPR_00937	K00604	MTFMT, fmt; methionyl-tRNA formyltransferase [EC:2.1.2.9]	124		
HPPR_00938	K01462	PDF, def; peptide deformylase [EC:3.5.1.88]	100		
HPPR_00939	K06187	recR; recombination protein RecR	87		
HPPR_00940	K09747	ebfC; nucleoid-associated protein EbfC	47		
HPPR_00941	K09125	yhhQ; queuosine precursor transporter	20		
HPPR_00942	K23993	fruB; multiphosphoryl transfer protein [EC:2.7.3.9 2.7.1.202]	258		
HPPR_00943	K02804	nagE; N-acetylglucosamine PTS system EIICBA or EIICB component [EC:2.7.1.193]	217		
HPPR_00944	K02564	nagB, GNPDA; glucosamine-6-phosphate deaminase [EC:3.5.99.6]	140		
HPPR_00945	K01443	nagA, AMDHD2; N-acetylglucosamine-6-phosphate deacetylase [EC:3.5.1.25]	215		
HPPR_00946	K07501	K07501; 3'-5' exonuclease	125		
HPPR_00947	K01895	ACSS1_2, acs; acetyl-CoA synthetase [EC:6.2.1.1]	314		

HPPR_00948			28	K03829	1
HPPR_00949	K02343	dnaX; DNA polymerase III subunit gamma/tau [EC:2.7.7.7]	121		
HPPR_00951			5	K01174	1
HPPR_00952	K06941	rlmN; 23S rRNA (adenine2503-C2)-methyltransferase [EC:2.1.1.192]	162		
HPPR_00953	K00979	kdsB; 3-deoxy-manno-octulosonate cytidylyltransferase (CMP-KDO synthetase) [EC:	121		
HPPR_00954	K13893	yejA; microcin C transport system substrate-binding protein	82		
HPPR_00955	K13894	yejB; microcin C transport system permease protein	90		
HPPR_00956	K13895	yejE; microcin C transport system permease protein	65	K13895/K13896	2
HPPR_00957	K02031	ddpD; peptide/nickel transport system ATP-binding protein	48	K13896	8
	K02032	ddpF; peptide/nickel transport system ATP-binding protein			
HPPR_00958	K06153	bacA; undecaprenyl-diphosphatase [EC:3.6.1.27]	101		
HPPR_00959	K14170	pheA; chorismate mutase / prephenate dehydratase [EC:5.4.99.5 4.2.1.51]	12		
HPPR_00960	K01069	gloB, gloC, HAGH; hydroxyacylglutathione hydrolase [EC:3.1.2.6]	76		
HPPR_00961	K03784	deoD; purine-nucleoside phosphorylase [EC:2.4.2.1]	141		
HPPR_00962			36	K09809	2
HPPR_00963	K01839	deoB; phosphopentomutase [EC:5.4.2.7]	220		
HPPR_00964			247		
HPPR_00965	K01868	TARS, thrS; threonyl-tRNA synthetase [EC:6.1.1.3]	353		
HPPR_00966	K02520	infC, MTIF3; translation initiation factor IF-3	72		
HPPR_00967	K02916	RP-L35, MRPL35, rpmI; large subunit ribosomal protein L35	38		
HPPR_00968	K02887	RP-L20, MRPL20, rplT; large subunit ribosomal protein L20	79		
HPPR_00969	K01889	FARSA, pheS; phenylalanyl-tRNA synthetase alpha chain [EC:6.1.1.20]	171		
HPPR_00970	K01890	FARSB, pheT; phenylalanyl-tRNA synthetase beta chain [EC:6.1.1.20]	260		
HPPR_00971			7	K00784	1
HPPR_00972	K01243	mtnN, mtn, pfs; adenosylhomocysteine nucleosidase [EC:3.2.2.9]	71		
HPPR_00973	K01183	E3.2.1.14; chitinase [EC:3.2.1.14]	101		
HPPR_00974	K01183	E3.2.1.14; chitinase [EC:3.2.1.14]	11		
HPPR_00975	K04077	groEL, HSPD1; chaperonin GroEL	408		
HPPR_00976	K04078	groES, HSPE1; chaperonin GroES	86		
HPPR_00977			151	K06158	9
HPPR_00978	K03657	uvrD, pcrA; ATP-dependent DNA helicase UvrD/PcrA [EC:5.6.2.4]	292		
HPPR_00979	K00525	E1.17.4.1A, nrdA, nrdE; ribonucleoside-diphosphate reductase alpha chain [EC:1.17.	347		
HPPR_00980	K00526	E1.17.4.1B, nrdB, nrdF; ribonucleoside-diphosphate reductase beta chain [EC:1.17.4	185		
HPPR_00981	K03647	nrdI; protein involved in ribonucleotide reduction	32		
HPPR_00982	K02687	prmA; ribosomal protein L11 methyltransferase [EC:2.1.1.-]	34		
HPPR_00983	K08281	pncA; nicotinamidase/pyrazinamidase [EC:3.5.1.19 3.5.1.-]	111		
HPPR_00984	K03116	tatA; sec-independent protein translocase protein TatA	19		
HPPR_00985	K04042	glmU; bifunctional UDP-N-acetylglucosamine pyrophosphorylase / glucosamine-1-p	177		
HPPR_00986					
HPPR_00987			2		
HPPR_00988	K03470	rnhB; ribonuclease HII [EC:3.1.26.4]	94		
HPPR_00989	K01129	dgt; dGTPase [EC:3.1.5.1]	156		
HPPR_00990			15	K03275	3
HPPR_00991	K16264	czcD, zitB; cobalt-zinc-cadmium efflux system protein	86		
HPPR_00992	K03596	lepA; GTP-binding protein LepA	436		
HPPR_00993	K01835	pgm; phosphoglucomutase [EC:5.4.2.2]	229		
HPPR_00994	K01491	folD; methylenetetrahydrofolate dehydrogenase (NADP+) / methenyltetrahydrofola	118		
HPPR_00995	K02221	yggT; YggT family protein	15		
HPPR_00996	K00942	gmk, GUK1; guanylate kinase [EC:2.7.4.8]	87		
HPPR_00997	K02528	ksgA; 16S rRNA (adenine1518-N6/adenine1519-N6)-dimethyltransferase [EC:2.1.1.1	81		
HPPR_00998	K00097	pdxA; 4-hydroxythreonine-4-phosphate dehydrogenase [EC:1.1.1.262]	102		
HPPR_00999	K03771	surA; peptidyl-prolyl cis-trans isomerase SurA [EC:5.2.1.8]	24		
HPPR_01000	K04744	lptD, imp, ostA; LPS-assembly protein	50		
HPPR_01001	K11720	lptG; lipopolysaccharide export system permease protein	72		
HPPR_01002			170		
HPPR_01003	K07566	tsaC, rimN, SUA5, YRDC; L-threonylcarbamoyladenylate synthase [EC:2.7.7.87]	110		
HPPR_01004	K01082	cysQ, MET22, BPNT1; 3'(2'), 5'-bisphosphate nucleotidase [EC:3.1.3.7]	75		
HPPR_01005	K01255	CARP, pepA; leucyl aminopeptidase [EC:3.4.11.1]	175		
HPPR_01006	K02339	holC; DNA polymerase III subunit chi [EC:2.7.7.7]	43		
HPPR_01007			4	K13481	1
HPPR_01008			41		
HPPR_01009					
HPPR_01010			11	K16148	1
HPPR_01011			30	K19427	3
HPPR_01012	K13581	ccrM; modification methylase [EC:2.1.1.72]	178		

HPPR_01013			142		
HPPR_01014			21	K19591	1
HPPR_01015	K09859	K09859; uncharacterized protein	36		
HPPR_01016	K07337	K07337; penicillin-binding protein activator	40		
HPPR_01017			5	K19802	1
HPPR_01018					
HPPR_01019	K06905	K06905; uncharacterized protein	20		
HPPR_01020			2	K01091	1
HPPR_01021					
HPPR_01022	K06903	K06903; uncharacterized protein	16		
HPPR_01023					
HPPR_01024			11	K01875	1
HPPR_01025	K06908	K06908; uncharacterized protein	57		
HPPR_01026	K06907	K06907; uncharacterized protein	107		
HPPR_01027			9	K19171	1
HPPR_01028			42		
HPPR_01029			82		
HPPR_01030					
HPPR_01031			5	K00088	1
HPPR_01032			7	K03526	1
HPPR_01033			14	K03279	2
HPPR_01035	K24845	ubiV; O2-independent ubiquinone biosynthesis protein UbiV	158		
HPPR_01036	K24844	ubiU; O2-independent ubiquinone biosynthesis protein UbiU	187		
HPPR_01037	K24843	ubiT; O2-independent ubiquinone biosynthesis accessory factor UbiT	14	K03690	1
HPPR_01038					
HPPR_01040			2	K00128	1
HPPR_01041	K02914	RP-L34, MRPL34, rpmH; large subunit ribosomal protein L34	36		
HPPR_01042	K03536	rnpA; ribonuclease P protein component [EC:3.1.26.5]	22		
HPPR_01043	K03217	yidC, spoIIIJ, OXA1, ccfA; YidC/Oxa1 family membrane protein insertase	224		
HPPR_01044	K03978	engB; GTP-binding protein	83		
HPPR_01045	K03299	TC.GNTP; gluconate:H+ symporter, GntP family	163		
HPPR_01046			55		
HPPR_01047	K13292	lgt, umpA; phosphatidylglycerol---prolipoprotein diacylglyceryl transferase [EC:2.5.1	140		
HPPR_01048	K18164	NDUFAF7; NADH dehydrogenase [ubiquinone] 1 alpha subcomplex assembly factor	48		
HPPR_01049	K06942	ychF; ribosome-binding ATPase	226		
HPPR_01050	K01056	PTH1, pth, spoVC; peptidyl-tRNA hydrolase, PTH1 family [EC:3.1.1.29]	80		
HPPR_01051	K02897	RP-L25, rplY; large subunit ribosomal protein L25	73		
HPPR_01052	K00948	PRPS, prsA; ribose-phosphate pyrophosphokinase [EC:2.7.6.1]	194		
HPPR_01053	K05810	LACC1, yfiH; purine-nucleoside/S-methyl-5'-thioadenosine phosphorylase / adenosi	121		
HPPR_01054	K07638	envZ; two-component system, OmpR family, osmolarity sensor histidine kinase Env	97		
HPPR_01055	K07659	ompR; two-component system, OmpR family, phosphate regulon response regulato	64		
HPPR_01056			102		
HPPR_01057	K08744	CRLS; cardiolipin synthase (CMP-forming) [EC:2.7.8.41]	72	K00995	5
HPPR_01058	K03703	uvrC; excinuclease ABC subunit C	221		
HPPR_01059	K08311	nudH; putative (di)nucleoside polyphosphate hydrolase [EC:3.6.1.-]	83		
HPPR_01060	K03797	E3.4.21.102, prc, ctpA; carboxyl-terminal processing protease [EC:3.4.21.102]	102		
HPPR_01061	K22719	envC; murein hydrolase activator	14		
HPPR_01062	K15633	gpmI; 2,3-bisphosphoglycerate-independent phosphoglycerate mutase [EC:5.4.2.12	253		
HPPR_01063	K00783	rlmH; 23S rRNA (pseudouridine1915-N3)-methyltransferase [EC:2.1.1.177]	65		
HPPR_01064	K09710	ybeB; ribosome-associated protein	37		
HPPR_01065	K00969	nadD; nicotinate-nucleotide adenylyltransferase [EC:2.7.7.18]	70		
HPPR_01066					
HPPR_01067	K03979	obgE, cgtA, MTG2; GTPase [EC:3.6.5.-]	145		
HPPR_01068	K02899	RP-L27, MRPL27, rpmA; large subunit ribosomal protein L27	72		
HPPR_01069	K02888	RP-L21, MRPL21, rplU; large subunit ribosomal protein L21	55		
HPPR_01070	K01295	cpg; glutamate carboxypeptidase [EC:3.4.17.11]	127		
HPPR_01071			9	K09815	1
HPPR_01072			9	K03597	1
HPPR_01073			9	K06720	1
HPPR_01074					
HPPR_01077	K00800	aroA; 3-phosphoshikimate 1-carboxyvinyltransferase [EC:2.5.1.19]	98	K24018	35
HPPR_01078	K00945	cmk; CMP/dCMP kinase [EC:2.7.4.25]	73		
HPPR_01079	K02945	RP-S1, rpsA; small subunit ribosomal protein S1	256		
HPPR_01080	K01963	accD; acetyl-CoA carboxylase carboxyl transferase subunit beta [EC:6.4.1.2 2.1.3.15]	147		
HPPR_01081	K11754	folC; dihydrofolate synthase / folylpolyglutamate synthase [EC:6.3.2.12 6.3.2.17]	118		

HPPR_01082	K03671	trxA; thioredoxin 1	52		
HPPR_01083	K16898	addA; ATP-dependent helicase/nuclease subunit A [EC:5.6.2.4 3.1.-.-]	258		
HPPR_01084	K16899	addB; ATP-dependent helicase/nuclease subunit B [EC:5.6.2.4 3.1.-.-]	220		
HPPR_01085	K06925	tsaE; tRNA threonylcarbamoyladenosine biosynthesis protein TsaE	32	K07102	1
HPPR_01086	K00384	trxB, TRR; thioredoxin reductase (NADPH) [EC:1.8.1.9]	158		
HPPR_01087	K03498	trkH, trkG, ktrB, ktrD; trk/ktr system potassium uptake protein	219		
HPPR_01088			1	K02027	1
HPPR_01089			1	K21449	1
HPPR_01090			2	K01256	1
HPPR_01091					
HPPR_01092			7	K02108	1
HPPR_01094			80		
HPPR_01095	K00567	ogt, MGMT; methylated-DNA-[protein]-cysteine S-methyltransferase [EC:2.1.1.63]	26		
HPPR_01096	K03322	mntH; manganese transport protein	288		
HPPR_01097			20	K01356	2
HPPR_01098			2	K00059	1
HPPR_01099			12	K00927	1
HPPR_01100			25	K02081	16
HPPR_01101	K01424	E3.5.1.1, ansA, ansB; L-asparaginase [EC:3.5.1.1]	112		
HPPR_01102	K03218	rlmB; 23S rRNA (guanosine2251-2'-O)-methyltransferase [EC:2.1.1.185]	73		
HPPR_01105	K02358	tuf, TUFM; elongation factor Tu	331		
HPPR_01107	K03073	secE; preprotein translocase subunit SecE	24		
HPPR_01108	K02601	nusG; transcription termination/antitermination protein NusG	77		
HPPR_01109	K02867	RP-L11, MRPL11, rplK; large subunit ribosomal protein L11	91		
HPPR_01110	K02863	RP-L1, MRPL1, rplA; large subunit ribosomal protein L1	132		
HPPR_01111	K02864	RP-L10, MRPL10, rplJ; large subunit ribosomal protein L10	76		
HPPR_01112	K02935	RP-L7, MRPL12, rplL; large subunit ribosomal protein L7/L12	66		
HPPR_01113	K03043	rpoB; DNA-directed RNA polymerase subunit beta [EC:2.7.7.6]	784		
HPPR_01114	K03046	rpoC; DNA-directed RNA polymerase subunit beta' [EC:2.7.7.6]	750	K13797	43
HPPR_01115	K02950	RP-S12, MRPS12, rpsL; small subunit ribosomal protein S12	142		
HPPR_01116	K02992	RP-S7, MRPS7, rpsG; small subunit ribosomal protein S7	117		
HPPR_01117	K02355	fusA, GFM, EFG; elongation factor G	525		
HPPR_01119	K02358	tuf, TUFM; elongation factor Tu	331		
HPPR_01120	K02946	RP-S10, MRPS10, rpsJ; small subunit ribosomal protein S10	110		
HPPR_01121	K02906	RP-L3, MRPL3, rplC; large subunit ribosomal protein L3	184		
HPPR_01122	K02926	RP-L4, MRPL4, rplD; large subunit ribosomal protein L4	121	K02906	4
HPPR_01123	K02892	RP-L23, MRPL23, rplW; large subunit ribosomal protein L23	48	K02906	1
HPPR_01124	K02886	RP-L2, MRPL2, rplB; large subunit ribosomal protein L2	189		
HPPR_01125	K02965	RP-S19, rpsS; small subunit ribosomal protein S19	95		
HPPR_01126	K02890	RP-L22, MRPL22, rplV; large subunit ribosomal protein L22	73		
HPPR_01127	K02982	RP-S3, rpsC; small subunit ribosomal protein S3	144		
HPPR_01128	K02878	RP-L16, MRPL16, rplP; large subunit ribosomal protein L16	148		
HPPR_01129	K02904	RP-L29, rpmC; large subunit ribosomal protein L29	29		
HPPR_01130	K02961	RP-S17, MRPS17, rpsQ; small subunit ribosomal protein S17	43		
HPPR_01131	K02874	RP-L14, MRPL14, rplN; large subunit ribosomal protein L14	138		
HPPR_01132	K02895	RP-L24, MRPL24, rplX; large subunit ribosomal protein L24	55		
HPPR_01133	K02931	RP-L5, MRPL5, rplE; large subunit ribosomal protein L5	118		
HPPR_01134	K02954	RP-S14, MRPS14, rpsN; small subunit ribosomal protein S14	75		
HPPR_01135	K02994	RP-S8, rpsH; small subunit ribosomal protein S8	65		
HPPR_01136	K02933	RP-L6, MRPL6, rplF; large subunit ribosomal protein L6	136		
HPPR_01137	K02881	RP-L18, MRPL18, rplR; large subunit ribosomal protein L18	58		
HPPR_01138	K02988	RP-S5, MRPS5, rpsE; small subunit ribosomal protein S5	109		
HPPR_01139	K02907	RP-L30, MRPL30, rpmD; large subunit ribosomal protein L30	59		
HPPR_01140	K02876	RP-L15, MRPL15, rplO; large subunit ribosomal protein L15	81		
HPPR_01141	K03076	secY; preprotein translocase subunit SecY	306		
HPPR_01142	K00939	adk, AK; adenylate kinase [EC:2.7.4.3]	89		
HPPR_01143	K02952	RP-S13, rpsM; small subunit ribosomal protein S13	89		
HPPR_01144	K02948	RP-S11, MRPS11, rpsK; small subunit ribosomal protein S11	92		
HPPR_01145	K03040	rpoA; DNA-directed RNA polymerase subunit alpha [EC:2.7.7.6]	202		
HPPR_01146	K02879	RP-L17, MRPL17, rplQ; large subunit ribosomal protein L17	104		
HPPR_01147					
HPPR_01148			77	K04771	9
HPPR_01149			2	K01823	1
HPPR_01150	K06179	rluC; 23S rRNA pseudouridine955/2504/2580 synthase [EC:5.4.99.24]	85		
HPPR_01151	K06190	ispZ; intracellular septation protein	90		

HPPR_01152	K03801	lipB; lipoyl(octanoyl) transferase [EC:2.3.1.181]	79		
HPPR_01154	K06895	lysE, argO; L-lysine exporter family protein LysE/ArgO	145		
HPPR_01155			1	K07498	1
HPPR_01156	K06213	mgtE; magnesium transporter	115		
HPPR_01157	K07091	lptF; lipopolysaccharide export system permease protein	35		
HPPR_01158	K00858	ppnK, NADK; NAD+ kinase [EC:2.7.1.23]	94		
HPPR_01159	K13588	chpT; histidine phosphotransferase ChpT	38		
HPPR_01160	K13584	ctrA; two-component system, cell cycle response regulator CtrA	104		
HPPR_01161	K01611	speD, AMD1; S-adenosylmethionine decarboxylase [EC:4.1.1.50]	86		
HPPR_01162	K00797	speE, SRM, SPE3; spermidine synthase [EC:2.5.1.16]	127		
HPPR_01163	K13587	cckA; two-component system, cell cycle sensor histidine kinase and response regula	90		
HPPR_01164	K03553	recA; recombination protein RecA	213		
HPPR_01165	K01872	AARS, alaS; alanyl-tRNA synthetase [EC:6.1.1.7]	370		
HPPR_01166			4	K03768	2
HPPR_01167	K00873	PK, pyk; pyruvate kinase [EC:2.7.1.40]	239		
HPPR_01168	K03585	acrA, mexA, adeI, smeD, mtrC, cmeA; membrane fusion protein, multidrug efflux sy	20		
HPPR_01169			278	K03296	73
HPPR_01170	K12340	tolC, bepC, cyaE, raxC, sapF, rsaF, hasF; outer membrane protein	86		
HPPR_01172	K02770	fruA; fructose PTS system EIIBC or EIIC component [EC:2.7.1.202]	132	K25814	34
HPPR_01173	K03702	uvrB; excinuclease ABC subunit B	339		
HPPR_01174			1		
HPPR_01175	K01095	pgpA; phosphatidylglycerophosphatase A [EC:3.1.3.27]	38		
HPPR_01176	K03624	greA; transcription elongation factor GreA	93		
HPPR_01177	K09117	K09117; uncharacterized protein	61		
HPPR_01178	K02316	dnaG; DNA primase [EC:2.7.7.101]	119		
HPPR_01179	K03086	rpoD; RNA polymerase primary sigma factor	186		
HPPR_01181			12	K18678	5
HPPR_01182	K11041	eta; exfoliative toxin A/B	129		
HPPR_01183	K08996	yagU; putative membrane protein	111		
HPPR_01184	K08996	yagU; putative membrane protein	110		
HPPR_01185			300		
HPPR_01186	K03760	eptA, pmrC; lipid A ethanolaminephosphotransferase [EC:2.7.8.43]	80		
HPPR_01187			104		
HPPR_01188			28		
HPPR_01189					
HPPR_01190					
