# Supplementary material for: The terrestrial isopod symbiont ‘Candidatus Hepatincola porcellionum’ is a potential nutrient scavenger related to Holosporales symbionts of protists
Source: ISME Commun. 2023 Mar 8;3:18. doi: 10.1038/s43705-023-00224-w (PMC9992710; doi:10.1038/s43705-023-00224-w)
Supplement: Supplementary file 7 — Table S6 [file 43705_2023_224_MOESM7_ESM.pdf]

Query	HMMER	Hotpep	DIAMOND	#ofTools
HAV_00064	-	GH18(64)	GH18	2
HAV_00132	GT2_Glycos_transf_2(366-523)+GT107(733-1007)	GT107(1)		2
HAV_00133	GT2_Glycos_transf_2(5-135)+GT107(346-721)	GT107(1)		2
HAV_00204	GH3(121-344)	GH3(38)		2
HAV_00438	GT19(6-351)	GT19(13)		2
HAV_00603	GT51(168-347)	GT51(36)	GT51	3
HAV_00634	CE11(3-278)	CE11(13)		2
HAV_00642	GT28(232-393)	GT28(67)		2
HAV_00940	GT2_Glycos_transf_2(9-174)	GT2(11)		2
HAV_00996	CE9(11-376)	CE9(57)	CE9	3

Query	HMMER	Hotpep	DIAMOND	#ofTools
HPDP_00052	GT2_Glycos_transf_2(5-131)+GT107(386-725)	GT107(1)		2
HPDP_00098	GH3(120-344)	GH3(38)		2
HPDP_00340	GT19(6-351)	GT19(13)		2
HPDP_00504	GT51(168-347)	GT51(36)	GT51	3
HPDP_00538	CE11(3-278)	CE11(13)		2
HPDP_00546	GT28(232-393)	GT28(67)		2
HPDP_00903	CE9(11-382)	CE9(57)	CE9	3

Query	HMMER	Hotpep	DIAMOND	#ofTools
HPPR_00079		GH18(64)	GH18	2
HPPR_00182	GH3(122-347)	GH3(38)		2
HPPR_00385	GT19(5-351)	GT19(13)		2
HPPR_00512	GH24(5-135)	GH24(11)		2
HPPR_00566	GT51(168-347)	GT51(36)	GT51	3
HPPR_00598	CE11(3-278)	CE11(13)		2
HPPR_00606	GT28(232-391)	GT28(67)		2
HPPR_00945	CE9(12-382)	CE9(57)	CE9	3
