# Supplementary material for: The terrestrial isopod symbiont ‘Candidatus Hepatincola porcellionum’ is a potential nutrient scavenger related to Holosporales symbionts of protists
Source: ISME Commun. 2023 Mar 8;3:18. doi: 10.1038/s43705-023-00224-w (PMC9992710; doi:10.1038/s43705-023-00224-w)
Supplement: Supplementary file 8 — Table S7 [file 43705_2023_224_MOESM8_ESM.pdf]

CDS	FID	Transporter Family	Subfamily	Substrate/Function
HAV_00060	ABC	The ATP-binding Cassette (ABC) Superfamily	membrane	lipoprotein releasing
HAV_00061	ABC	The ATP-binding Cassette (ABC) Superfamily	membrane	lipoprotein releasing
HAV_00062	ABC	The ATP-binding Cassette (ABC) Superfamily	binding	lipoprotein
HAV_00096	ABC	The ATP-binding Cassette (ABC) Superfamily	membrane	manganese/zinc ion
HAV_00097	ABC	The ATP-binding Cassette (ABC) Superfamily	binding	molybdate
HAV_00111	ABC	The ATP-binding Cassette (ABC) Superfamily	binding	zinc
HAV_00112	ABC	The ATP-binding Cassette (ABC) Superfamily	binding	zinc
HAV_00259	ABC	The ATP-binding Cassette (ABC) Superfamily	membrane	lipoprotein releasing
HAV_00260	ABC	The ATP-binding Cassette (ABC) Superfamily	binding	lipoprotein
HAV_00281	ABC	The ATP-binding Cassette (ABC) Superfamily	binding protein	toluene tolerance
HAV_00282	ABC	The ATP-binding Cassette (ABC) Superfamily	binding protein	toluene tolerance
HAV_00365	ABC	The ATP-binding Cassette (ABC) Superfamily	binding	methionine
HAV_00366	ABC	The ATP-binding Cassette (ABC) Superfamily	binding	methionine
HAV_00367	ABC	The ATP-binding Cassette (ABC) Superfamily	membrane	D-methionine
HAV_00368	ABC	The ATP-binding Cassette (ABC) Superfamily	binding	D-methionine
HAV_00573	ABC	The ATP-binding Cassette (ABC) Superfamily	binding	toluene tolerance
HAV_00589	ABC	The ATP-binding Cassette (ABC) Superfamily	binding	sulfate
HAV_00590	ABC	The ATP-binding Cassette (ABC) Superfamily	membrane	toluene tolerance
HAV_00679	ABC	The ATP-binding Cassette (ABC) Superfamily	binding	heme
HAV_00737	ABC	The ATP-binding Cassette (ABC) Superfamily	membrane	polysaccharide export
HAV_00763	ABC	The ATP-binding Cassette (ABC) Superfamily	ABC+ membrane	toxin secretion (annotated as T1SS ATPase)
HAV_00823	ABC	The ATP-binding Cassette (ABC) Superfamily	binding	lipid A
HAV_00846	ABC	The ATP-binding Cassette (ABC) Superfamily	binding	phosphonate
HAV_00847	ABC	The ATP-binding Cassette (ABC) Superfamily	membrane	cell division
HAV_00865	ABC	The ATP-binding Cassette (ABC) Superfamily	binding	daunorubicin
HAV_00885	ABC	The ATP-binding Cassette (ABC) Superfamily	binding	sulfate
HAV_00886	ABC	The ATP-binding Cassette (ABC) Superfamily		organic solvents
HAV_00962	ABC	The ATP-binding Cassette (ABC) Superfamily	membrane	phosphate
HAV_00963	ABC	The ATP-binding Cassette (ABC) Superfamily	membrane	phosphate
HAV_00964	ABC	The ATP-binding Cassette (ABC) Superfamily	binding	phosphate
HAV_01007	ABC	The ATP-binding Cassette (ABC) Superfamily	binding protein	dipeptide/oligopeptide
HAV_01008	ABC	The ATP-binding Cassette (ABC) Superfamily	membrane	dipeptide/oligopeptide
HAV_01009	ABC	The ATP-binding Cassette (ABC) Superfamily	membrane	dipeptide/oligopeptide
HAV_01010	ABC	The ATP-binding Cassette (ABC) Superfamily	binding	lipoprotein
HAV_01031	ABC	The ATP-binding Cassette (ABC) Superfamily	ABC	? (Uup homolog/duplicated ATPase)
HAV_01062	ABC	The ATP-binding Cassette (ABC) Superfamily	binding protein	amino acid (glutamine/glutamate/aspartate?)
HAV_00014	ACR3	The Arsenical Resistance-3 (ACR3) Family		arsenite
HAV_01044	CDF	The Cation Diffusion Facilitator (CDF) Family		cation efflux
HAV_00202	CHR	The Chromate Ion Transporter (CHR) Family		chromate ion
HAV_00203	CHR	The Chromate Ion Transporter (CHR) Family		chromate ion
HAV_00019	CNT	The Concentrative Nucleoside Transporter (CNT) Family		sodium ion:nucleoside symporter
HAV_00247	CPA3	The Monovalent Cation (K+ or Na+):Proton Antiporter-3 (CPA3) Family	MnhA	multicomponent sodium ion:proton antiporter
HAV_00533	CPA3	The Monovalent Cation (K+ or Na+):Proton Antiporter-3 (CPA3) Family	MnhE	multicomponent sodium ion:proton antiporter
HAV_00534	CPA3	The Monovalent Cation (K+ or Na+):Proton Antiporter-3 (CPA3) Family	MnhF	multicomponent sodium ion:proton antiporter
HAV_00535	CPA3	The Monovalent Cation (K+ or Na+):Proton Antiporter-3 (CPA3) Family	MnhG	multicomponent sodium ion:proton antiporter
HAV_00536	CPA3	The Monovalent Cation (K+ or Na+):Proton Antiporter-3 (CPA3) Family		sodium ion:proton antiporter

HAV_00537	CPA3	The Monovalent Cation (K+ or Na+):Proton Antiporter-3 (CPA3) Family	MnhC	multicomponent sodium ion:proton antiporter
HAV_00539	CPA3	The Monovalent Cation (K+ or Na+):Proton Antiporter-3 (CPA3) Family	MnhA	multicomponent sodium ion:proton antiporter
HAV_00685	DAACS	The Dicarboxylate/Amino Acid:Cation (Na+ or H+) Symporter (DAACS) Family		proton/sodium ion:glutamate/aspartate symporter
HAV_01066	DAACS	The Dicarboxylate/Amino Acid:Cation (Na+ or H+) Symporter (DAACS) Family		proton/sodium ion:glutamate/aspartate symporter
HAV_00409	DASS	The Divalent Anion:Na+ Symporter (DASS) Family		sodium ion:dicarboxylate/sulfate
HAV_00433	Dcu	The C4-Dicarboxylate Uptake (Dcu) Family		C4-dicarboxylate
HAV_00434	Dcu	The C4-Dicarboxylate Uptake (Dcu) Family		C4-dicarboxylate
HAV_00350	DcuC	The C4-dicarboxylate Uptake C (DcuC) Family		C4-dicarboxylate
HAV_00519	DcuC	The C4-dicarboxylate Uptake C (DcuC) Family		C4-dicarboxylate
HAV_00027	DMT	The Drug/Metabolite Transporter (DMT) Superfamily		drug/metabolite?
HAV_00028	DMT	The Drug/Metabolite Transporter (DMT) Superfamily	RarD	chloramphenicol (RarD homolog)
HAV_00029	DMT	The Drug/Metabolite Transporter (DMT) Superfamily		drug/metabolite?
HAV_00490	DMT	The Drug/Metabolite Transporter (DMT) Superfamily		drug/metabolite?
HAV_00773	DNA-T	The Bacterial Competence-related DNA Transformation Transporter (DNA-T) Family		
HAV_00690	F-ATPase	The H+- or Na+-translocating F-type, V-type and A-type ATPase (F-ATPase) Superfamily		protons
HAV_00691	F-ATPase	The H+- or Na+-translocating F-type, V-type and A-type ATPase (F-ATPase) Superfamily		protons
HAV_00692	F-ATPase	The H+- or Na+-translocating F-type, V-type and A-type ATPase (F-ATPase) Superfamily		protons
HAV_00757	F-ATPase	The H+- or Na+-translocating F-type, V-type and A-type ATPase (F-ATPase) Superfamily		protons
HAV_00758	F-ATPase	The H+- or Na+-translocating F-type, V-type and A-type ATPase (F-ATPase) Superfamily		protons
HAV_00759	F-ATPase	The H+- or Na+-translocating F-type, V-type and A-type ATPase (F-ATPase) Superfamily		protons
HAV_00760	F-ATPase	The H+- or Na+-translocating F-type, V-type and A-type ATPase (F-ATPase) Superfamily		protons
HAV_00783	FeoB	The Ferrous Iron Uptake (FeoB) Family		ferrous ion
HAV_01100	GntP	The Gluconate:H+ Symporter (GntP) Family		gluconate
HAV_00992	GPTS	General PTS	EnzymeI	
HAV_00562	HAAAP	The Hydroxy/Aromatic Amino Acid Permease (HAAAP) Family		serine
HAV_00144	HCC	The HlyC/CorC (HCC) Family		heavy metal ion
HAV_00154	IVSP	The Type IV (Conjugal DNA-Protein Transfer or VirB) Secretory Pathway (IVSP) Family		
HAV_00167	IVSP	The Type IV (Conjugal DNA-Protein Transfer or VirB) Secretory Pathway (IVSP) Family		
HAV_00336	LctP	The Lactate Permease (LctP) Family		L-lactate
HAV_00131	LysE	The L-Lysine Exporter (LysE) Family		lysine efflux
HAV_00335	MFS	The Major Facilitator Superfamily (MFS)		multidrug efflux
HAV_00393	MFS	The Major Facilitator Superfamily (MFS)		glycerol-3-phosphate
HAV_00564	MFS	The Major Facilitator Superfamily (MFS)		multidrug efflux
HAV_00715	MFS	The Major Facilitator Superfamily (MFS)		sugar
HAV_00718	MFS	The Major Facilitator Superfamily (MFS)		sugar
HAV_00756	MFS	The Major Facilitator Superfamily (MFS)		multidrug efflux (Bcr/CflA subfamily)
HAV_00808	MFS	The Major Facilitator Superfamily (MFS)		multidrug efflux
HAV_00810	MFS	The Major Facilitator Superfamily (MFS)		multidrug efflux
HAV_00811	MFS	The Major Facilitator Superfamily (MFS)		multidrug efflux
HAV_00815	MFS	The Major Facilitator Superfamily (MFS)		multidrug efflux
HAV_01205	MgtE	The Mg2+ Transporter-E (MgtE) Family		magnesium ion
HAV_00124	MIP	The Major Intrinsic Protein (MIP) Family		glycerol uptake
HAV_00363	MOP	The Multidrug/Oligosaccharidyl-lipid/Polysaccharide (MOP) Flippase Superfamily	MATE	multidrug efflux
HAV_00882	MOP	The Multidrug/Oligosaccharidyl-lipid/Polysaccharide (MOP) Flippase Superfamily	MATE	multidrug efflux
HAV_00924	MOP	The Multidrug/Oligosaccharidyl-lipid/Polysaccharide (MOP) Flippase Superfamily	MVF	virulence factor MviN (annotated as putative lipid II flippase MurJ)
HAV_00792	Mot/Exb	The H+- or Na+-translocating Bacterial Flagellar Motor 1ExbBD Outer Membrane Transport Energizer (Mo		
HAV_00793	Mot/Exb	The H+- or Na+-translocating Bacterial Flagellar Motor 1ExbBD Outer Membrane Transport Energizer (Mo		

HAV_00136	MscS	The Small Conductance Mechanosensitive Ion Channel (MscS) Family		small-conductance mechanosensitive ion channel
HAV_00256	NCS2	The Nucleobase:Cation Symporter-2 (NCS2) Family		xanthine/uracil
HAV_00470	NCS2	The Nucleobase:Cation Symporter-2 (NCS2) Family		xanthine/uracil
HAV_01149	Nramp	The Metal Ion (Mn2+-iron) Transporter (Nramp) Family		manganese/iron ion
HAV_00216	NSS	The Neurotransmitter:Sodium Symporter (NSS) Family		sodium ion:solute symporter
HAV_00217	NSS	The Neurotransmitter:Sodium Symporter (NSS) Family		sodium ion:solute symporter
HAV_00379	NSS	The Neurotransmitter:Sodium Symporter (NSS) Family		sodium ion:solute symporter
HAV_01098	Oxa1	The Cytochrome Oxidase Biogenesis (Oxa1) Family		60 KD inner membrane protein OxaA homolog
HAV_00121	P-ATPase	The P-type ATPase (P-ATPase) Superfamily		zinc/cadmium/cobalt ion
HAV_00185	POT	The Proton-dependent Oligopeptide Transporter (POT) Family		proton:dipeptide/tripeptide symporter
HAV_00106	RND	The Resistance-Nodulation-Cell Division (RND) Superfamily	SecDF	protein export (SecDF)
HAV_01218	RND	The Resistance-Nodulation-Cell Division (RND) Superfamily	HAE1	multidrug/solvent efflux (HAE1 subfamily)
HAV_00048	SSPTS	Sugar Specific PTS	EnzymeIIABC	glucose/maltose/N-acetylglucosamine
HAV_00375	SSPTS	Sugar Specific PTS	EnzymeIIABC	glucose/maltose/N-acetylglucosamine
HAV_00554	SSPTS	Sugar Specific PTS	EnzymeIIABC	mannitol
HAV_00993	SSPTS	Sugar Specific PTS	EnzymeIIABC	glucose/maltose/N-acetylglucosamine
HAV_01221	SSPTS	Sugar Specific PTS	EnzymeIIABC	fructose
HAV_00013	SSS	The Solute:Sodium Symporter (SSS) Family		sodium ion:proline symporter
HAV_00492	Tat	The Twin Arginine Targeting (Tat) Family		protein export
HAV_01038	Tat	The Twin Arginine Targeting (Tat) Family		protein export
HAV_01040	Tat	The Twin Arginine Targeting (Tat) Family		protein export
HAV_01230	TDT	The Telurite-resistance/Dicarboxylate Transporter (TDT) Family		tellurite
HAV_00229	TerC	The Tellurium Ion Resistance (TerC) Family		tellurium ion efflux
HAV_01142	Trk	The K+ Transporter (Trk) Family		potassium ion uptake

CDS	FID	Transporter Family	Subfamily	Substrate/Function
HPDP_00032	ABC	The ATP-binding Cassette (ABC) Superfamily	binding	zinc
HPDP_00154	ABC	The ATP-binding Cassette (ABC) Superfamily	membrane	lipoprotein releasing
HPDP_00155	ABC	The ATP-binding Cassette (ABC) Superfamily	binding	lipoprotein
HPDP_00179	ABC	The ATP-binding Cassette (ABC) Superfamily	binding protein	toluene tolerance
HPDP_00180	ABC	The ATP-binding Cassette (ABC) Superfamily	binding protein	toluene tolerance
HPDP_00261	ABC	The ATP-binding Cassette (ABC) Superfamily	binding	methionine
HPDP_00262	ABC	The ATP-binding Cassette (ABC) Superfamily	binding	methionine
HPDP_00263	ABC	The ATP-binding Cassette (ABC) Superfamily	membrane	D-methionine
HPDP_00264	ABC	The ATP-binding Cassette (ABC) Superfamily	binding	D-methionine
HPDP_00473	ABC	The ATP-binding Cassette (ABC) Superfamily	binding	toluene tolerance
HPDP_00490	ABC	The ATP-binding Cassette (ABC) Superfamily	binding	sulfate
HPDP_00491	ABC	The ATP-binding Cassette (ABC) Superfamily	membrane	toluene tolerance
HPDP_00586	ABC	The ATP-binding Cassette (ABC) Superfamily	binding	heme
HPDP_00643	ABC	The ATP-binding Cassette (ABC) Superfamily	membrane	polysaccharide export
HPDP_00669	ABC	The ATP-binding Cassette (ABC) Superfamily	ABC+ membrane	toxin secretion (annotated as T1SS ATPase)
HPDP_00730	ABC	The ATP-binding Cassette (ABC) Superfamily	binding	lipid A
HPDP_00753	ABC	The ATP-binding Cassette (ABC) Superfamily	binding	phosphonate
HPDP_00754	ABC	The ATP-binding Cassette (ABC) Superfamily	membrane	cell division
HPDP_00771	ABC	The ATP-binding Cassette (ABC) Superfamily	binding	daunorubicin
HPDP_00791	ABC	The ATP-binding Cassette (ABC) Superfamily	binding	sulfate
HPDP_00792	ABC	The ATP-binding Cassette (ABC) Superfamily		organic solvents
HPDP_00868	ABC	The ATP-binding Cassette (ABC) Superfamily	membrane	phosphate
HPDP_00869	ABC	The ATP-binding Cassette (ABC) Superfamily	membrane	phosphate
HPDP_00870	ABC	The ATP-binding Cassette (ABC) Superfamily	binding	phosphate
HPDP_00914	ABC	The ATP-binding Cassette (ABC) Superfamily	binding protein	dipeptide/oligopeptide
HPDP_00915	ABC	The ATP-binding Cassette (ABC) Superfamily	membrane	dipeptide/oligopeptide
HPDP_00916	ABC	The ATP-binding Cassette (ABC) Superfamily	membrane	dipeptide/oligopeptide
HPDP_00917	ABC	The ATP-binding Cassette (ABC) Superfamily	binding	lipoprotein
HPDP_00939	ABC	The ATP-binding Cassette (ABC) Superfamily	ABC	? (Uup homolog/duplicated ATPase)
HPDP_00970	ABC	The ATP-binding Cassette (ABC) Superfamily	binding protein	amino acid (glutamine/glutamate/aspartate?)
HPDP_00014	ACR3	The Arsenical Resistance-3 (ACR3) Family		arsenite
HPDP_00952	CDF	The Cation Diffusion Facilitator (CDF) Family		cation efflux
HPDP_00096	CHR	The Chromate Ion Transporter (CHR) Family		chromate ion
HPDP_00097	CHR	The Chromate Ion Transporter (CHR) Family		chromate ion
HPDP_00019	CNT	The Concentrative Nucleoside Transporter (CNT) Family		sodium ion:nucleoside symporter
HPDP_00141	CPA3	The Monovalent Cation (K+ or Na+):Proton Antiporter-3 (CPA3) Family	MnhA	multicomponent sodium ion:proton antiporter
HPDP_00431	CPA3	The Monovalent Cation (K+ or Na+):Proton Antiporter-3 (CPA3) Family	MnhE	multicomponent sodium ion:proton antiporter
HPDP_00432	CPA3	The Monovalent Cation (K+ or Na+):Proton Antiporter-3 (CPA3) Family	MnhF	multicomponent sodium ion:proton antiporter
HPDP_00433	CPA3	The Monovalent Cation (K+ or Na+):Proton Antiporter-3 (CPA3) Family	MnhG	multicomponent sodium ion:proton antiporter
HPDP_00434	CPA3	The Monovalent Cation (K+ or Na+):Proton Antiporter-3 (CPA3) Family		sodium ion:proton antiporter
HPDP_00435	CPA3	The Monovalent Cation (K+ or Na+):Proton Antiporter-3 (CPA3) Family	MnhC	multicomponent sodium ion:proton antiporter
HPDP_00437	CPA3	The Monovalent Cation (K+ or Na+):Proton Antiporter-3 (CPA3) Family	MnhA	multicomponent sodium ion:proton antiporter
HPDP_00592	DAACS	The Dicarboxylate/Amino Acid:Cation (Na+ or H+) Symporter (DAACS) Family		proton/sodium ion:glutamate/aspartate symporter
HPDP_00974	DAACS	The Dicarboxylate/Amino Acid:Cation (Na+ or H+) Symporter (DAACS) Family		proton/sodium ion:glutamate/aspartate symporter
HPDP_00306	DASS	The Divalent Anion:Na+ Symporter (DASS) Family		sodium ion:dicarboxylate/sulfate
HPDP_00335	Dcu	The C4-Dicarboxylate Uptake (Dcu) Family		C4-dicarboxylate
HPDP_00336	Dcu	The C4-Dicarboxylate Uptake (Dcu) Family		C4-dicarboxylate

HPDP_00246	DcuC	The C4-dicarboxylate Uptake C (DcuC) Family		C4-dicarboxylate
HPDP_00414	DcuC	The C4-dicarboxylate Uptake C (DcuC) Family		C4-dicarboxylate
HPDP_00027	DMT	The Drug/Metabolite Transporter (DMT) Superfamily	RarD	chloramphenicol (RarD homolog)
HPDP_00028	DMT	The Drug/Metabolite Transporter (DMT) Superfamily	RarD	chloramphenicol (RarD homolog)
HPDP_00029	DMT	The Drug/Metabolite Transporter (DMT) Superfamily		drug/metabolite?
HPDP_00387	DMT	The Drug/Metabolite Transporter (DMT) Superfamily	RarD	chloramphenicol (RarD homolog)
HPDP_00444	DMT	The Drug/Metabolite Transporter (DMT) Superfamily		drug/metabolite?
HPDP_00678	DNA-T	The Bacterial Competence-related DNA Transformation Transporter (DNA-T) Family		
HPDP_00597	F-ATPase	The H+- or Na+-translocating F-type, V-type and A-type ATPase (F-ATPase) Superfamily		protons
HPDP_00598	F-ATPase	The H+- or Na+-translocating F-type, V-type and A-type ATPase (F-ATPase) Superfamily		protons
HPDP_00599	F-ATPase	The H+- or Na+-translocating F-type, V-type and A-type ATPase (F-ATPase) Superfamily		protons
HPDP_00663	F-ATPase	The H+- or Na+-translocating F-type, V-type and A-type ATPase (F-ATPase) Superfamily		protons
HPDP_00664	F-ATPase	The H+- or Na+-translocating F-type, V-type and A-type ATPase (F-ATPase) Superfamily		protons
HPDP_00665	F-ATPase	The H+- or Na+-translocating F-type, V-type and A-type ATPase (F-ATPase) Superfamily		protons
HPDP_00666	F-ATPase	The H+- or Na+-translocating F-type, V-type and A-type ATPase (F-ATPase) Superfamily		protons
HPDP_00689	FeoB	The Ferrous Iron Uptake (FeoB) Family		ferrous ion
HPDP_01012	GntP	The Gluconate:H+ Symporter (GntP) Family		gluconate
HPDP_00897	GPTS	General PTS	EnzymeI	
HPDP_00461	HAAAP	The Hydroxy/Aromatic Amino Acid Permease (HAAAP) Family		serine
HPDP_00063	HCC	The HlyC/CorC (HCC) Family		heavy metal ion
HPDP_00232	LctP	The Lactate Permease (LctP) Family		L-lactate
HPDP_00051	LysE	The L-Lysine Exporter (LysE) Family		lysine efflux
HPDP_00070	MFS	The Major Facilitator Superfamily (MFS)		multidrug efflux
HPDP_00227	MFS	The Major Facilitator Superfamily (MFS)		multidrug efflux
HPDP_00228	MFS	The Major Facilitator Superfamily (MFS)		multidrug efflux
HPDP_00291	MFS	The Major Facilitator Superfamily (MFS)		glycerol-3-phosphate
HPDP_00464	MFS	The Major Facilitator Superfamily (MFS)		multidrug efflux
HPDP_00617	MFS	The Major Facilitator Superfamily (MFS)		sugar efflux?
HPDP_00619	MFS	The Major Facilitator Superfamily (MFS)		tetracycline efflux?
HPDP_00715	MFS	The Major Facilitator Superfamily (MFS)		multidrug efflux
HPDP_00717	MFS	The Major Facilitator Superfamily (MFS)		multidrug efflux
HPDP_00718	MFS	The Major Facilitator Superfamily (MFS)		multidrug efflux
HPDP_00722	MFS	The Major Facilitator Superfamily (MFS)		multidrug efflux
HPDP_01119	MgtE	The Mg2+ Transporter-E (MgtE) Family		magnesium ion
HPDP_00045	MIP	The Major Intrinsic Protein (MIP) Family		glycerol uptake
HPDP_00260	MOP	The Multidrug/Oligosaccharidyl-lipid/Polysaccharide (MOP) Flippase Superfamily	MATE	multidrug efflux
HPDP_00788	MOP	The Multidrug/Oligosaccharidyl-lipid/Polysaccharide (MOP) Flippase Superfamily	MATE	multidrug efflux
HPDP_00830	MOP	The Multidrug/Oligosaccharidyl-lipid/Polysaccharide (MOP) Flippase Superfamily	MVF	virulence factor MviN (annotated as putative lipid II flippase MurJ)
HPDP_00699	Mot/Exb	The H+- or Na+-translocating Bacterial Flagellar Motor 1ExbBD Outer Membrane Transport Energizer (Mo		
HPDP_00700	Mot/Exb	The H+- or Na+-translocating Bacterial Flagellar Motor 1ExbBD Outer Membrane Transport Energizer (Mo		
HPDP_00055	MscS	The Small Conductance Mechanosensitive Ion Channel (MscS) Family		small-conductance mechanosensitive ion channel
HPDP_00151	NCS2	The Nucleobase:Cation Symporter-2 (NCS2) Family		xanthine/uracil
HPDP_00372	NCS2	The Nucleobase:Cation Symporter-2 (NCS2) Family		xanthine/uracil
HPDP_01060	Nramp	The Metal Ion (Mn2+-iron) Transporter (Nramp) Family		manganese/iron ion
HPDP_00110	NSS	The Neurotransmitter:Sodium Symporter (NSS) Family		sodium ion:solute symporter
HPDP_00111	NSS	The Neurotransmitter:Sodium Symporter (NSS) Family		sodium ion:solute symporter
HPDP_00276	NSS	The Neurotransmitter:Sodium Symporter (NSS) Family		sodium ion:solute symporter
HPDP_00558	Oxa1	The Cytochrome Oxidase Biogenesis (Oxa1) Family		60 KD inner membrane protein OxaA homolog

HPDP_01010	Oxa1	The Cytochrome Oxidase Biogenesis (Oxa1) Family		60 KD inner membrane protein OxaA homolog
HPDP_00042	P-ATPase	The P-type ATPase (P-ATPase) Superfamily		zinc/cadmium/cobalt ion
HPDP_00079	POT	The Proton-dependent Oligopeptide Transporter (POT) Family		proton:dipeptide/tripeptide symporter
HPDP_01132	RND	The Resistance-Nodulation-Cell Division (RND) Superfamily	HAE1	multidrug/solvent efflux (HAE1 subfamily)
HPDP_00272	SSPTS	Sugar Specific PTS	EnzymeIIABC	glucose/maltose/N-acetylglucosamine
HPDP_00453	SSPTS	Sugar Specific PTS	EnzymeIIABC	mannitol
HPDP_00900	SSPTS	Sugar Specific PTS	EnzymeIIABC	glucose/maltose/N-acetylglucosamine
HPDP_01135	SSPTS	Sugar Specific PTS	EnzymeIIABC	fructose
HPDP_00013	SSS	The Solute:Sodium Symporter (SSS) Family		sodium ion:proline symporter
HPDP_00389	Tat	The Twin Arginine Targeting (Tat) Family		protein export
HPDP_00946	Tat	The Twin Arginine Targeting (Tat) Family		protein export
HPDP_00948	Tat	The Twin Arginine Targeting (Tat) Family		protein export
HPDP_01144	TDT	The Telurite-resistance/Dicarboxylate Transporter (TDT) Family		tellurite
HPDP_00123	TerC	The Tellurium Ion Resistance (TerC) Family		tellurium ion efflux
HPDP_01053	Trk	The K+ Transporter (Trk) Family		potassium ion uptake

CDS	FID	Transporter Family	Subfamily	Substrate/Function
HPPR_00071	ABC	The ATP-binding Cassette (ABC) Superfamily	membrane	lipoprotein releasing
HPPR_00072	ABC	The ATP-binding Cassette (ABC) Superfamily	membrane	lipoprotein releasing
HPPR_00073	ABC	The ATP-binding Cassette (ABC) Superfamily	binding	lipoprotein
HPPR_00106	ABC	The ATP-binding Cassette (ABC) Superfamily	membrane	manganese/zinc ion
HPPR_00107	ABC	The ATP-binding Cassette (ABC) Superfamily	binding	molybdate
HPPR_00120	ABC	The ATP-binding Cassette (ABC) Superfamily	binding	zinc
HPPR_00121	ABC	The ATP-binding Cassette (ABC) Superfamily	binding	zinc
HPPR_00236	ABC	The ATP-binding Cassette (ABC) Superfamily	membrane	lipoprotein releasing
HPPR_00237	ABC	The ATP-binding Cassette (ABC) Superfamily	binding	lipoprotein
HPPR_00259	ABC	The ATP-binding Cassette (ABC) Superfamily	binding protein	toluene tolerance
HPPR_00260	ABC	The ATP-binding Cassette (ABC) Superfamily	binding protein	toluene tolerance
HPPR_00314	ABC	The ATP-binding Cassette (ABC) Superfamily	binding	methionine
HPPR_00315	ABC	The ATP-binding Cassette (ABC) Superfamily	binding	methionine
HPPR_00316	ABC	The ATP-binding Cassette (ABC) Superfamily	membrane	D-methionine
HPPR_00317	ABC	The ATP-binding Cassette (ABC) Superfamily	binding	D-methionine
HPPR_00535	ABC	The ATP-binding Cassette (ABC) Superfamily	binding	toluene tolerance
HPPR_00552	ABC	The ATP-binding Cassette (ABC) Superfamily	binding	sulfate
HPPR_00553	ABC	The ATP-binding Cassette (ABC) Superfamily	membrane	toluene tolerance
HPPR_00643	ABC	The ATP-binding Cassette (ABC) Superfamily	binding	heme
HPPR_00694	ABC	The ATP-binding Cassette (ABC) Superfamily	membrane	polysaccharide export
HPPR_00720	ABC	The ATP-binding Cassette (ABC) Superfamily	ABC+ membrane	toxin secretion (annotated as T1SS ATPase)
HPPR_00781	ABC	The ATP-binding Cassette (ABC) Superfamily	binding	lipid A
HPPR_00803	ABC	The ATP-binding Cassette (ABC) Superfamily	binding	phosphonate
HPPR_00804	ABC	The ATP-binding Cassette (ABC) Superfamily	membrane	cell division
HPPR_00821	ABC	The ATP-binding Cassette (ABC) Superfamily	binding	daunorubicin
HPPR_00840	ABC	The ATP-binding Cassette (ABC) Superfamily	binding	sulfate
HPPR_00841	ABC	The ATP-binding Cassette (ABC) Superfamily		organic solvents
HPPR_00913	ABC	The ATP-binding Cassette (ABC) Superfamily	membrane	phosphate
HPPR_00914	ABC	The ATP-binding Cassette (ABC) Superfamily	membrane	phosphate
HPPR_00915	ABC	The ATP-binding Cassette (ABC) Superfamily	binding	phosphate
HPPR_00933	ABC	The ATP-binding Cassette (ABC) Superfamily	binding	leucine/valine
HPPR_00954	ABC	The ATP-binding Cassette (ABC) Superfamily	binding protein	dipeptide/oligopeptide
HPPR_00955	ABC	The ATP-binding Cassette (ABC) Superfamily	membrane	dipeptide/oligopeptide
HPPR_00956	ABC	The ATP-binding Cassette (ABC) Superfamily	membrane	dipeptide/oligopeptide
HPPR_00957	ABC	The ATP-binding Cassette (ABC) Superfamily	binding	lipoprotein
HPPR_00977	ABC	The ATP-binding Cassette (ABC) Superfamily	ABC	? (Uup homolog/duplicated ATPase)
HPPR_01009	ABC	The ATP-binding Cassette (ABC) Superfamily	binding protein	?
HPPR_00991	CDF	The Cation Diffusion Facilitator (CDF) Family		cation efflux
HPPR_00180	CHR	The Chromate Ion Transporter (CHR) Family		chromate ion
HPPR_00181	CHR	The Chromate Ion Transporter (CHR) Family		chromate ion
HPPR_00023	CNT	The Concentrative Nucleoside Transporter (CNT) Family		sodium ion:nucleoside symporter
HPPR_00226	CPA3	The Monovalent Cation (K+ or Na+):Proton Antiporter-3 (CPA3) Family	MnhA	multicomponent sodium ion:proton antiporter
HPPR_00471	CPA3	The Monovalent Cation (K+ or Na+):Proton Antiporter-3 (CPA3) Family	MnhE	multicomponent sodium ion:proton antiporter
HPPR_00472	CPA3	The Monovalent Cation (K+ or Na+):Proton Antiporter-3 (CPA3) Family		sodium ion:proton antiporter
HPPR_00473	CPA3	The Monovalent Cation (K+ or Na+):Proton Antiporter-3 (CPA3) Family	MnhG	multicomponent sodium ion:proton antiporter
HPPR_00474	CPA3	The Monovalent Cation (K+ or Na+):Proton Antiporter-3 (CPA3) Family		sodium ion:proton antiporter
HPPR_00475	CPA3	The Monovalent Cation (K+ or Na+):Proton Antiporter-3 (CPA3) Family	MnhC	multicomponent sodium ion:proton antiporter

HPPR_00476	CPA3	The Monovalent Cation (K+ or Na+):Proton Antiporter-3 (CPA3) Family	MnhD	multicomponent sodium ion:proton antiporter
HPPR_00477	CPA3	The Monovalent Cation (K+ or Na+):Proton Antiporter-3 (CPA3) Family	MnhA	multicomponent sodium ion:proton antiporter
HPPR_00648	DAACS	The Dicarboxylate/Amino Acid:Cation (Na+ or H+) Symporter (DAACS) Family		proton/sodium ion:glutamate/aspartate symporter
HPPR_01013	DAACS	The Dicarboxylate/Amino Acid:Cation (Na+ or H+) Symporter (DAACS) Family		proton/sodium ion:glutamate/aspartate symporter
HPPR_00355	DASS	The Divalent Anion:Na+ Symporter (DASS) Family		sodium ion:dicarboxylate/sulfate
HPPR_00380	Dcu	The C4-Dicarboxylate Uptake (Dcu) Family		C4-dicarboxylate
HPPR_00381	Dcu	The C4-Dicarboxylate Uptake (Dcu) Family		C4-dicarboxylate
HPPR_00298	DcuC	The C4-dicarboxylate Uptake C (DcuC) Family		C4-dicarboxylate
HPPR_00457	DcuC	The C4-dicarboxylate Uptake C (DcuC) Family		C4-dicarboxylate
HPPR_00038	DMT	The Drug/Metabolite Transporter (DMT) Superfamily		drug/metabolite?
HPPR_00039	DMT	The Drug/Metabolite Transporter (DMT) Superfamily	RarD	chloramphenicol (RarD homolog)
HPPR_00040	DMT	The Drug/Metabolite Transporter (DMT) Superfamily		drug/metabolite?
HPPR_00432	DMT	The Drug/Metabolite Transporter (DMT) Superfamily	RarD	chloramphenicol (RarD homolog)
HPPR_00653	F-ATPase	The H+- or Na+-translocating F-type, V-type and A-type ATPase (F-ATPase) Superfamily		protons
HPPR_00654	F-ATPase	The H+- or Na+-translocating F-type, V-type and A-type ATPase (F-ATPase) Superfamily		protons
HPPR_00655	F-ATPase	The H+- or Na+-translocating F-type, V-type and A-type ATPase (F-ATPase) Superfamily		protons
HPPR_00714	F-ATPase	The H+- or Na+-translocating F-type, V-type and A-type ATPase (F-ATPase) Superfamily		protons
HPPR_00715	F-ATPase	The H+- or Na+-translocating F-type, V-type and A-type ATPase (F-ATPase) Superfamily		protons
HPPR_00716	F-ATPase	The H+- or Na+-translocating F-type, V-type and A-type ATPase (F-ATPase) Superfamily		protons
HPPR_00717	F-ATPase	The H+- or Na+-translocating F-type, V-type and A-type ATPase (F-ATPase) Superfamily		protons
HPPR_00741	FeoB	The Ferrous Iron Uptake (FeoB) Family		ferrous ion
HPPR_01045	GntP	The Gluconate:H+ Symporter (GntP) Family		gluconate
HPPR_00942	GPTS	General PTS	EnzymeI	
HPPR_00498	HAAAP	The Hydroxy/Aromatic Amino Acid Permease (HAAAP) Family		serine
HPPR_00150	HCC	The HlyC/CorC (HCC) Family		heavy metal ion
HPPR_00284	LctP	The Lactate Permease (LctP) Family		L-lactate
HPPR_01154	LysE	The L-Lysine Exporter (LysE) Family		lysine efflux
HPPR_00032	MFS	The Major Facilitator Superfamily (MFS)		multidrug efflux
HPPR_00339	MFS	The Major Facilitator Superfamily (MFS)		glycerol-3-phosphate
HPPR_00500	MFS	The Major Facilitator Superfamily (MFS)		multidrug efflux
HPPR_00673	MFS	The Major Facilitator Superfamily (MFS)		sugar
HPPR_00766	MFS	The Major Facilitator Superfamily (MFS)		multidrug efflux
HPPR_00768	MFS	The Major Facilitator Superfamily (MFS)		multidrug efflux
HPPR_00769	MFS	The Major Facilitator Superfamily (MFS)		multidrug efflux
HPPR_00773	MFS	The Major Facilitator Superfamily (MFS)		multidrug efflux
HPPR_01094	MFS	The Major Facilitator Superfamily (MFS)		sugar
HPPR_01156	MgtE	The Mg2+ Transporter-E (MgtE) Family		magnesium ion
HPPR_00134	MIP	The Major Intrinsic Protein (MIP) Family		glycerol uptake
HPPR_00311	MOP	The Multidrug/Oligosaccharidyl-lipid/Polysaccharide (MOP) Flippase Superfamily	MATE	multidrug efflux
HPPR_00837	MOP	The Multidrug/Oligosaccharidyl-lipid/Polysaccharide (MOP) Flippase Superfamily	MATE	multidrug efflux
HPPR_00879	MOP	The Multidrug/Oligosaccharidyl-lipid/Polysaccharide (MOP) Flippase Superfamily	MVF	virulence factor MviN (annotated as putative lipid II flippase MurJ)
HPPR_00750	Mot/Exb	The H+- or Na+-translocating Bacterial Flagellar Motor 1ExbBD Outer Membrane Transport Energizer (Mo		
HPPR_00751	Mot/Exb	The H+- or Na+-translocating Bacterial Flagellar Motor 1ExbBD Outer Membrane Transport Energizer (Mo		
HPPR_00142	MscS	The Small Conductance Mechanosensitive Ion Channel (MscS) Family		small-conductance mechanosensitive ion channel
HPPR_00234	NCS2	The Nucleobase:Cation Symporter-2 (NCS2) Family		xanthine/uracil
HPPR_00416	NCS2	The Nucleobase:Cation Symporter-2 (NCS2) Family		xanthine/uracil
HPPR_01096	Nramp	The Metal Ion (Mn2+-iron) Transporter (Nramp) Family		manganese/iron ion
HPPR_00194	NSS	The Neurotransmitter:Sodium Symporter (NSS) Family		sodium ion:solute symporter

HPPR_00195	NSS	The Neurotransmitter:Sodium Symporter (NSS) Family		sodium ion:solute symporter
HPPR_00328	NSS	The Neurotransmitter:Sodium Symporter (NSS) Family		sodium ion:solute symporter
HPPR_00618	Oxa1	The Cytochrome Oxidase Biogenesis (Oxa1) Family		60 KD inner membrane protein OxaA homolog
HPPR_01043	Oxa1	The Cytochrome Oxidase Biogenesis (Oxa1) Family		60 KD inner membrane protein OxaA homolog
HPPR_00130	P-ATPase	The P-type ATPase (P-ATPase) Superfamily		zinc/cadmium/cobalt ion
HPPR_00163	POT	The Proton-dependent Oligopeptide Transporter (POT) Family		proton:dipeptide/tripeptide symporter
HPPR_00116	RND	The Resistance-Nodulation-Cell Division (RND) Superfamily	SecDF	protein export (SecDF)
HPPR_01169	RND	The Resistance-Nodulation-Cell Division (RND) Superfamily	HAE1	multidrug/solvent efflux (HAE1 subfamily)
HPPR_00058	SSPTS	Sugar Specific PTS	EnzymeIIABC	glucose/maltose/N-acetylglucosamine
HPPR_00324	SSPTS	Sugar Specific PTS	EnzymeIIABC	glucose/maltose/N-acetylglucosamine
HPPR_00491	SSPTS	Sugar Specific PTS	EnzymeIIABC	mannitol
HPPR_00943	SSPTS	Sugar Specific PTS	EnzymeIIABC	glucose/maltose/N-acetylglucosamine
HPPR_01172	SSPTS	Sugar Specific PTS	EnzymeIIABC	fructose
HPPR_00018	SSS	The Solute:Sodium Symporter (SSS) Family		sodium ion:proline symporter
HPPR_00433	Tat	The Twin Arginine Targeting (Tat) Family		protein export
HPPR_00984	Tat	The Twin Arginine Targeting (Tat) Family		protein export
HPPR_00986	Tat	The Twin Arginine Targeting (Tat) Family		protein export
HPPR_01182	TDT	The Telurite-resistance/Dicarboxylate Transporter (TDT) Family		tellurite
HPPR_00208	TerC	The Tellurium Ion Resistance (TerC) Family		tellurium ion efflux
HPPR_01087	Trk	The K+ Transporter (Trk) Family		potassium ion uptake
