# Supplementary material for: The terrestrial isopod symbiont ‘Candidatus Hepatincola porcellionum’ is a potential nutrient scavenger related to Holosporales symbionts of protists
Source: ISME Commun. 2023 Mar 8;3:18. doi: 10.1038/s43705-023-00224-w (PMC9992710; doi:10.1038/s43705-023-00224-w)
Supplement: Supplementary file 9 — Table S8 [file 43705_2023_224_MOESM9_ESM.pdf]

Secretion System	Protein	HepAv	HepPdp	HepPp
Sec Translocon	SecA	HAV_00036		HPPR_00047
Sec Translocon	SecD	HAV_00105		HPPR_00115
Sec Translocon	SecF	HAV_00106		HPPR_00116
Sec Translocon	SecG	HAV_00384	HPDP_00280	HPPR_00332
Sec Translocon	SecE		HPDP_01073	HPPR_01107
Sec Translocon	SecY	HAV_01192	HPDP_01106	HPPR_01141
Sec Translocon	YidC	HAV_01098	HPDP_00558, HPDP_01010	HPPR_00618, HPPR_01043
Sec Translocon	YajC	HAV_00104		HPPR_00114
Sec Translocon	LepB	HAV_00343	HPDP_00239	HPPR_00291
Twin-arginine Translocon	TatA	HAV_01038	HPDP_00946	HPPR_00984
Twin-arginine Translocon	TatB	HAV_01040	HPDP_00948	HPPR_00986
Twin-arginine Translocon	TatC	HAV_00492	HPDP_00389	HPPR_00433
T1SS	AprE/mfp	HAV_00762	HPDP_00668	HPPR_00719
T1SS	AprD/ATPase	HAV_00763	HPDP_00669	HPPR_00720
T1SS	TolC	HAV_00766	HPDP_00672	HPPR_00723
T1SS	TolC	HAV_01219	HPDP_01133	HPPR_01170
T4SS	VirB1			
T4SS	VirB2	HAV_00151		
T4SS	VirB3	HAV_00153		
T4SS	VirB4	HAV_00154		
T4SS	VirB5	HAV_00161		
T4SS	VirB6	HAV_00162		
T4SS	VirB7			
T4SS	VirB8	HAV_00163		
T4SS	VirB9	HAV_00164		
T4SS	VirB10	HAV_00165		
T4SS	VirB11	HAV_00158		
T4SS	VirD4	HAV_00167		
T5SSa	Autotransporter	HAV_00587	HPDP_00488	HPPR_00550
T5SSa	BamA	HAV_00484	HPDP_00381	HPPR_00426
T5SSa	BamB	HAV_00039		HPPR_00050
T5SSa	BamD	HAV_00633	HPDP_00537	HPPR_00597
T5SSa	BamE	HAV_00465	HPDP_00367	HPPR_00411
T5SSa	skp/ompH	HAV_00480	HPDP_00379	HPPR_00424
T5SSa	SurA	HAV_01052	HPDP_00960	HPPR_00999
