# Supplementary material for: The terrestrial isopod symbiont ‘Candidatus Hepatincola porcellionum’ is a potential nutrient scavenger related to Holosporales symbionts of protists
Source: ISME Commun. 2023 Mar 8;3:18. doi: 10.1038/s43705-023-00224-w (PMC9992710; doi:10.1038/s43705-023-00224-w)
Supplement: Supplementary file 10 — Table S9 [file 43705_2023_224_MOESM10_ESM.pdf]

Gene	Signal Peptide	Annotation
HAV_00022	SP(Sec/SPI)	Hypothetical protein
HAV_00025	SP(Sec/SPI)	Outer membrane beta-barrel protein
HAV_00039	SP(Sec/SPI)	Outer membrane protein assembly factor bamB
HAV_00050	SP(Sec/SPI)	Ribosome-inactivating protein
HAV_00051	SP(Sec/SPI)	Hypothetical protein
HAV_00059	SP(Sec/SPI)	Outer membrane lipoprotein-sorting protein
HAV_00063	SP(Sec/SPI)	Hypothetical protein
HAV_00069	SP(Sec/SPI)	Hypothetical protein
HAV_00078	SP(Sec/SPI)	Hypothetical protein
HAV_00098	LIPO(Sec/SPII)	ABC transporter substrate-binding protein
HAV_00103	SP(Sec/SPI)	Peptidoglycan DD-metalloendopeptidase family protein
HAV_00111	SP(Sec/SPI)	Zinc-uptake complex periplasmic component A
HAV_00112	SP(Sec/SPI)	Zinc ABC transporter substrate-binding protein
HAV_00134	SP(Sec/SPI)	Thiol:disulfide interchange protein dsbD
HAV_00138	SP(Sec/SPI)	Hypothetical protein
HAV_00143	SP(Sec/SPI)	Gram-negative porin
HAV_00144	SP(Sec/SPI)	hlyC/corC family transporter
HAV_00160	LIPO(Sec/SPII)	Hypothetical protein
HAV_00161	SP(Sec/SPI)	virB5 protein family
HAV_00162	SP(Sec/SPI)	trbL/virB6 family protein
HAV_00164	SP(Sec/SPI)	trbG/virB9 family protein
HAV_00165	SP(Sec/SPI)	trbI/virB10 family protein
HAV_00166	SP(Sec/SPI)	Hypothetical protein
HAV_00172	SP(Sec/SPI)	Hypothetical protein
HAV_00181	LIPO(Sec/SPII)	Cytochrome bo(3) ubiquinol oxidase subunit 2 (cyoA)
HAV_00187	LIPO(Sec/SPII)	Murein DD-endopeptidase mepH
HAV_00207	SP(Sec/SPI)	HAD family hydrolase
HAV_00208	LIPO(Sec/SPII)	Hypothetical protein
HAV_00219	LIPO(Sec/SPII)	OmpA family protein
HAV_00223	SP(Sec/SPI)	OmpA family protein
HAV_00227	SP(Sec/SPI)	Hypothetical protein
HAV_00230	SP(Sec/SPI)	Hypothetical protein
HAV_00255	LIPO(Sec/SPII)	Putative alkyl/aryl sulfatase yjcS
HAV_00262	SP(Sec/SPI)	Hypothetical protein
HAV_00268	SP(Sec/SPI)	Outer membrane protein beta-barrel domain protein
HAV_00269	SP(Sec/SPI)	Outer membrane protein beta-barrel domain protein
HAV_00271	SP(Sec/SPI)	Endolytic peptidoglycan transglycosylase rlpA
HAV_00272	SP(Sec/SPI)	D-alanyl-D-alanine carboxypeptidase dacA
HAV_00278	SP(Sec/SPI)	D-alanyl-D-alanine carboxypeptidase
HAV_00280	SP(Sec/SPI)	NADH-ubiquinone oxidoreductase subunit NDUFA12
HAV_00283	SP(Sec/SPI)	DUF2155 domain-containing protein
HAV_00284	SP(Sec/SPI)	NLP/P60-like protein
HAV_00314	LIPO(Sec/SPII)	Putative phage protein
HAV_00334	SP(Sec/SPI)	Metallo-beta-lactamase type 2
HAV_00338	SP(Sec/SPI)	Hypothetical protein
HAV_00347	SP(Sec/SPI)	Thiol-disulfide oxidoreductase resA
HAV_00352	SP(Sec/SPI)	Hypothetical protein
HAV_00365	SP(Sec/SPI)	Methionine-binding lipoprotein metQ
HAV_00366	SP(Sec/SPI)	Methionine-binding lipoprotein metQ

HAV_00399	SP(Sec/SPI)	Hypothetical protein
HAV_00406	LIPO(Sec/SPII)	Trl-like protein
HAV_00414	LIPO(Sec/SPII)	Hypothetical protein
HAV_00427	LIPO(Sec/SPII)	Hypothetical protein
HAV_00441	SP(Sec/SPI)	Flavodoxin
HAV_00442	SP(Sec/SPI)	4-carboxymuconolactone decarboxylase
HAV_00447	LIPO(Sec/SPII)	Hypothetical protein
HAV_00449	SP(Sec/SPI)	Hypothetical protein
HAV_00460	LIPO(Sec/SPII)	Aldo-keto reductase iolS
HAV_00461	LIPO(Sec/SPII)	Aldo-keto reductase iolS
HAV_00465	LIPO(Sec/SPII)	Outer membrane protein assembly factor bamE
HAV_00471	SP(Sec/SPI)	DUF3604 domain-containing protein
HAV_00473	SP(Sec/SPI)	hupE/ureJ family protein
HAV_00480	SP(Sec/SPI)	OmpH family outer membrane protein (skp)
HAV_00502	LIPO(Sec/SPII)	Hypothetical protein
HAV_00518	SP(Sec/SPI)	Nucleoside-specific channel-forming protein
HAV_00542	SP(Sec/SPI)	Hypothetical protein
HAV_00544	SP(Sec/SPI)	Hypothetical protein
HAV_00546	LIPO(Sec/SPII)	Hypothetical protein
HAV_00553	LIPO(Sec/SPII)	Hypothetical protein
HAV_00555	SP(Sec/SPI)	Hypothetical protein
HAV_00557	SP(Sec/SPI)	Hypothetical protein
HAV_00558	SP(Sec/SPI)	DUF1509 domain-containing protein
HAV_00566	SP(Sec/SPI)	Class B acid phosphatase aphA
HAV_00573	SP(Sec/SPI)	ABC transporter substrate-binding protein
HAV_00587	SP(Sec/SPI)	Autotransporter beta-domain protein
HAV_00604	SP(Sec/SPI)	N-acetylmuramoyl-L-alanine amidase
HAV_00606	SP(Sec/SPI)	Beta-barrel assembly-enhancing protease bepA
HAV_00607	SP(Sec/SPI)	Outer membrane protein beta-barrel protein
HAV_00624	SP(Sec/SPI)	Hypothetical protein
HAV_00633	LIPO(Sec/SPII)	Outer membrane protein assembly factor bamD
HAV_00674	LIPO(Sec/SPII)	Hypothetical protein
HAV_00680	LIPO(Sec/SPII)	Hypothetical protein
HAV_00683	SP(Sec/SPI)	Cytochrome B5
HAV_00684	SP(Sec/SPI)	Hypothetical protein
HAV_00736	SP(Sec/SPI)	META domain-containing protein
HAV_00743	SP(Sec/SPI)	Membrane-bound lytic murein transglycosylase C (mltC)
HAV_00750	SP(Sec/SPI)	Endonuclease YhcR
HAV_00755	SP(Sec/SPI)	Disulfide bond formation protein D
HAV_00766	SP(Sec/SPI)	Outer membrane protein TolC
HAV_00788	SP(Sec/SPI)	Chromosome segregation ATPase
HAV_00789	LIPO(Sec/SPII)	Peptidoglycan-associated lipoprotein pal
HAV_00807	SP(Sec/SPI)	Hypothetical protein
HAV_00816	SP(Sec/SPI)	Peptidase S41
HAV_00834	SP(Sec/SPI)	Outer membrane lipoprotein carrier protein lolA
HAV_00837	SP(Sec/SPI)	Hypothetical protein
HAV_00838	SP(Sec/SPI)	DUF3576 domain-containing protein
HAV_00840	SP(Sec/SPI)	Hypothetical protein
HAV_00844	LIPO(Sec/SPII)	Hypothetical protein
HAV_00856	LIPO(Sec/SPII)	Hypothetical protein

HAV_00863	SP(Sec/SPI)	Hypothetical protein
HAV_00884	LIPO(Sec/SPII)	Hypothetical protein
HAV_00926	SP(Sec/SPI)	Hypothetical protein
HAV_00927	SP(Sec/SPI)	Porin family protein
HAV_00930	SP(Sec/SPI)	Hypothetical protein
HAV_00931	LIPO(Sec/SPII)	Hypothetical protein
HAV_00944	SP(Sec/SPI)	Hypothetical protein
HAV_00961	SP(Sec/SPI)	Phosphate ABC transporter substrate-binding protein
HAV_00966	SP(Sec/SPI)	Enoyl-CoA hydratase
HAV_00982	LIPO(Sec/SPII)	Penicillin-binding protein activator lpoA
HAV_01007	SP(Sec/SPI)	Extracellular solute-binding protein
HAV_01009	LIPO(Sec/SPII)	Inner membrane ABC transporter permease protein yejE
HAV_01016	SP(Sec/SPI)	Phosphopentomutase deoB
HAV_01027	SP(Sec/SPI)	Chitinase A1
HAV_01028	SP(Sec/SPI)	Glycoside hydrolase family 18
HAV_01052	LIPO(Sec/SPII)	Chaperone SurA
HAV_01053	SP(Sec/SPI)	LPS-assembly protein lptD
HAV_01067	LIPO(Sec/SPII)	Lipoprotein
HAV_01068	LIPO(Sec/SPII)	Hypothetical protein
HAV_01069	LIPO(Sec/SPII)	Penicillin-binding protein activator lpoB
HAV_01070	SP(Sec/SPI)	LPP20 family protein
HAV_01091	SP(Sec/SPI)	Lipid carrier protein
HAV_01094	LIPO(Sec/SPII)	Hypothetical protein
HAV_01116	SP(Sec/SPI)	CDP-alcohol phosphatidyltransferase
HAV_01126	LIPO(Sec/SPII)	Hypothetical protein
HAV_01127	LIPO(Sec/SPII)	Hypothetical protein
HAV_01128	LIPO(Sec/SPII)	Hypothetical protein
HAV_01129	SP(Sec/SPI)	Hypothetical protein
HAV_01145	SP(Sec/SPI)	Hypothetical protein
HAV_01146	SP(Sec/SPI)	Porin superfamily protein
HAV_01199	SP(Sec/SPI)	DegQ family serine endoprotease
HAV_01200	SP(Sec/SPI)	Hypothetical protein
HAV_01215	SP(Sec/SPI)	Peptidylprolyl isomerase

Gene	Signal Peptide	Annotation
HPDP_00022	SP(Sec/SPI)	Hypothetical protein
HPDP_00025	SP(Sec/SPI)	Outer membrane beta-barrel protein
HPDP_00031	SP(Sec/SPI)	Zinc-uptake complex periplasmic component A
HPDP_00032	SP(Sec/SPI)	Zinc ABC transporter substrate-binding protein
HPDP_00057	SP(Sec/SPI)	Hypothetical protein
HPDP_00062	SP(Sec/SPI)	Gram-negative porin
HPDP_00063	SP(Sec/SPI)	hlyC/corC family transporter
HPDP_00075	LIPO(Sec/SPII)	Cytochrome bo(3) ubiquinol oxidase subunit 2 (cyoA)
HPDP_00081	LIPO(Sec/SPII)	Murein DD-endopeptidase mepH
HPDP_00101	SP(Sec/SPI)	HAD family hydrolase
HPDP_00102	LIPO(Sec/SPII)	Hypothetical protein
HPDP_00113	LIPO(Sec/SPII)	OmpA family protein
HPDP_00117	SP(Sec/SPI)	OmpA family protein
HPDP_00121	SP(Sec/SPI)	Hypothetical protein
HPDP_00124	SP(Sec/SPI)	OmpA family protein
HPDP_00150	LIPO(Sec/SPII)	Putative alkyl/aryl-sulfatase yjcS
HPDP_00158	SP(Sec/SPI)	Hypothetical protein
HPDP_00164	SP(Sec/SPI)	Outer membrane protein beta-barrel domain protein
HPDP_00165	SP(Sec/SPI)	Outer membrane protein beta-barrel domain protein
HPDP_00167	SP(Sec/SPI)	Endolytic peptidoglycan transglycosylase rlpA
HPDP_00168	SP(Sec/SPI)	D-alanyl-D-alanine carboxypeptidase dacA
HPDP_00176	SP(Sec/SPI)	D-alanyl-D-alanine carboxypeptidase
HPDP_00178	SP(Sec/SPI)	NADH-ubiquinone oxidoreductase subunit NDUFA12
HPDP_00181	SP(Sec/SPI)	DUF2155 domain-containing protein
HPDP_00183	SP(Sec/SPI)	NLP/P60-like protein
HPDP_00226	SP(Sec/SPI)	Metallo-beta-lactamase type 2
HPDP_00234	SP(Sec/SPI)	Hypothetical protein
HPDP_00243	SP(Sec/SPI)	Thiol-disulfide oxidoreductase resA
HPDP_00248	SP(Sec/SPI)	Hypothetical protein
HPDP_00261	SP(Sec/SPI)	Methionine-binding lipoprotein metQ
HPDP_00262	SP(Sec/SPI)	Methionine-binding lipoprotein metQ
HPDP_00297	SP(Sec/SPI)	Hypothetical protein
HPDP_00303	LIPO(Sec/SPII)	TRL-like protein
HPDP_00307	SP(Sec/SPI)	Lipoyl synthase LipA
HPDP_00313	LIPO(Sec/SPII)	Hypothetical protein
HPDP_00327	LIPO(Sec/SPII)	Hypothetical protein
HPDP_00328	SP(Sec/SPI)	Hypothetical protein
HPDP_00343	SP(Sec/SPI)	Flavodoxin
HPDP_00344	SP(Sec/SPI)	4-carboxymuconolactone decarboxylase
HPDP_00349	LIPO(Sec/SPII)	Hypothetical protein
HPDP_00351	SP(Sec/SPI)	Hypothetical protein
HPDP_00363	LIPO(Sec/SPII)	Aldo-keto reductase iolS
HPDP_00367	LIPO(Sec/SPII)	Outer membrane protein assembly factor bamE
HPDP_00379	SP(Sec/SPI)	OmpH family outer membrane protein (skp)
HPDP_00397	LIPO(Sec/SPII)	Hypothetical protein
HPDP_00413	SP(Sec/SPI)	Nucleoside-specific channel-forming protein
HPDP_00427	LIPO(Sec/SPII)	Hypothetical protein
HPDP_00440	SP(Sec/SPI)	Hypothetical protein
HPDP_00443	SP(Sec/SPI)	Hypothetical protein

HPDP_00447	LIPO(Sec/SPII)	Hypothetical protein
HPDP_00454	SP(Sec/SPI)	Hypothetical protein
HPDP_00456	SP(Sec/SPI)	Hypothetical protein
HPDP_00457	SP(Sec/SPI)	DUF1509 domain-containing protein
HPDP_00465	SP(Sec/SPI)	Class B acid phosphatase aphA
HPDP_00473	SP(Sec/SPI)	ABC transporter substrate-binding protein
HPDP_00488	SP(Sec/SPI)	Autotransporter beta-domain protein
HPDP_00505	SP(Sec/SPI)	N-acetylmuramoyl-L-alanine amidase
HPDP_00507	SP(Sec/SPI)	Beta-barrel assembly-enhancing protease bepA
HPDP_00509	SP(Sec/SPI)	Outer membrane protein beta-barrel protein
HPDP_00526	SP(Sec/SPI)	Hypothetical protein
HPDP_00529	SP(Sec/SPI)	Hypothetical protein
HPDP_00537	LIPO(Sec/SPII)	Outer membrane protein assembly factor bamD
HPDP_00580	LIPO(Sec/SPII)	Hypothetical protein
HPDP_00587	LIPO(Sec/SPII)	Hypothetical protein
HPDP_00590	SP(Sec/SPI)	Cytochrome B5
HPDP_00591	SP(Sec/SPI)	Hypothetical protein
HPDP_00642	SP(Sec/SPI)	META domain-containing protein
HPDP_00649	SP(Sec/SPI)	Membrane-bound lytic murein transglycosylase C (mltC)
HPDP_00656	SP(Sec/SPI)	Endonuclease YhcR
HPDP_00661	SP(Sec/SPI)	Disulfide bond formation protein D
HPDP_00672	SP(Sec/SPI)	Outer membrane protein TolC
HPDP_00695	SP(Sec/SPI)	Chromosome partition protein Smc
HPDP_00696	LIPO(Sec/SPII)	Peptidoglycan-associated lipoprotein pal
HPDP_00723	SP(Sec/SPI)	Peptidase S41
HPDP_00741	SP(Sec/SPI)	Outer membrane lipoprotein carrier protein lolA
HPDP_00744	SP(Sec/SPI)	Hypothetical protein
HPDP_00745	SP(Sec/SPI)	DUF3576 domain-containing protein
HPDP_00747	LIPO(Sec/SPII)	Hypothetical protein
HPDP_00751	LIPO(Sec/SPII)	Hypothetical protein
HPDP_00769	SP(Sec/SPI)	Hypothetical protein
HPDP_00790	LIPO(Sec/SPII)	Hypothetical protein
HPDP_00832	SP(Sec/SPI)	Hypothetical protein
HPDP_00834	SP(Sec/SPI)	Porin family protein
HPDP_00837	SP(Sec/SPI)	Hypothetical protein
HPDP_00838	LIPO(Sec/SPII)	Hypothetical protein
HPDP_00848	SP(Sec/SPI)	Hypothetical protein
HPDP_00872	SP(Sec/SPI)	Enoyl-CoA hydratase
HPDP_00888	LIPO(Sec/SPII)	Penicillin-binding protein activator lpoA
HPDP_00914	SP(Sec/SPI)	Extracellular solute-binding protein
HPDP_00916	SP(Sec/SPI)	Inner membrane ABC transporter permease protein yejE
HPDP_00923	SP(Sec/SPI)	Phosphopentomutase deoB
HPDP_00934	SP(Sec/SPI)	Chitinase A1
HPDP_00935	SP(Sec/SPI)	Glycoside hydrolase family 18
HPDP_00960	LIPO(Sec/SPII)	Chaperone SurA
HPDP_00961	SP(Sec/SPI)	LPS-assembly protein lptD
HPDP_00975	LIPO(Sec/SPII)	Lipoprotein
HPDP_00976	LIPO(Sec/SPII)	Hypothetical protein
HPDP_00977	LIPO(Sec/SPII)	Penicillin-binding protein activator lpoB
HPDP_00978	SP(Sec/SPI)	LPP20 family protein

HPDP_01003	SP(Sec/SPI)	Lipid carrier protein
HPDP_01006	LIPO(Sec/SPII)	Hypothetical protein
HPDP_01028	SP(Sec/SPI)	CDP-alcohol phosphatidyltransferase
HPDP_01038	LIPO(Sec/SPII)	Hypothetical protein
HPDP_01039	LIPO(Sec/SPII)	Hypothetical protein
HPDP_01040	LIPO(Sec/SPII)	Hypothetical protein
HPDP_01056	SP(Sec/SPI)	Hypothetical protein
HPDP_01057	SP(Sec/SPI)	Porin superfamily protein
HPDP_01113	SP(Sec/SPI)	DegQ family serine endoprotease
HPDP_01114	SP(Sec/SPI)	Hypothetical protein
HPDP_01133	SP(Sec/SPI)	Outer membrane protein TolC

Gene	Signal Peptide	Annotation
HPPR_00033	LIPO(Sec/SPII)	Hypothetical protein
HPPR_00036	SP(Sec/SPI)	Outer membrane beta-barrel protein
HPPR_00050	SP(Sec/SPI)	Outer membrane protein assembly factor bamB
HPPR_00061	SP(Sec/SPI)	Ribosome-inactivating protein
HPPR_00070	SP(Sec/SPI)	Outer membrane lipoprotein-sorting protein
HPPR_00074	SP(Sec/SPI)	Hypothetical protein
HPPR_00081	SP(Sec/SPI)	Hypothetical protein
HPPR_00087	LIPO(Sec/SPII)	Hypothetical protein
HPPR_00108	LIPO(Sec/SPII)	ABC transporter substrate-binding protein
HPPR_00113	SP(Sec/SPI)	Peptidoglycan DD-metalloendopeptidase family protein
HPPR_00120	SP(Sec/SPI)	Zinc-uptake complex periplasmic component A
HPPR_00121	SP(Sec/SPI)	Zinc ABC transporter substrate-binding protein
HPPR_00140	SP(Sec/SPI)	Thiol:disulfide interchange protein dsbD
HPPR_00142	SP(Sec/SPI)	Mechanosensitive ion channel
HPPR_00144	SP(Sec/SPI)	Octaprenyl diphosphate synthase
HPPR_00149	SP(Sec/SPI)	Gram-negative porin
HPPR_00150	SP(Sec/SPI)	hlyC/corC family transporter
HPPR_00158	LIPO(Sec/SPII)	Cytochrome bo(3) ubiquinol oxidase subunit 2 (cyoA)
HPPR_00185	SP(Sec/SPI)	HAD family hydrolase
HPPR_00186	LIPO(Sec/SPII)	Hypothetical protein
HPPR_00197	LIPO(Sec/SPII)	OmpA family protein
HPPR_00202	SP(Sec/SPI)	OmpA family protein
HPPR_00206	SP(Sec/SPI)	Hypothetical protein
HPPR_00209	SP(Sec/SPI)	Hypothetical protein
HPPR_00233	LIPO(Sec/SPII)	Putative alkyl/aryl sulfatase yjcS
HPPR_00239	SP(Sec/SPI)	Hypothetical protein
HPPR_00244	LIPO(Sec/SPII)	Hypothetical protein
HPPR_00246	SP(Sec/SPI)	Outer membrane protein beta-barrel domain protein
HPPR_00247	SP(Sec/SPI)	Outer membrane protein beta-barrel domain protein
HPPR_00249	SP(Sec/SPI)	Endolytic peptidoglycan transglycosylase rlpA
HPPR_00250	SP(Sec/SPI)	D-alanyl-D-alanine carboxypeptidase dacA
HPPR_00256	SP(Sec/SPI)	D-alanyl-D-alanine carboxypeptidase
HPPR_00261	SP(Sec/SPI)	Hypothetical protein
HPPR_00263	SP(Sec/SPI)	NLP/P60-like protein
HPPR_00280	LIPO(Sec/SPII)	Metallo-beta-lactamase type 2
HPPR_00286	SP(Sec/SPI)	Hypothetical protein
HPPR_00295	SP(Sec/SPI)	Thiol-disulfide oxidoreductase resA
HPPR_00300	SP(Sec/SPI)	Hypothetical protein
HPPR_00314	SP(Sec/SPI)	Methionine-binding lipoprotein metQ
HPPR_00315	SP(Sec/SPI)	Methionine-binding lipoprotein metQ
HPPR_00346	SP(Sec/SPI)	Hypothetical protein
HPPR_00347	SP(Sec/SPI)	Hypothetical protein
HPPR_00350	LIPO(Sec/SPII)	Hypothetical protein
HPPR_00352	LIPO(Sec/SPII)	Trl-like protein
HPPR_00359	SP(Sec/SPI)	Trigger factor
HPPR_00360	LIPO(Sec/SPII)	Hypothetical protein
HPPR_00374	LIPO(Sec/SPII)	Hypothetical protein
HPPR_00388	SP(Sec/SPI)	Putative alkyl/aryl-sulfatase YjcS
HPPR_00395	SP(Sec/SPI)	Hypothetical protein

HPPR_00411	LIPO(Sec/SPII)	Outer membrane protein assembly factor bamE
HPPR_00424	SP(Sec/SPI)	OmpH family outer membrane protein (skp)
HPPR_00438	LIPO(Sec/SPII)	Hypothetical protein
HPPR_00441	LIPO(Sec/SPII)	Hypothetical protein
HPPR_00456	SP(Sec/SPI)	Nucleoside-specific channel-forming protein
HPPR_00480	SP(Sec/SPI)	Hypothetical protein
HPPR_00481	LIPO(Sec/SPII)	Hypothetical protein
HPPR_00482	SP(Sec/SPI)	Hypothetical protein
HPPR_00492	SP(Sec/SPI)	Hypothetical protein
HPPR_00493	SP(Sec/SPI)	Hypothetical protein
HPPR_00494	SP(Sec/SPI)	Hypothetical protein
HPPR_00501	SP(Sec/SPI)	Class B acid phosphatase aphA
HPPR_00518	SP(Sec/SPI)	Hypothetical protein
HPPR_00535	SP(Sec/SPI)	ABC transporter substrate-binding protein
HPPR_00537	SP(Sec/SPI)	Hypothetical protein
HPPR_00550	SP(Sec/SPI)	Autotransporter beta-domain protein
HPPR_00567	SP(Sec/SPI)	N-acetylmuramoyl-L-alanine amidase
HPPR_00570	SP(Sec/SPI)	Outer membrane protein beta-barrel protein
HPPR_00588	SP(Sec/SPI)	Hypothetical protein
HPPR_00591	SP(Sec/SPI)	Hypothetical protein
HPPR_00597	LIPO(Sec/SPII)	Outer membrane protein assembly factor bamD
HPPR_00637	SP(Sec/SPI)	Zinc protease
HPPR_00638	LIPO(Sec/SPII)	Hypothetical protein
HPPR_00644	SP(Sec/SPI)	Hypothetical protein
HPPR_00647	SP(Sec/SPI)	Cytochrome B5
HPPR_00680	SP(Sec/SPI)	Hemin receptor hemR
HPPR_00693	SP(Sec/SPI)	META domain-containing protein
HPPR_00700	SP(Sec/SPI)	Membrane-bound lytic murein transglycosylase C (mltC)
HPPR_00707	SP(Sec/SPI)	Endonuclease YhcR
HPPR_00712	SP(Sec/SPI)	Disulfide bond formation protein D
HPPR_00723	SP(Sec/SPI)	Outer membrane protein TolC
HPPR_00734	SP(Sec/SPI)	Hypothetical protein
HPPR_00746	SP(Sec/SPI)	Chromosome segregation ATPase
HPPR_00747	LIPO(Sec/SPII)	Peptidoglycan-associated lipoprotein pal
HPPR_00748	SP(Sec/SPI)	TolB
HPPR_00774	SP(Sec/SPI)	Peptidase S41
HPPR_00792	SP(Sec/SPI)	Outer membrane lipoprotein carrier protein lolA
HPPR_00795	SP(Sec/SPI)	Hypothetical protein
HPPR_00796	SP(Sec/SPI)	Hypothetical protein
HPPR_00798	LIPO(Sec/SPII)	Hypothetical protein
HPPR_00819	SP(Sec/SPI)	Fladoxin
HPPR_00839	LIPO(Sec/SPII)	Beta sliding clamp protein
HPPR_00881	SP(Sec/SPI)	Hypothetical protein
HPPR_00882	SP(Sec/SPI)	Porin family protein
HPPR_00883	SP(Sec/SPI)	Hypothetical protein
HPPR_00885	SP(Sec/SPI)	Porin family protein
HPPR_00886	LIPO(Sec/SPII)	Hypothetical protein
HPPR_00896	SP(Sec/SPI)	Hypothetical protein
HPPR_00900	LIPO(Sec/SPII)	Hypothetical protein
HPPR_00901	LIPO(Sec/SPII)	Hypothetical protein

HPPR_00912	SP(Sec/SPI)	Phosphate ABC transporter substrate-binding protein
HPPR_00917	SP(Sec/SPI)	Enoyl-CoA hydratase
HPPR_00933	LIPO(Sec/SPII)	Penicillin-binding protein activator lpoA
HPPR_00951	SP(Sec/SPI)	Hypothetical protein
HPPR_00954	SP(Sec/SPI)	Extracellular solute-binding protein
HPPR_00956	LIPO(Sec/SPII)	Inner membrane ABC transporter permease protein yejE
HPPR_00974	SP(Sec/SPI)	Glycoside hydrolase family 18
HPPR_00999	SP(Sec/SPI)	Chaperone SurA
HPPR_01000	SP(Sec/SPI)	LPS-assembly protein lptD
HPPR_01007	SP(Sec/SPI)	Hypothetical protein
HPPR_01014	LIPO(Sec/SPII)	Lipoprotein
HPPR_01015	LIPO(Sec/SPII)	Hypothetical protein
HPPR_01016	LIPO(Sec/SPII)	Penicillin-binding protein activator lpoB
HPPR_01017	SP(Sec/SPI)	LPP20 family protein
HPPR_01040	LIPO(Sec/SPII)	Hypothetical protein
HPPR_01061	SP(Sec/SPI)	CDP-alcohol phosphatidyltransferase
HPPR_01071	LIPO(Sec/SPII)	Hypothetical protein
HPPR_01072	LIPO(Sec/SPII)	Hypothetical protein
HPPR_01073	LIPO(Sec/SPII)	Hypothetical protein
HPPR_01074	SP(Sec/SPI)	Hypothetical protein
HPPR_01090	SP(Sec/SPI)	Hypothetical protein
HPPR_01091	SP(Sec/SPI)	Porin superfamily protein
HPPR_01101	SP(Sec/SPI)	Putative L-asparaginase ansA
HPPR_01148	SP(Sec/SPI)	DegQ family serine endoprotease
HPPR_01149	SP(Sec/SPI)	Hypothetical protein
HPPR_01155	LIPO(Sec/SPII)	Hypothetical protein
HPPR_01166	SP(Sec/SPI)	Peptidylprolyl isomerase
HPPR_01170	SP(Sec/SPI)	Outer membrane protein TolC
